# Site-selective photocatalytic functionalization of peptides and proteins at selenocysteine

**DOI:** 10.1038/s41467-022-34530-z

**Published:** 2022-11-12

**Authors:** Luke J. Dowman, Sameer S. Kulkarni, Juan V. Alegre-Requena, Andrew M. Giltrap, Alexander R. Norman, Ashish Sharma, Liliana C. Gallegos, Angus S. Mackay, Adarshi P. Welegedara, Emma E. Watson, Damian van Raad, Gerhard Niederacher, Susanne Huhmann, Nicholas Proschogo, Karishma Patel, Mark Larance, Christian F. W. Becker, Joel P. Mackay, Girish Lakhwani, Thomas Huber, Robert S. Paton, Richard J. Payne

**Affiliations:** 1 School of Chemistry, The University of Sydney, Sydney, NSW 2006 Australia; 2 Australian Research Council Centre of Excellence for Innovations in Peptide and Protein Science, The University of Sydney, Sydney, NSW 2006 Australia; 3 Department of Chemistry, Colorado State University, Fort Collins, CO 80523-1872 USA; 4 Australian Research Council Centre of Excellence in Exciton Science, The University of Sydney, Sydney, NSW 2006 Australia; 5 Research School of Chemistry, Australian National University, Canberra, ACT 2601 Australia; 6 Institute of Biological Chemistry, Faculty of Chemistry, University of Vienna, Vienna, Austria; 7 School of Life and Environmental Sciences, The University of Sydney, Sydney, NSW 2006 Australia; 8 Charles Perkins Centre and School of Medical Sciences, The University of Sydney, Sydney, NSW 2006 Australia

**Keywords:** Synthetic chemistry methodology, Proteins, Peptides

## Abstract

The importance of modified peptides and proteins for applications in drug discovery, and for illuminating biological processes at the molecular level, is fueling a demand for efficient methods that facilitate the precise modification of these biomolecules. Herein, we describe the development of a photocatalytic method for the rapid and efficient dimerization and site-specific functionalization of peptide and protein diselenides. This methodology, dubbed the photocatalytic diselenide contraction, involves irradiation at 450 nm in the presence of an iridium photocatalyst and a phosphine and results in rapid and clean conversion of diselenides to reductively stable selenoethers. A mechanism for this photocatalytic transformation is proposed, which is supported by photoluminescence spectroscopy and density functional theory calculations. The utility of the photocatalytic diselenide contraction transformation is highlighted through the dimerization of selenopeptides, and by the generation of two families of protein conjugates via the site-selective modification of calmodulin containing the 21^st^ amino acid selenocysteine, and the C-terminal modification of a ubiquitin diselenide.

## Abstract (web summary)

The modification of peptides and proteins for application in drug discovery and chemical biology is currently a rapidly growing field of research. Here, the authors report a photocatalytic diselenide contraction method for the dimerization and site-specific functionalisation of peptides and protein.

## Introduction

Proteins bearing post-translational modifications (PTMs) or designer modifications, such as fluorescent tags, affinity handles and polyethylene glycol (PEG) moieties at pre-determined positions, have emerged as powerful tools to investigate key biological processes and for applications in drug discovery (e.g., for the development of biologics)^1–7^. Overexpression in bacterial hosts such as *Escherichia coli* is the cornerstone of recombinant protein production, however, these systems generally lack the enzymatic machinery capable of introducing complex eukaryotic PTMs. While modified proteins can be produced in *E. coli* by unnatural amino acid incorporation using genetic code expansion and reprogramming, e.g., amber codon (UAG) suppression technologies^8–10^, the diversity of amino acids that can be installed is largely limited to analogues of tyrosine (Tyr) and lysine (Lys) bearing small modifications to the side chains. In addition, large scale protein production with these methods can be challenging^11^. Insect or mammalian cell expression systems can instead be used to generate proteins containing native PTMs, however, these modifications are generally present as complex inseparable heterogeneous mixtures^12^, thus preventing meaningful study of the functional influence of a single PTM structure at a specific site on a protein. An alternative approach to generate homogeneously modified proteins is through synthetic or semi-synthetic means by leveraging peptide ligation chemistry^13–20^. While synthesis offers unparalleled and exquisite control over the chemical identity of the protein and the locations of modifications (both natural and unnatural), access to larger protein targets, especially via total chemical synthesis, remains a very challenging and labor-intensive task^20^.

The late-stage functionalization of recombinant proteins has also emerged as a powerful approach to access site-specifically modified proteins^2,3,21,22^. The most widely employed methods for protein conjugation (and dimerization) rely on the reaction of the nucleophilic side chains of cysteine (Cys) and Lys residues with electrophilic maleimides^6^ and *N*-hydroxysuccinimidyl esters^3^, respectively. Despite the widespread use of these methods for the generation of bioconjugates, including a number of clinically approved biologics, the lack of regioselectivity of these chemistries means that, outside a handful of examples employing non-generalizable pH or surface exposure control^23,24^, isoxazolinium reagents^25^ or the use of engineered selectivity-inducing sequence motifs (e.g., π-clamp)^26^, they cannot usually be employed for the generation of homogeneous and site-specifically modified proteins^27^. To overcome the drawbacks of the above reaction manifolds, new synthetic methods have been developed for the late-stage functionalization of a specific amino acid side chain within proteins, albeit with varying degrees of chemoselectivity^3,22^. For example, cysteine (Cys) has been functionalized using a broad array of chemistries, including but not limited to the reaction with thiosulfonates^28–30^, hypervalent iodine reagents^31–33^, alkynyl phosphonoamidates^34^, strained cyclic systems^35^, desulfurative radical addition chemistry^36^, and transition-metal catalyzed arylation chemistry^37–40^. Indirect functionalization of Cys has also been performed through elimination of the sulfhydryl side chain to form dehydroalanine, with subsequent elaboration by radical or Michael addition^41–43^. Late-stage functionalization chemistries have also been developed for other proteinogenic amino acid side chains, including, but not limited to methionine (Met) using sulfur-imidation by oxaziridines^44^, hypervalent iodine reagents^45^ or photoredox catalysis^46^, serine (Ser) with phosphorous(V) reagents^47^ and Tyr using photocatalytic methods^48–51^. While each of these methods possesses exquisite chemoselectivity, all suffer from a lack of regioselectivity that arises from targeting amino acids that can occur at multiple positions within a protein sequence. It should be noted that methods have been developed for the regioselective functionalization of the N- or C-terminus of proteins under a variety of different reaction manifolds^52–58^.

Collectively, the methods outlined above provide the research community with a plethora of tools for protein modification, however, chemistry that facilitates the truly regioselective modification of proteins at any site of a protein (in addition to the termini) would be a transformative advance for the field. A number of powerful bioorthogonal chemistries^59^ (e.g., the Huisgen-type azide-alkyne cycloaddition^4,60,61^, inverse demand Diels Alder chemistry^62,63^ and the Staudinger reaction^64,65^) fit this desirable profile by providing absolute control over the site of modification. This is a result of the unique bioorthogonal functional handles employed in these reactions (e.g., azides, alkynes/cyclooctynes, tetrazines, phosphines, etc.) that exhibit minimal or no cross-reactivity with the functionalities found within the 21 proteinogenic amino acids. While some of these reactions can suffer from sluggish kinetics, and in most cases leave a large non-natural scar between the peptide backbone and the target modification, these methods have revolutionized the site-specific labeling of proteins, including within live cells^3,59^.

Selenocysteine (Sec; U) is commonly referred to as the 21st proteinogenic amino acid and is known to be incorporated into at least 25 discrete selenoproteins in humans^66^. The unique reactivity of the selenol side chain of Sec, namely its low pK_a_ and high oxidation potential and nucleophilicity relative to Cys, has led to significant interest in leveraging the amino acid for a number of applications in protein science^67,68^. While Sec has been used for bioconjugation applications, the methods developed to date have centered primarily on alkylation or arylation chemistries which often possess modest chemoselectivity over other nucleophilic side chains (e.g., Cys, Lys), poor kinetics/conversions, and/or use non-biocompatible reaction conditions^38,69–73^. Nonetheless, given that Sec is a native, yet exceptionally rare amino acid in the proteome, it remains a highly attractive candidate for serving as a linchpin for new protein modification methods with exquisite chemo- and regioselectivity, akin to the powerful biorthogonal chemistries at non-native functionalities highlighted above.

Herein, we describe the development of a photocatalytic method for the rapid and high yielding dimerization and site-specific functionalization of peptides and proteins that capitalizes on unique reactivity at Sec. Specifically, we demonstrate that in the presence of an iridium photocatalyst, a phosphine and blue LED irradiation (*λ* = 450 nm), peptide and protein diselenides can be cleanly converted to reductively stable selenoethers with the formal extrusion of a single selenium atom. We propose a mechanism for this interesting photocatalytic transformation which is supported by time-resolved photoluminescence (PL) spectroscopy, cyclic voltammetry, as well as comprehensive computational studies. The power of this reaction—called the photocatalytic diselenide contraction (PDC) reaction—is highlighted through the dimerization of several synthetic selenopeptides, and by the site-selective functionalization of recombinantly expressed proteins bearing a diselenide motif.

## Results and discussion

### Serendipitous discovery of the photocatalytic diselenide contraction (PDC) reaction

**Fig. 1:**
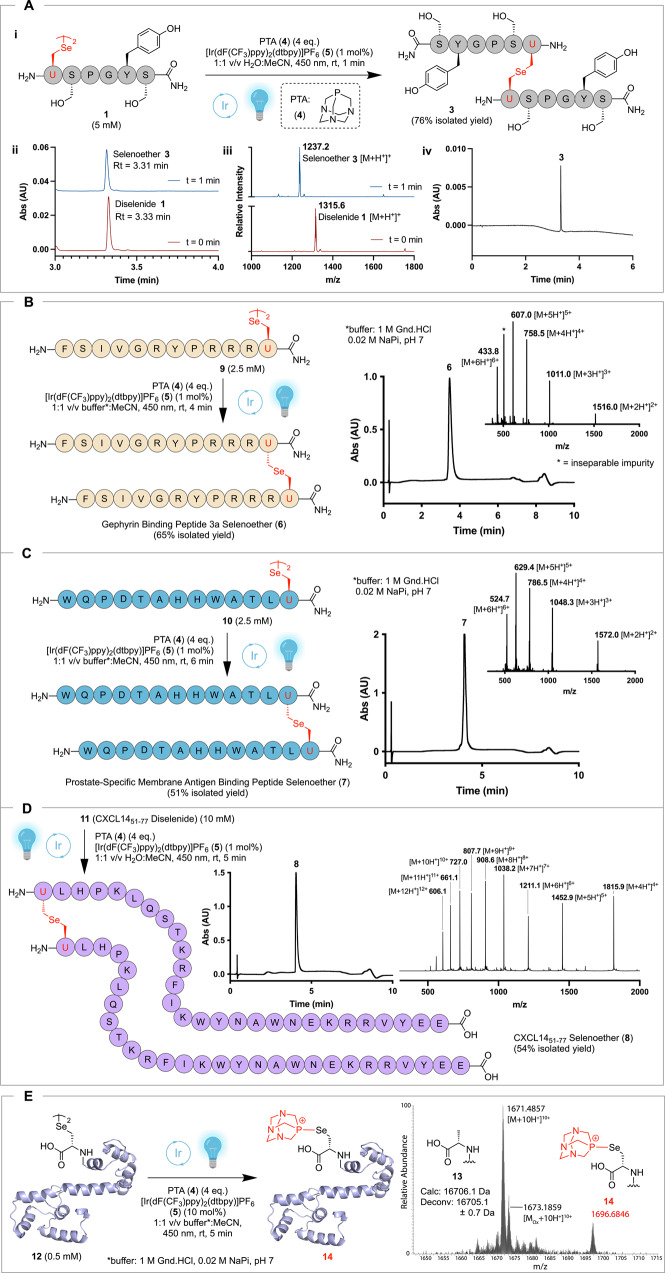
Dimerization of selenopeptides via the PDC reaction. **A** Optimization of the PDC dimerization reaction on [H_2_N-USPGYS-NH_2_]_2_ model peptide **1**. Site-specific PDC dimerization of **B** Gephyrin Binding Peptide 3a diselenide **9**; **C** Prostate-Specific Membrane Antigen (PSMA) Binding Peptide diselenide **10**; **D** CXCL14_51-77_ diselenide **11** to afford the reductively stable selenoether-linked peptide dimers **6**–**8**. Analytical HPLC and ESI-MS of the purified selenoether products data are inset. **E** Observation of selanylphosphonium **14** when CaM (K148U) diselenide **12** was submitted to optimized PDC conditions. HRMS data inset showing [M + 10H^+^]^10+^ ions of the deselenized CaM (A148) (**13**) as the major product together with selanylphosphonium adduct **14**. PTA = 1,3,5-triaza-7-phosphaadamantane, MeCN acetonitrile, AU absorbance units. Created with BioRender.com.

In our efforts to develop methods for chemical protein synthesis, we attempted to perform photocatalytic deselenization of Sec on model peptide [H_2_N-USPGYS-NH_2_]_2_ (**1**) to alanine (Ala) in H_2_N-ASPGYS-NH_2_ (**2**) using Eosin Y as the photocatalyst, the phosphine *tris*-carboxyethylphosphine (TCEP) and 450 nm blue LED light. Unexpectedly, under these conditions we observed the formation of dimeric selenoether-bridged peptide **3** as a minor side product (Supplementary Figs. 1–3). Intriguingly, generation of selenoether dimer **3** corresponds to the formal extrusion of a single selenium atom from diselenide **1**. While there have been early examples that have shown phosphine-mediated disulfide and benzylic diselenide contraction in refluxing benzene or extended UV irradiation^74,75^, to our knowledge this represents an unprecedented photocatalytic reaction at Sec. Given the potential value of dimerizing selenopeptides to reductively stable selenoether-linked dimeric peptides, together with the lack of precedent for this reaction under mild photocatalytic conditions, we sought to optimize conditions for the preferential formation of selenoether dimer **3** over the deselenization product **2**. Gratifyingly, following optimization studies on model peptide **1**, we found that treatment with 4 equivalents of the phosphine 1,3,5-triaza-7-phosphaadamantane (PTA) (**4**), and 1 mol% of the iridium photocatalyst [Ir(dF(CF_3_)ppy)_2_(dtbpy)]PF_6_ (**5**) with LED irradiation at 450 nm for 1 min, led to exclusive formation of the dimeric selenoether **3**, which could be isolated in 76% yield following reverse-phase HPLC purification (Fig. 1A, Supplementary Figs. 4–13, Supplementary Tables 1–4). Importantly, in the absence of phosphine, photocatalyst, or without 450 nm irradiation, no formation of **3** was observed (Supplementary Figs. 14–16). After verifying the identity of selenoether **3** by HRMS (Supplementary Figs. 17–20), we also confirmed the retention of stereochemical integrity at the Cα-center of the Sec residue following PDC by ^1^H and ^77^Se NMR spectroscopy (Supplementary Figs. 21–23), suggesting a reaction pathway that is mechanistically distinct to the two electron elimination-addition pathway through dehydroalanine^41,42^, including the phosphine-mediated disulfide contraction chemistry for the formation of thioether conjugates reported by Davis and co-workers^43^. Notably, the selenoether linkage of **3** was shown to be completely stable to a range of basic, acidic, and biologically relevant reducing conditions, as well as human plasma, as confirmed by analytical HPLC and mass spectrometry (Supplementary Figs. 24–26). Taken together, these experiments provide clear evidence that each of the components are essential for the transformation, and that the reaction likely proceeds through a distinct photocatalytic manifold which we have termed the photocatalytic diselenide contraction (PDC). Furthermore, the demonstrated stability of the resultant selenoether functionality opens the possibility of implementing the PDC reaction for the generation of high value conjugates with therapeutic potential.

### Site-specific dimerization of selenopeptides via PDC

With optimized conditions for the PDC transformation in hand, we next sought to demonstrate the utility of the methodology for the rapid and efficient generation of three selenoether-linked homo-dimeric target peptides that are known to exhibit improved activity in dimeric form; these included gephyrin-binding peptide 3a^76^ (GBP3a) (**6**), prostate-specific membrane antigen binding peptide^77^ (PSMABP) (**7**) and a peptide derived from the C-terminal region of the CXC chemokine ligand 14, CXCL14_51-77_ (**8**)^78^. Following synthesis of the diselenide dimer peptides **9**–**11** by solid-phase peptide synthesis (Supplementary Figs. 27–29), each was subjected to the optimized PDC conditions (vide supra) and led to excellent conversions to the corresponding selenoether-linked dimers, **6**–**8** (isolated yields: 51–65% following reverse-phase HPLC purification, Fig. 1B–D, Supplementary Figs. 30–35). To probe the limits of this PDC dimerization methodology, we next subjected a recombinant calmodulin (CaM) protein diselenide dimer (**12**), bearing a Lys-148 to Sec mutation [CaM (K148U)], to the PDC conditions (Fig. 1E). This selenoprotein was expressed using a Sec-incorporating amber-suppression platform in the evolved Sec-dependent *E. coli* β_UU3-T7 strain and employing an engineered tRNA^Sec^, based on recent reports by Ellington and co-workers (see Supplementary Figs. 36–39)^79,80^. Interestingly, when CaM (K148U) diselenide (**12**) was subjected to the optimized PDC conditions, instead of dimerization, we observed near-exclusive deselenization of Sec-148 to Ala to afford **13**, together with a species corresponding to a selanylphosphonium adduct of CaM (K148U) (**14**) (Fig. 1E, Supplementary Figs. 40 and 41). We hypothesized that this PTA adduct was a genuine intermediate in the PDC reaction pathway and therefore sought to capitalize on this finding to systematically interrogate the mechanism of the reaction using photophysical and computational experiments.

### Probing the mechanism of the PDC transformation using photophysical and computational experiments

**Fig. 2:**
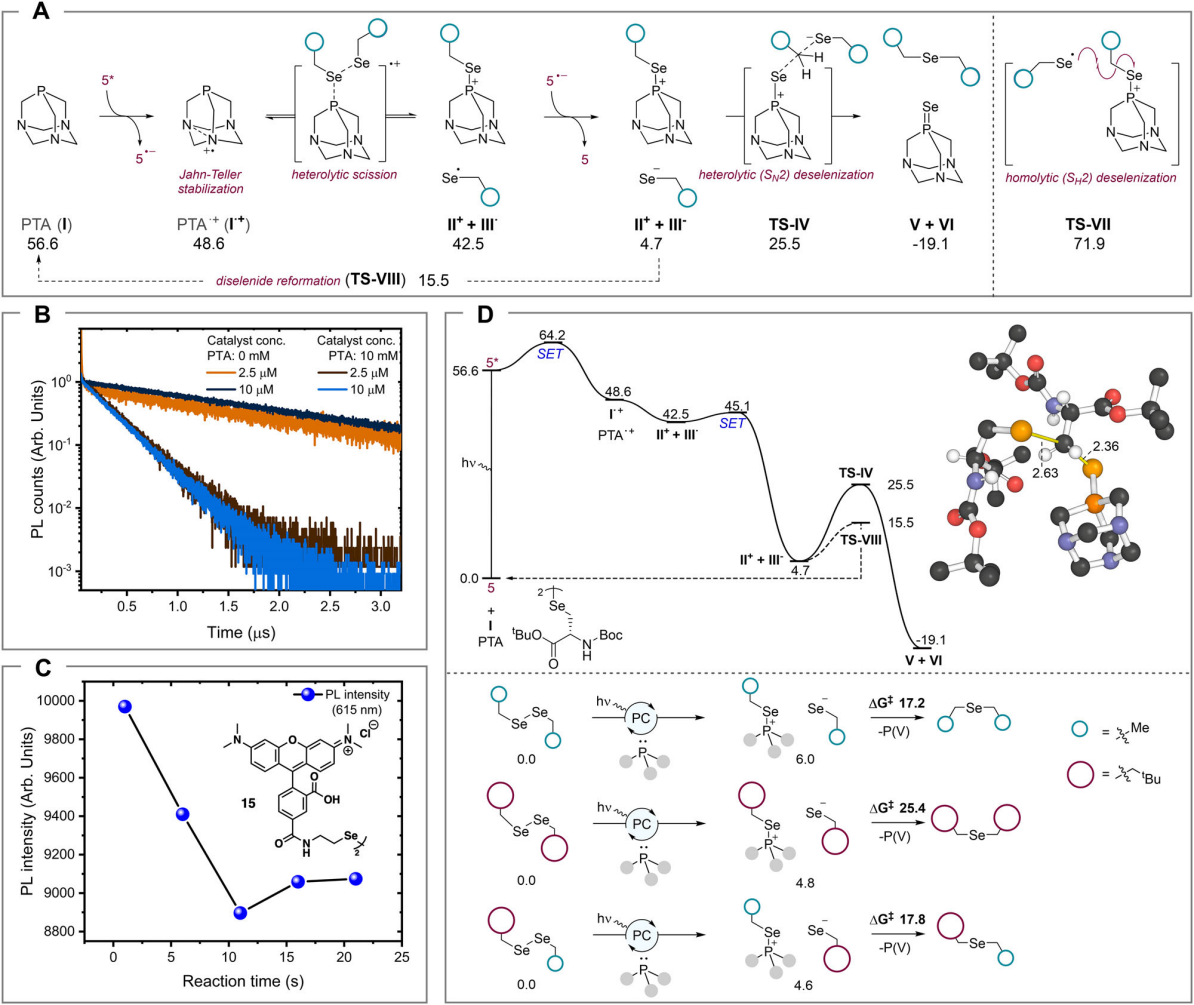
Mechanistic insights into the PDC reaction. **A** Proposed mechanism for the PDC reaction that converts diselenides to selenoethers. **B** Normalized photoluminescence (PL) counts of photocatalyst [Ir(dF(CF_3_)ppy)_2_(dtbpy)] (**5**) (*c* = 2.5 μM and 10 μM) in argon-sparged 1:1 v/v H_2_O:MeCN over a period of 3 μs monitored at 470 nm (excitation wavelength 415 nm) with or without PTA (**4**) (0 mM and 10 mM). **C** Changes in the PL intensity of 5-carboxytetramethylrhodamine (TAMRA)-derivatized selenocystamine (**15**) in the presence of [Ir(dF(CF_3_)ppy)_2_(dtbpy)] (**5**) and PTA (**4**) monitored at 615 nm (excitation wavelength 550 nm). **D** Top: B3PW91-GD3(BJ)/6-311 + G(2df,p)//B3PW91-GD3(BJ)/6-31 + G(d,p) computed Gibbs free energy profile in kcal/mol (SMD MeCN solvent, 298.15 K) including main and competitive pathways for [*N*-Boc-l-Sec-O*t*Bu]_2_; bottom: sensitivity of activation barriers for heterolytic deselenization to steric effects. PTA = 1,3,5-triaza-7-phosphaadamantane, Arb. Units arbitrary units, SET single electron transfer.

To interrogate the mechanism of the reaction, we employed photoluminescence (PL) spectroscopy, cyclic voltammetry (CV), and quantum chemical calculations. We probed the first steps of the mechanism through time-resolved PL spectroscopy. Specifically, time-correlated single-photon counting (TCSPC) experiments were conducted whereby photoexcitation of [Ir(dF(CF_3_)ppy)_2_(dtbpy)] (**5**) at 415 nm was performed in argon-sparged 1:1 v/v H_2_O:MeCN (as per the typical PDC reaction conditions). PTA (**4**) was added to a final concentration of 10 mM, with the photocatalyst concentration ranging from 2.5–250 μM. The PL kinetics of these solutions were monitored at different wavelengths spanning the entire PL spectrum of **5** from 430–630 nm. Plotting of the wavelength-dependent PL intensity for different delays of the laser pulse from the TCSPC data revealed that the emission spectra of the photocatalyst **5** showed no observable variation over the entire time window (0.01–0.30 µs) at different concentrations of **5**, consistent with it not partaking in direct covalent chemistry with the phosphine (Supplementary Figs. 42 and 43). Interestingly, the PL kinetics at each wavelength showed a clear dependence on the presence of PTA (**4**) (Fig. 2B). Specifically, upon addition of phosphine, the PL lifetime of the photocatalyst was diminished at least by a factor of two with a concomitant decrease in PL intensity. This suggests a non-radiative decay mechanism involving the PTA substrate leading to quenching of the photoexcited state of the photocatalyst. By comparing the PL kinetics of solutions with different molar equivalents of the photocatalyst and PTA, we inferred that an excess of PTA is needed for significant change in the PL dynamics of the photocatalyst, consistent with our experimental observations (Supplementary Figs. 44 and 45). This observed quenching of the photocatalyst by PTA is likely due to an electron transfer event between the photocatalyst and PTA, in agreement with previous reports^81^. Moreover, CV experiments confirmed that PTA (**4**) undergoes more facile single electron oxidation than the diselenide species in the reaction mixture, further supporting phosphine oxidation as the initial step of the reaction (Supplementary Figs. 46 and 47). In conjunction with our synthetic experimental observations, we proposed that the PDC reaction was initiated via a single-electron oxidation of the phosphine, PTA (**4**, **I**), mediated by the photoexcited photocatalyst, [Ir(dF(CF_3_)ppy)_2_(dtbpy)]^*^ (5*), to afford phosphine radical cation **I**^**•+**^ (Fig. 2A). It follows that this species could then intercept a diselenide substrate to yield the observed selanylphosphonium intermediate **II**^**+**^ and an equivalent of selanyl radical **III**^**•**^.

We also sought to probe the second step of our proposed mechanism using time-resolved PL spectroscopy. To this end, we synthesized selenocystamine derivatized with a 5-carboxytetramethylrhodamine (TAMRA) fluorophore (**15**). TAMRA was specifically chosen due to its red-shifted absorption spectrum relative to [Ir(dF(CF_3_)ppy)_2_(dtbpy)] (**5**), thus allowing for selective photoexcitation to monitor the PL response from the diselenide. The fluorescent TAMRA-labelled diselenide **15** was selectively excited with a 500 nm laser pulse (pulse width ~200 ps, repetition rate −80 MHz) in the presence of PTA (**4**) and/or [Ir(dF(CF_3_)ppy)_2_(dtbpy)] (**5**) and with continuous background LED irradiation at 450 nm to promote the PDC dimerization (Fig. 2C). The PL kinetics of **15** were monitored at different emission wavelengths over the course of continuous UV irradiation spanning 1–20 min. A subtle broadening and red shift of the steady-state emission spectra of **15** was observed throughout the course of the reaction as revealed by changes in the intensity and spectral shape of the emission profile (Fig. 2C, Supplementary Figs. 48–50). These results support the notion that, after initial oxidation, PTA (**4**) undergoes chemical reaction with the peptide diselenide, which in and of itself has no or minimal interaction with the photocatalyst, [Ir(dF(CF_3_)ppy)_2_(dtbpy)] (**5**), to generate the highly electrophilic selanylphosphonium **II**^**+**^. To close the photocatalytic cycle, we propose that an equivalent of nucleophilic selenolate anion **III**^**−**^ could be generated through single-electron reduction of selanyl radical **III**^**•**^, which could facilitate selenoether **V** formation through nucleophilic attack on the carbon center α to the Se-P bond of the activated selanylphosphonium **II**^**+**^ via a hypothetical transition state (**TS-IV**). This process would lead to the formation of a thermodynamically favourable π-bond between Se and P and the extrusion of phosphine selenide **VI**, that is observed by both mass spectrometry and ^31^P NMR spectroscopy following PDC reactions (Supplementary Fig. 51). Given that phosphine reagents can undergo S_N_2 attack on diselenides in the absence of light, that would also generate phosphine selenide **VI** (through an alternative deselenization pathway), we conducted a ^31^P NMR time study in which PTA (**4**) was incubated with [H_2_N-USPGYS-NH_2_]_2_ dimer (**1**) in D_2_O. This investigation confirmed that there is no background reaction to generate phosphine selenide **VI** over the timescale of the PDC reaction (up to 60 min); PTA oxide was the only species that could be observed (Supplementary Fig. 52).

Having established a working hypothesis for the mechanism of the PDC transformation and corroborated the feasibility of the first two proposed steps by time-resolved PL spectroscopy, we next sought to further probe the mechanistic hypothesis using quantum chemical calculations. We used density functional theory (DFT) calculations at the B3PW91-GD3(BJ)/6-311 + G(2df,p)//B3PW91-GD3(BJ)/6-31 + G(d,p) level of theory^82–85^, with barriers for electron-transfer estimated using protocols described previously by Houk and Buchwald^86^, Vaissier^87^ and a hybrid approach (used in Fig. 2D), obtaining similar conclusions (Supplementary Figs. 53–62, Supplementary Tables 5–8, Supplementary Data 1–2). An [*N*-Boc-l-Sec-O*t*Bu]_2_ amino acid diselenide system was studied to generate energy profiles, since its structure emulates the peptide-like environments employed experimentally, showing activation barriers accessible at room temperature (Fig. 2A, D).

The long-lived T_1_ triplet state of catalyst **5** formed following photoexcitation is 56.6 kcal/mol above its ground state. Single-electron oxidation of **PTA** (**I**) by 5* is computed to occur favourably (ΔG = −8.0 kcal/mol) with a small barrier, in line with the experimental PL spectroscopy data (Fig. 2B). Subsequently, the reaction of radical cation **I**^**•+**^ and diselenide is barrierless and exergonic by 6.1 kcal/mol, forming a selanyl phosphonium cation and selanyl radical (**II**^**+**^ and **III**^**•**^). We considered a *S*_*H*_*2* homolytic substitution pathway involving these species to form the Se-C bond (via the transition state **TS-VII**), however, the barrier is unfavorable (ΔG^‡^ = 29.4 kcal/mol). In contrast, back electron transfer from the catalyst to reduce the selanyl radical **III**^**•**^ to selenolate anion **III**^**−**^ is computed to be more facile (ΔG^‡^ = 2.6 kcal/mol) and highly exergonic. From the selanyl phosphonium/selenolate ion-pair, *S*_*N*_*2* nucleophilic substitution can occur at the α-carbon center to form the contracted product (through **TS-IV**) or at selenium center to reform the diselenide starting material (**TS-VIII**). Other pathways were considered, however they did not significantly affect the kinetics of the reaction. This reaction network was used to generate a microkinetic model for the conversion of a model substrate, dipentafluorobenzyl diselenide (see Supplementary Figs. 63–64)^88^. Comparison of simulated concentration profiles against experiment are consistent with the proposed mechanism and support the observed accumulation of phosphine selenide **VI** over the course of the reaction. We next interrogated the influence of sterics at the site of diselenide contraction to help rationalize the inability of the PDC conditions to dimerize the CaM (K148U) diselenide dimer protein **12** (Fig. 2D). Computationally, we found that the barrier for the heterolytic deselenization step (**TS-IV**) does not change significantly if there are relatively small substituents on the other side of the diselenide (ΔG^‡^ from 17.2 to 17.8 kcal/mol when a Me is replaced with a *t*Bu group). However, two sterically demanding substituents cause a substantial increase in barrier height (ΔG^‡^ of 25.4 kcal/mol); this potentially explains why CaM (K148U) **12** could not be dimerized under PDC conditions. Importantly however, these computational studies raised the exciting possibility that the PDC reaction could be applied to the rapid and site-selective functionalization of large selenoproteins using smaller diselenides.

### Site-specific functionalization of selenoproteins via PDC

Based on the computational studies above, we envisaged carrying out the kinetically driven formation of an asymmetric protein-small molecule diselenide by adding an excess of a small molecule diselenide to a protein diselenide. Contraction of this asymmetric diselenide under PDC conditions could then be carried out to execute rapid and site-specific protein functionalization at Sec. The feasibility of the late-stage functionalization strategy was first validated on model peptides, which pleasingly led to quantitative peptide functionalization within 5 min using slightly modified PDC conditions to those used for dimerization (see Supplementary Figs. 65–71). Importantly, the chemoselectivity of the transformation was also demonstrated on a model peptide containing a Sec and two Cys residues, with exclusive functionalisation observed at the Sec residue as demonstrated by mass spectrometry (see Supplementary Figs. 72 and 73).

**Fig. 3:**
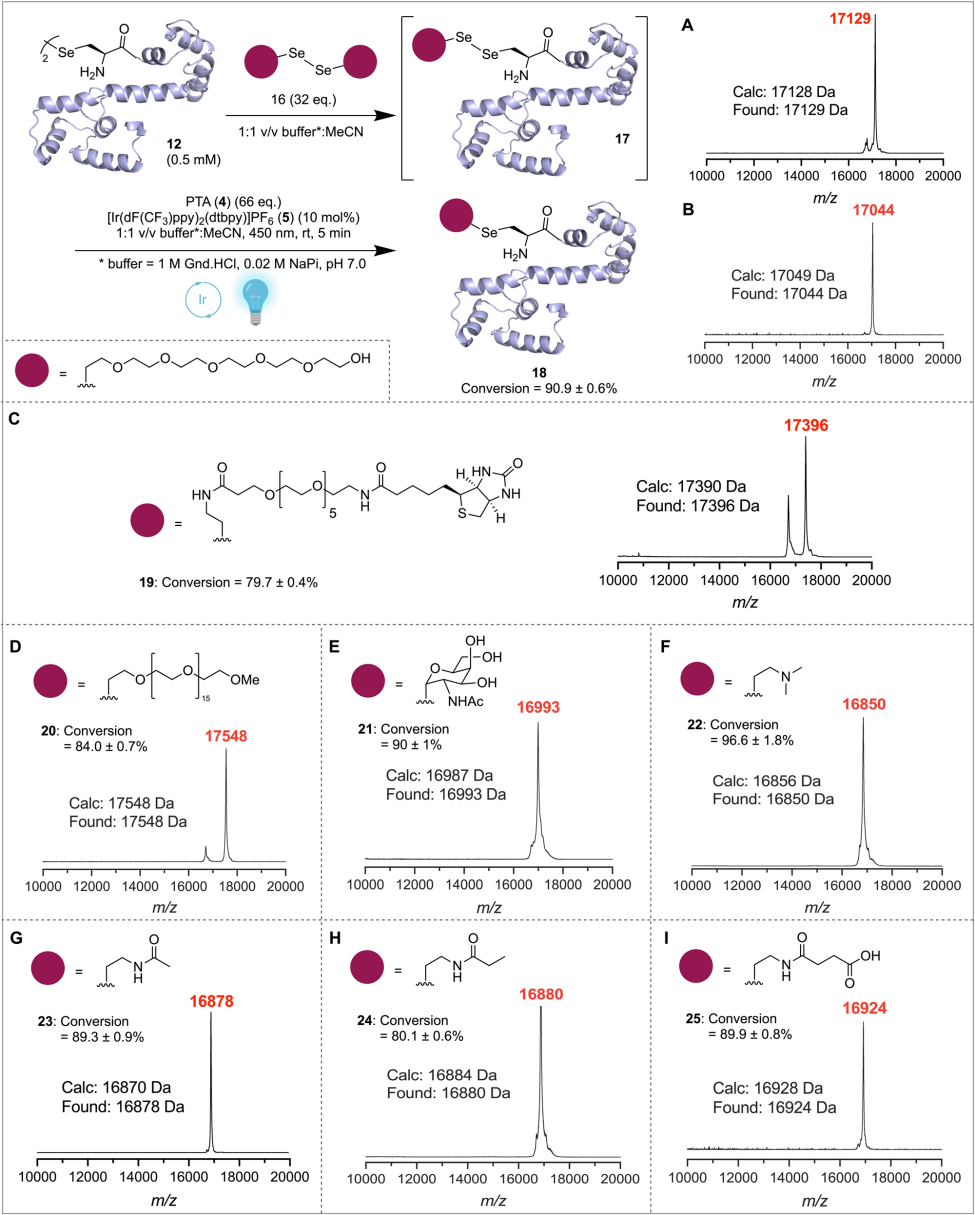
Late-stage modification of calmodulin (K148U) diselenide **12** via PDC. Functionalization of a CaM (K148U) diselenide **12** with [PEG_6_-Se]_2_ (**16**) to afford PEG_6_-modified CaM (K148U) **18**; mass spectra of the asymmetric diselenide **17** formed from mixing **12** and **16** (**A**) and subsequent photocatalytic contraction to afford **18** (**B**) are shown as insets. MALDI-TOF spectra of crude desalted reaction mixtures after PDC functionalisation with **C** biotin-PEG_5_- (**19**), **D** mPEG_17_- (**20**), **E** GalNAc- (**21**), **F** (Me)_2_NCH_2_CH_2_- (**22**), **G** AcHNCH_2_CH_2_- (**23**), **H** PropionylHNCH_2_CH_2_- (**24**), and **I** SuccHNCH_2_CH_2_- (**25**) are also inset. Reaction conversions were calculated through averaging integrations of HRMS-derived extracted ion chromatograms of the [M + 11H^+^]^11+^, [M + 10H^+^]^10+^ and [M + 9H^+^]^9+^ charge states and errors are reported as the standard deviation of the integration of these three ion peaks for a single experiment (see Supplementary Information). Ribbon structure used depicts the X-ray crystal structure solved for **18** (PDB: 7T2Q); PTA 1,3,5-triaza-7-phosphaadamantane, MeCN acetonitrile, Gnd.HCl guanidinium hydrochloride, NaP_i_ sodium phosphate. Created with BioRender.com.

Motivated by these results on peptidic systems, we turned our attention to the late-stage functionalization of the recombinant CaM (K148U) diselenide dimer protein **12**. To this end, **12** was first mixed with super-stoichiometric amounts of a synthetic hexaethylene glycol diselenide ([Se-PEG_6_]_2_; **16**). This led to exclusive formation of the CaM-PEG_6_ asymmetric diselenide **17** after mixing as judged by HPLC-MS analysis (Fig. 3). Addition of PTA (**4**) (66 equiv.) and 10 mol% [Ir(dF(CF_3_)ppy)_2_(dtbpy)]PF_6_ (**5**) and LED irradiation at 450 nm led to clean contraction to the desired PEG_6_-functionalized CaM (K148U) **18** in 5 min (91% conversion, Supplementary Figs. 74 and 75). The PEG_6_-CaM (K148U) selenoether conjugate **18** generated from a multi-milligram PDC reaction was submitted directly (without purification) to a crystallization screen (Supplementary Fig. 76). A crystal diffracted to 1.95 Å resolution by X-ray crystallography (Supplementary Fig. 77 and Supplementary Table 9), with the refined structure of **18** nearly identical to ligand-bound conformations of CaM published in the protein databank, thus confirming the retention of higher order secondary and tertiary structural features. Importantly, this highlights the biocompatibility of the PDC conditions (see Fig. 3 for solved protein structure). While electron density surrounding the PEG_6_ selenoether modification could not to be resolved, the presence of a Se atom in this structure was confirmed through X-ray absorption at the Se edge (Kα; E = ~1152 deV) (Supplementary Fig. 78).

Having established conditions for the rapid functionalization of CaM (K148U) diselenide (**12**) with PEG_6_, we next explored the scope of the PDC functionalization chemistry using a diverse library of PTM mimics, PEG oligomers and purification handles (see Supplementary Figs. 79–103 for small molecule diselenide characterization). Gratifyingly, reaction with larger diselenides ([Biotin-PEG_5_-Se]_2_ and [mPEG_17_-Se]_2_) under the optimized PDC conditions led to clean site-specific functionalization of CaM (K148U) (**12**) within 5 min to afford conjugates **19** and **20** (Fig. 3, Supplementary Figs. 104–107). CaM (K148U) (**12**) could also be rapidly and efficiently modified with PTMs including *N*-acetylgalactosamine (α-GalNAc) (**21**), and mimics of Lys dimethylation (**22**), acetylation (**23**), propionylation (**24**) and succinylation (**25**) (Fig. 3, Supplementary Figs. 108–117). Pleasingly, in all the cases, functionalization of CaM (K148U) (**12**) was accomplished within 5 min following treatment with the corresponding diselenides (see Supplementary Information for synthetic details), PTA (**4**) and irradiation at 450 nm, with excellent conversions (80–97%). Taken together, these examples showcase the utility of the PDC methodology for the simple, fast and efficient installation of PTM mimics and designer modifications such as PEGylation and affinity handles to proteins in a chemo- and regioselective manner.

**Fig. 4:**
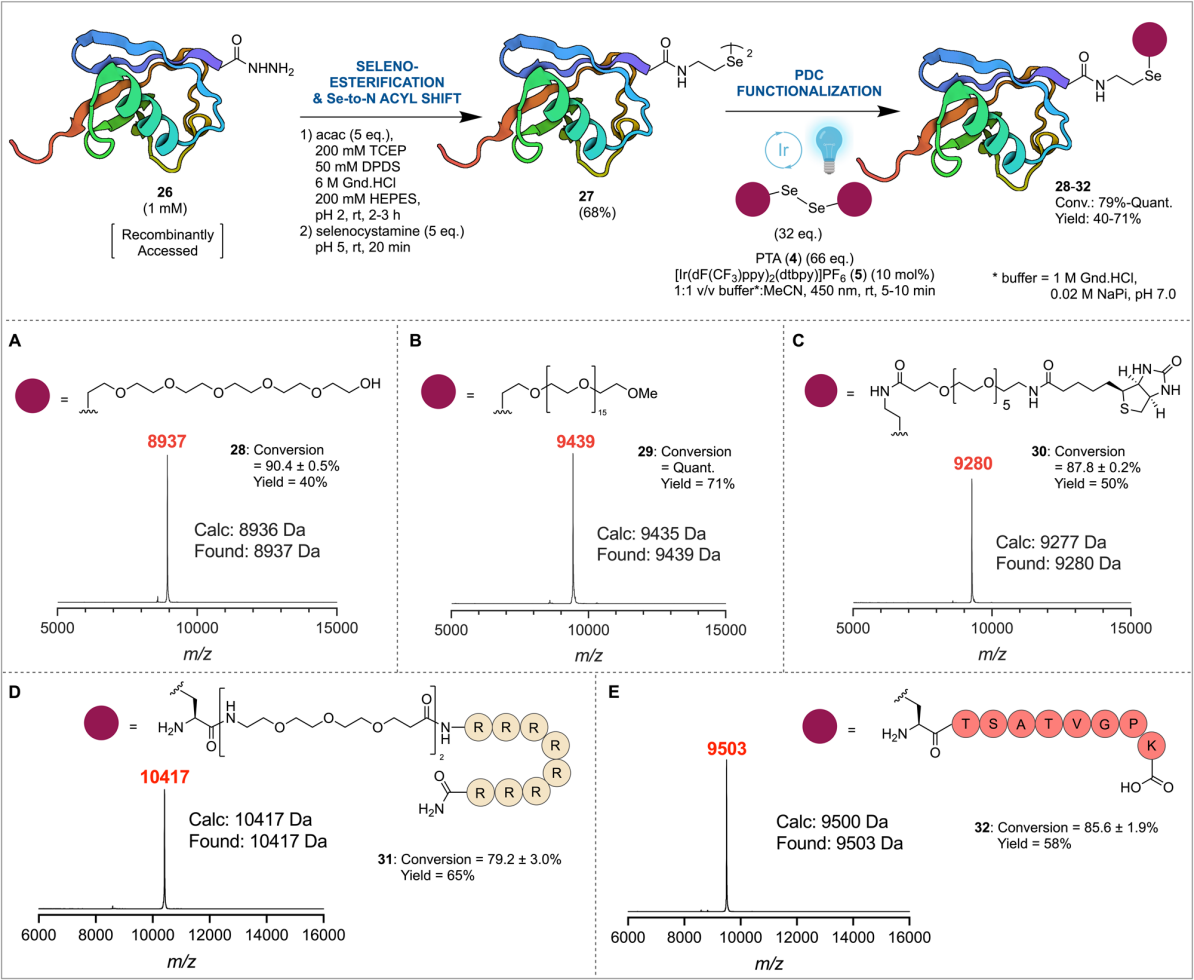
Site-specific C-terminal functionalization of ubiquitin diselenide **27** via PDC. Conversion of ubiquitin acyl hydrazide **26** to ubiquitin diselenide **27** and MALDI-TOF spectra of crude desalted reaction mixtures after C-terminal functionalization with **A** PEG_6_- (**28**), **B** mPEG_17_- (**29**), **C** Biotin-PEG_5_- (**30**), **D** Arg_8_-(PEG_4_)_2_-Ala- (**31**) and **E** H2AX- (**32**) under optimized PDC conditions. Reaction conversions were calculated through averaging integrations of HRMS-derived extracted ion chromatograms of the [M + 9H^+^]^9+^, [M + 8H^+^]^8+^ and [M + 7H^+^]^7+^ charge states and errors are reported as the standard deviation of the integration of these three ion peaks for a single experiment (see Supplementary Information); acac acetylacetone, TCEP *tris*-carboxyethylphosphine, DPDS diphenyl diselenide, Gnd.HCl guanidinium hydrochloride, HEPES = 4-(2-hydroxyethyl)−1-piperazineethanesulfonic acid, PTA 1,3,5-triaza-7-phosphaadamantane, MeCN acetonitrile, NaP sodium phosphate, Quant. quantitative conversion, Yield isolated yield of modified protein following reverse-phase HPLC purification. Created with BioRender.com.

Having demonstrated the rapid and efficient functionalization of an expressed protein bearing the 21st amino acid, Sec, we next sought to assess the utility of the PDC technology for the C-terminal functionalization of an expressed protein, namely human ubiquitin. To this end, a gene encoding the full-length ubiquitin sequence was fused to the *Mycobacterium xenopi* DNA Gyrase A intein and a chitin-binding domain. Following expression in *E. coli*, the fusion protein was immobilized on chitin beads and the target ubiquitin acyl hydrazide **26** liberated by treatment with aqueous hydrazine (Fig. 4, Supplementary Figs. 118 and 119). Acetylacetone (acac)-based activation and treatment with selenocystamine then provided ubiquitin diselenide dimer **27** (via initial selenoester formation and a rapid *Se*-to-*N* acyl shift^89^, Supplementary Fig. 120). In addition, a Cys mutant of ubiquitin (K48C) was generated bearing a C-terminal selenocystamine moiety (using the same intein-fusion approach used for the wild-type protein) to assess the chemoselectivity of PDC functionalization in the presence of Cys (Supplementary Fig. 121). To explore PDC-functionalization at the C-terminus of ubiquitin diselenide **27**, we selected a range of reagents, including [PEG_6_-Se]_2_, [mPEG_17_-Se]_2_ and [Biotin-PEG_5_-Se]_2_. Pleasingly, reaction under the optimized photocatalytic conditions afforded the C-terminally functionalized ubiquitin bearing PEG_6_ (**28**), mPEG_17_ (**29**), and PEG_5_-biotin (**30**) with 88% to quantitative conversions in 5–10 min and with very high crude purities (Fig. 4, Supplementary Figs. 122–127). Purification by reverse-phase HPLC then provided each of the conjugates in 40–71% isolated yield. Pleasingly, the Cys-containing mutant, ubiquitin (K48C), also underwent chemo- and regioselective functionalization at the C-terminal diselenide moiety with [PEG_6_-Se]_2_ diselenide (**16**) under modified PDC conditions, whereby the amount of phosphine [PTA (**4**)] was reduced to 18 molar equivalents to prevent deleterious desulfurization of the Cys residue (Supplementary Fig. 128). Finally, to explore the conjugation of larger structures to the C-terminus of **27**, we synthesized a Sec-(PEG_4_)_2_-Arg_8_ diselenide dimer peptide as an example of a cell penetrating peptide motif^90^ (see Supplementary Fig. 129 for synthesis). Pleasingly, treatment of ubiquitin diselenide **27** with this larger peptidic diselenide under optimized PDC conditions led to clean conversion to the selenoether-linked conjugate **31** within 5 min and was isolated in good yield following purification by reverse-phase HPLC (Fig. 4, Supplementary Figs. 130 and 131). Finally, a selenopeptide derived from the histone protein H2AX^91^ (see Supplementary Fig. 132 for synthesis) was also fused to the C-terminus of ubiquitin via PDC in excellent yield to afford selenoether-linked conjugate **32**, which represents a stable structural mimic of ubiquitin linked to a Lys side chain within a polypeptide (Fig. 4, Supplementary Figs. 133 and 134). Taken together, these examples of asymmetric protein-peptide coupling, a transformation that would be difficult to achieve using existing bioconjugation methods, lay the foundation for expanding the scope of the PDC manifold toward a wide range of other high value peptide–protein conjugates in the future, for example antibody-drug conjugates bearing cytotoxic payloads.

In summary, we have discovered a synthetic transformation called the photocatalytic diselenide contraction (PDC) that enables the rapid and efficient conversion of diselenides to stable selenoethers with the formal extrusion of a selenium atom. The mild PDC conditions (comprising an iridium photocatalyst, a phosphine and blue LED irradiation) enabled fast and clean conversion of several Sec-containing diselenide dimer peptides to selenoether dimers within 5 min, however, larger protein diselenide dimers could not be similarly dimerized. Detailed interrogation of the proposed reaction pathway by PL spectroscopy and DFT calculations was used to substantiate the proposed mechanism of this reaction, and provided key insight into a PDC reaction pathway for the site-selective functionalization of proteins through the contraction of asymmetric protein-small molecule diselenides. The feasibility and utility of this late-stage functionalization chemistry was demonstrated through the site-specific modification of selenocalmodulin and the C-terminal modification of ubiquitin with a diverse array of small molecule diselenides, including examples with larger polypeptide chains. By virtue of the chemoselectivity and regioselectivity of the PDC transformation at diselenides (including at the side chain of the very rare 21st proteinogenic amino acid Sec) we envisage that the PDC methodology will find widespread utility in the generation of modified proteins and therapeutically valuable protein conjugates in the future.

## Methods

### Ethics

All procedures involving the collection of blood from healthy donors were approved by the University of Sydney Human Research Ethics Committee (HREC, Project 2014/244) and all studies conformed to the principles outlined in the Declaration of Helsinki. Written informed consent was obtained from all human research participants.

### General PDC conditions for peptide dimerization

Peptide diselenide (1 eq.) was dissolved in a solution of [Ir(dF(CF_3_)ppy)_2_(dtbpy)]PF_6_ (1 mol%). in either 1:1 v/v MeCN:H_2_O or 1:1 v/v MeCN:1 M Gnd.HCl, 0.02 M NaP_i_, pH 7.0 buffer, to a final peptide concentration of 2.5–10 mM. This solution was then used to dissolve PTA (4 eq.), and the reaction mixture irradiated with 450 nm LED light for 4–6 min. Aliquots were taken at 0 min and 5 min following irradiation for analysis of reaction progression by UPLC-MS and UPLC. The crude reaction mixture was then purified by reverse-phase HPLC to afford selenoether-linked peptide dimers following lyophilization.

### General PDC conditions for protein functionalization

Protein diselenide (1 eq.), small molecule diselenide (32 eq.), PTA (66 eq.), and [Ir(dF(CF_3_)ppy)_2_(dtbpy)]PF_6_ (10 mol% relative to protein diselenide) were dissolved in 1:1 v/v MeCN:1 M Gnd.HCl, 0.02 M NaP_i_, pH 7.0 buffer, to give a final protein concentration of 0.5 mM. The reaction mixture was then irradiated with 450 nm LED light for 5 min. Aliquots were taken at 0 min and 5 min for analysis of reaction progression by MALDI-TOF MS and LC-HRMS. Isolated yields of functionalized ubiquitin proteins were obtained after crude reaction mixtures were purified by reverse-phase HPLC and lyophilized.

### Reporting summary

Further information on research design is available in the Nature Portfolio Reporting Summary linked to this article.

## Supplementary information

Supplementary Information

Description of Additional Supplementary Files

Supplementary Dataset 1

Supplementary Dataset 2

Reporting Summary

## Data Availability

Thermochemical data (Supplementary Data 1) and molecular coordinates from computational mechanistic studies (Supplementary Data 2), and the PDB validation report for 7T2Q are provided with this manuscript as Supplementary Data Sets. The thermochemical data (obtained with the GoodVibes software, https://github.com/bobbypaton/GoodVibes) and molecular coordinates from computational mechanistic studies are also available in Zenodo (10.5281/zenodo.7224862). The mass spectrometry proteomics data have been deposited to the ProteomeXchange Consortium via the PRIDE partner repository with the dataset identifier PXD037525. Data is available from the corresponding authors upon request.

## Code availability

The AQME program (https://github.com/jvalegre/aqme) was used to carry out the conformational sampling in computational studies. The CSV input file and Jupyter Notebook workflow used are available in Zenodo: 10.5281/zenodo.7224862.
