# Supplementary material for: Site-selective photocatalytic functionalization of peptides and proteins at selenocysteine
Source: Nat Commun. 2022 Nov 12;13:6885. doi: 10.1038/s41467-022-34530-z (PMC9653470; doi:10.1038/s41467-022-34530-z)
Supplement: Supplementary file 1 — Supplementary Information [file 41467_2022_34530_MOESM1_ESM.pdf]

Supplementary Information

Site-Selective Photocatalytic Functionalization of Peptides and Proteins at Selenocysteine

Luke J. Dowman[1,2], Sameer S. Kulkarni[1,2], Juan V. Alegre-Requena[3], Andrew M. Giltrap[1,2], Alexander R. Norman[1,2], Ashish Sharma[1,4] Liliana C. Gallegos[3], Angus S. Mackay[1,2], Adarshi P. Welegedara[5], Emma E. Watson[1,2], Damian van Raad[5], Gerhard Niederacher[6], Susanne Huhmann[6], Nicholas Proschogo[1], Karishma Patel[7], Mark Larance[8], Christian F. W. Becker[6], Joel P. Mackay[7], Girish Lakhwani[1,4], Thomas Huber[5], Robert S. Paton[3], Richard J. Payne[1,2*]

Affiliations:

[1]School of Chemistry, The University of Sydney, Sydney, NSW 2006, Australia

[2]Australian Research Council Centre of Excellence for Innovations in Peptide and Protein Science, The University of Sydney, NSW 2006, Australia

[3]Department of Chemistry, Colorado State University, Fort Collins, CO 80523-1872, United States of America

[4]Australian Research Council Centre of Excellence in Exciton Science, The University of Sydney, NSW 2006, Australia

[5]Research School of Chemistry, Australian National University, Canberra, ACT 2601, Australia

[6]Institute of Biological Chemistry, Faculty of Chemistry, University of Vienna, Vienna, Austria

[7]School of Life and Environmental Sciences, The University of Sydney, Sydney, NSW 2006, Australia

[8]Charles Perkins Centre and School of Medical Sciences, The University of Sydney, Sydney, NSW 2006, Australia

*Correspondence to: richard.payne@sydney.edu.au

Table of Contents

Supplementary Methods……………………………………………..……………….4

General Procedures 4

General Materials and Methods 4
Fmoc Solid-Phase Peptide Synthesis 5
Manual Coupling of Fmoc-Sec(Bn)-OH and [Boc-Sec-OH]$_2$ 6
Preparative High-Performance Liquid Chromatography (HPLC) 6
Ultra-Performance Liquid Chromatography-Mass Spectrometry (UPLC-MS) 6
Analytical Ultra-Performance Liquid Chromatography (UPLC) 7
Nuclear Magnetic Resonance (NMR) Spectroscopy 7
Mass Spectrometry (MS) 7
Culture Media 8
Agarose Gel Electrophoresis (AGE) 9
Sodium Dodecyl Sulfate-Polyacrylamide Gel Electrophoresis (SDS-PAGE) 9
Plasmid DNA Amplification and Purification 10
DNA Gel Purification 10
Preparative Fast Protein Liquid Chromatography (FPLC) 10
Photocatalytic Diselenide Contraction (PDC) Reactions 11
Synthesis of Selenocysteine Variants 11
Synthesis of Model Selenopeptide [H$_2$N-USPGYS-NH$_2$]$_2$……………………………………………..12

Initial Photocatalytic Deselenization Studies 12

Model System Optimization Studies 15

Photocatalyst Screen 15
Phosphine Screen 19
Phosphine Equivalents Screen 23
Solvent Screen 26
Concentration Screen 29
'No Phosphine' Control PDC Dimerization Reaction 33
'No Photocatalyst' Control PDC Dimerization Reaction 34
'No Light' Control PDC Dimerization Reaction 35

Mass Spectrometric and NMR Analysis of Model Selenoether Dimers 36

High-Resolution Mass Spectrometric Analysis of Selenoether Dimer **3** 36

Synthesis of [Boc-Sec-Phe-OMe]$_2$ diselenide 39

Synthesis of a Boc-Sec-Phe-OMe Selenoether by PDC Dimerization 40
NMR Characterization of Boc-Sec-Phe-OMe Selenoether Dimer 40

Stability Studies on Model Selenoether Dimer 43

Stability Studies on Selenoether Dimer **3** under Acidic, Basic, and Reducing Conditions 43
Stability Studies on Selenoether Dimer **3** in Human Plasma 46

Synthesis of Diselenide Dimer Peptides 9-11……………………………………………………….47

PDC Dimerization of Peptide Targets 53

Bacterial Expression of CaM (K148U) Diselenide 12 59

Attempted PDC Dimerization of CaM (K148U) Diselenide 12 63

Photoluminescence (PL) Spectroscopy and Cyclic Voltammetry 65

^{31}P NMR Spectroscopy Studies on [H$_2$N-USPGYS-NH$_2$]$_2$ 71

Computational Mechanistic Studies 74

Computational Methods 74
Thermochemical Data Calculation with *GoodVibes* 75
Example Input Files 76
Complete PES and Representations of the Most Stable Conformers 77
SET Calculations 81
Berkeley Madonna Kinetic Simulations Using $(C_6F_5\text{-}CH_2\text{-}Se)_2$ 85
Molecular Coordinates 90

Optimization of PDC Functionalization on a Model Selenopeptide System 91

Synthesis of Model Selenopeptide MUC1 (U11) 91
Small Molecule Equivalents Screen: PTA (**4**) Scaling = 4 Equiv. 92
Small Molecule Equivalents Screen: PTA (**4**) Scaling = 2 Equiv. 94
Solvent Screen 96
Concentration Screen 97
Control Reactions 99

Optimized PDC Functionalization of $[MUC1 (U11)]_2$ Diselenide 101

Chemoselective functionalization of model peptide H_2N-USPCYSC-NH_2 with PEG_6 diselenide (at Sec) 102

Late-Stage PDC Functionalization of CaM (K148U) Diselenide 12 102

Crystallization Trials of PEG_6-CaM (K148U) Selenoether **18** 106
X-Ray Crystallography of PEG_6-CaM (K148U) Selenoether **18** 107

NMR Spectra 110

Bacterial Expression of Ubiquitin (Ub, Ub (K48C)) Acyl Hydrazides 150

One-pot C-terminal Selenoesterification of Ubiquitin (26) and Selenocystamine Fusion 152

One-pot C-terminal Selenoesterification of Ubiquitin (K48C) Selenoesterification and Selenocystamine Fusion 154

Late-Stage C-Terminal PDC Functionalization of Ubiquitin Diselenide 27 156

Synthesis of Small Molecule Diselenides 175

Plasmid DNA Sequence of pUC-PT7-His_6-MBP-TEV-CaM (K148U) 190

Plasmid Sequence for pTXB1-Ub-Mxe-His-CBD 191

Primer Oligonucleotide DNA Sequences 194

Calmodulin Protein Sequences 195

Ubiquitin Protein Sequences 196

Supplementary References 197

Supplementary Methods

General Procedures

General Materials and Methods

Peptide grade *N*,*N*-dimethylformamide (DMF) and CH_2Cl_2 for peptide synthesis were purchased from RCI Labscan (Bangkok, Thailand) and Merck (Darmstadt, Germany), respectively. Gradient grade MeCN for chromatography was purchased from Sigma-Aldrich (MO, USA) and ultrapure H_2O (Type 1) was obtained from a Direct-Q 5 Water Purification System (Millipore, MA, USA). Standard Fmoc-protected amino acids, coupling reagents and resins were purchased from Mimotopes (VIC, Australia) and Novabiochem (Darmstadt, Germany). All non-commercially available amino acids were synthesised according to literature procedures as described below. Fmoc-SPPS was performed manually with these reagents and solvents in polypropylene Teflon-fritted syringes purchased from Torviq or through automated synthesis on a Syro I peptide synthesizer (Biotage, Uppsala, Sweden). Photocatalysts were purchased from Strem Chemicals (MA, USA) or Sigma-Aldrich (MO, USA) and all other reagents were purchased from Sigma-Aldrich (MO, USA) or AK Scientific (CA, USA).

All reactions were carried out under an Ar atmosphere and at room temperature (25 ^oC) unless the reaction was performed under aqueous conditions or unless otherwise specified. Reactions undertaken at -78 ^oC utilized a bath of dry ice and acetone. Reactions carried out at 0 ^oC employed a bath of H_2O and ice. Anhydrous THF, CH_2Cl_2 and MeOH were obtained using a PureSolv solvent purification system with water detectable only in low ppm levels. Reactions were monitored by thin layer chromatography (TLC) on aluminium backed silica plates (Merck Silica Gel 60 F254). Visualization of TLC plates was undertaken with an ultraviolet (UV) light at λ = 254 nm and staining with solutions of vanillin, ninhydrin, phosphomolybdic acid (PMA), potassium permanganate or sulfuric acid, followed by exposure of the stained plates to heat. Silica flash column chromatography (Merck Silica Gel 60 40 – 63 μm) was undertaken to purify crude reaction mixtures using solvents as specified.

Fmoc Solid-Phase Peptide Synthesis

Resin Loading

2-Chlorotrityl Chloride Resin (2-CTC): 2-CTC resin (1.14 mmol g^{-1} loading) was swollen in CH_2Cl_2 for 10 min and then washed with CH_2Cl_2 (4 × 2 mL). The resin was treated with a solution of thionyl chloride in CH_2Cl_2 (2 vol%) for 30 minutes and then thoroughly washed with CH_2Cl_2 (10 × 2 mL). The washed resin was then treated with a solution of Fmoc-protected amino acid (2 equiv.) and iPr_2NEt (8 equiv.) in CH_2Cl_2 (c = 0.1 M) for 16 h at room temperature. The resin was then filtered and washed with CH_2Cl_2 (4 × 2 mL) before being treated with a solution of methanol and iPr_2NEt in CH_2Cl_2 (17:2:1 v/v/v CH_2Cl_2:MeOH:iPr_2NEt) for 30 mins at room temperature. The resin was finally filtered and washed with CH_2Cl_2 (4 × 2 mL) and DMF (4 × 2 mL) prior to iterative peptide assembly.

Rink Amide Resin: Rink amide resin (0.3–0.49 mmol g^{-1} loading) was swollen in CH_2Cl_2 (2 mL) for 30 min and then washed with CH_2Cl_2 (4 × 2 mL) and DMF (4 × 2 mL). the resin was then Fmoc-deprotected by treatment with a solution of 20 vol% piperidine in DMF (2 mL, 2 × 3 min) then washed with DMF (4 × 2 mL). The resin was then treated with a solution of Fmoc-protected amino acid (5 equiv.), DIC (5 equiv.) and Oxyma (5 equiv.) in DMF ([Fmoc-Xaa-OH] = 0.3 M) at 50 ºC for 30 min. The resin was then washed with DMF (4 × 2 mL) and treated with a solution of Ac_2O (2.5 vol%) and iPr_2NEt (5 vol%) in DMF for 5 min at room temperature then washed with DMF (4 × 2 mL).

Automated Peptide Synthesis (Biotage Syro I Peptide Synthesizer)

Deprotection: The resin (50 µmol) was treated with a solution of 40 vol% piperidine in DMF (800 µL) for 4 min, drained then treated with 20 vol% piperidine in DMF (800 µL) for 4 min, drained and then washed with DMF (4 × 1.25 mL).

Coupling (standard): The resin was treated with a solution of Fmoc-protected amino acid (200 µmol, 4 eq.) and Oxyma (220 µmol, 4.4 eq.) in DMF (400 µL), DIC (200 µmol, 4 eq.) in DMF (400 µL) and a solution of 1,3-diisopropyl-2-thiourea in DMF (1 wt.%, 400 µL). All coupling reactions were conducted at 40 ºC for 45 mins. The resin was then drained and washed with DMF (4 × 1.25 mL).

Capping: The resin was treated with a solution of acetic anhydride (5 vol%) and DIPEA (10 vol%) in DMF (800 µL) for 6 min at room temperature. The resin was drained and then washed with DMF (4 × 1.25 mL).

Manual Coupling of Fmoc-Sec(Bn)-OH and [Boc-Sec-OH]$_2$

The resin was treated with a solution of Fmoc-Sec(Bn)-OH (1.5 eq.) or [Boc-Sec-OH]$_2$ (0.6 eq.), DIC (1.5 eq.) and HOAt (3 eq.) in DMF ([Fmoc-Xaa-OH] = 0.1 M) for 16 h at room temperature. The resin was then filtered and washed with DMF (4 × 4 mL).

Preparative High-Performance Liquid Chromatography (HPLC)

Preparative and semi-preparative reversed-phase high performance liquid chromatography (HPLC) was performed using a Waters 600E multisolvent delivery system with a Rheodyne 7725i injection valve (5 mL loading loop) with a Waters 500 pump and a Waters 490E programmable wavelength detector operating at 214 nm and 254 nm. Preparative reversed-phase HPLC was performed using a Waters Sunfire C18 column (5 µm, 19 × 150 mm) at a flow rate of 14 mL min^{-1}. Semi-preparative reversed-phase HPLC was performed using a Waters Sunfire C18 column (5 µm, 10 × 250 mm) at a flow rate of 4 mL min^{-1}. All preparative and semi-preparative HPLC used a mobile phase of ultrapure (type 1) water (Solvent A) and acetonitrile (Solvent B) with 0.1 vol% trifluoroacetic acid (TFA).

Ultra-Performance Liquid Chromatography-Mass Spectrometry (UPLC-MS)

Ultra-Performance Liquid Chromatography-Mass Spectrometry (UPLC-MS) was performed on a Shimadzu 2020 UPLC-MS instrument with a Nexera X2 LC-30AD pump, Nexera X2 SPD-M30A UV/Vis diode array detector and a Shimadzu 2020 (ESI) mass spectrometer operating in positive mode. Separations were performed on a Waters Acquity BEH300 1.7 µm, 2.1 × 50 mm (C18) column at a flow rate of 0.6 mL min^{-1}. All separations were performed

using a mobile phase of 0.1 vol% formic acid in water (Solvent A) and 0.1 vol% formic acid in MeCN (Solvent B) using linear gradients as specified.

Analytical Ultra-Performance Liquid Chromatography (UPLC)

Analytical UPLC was performed on a Waters Acquity UPLC system equipped with a PDA eλdetector (λ= 210–400 nm), a sample manager FAN and Quaternary Solvent Manager (H-Class) modules. Separations were performed on a Waters Acquity BEH300 1.7 μm, 2.1 × 50 mm (C18) column at a flow rate of 0.6 mL min^{-1}. All separations were performed using a mobile phase of 0.1 vol% TFA in water (Solvent A) and 0.1 vol% TFA in MeCN (Solvent B) using linear gradients as specified.

Nuclear Magnetic Resonance (NMR) Spectroscopy

^{1}H NMR spectra were obtained using a Bruker DRX 400 or DRX 500 at frequencies of 400 MHz or 500 MHz respectively in $CDCl_3$, MeOD-d_4 or DMSO-d_6. Chemical shifts are reported in parts per million (ppm) and coupling constants in Hertz (Hz). The residual solvent peaks were used as internal standards without the use of tetramethylsilane (TMS). ^{1}H NMR data is reported as follows: chemical shift values (ppm), relative integral, multiplicity (s = singlet, brs = broad singlet, d = doublet, t = triplet, q = quartet, m = multiplet), coupling constant(s) and assigned peak. ^{13}C NMR spectra were obtained using a Bruker DRX 400 or DRX 500 at 101 MHz or 126 MHz in $CDCl_3$, MeOD-d_4, or DMSO-d_6 unless otherwise specified. ^{13}C NMR data is reported as chemical shift values (ppm). ^{77}Se NMR spectra were obtained on a Bruker DRX400 or DRX 500 at frequencies of 76 MHz or 95 MHz, respectively.

Mass Spectrometry (MS)

Small Molecule and Peptide MS:

Low resolution mass spectra for novel compounds were recorded on a Bruker amaZon SL mass spectrometer (ESI) operating in positive mode or on a Shimadzu 2020 (ESI) mass spectrometer operating in positive mode. High resolution mass spectra were recorded on a Bruker-Daltronics Apex Ultra 7.0 T Fourier transform (FTICR) mass spectrometer.

Protein MS:

Protein samples (purified and crude PDC reaction mixtures) were analyzed by Matrix-Assisted Laser Desorption/Ionisation Time-of-Flight (MALDI-TOF) and LC-HRMS.

MALDI-TOF mass spectra were measured on a Bruker Ultraflex MALDI-TOF instrument operating in linear mode and using a matrix of 2,5-dihydroxyacetophenone (DHAP) and diammonium hydrogen citrate in 1:1 v/v MeCN:H_2O supplemented with 1 vol% TFA. LC-HRMS was performed on a Thermo Fusion, Fusion Lumos or QExactive HFX mass spectrometer connected to a Thermo Dionex Ultimate 3000 LC system. Sample injections (10 μL) were separated on a 4.6 × 150 mm Halo C4 column (Advanced materials Technology, DE, USA) at a flow rate of 1 mL min^{-1} and over a gradient of 5-98% B (A: 0.1 vol% FA in H_2O; B: 0.1 vol% FA in 4:1 v/v MeCN:H_2O) over 15 min. ESI settings were as follows:

Source:	HESI
Spray Voltage:	3.7-4.0 kV
Sheath Gas:	10 L min^{-1}
Aux Gas:	3 L min^{-1}
Sweep Gas:	5 L min^{-1}
Aux Gas Temperature:	80 ^{o}C
MS Scan Width:	500-2000 *m/z* (profile mode)
Resolution:	120,000
Max Injection Time:	246 ms
AGC target:	$3x10^6$ (QExactive) or $4x10^5$ (Fusion)

Culture Media

All media reagents were used as supplied by Amyl Media (VIC, Australia) and Chem-Supply (SA, Australia).

Luria-Bertani (LB) Medium

LB media consisted of yeast extract (0.5% w/v), tryptone (1% w/v) and NaCl (1% w/v) dissolved in milliQ H_2O. All media were sterilized by autoclave at 121 ^{o}C before use and supplemented with growth factors and/or antibiotics as specified.

Terrific Broth (TB) Medium

TB media consisted of yeast extract (2.4% w/v), tryptone (2% w/v) and glycerol (0.4% w/v) dissolved in milliQ H_2O. All TB media were sterilised by autoclave at 121 ^oC before addition of phosphate buffer *via* sterile filtration (PES membrane, 0.22 μm) to final concentrations of: KH_2PO_4 (0.017 M) and K_2HPO_4 (0.072 M). Media were used sterile and supplemented with growth factors and/or antibiotics as specified.

Super Optimal Broth - Catabolite Repression (SOC) Medium

SOC medium was used sterile and as supplied by New England Biolabs (MA, USA).

LB-Agar Plates

LB media consisted of yeast extract (0.5% w/v), tryptone (1% w/v), NaCl (1% w/v) and agar (1.5% w/v) dissolved in milliQ H_2O. All media were sterilized by autoclave at 121 ^oC and then allowed to cool to ~50 ^oC before supplementation with growth factors and/or antibiotics as specified and pouring into Eppendorf non-treated cell culture dishes.

Agarose Gel Electrophoresis (AGE)

AGE was performed at using gels pre-cast at agarose concentrations of either 0.8%, 1% or 1.2% (w/v) in 50 mL of 1X TAE buffer containing SYBR Safe (Thermo Fisher Scientific, MA, USA). After setting of the gel, AGE was performed in 1X TAE buffer at 100 V for 40 min (1% agarose) or 100 V for 30 min (0.8% agarose). Samples (up to 50 μL) were prepared by addition of 10X TriTrack loading dye (Thermo Fisher Scientific, MA, USA) and loaded into wells alongside the 1 kb Plus ladder (5 μL, New England Biolabs, MA, USA). After completion of gel runs, gels were visualised directly under UV irradiation using a Bio-Rad (CA, USA) Gel Doc XR+ UV transilluminator.

Sodium Dodecyl Sulfate-Polyacrylamide Gel Electrophoresis (SDS-PAGE)

SDS-PAGE electrophoresis was carried out using pre-cast Thermo Fisher Scientific (MA, USA) Bolt Bis-Tris Plus 4-12% gradient polyacrylamide gels and 1X NuPAGE MES running buffer. Sample aliquots (25 μL) were prepared through addition of 5 μL of 6X Gel Loading Dye (New England Biolabs, MA, USA) supplemented with 5 vol% 2-mercaptoethanol,

followed by incubation at 95 °C for 10 min. 15 μL of sample was then loaded to the SDS-PAGE gel. Mark12 ladder (8 μL, Thermo Fisher Scientific) was used as a protein standard for all SDS-PAGE analyses. Electrophoresis was performed using an Invitrogen (MA, USA) Mini Gel Tank and GE (MA, USA) Electrophoresis Power Supply EPS601 operating under a constant voltage manifold (165 mV) for 35 min. Gels were washed in deionised H_2O, stained with Coomassie G-250 (0.3 vol% HCl) for 20-60 min with rocking and finally de-stained with deionised H_2O for 16 h before visualization using a Bio-Rad (CA, USA) GS-900 calibrated densitometer.

Plasmid DNA Amplification and Purification

Plated DH5-α or NEB 5-α cells containing the plasmid of interest were used to inoculate LB medium (5 mL) supplemented with chloramphenicol (33 μg mL^{-1}) and grown to saturation at 37 °C over 16 h with orbital shaking (150 rpm). After 16 h, cells were harvested by centrifugation (8000 x g, 10 min, 4 °C) and the supernatant decanted. Cells were then lysed and purified using either a GeneJET plasmid miniprep kit (Thermo Fisher Scientific, MA, USA) or a Monarch plasmid miniprep kit (New England Biolabs, MA, USA) according to standard protocols albeit with elution in sterile nuclease-free H_2O (30 μL). Purified plasmid DNA was quantified by NanoDrop (Thermo Fisher Scientific, MA, USA) UV-Vis spectroscopy at 260/280 nm and all samples were stored at -20 °C until further use.

DNA Gel Purification

DNA fragment and/or plasmids were separated by AGE according to standard procedures (*see above*). Bands of interest were excised from the gel under UV transillumination (UV transilluminator 2000, Bio-Rad, CA, USA) using a scalpel blade. Excised bands were then digested and purified using either a GeneJET gel extraction kit (Thermo Fisher Scientific, MA, USA) or a Monarch gel kit (New England Biolabs, MA, USA) according to standard protocols albeit with elution in sterile nuclease-free H_2O (30 μL). Purified plasmid DNA was quantified by NanoDrop (Thermo Fisher Scientific, MA, USA) UV-Vis spectroscopy at 260/280 nm and all samples were stored at -20 °C.

Preparative Fast Protein Liquid Chromatography (FPLC)

All recombinant protein purifications were performed using columns and buffer systems as specified on a GE (MA, USA) ÄKTA Pure FPLC system at 4 °C.

Photocatalytic Diselenide Contraction (PDC) Reactions

All PDC reactions were as specified for individual experiments in a Penn Optical Coatings (PA, USA) M1 photoreactor using a 450 nm LED light source with incubation at 25-37 ºC. Protein samples were prepared under a stream of N_2. Reaction time points were quenched by removal from the light source and dilution in either 0.1 vol% TFA in H_2O or 6 M Gnd.HCl, 100 mM NaP_i, pH 7.4 buffer.

Synthesis of Selenocysteine Variants

1. **[Boc-Sec-OH]$_2$:** L-selenocystine (1.5 g, 4.5 mmol, 1.0 eq.) was dissolved in H_2O (20 mL) and treated with Et_3N (1.88 mL, 13.5 mmol, 3.0 eq.). After cooling the reaction mixture to 0 °C, Boc_2O (2.95 g, 13.5 mmol, 3.0 eq.) was added in small portions and the reaction was stirred at rt for 16 h. After complete conversion, 0.5 M HCl was added to the reaction mixture, followed by EtOAc extraction (3 x 60 mL). The combined EtOAc layers were dried over Na_2SO_4 and concentrated in vacuo. The crude product was purified using flash column chromatography (19:1, CH_2Cl_2:MeOH with 1 vol.% AcOH) to afford [Boc-Sec-OH]$_2$ as a yellow solid (1.8 g, 3.37 mmol, 75%). The characterization data was in accordance with the previously reported data in the literature.[1]

2. Fmoc-Sec(Bn)-OH: To a solution of Boc-L-Selenocystine (1.13 g, 2.1 mmol, 1 eq.) and benzyl bromide (0.9 mL, 8 mmol, 3.8 eq.) in THF (28 mL) and ethanol (8.5 mL) at 0 ºC was added $NaBH_4$ (240 mg, 6.3 mmol, 3 eq.). The mixture was stirred at room temperature for 1.5 h before quenching with 1 M HCl (75 mL). The aqueous phase was extracted with EtOAc (3 x 75 mL). The organic extracts were washed with brine, dried over Na_2SO_4 and concentrated *in vacuo*. The residue was purified by flash chromatography on silica gel (0–100% EtOAc in hexanes) to give the title compound (1.20 g, 3.3 mmol, 80%) as a yellow oil.

To a solution of Boc-Sec(Bn)-OH (500 mg, 1.40 mmol) in CH_2Cl_2 (10 mL) was added 4 M HCl in dioxane (3.5 mL, 13 mmol). The solution was stirred at room temperature for 1.5 h before concentrating *in vacuo*. The residue was dissolved in a mixture of THF (10 mL) and saturated aqueous $NaHCO_3$ (5 mL) before Fmoc-OSu (520 mg, 1.5 mmol) was added. The mixture was stirred at room temperature for 18 h before water (75 mL) was added and acidified to pH 3 with 1 M HCl. The aqueous phase was extracted with Et_2O (3 x 75 mL). The organic extracts were washed with brine (75 mL), dried over Na_2SO_4 and concentrated *in vacuo*. The residue was purified by flash chromatography on silica gel (0–100% EtOAc in hexanes) to give the title compound (624 mg, 1.30 mmol, 93%) as a pale-yellow solid. The characterization data was in accordance with the previously reported data in the literature.[2]

Synthesis of Model Selenopeptide [H_2N-USPGYS-NH_2]$_2$

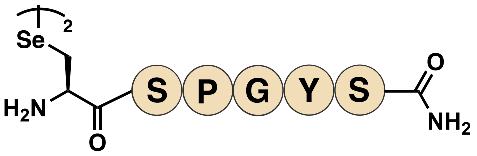

[H_2N-USPGYS-NH_2]$_2$ diselenide (1)

Rink amide resin (666 mg, 0.3 mmol g^{-1}) was swollen in CH_2Cl_2 (5 mL), then washed with DMF (5 x 2 mL). The resin was transferred to an Biotage Syro I automated peptide synthesizer for iterative coupling of Ser, Tyr, Gly, Pro and Ser (see *General Procedures*). The resin was then removed from the synthesizer for manual Fmoc-deprotection with 20 vol% piperidine in DMF (3 x 2 mL x 3 min). [Boc-Sec-OH]$_2$ (100 μmol, 0.5 eq.) was then coupled with DIC (200 μmol, 1 eq.) and HOAt (400 μmol, 2 eq.) in DMF (2 mL) at room temperature for 16 h. The complete peptide was then deprotected and cleaved from resin through treatment with 90:5:5 v/v/v TFA:*i*Pr_3SiH:H_2O (2 mL) for 2 h at room temperature. The cleavage solution was added to a 50 mL centrifuge tube and worked up according to standard procedures (see *General Procedures*). The crude peptide pellet was then redissolved in 0.1 vol% TFA in H_2O (5 mL) for purification by RP-HPLC (0-25% B over 40 min at 38 mL min^{-1}, Waters XBridge 5 μm, 30 x 150 mm, λ = 214 nm). Purity of fractions was analysed by UPLC-MS and pure fractions were lyophilized to afford [H_2N-USPGYS-NH_2]$_2$ (**1**) as a diselenide dimer (42.5 mg, 27.55 μmol, 28% from theoretical resin loading of 200 μmol).

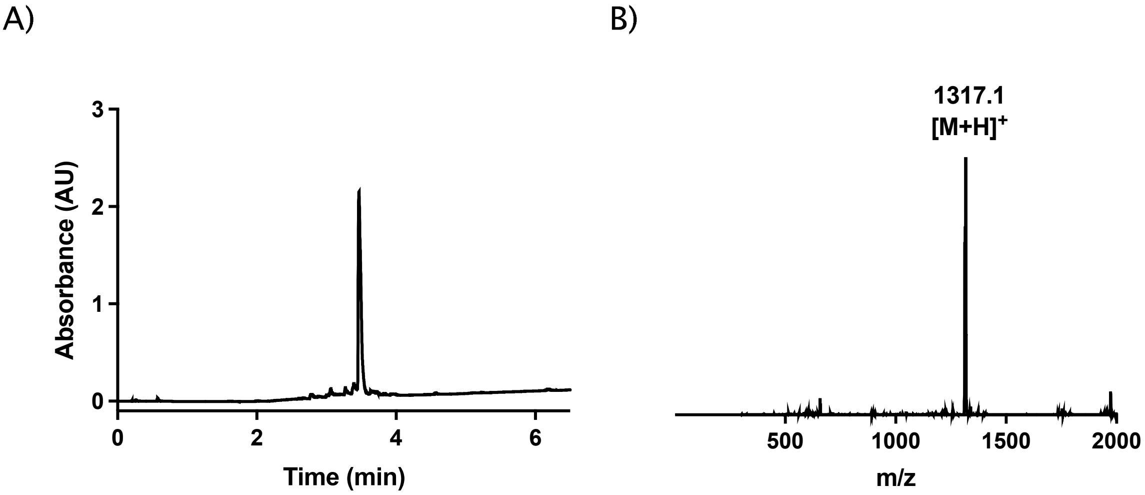

Supplementary Figure 1. A) UPLC trace (0-40% B over 5 min at 0.6 mL min^{-1}, 0.1% v/v TFA, λ = 214 nm; Waters Acquity BEH300 C18 1.7 μm, 2.1 x 50 mm) of purified [H_2N-USPGYS-NH_2]$_2$ (1). **B)** Associated MS (ESI+) spectrum at Rt = 3.47 min.

Initial Photocatalytic Deselenization Studies

[H_2N-USPGYS-NH_2]$_2$ diselenide (**1**) (0.825 mg, 0.535 μmol) was dissolved in H_2O (54 μL) containing Eosin Y (0.1 μmol mL^{-1}) for a photocatalyst loading of 1 mol%. This solution was then further diluted with H_2O (374 μL) to bring the total volume to 428 μL for a peptide concentration of 1.25 mM (relative to the diselenide dimer **1**). This solution was used to dissolve TCEP (0.61 mg, 2.14 μmol, 4 equiv.) and the resulting reaction mixture was irradiated with 450 nm light for the following time points: 0, 1, 2, 4, 8, 16, 32 and 64 min. At each time point, a 5 μL aliquot of the reaction mixture was taken and diluted 5-fold into H_2O (0.1 vol% formic acid). Reaction analysis was performed on these aliquots using UPLC and UPLC-MS on a gradient of 0-20% B over 5 min (λ = 214 nm).

Complete deselenization was observed after 64 min irradiation, affording H_2N-ASPGYS-NH_2 (**2**), alongside oxidative deselenization by-product H_2N-SSPGYS-NH_2 (*Supplementary Figure 2*). Selenoether dimer (**3**) was observed at the same retention time as the [H_2N-USPGYS-NH_2]$_2$ diselenide dimer (**1**) starting material (*Supplementary Figure 2*) and was identified by MS analysis (*Supplementary Figure 3*).

See next page for analytical data.

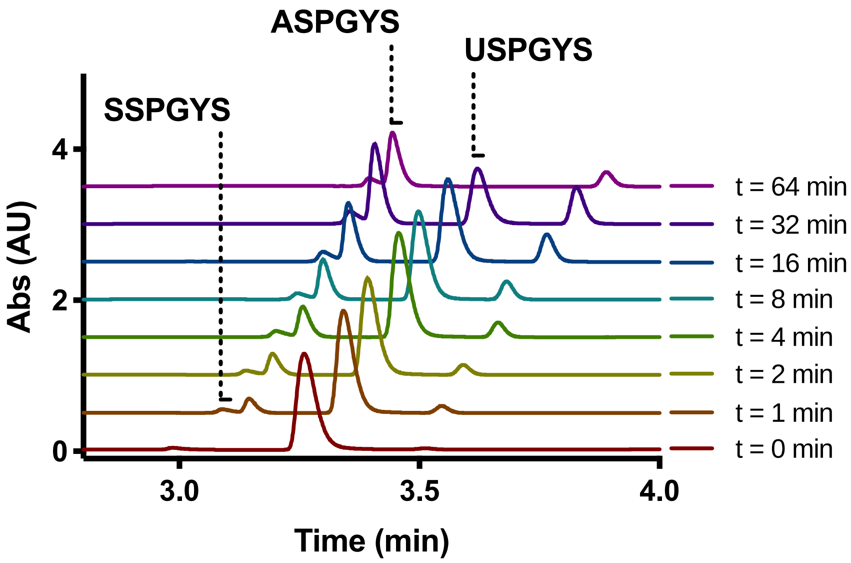

Supplementary Figure 2. Time point UPLC analysis (0-20% B over 5 min, Waters Acquity BEH300 C18 1.7 μm, 2.1 x 50 mm, λ = 214 nm) of initial studies into the use of Eosin Y (1 mol%) and TCEP (4 equiv.) for the photocatalytic deselenization of a model peptide diselenide [H_2N-USPGYS-NH_2]$_2$ (**1**).

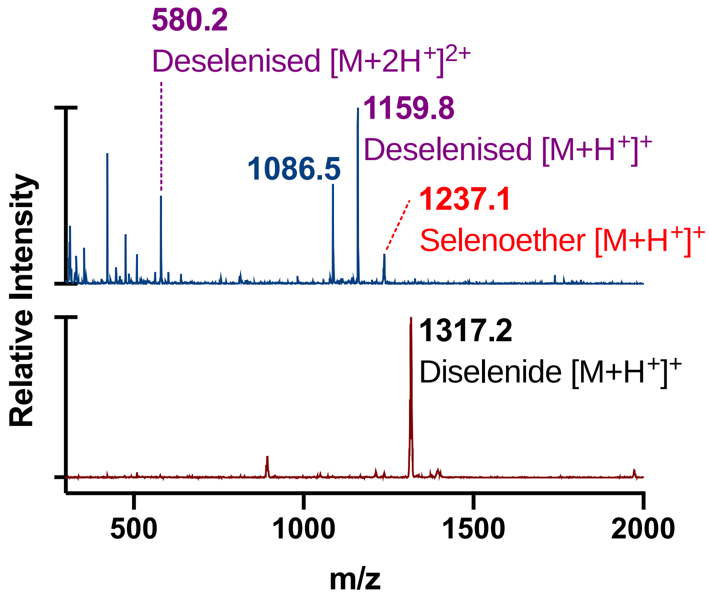

Supplementary Figure 3. Associated MS (ESI+) spectrum of reaction mixture at t = 32 min, mass peaks for deselenized product **2** are shown in black, selenoether **3** peaks are shown in red and by-product peak in blue.

Model System Optimization Studies

Photocatalyst Screen

$[H_2N\text{-USPGYS-}NH_2]_2$ diselenide (**1**) (0.825 mg, 0.535 μmol) was dissolved in 5 vol% formic acid in MeCN* (54 μL) containing either one of the following photocatalysts for a loading of 1 mol%:

1. $[Ru(bpy)_3]Cl_2$ (0.1 μmol mL^{-1})
2. $[Ru(bpz)_3](PF_6)_2$ (0.1 μmol mL^{-1})
3. $[Ru(phen)_3](PF_6)_2$ (0.1 μmol mL^{-1})
4. Eosin Y (0.1 μmol mL^{-1})
5. 4-CzIPN (0.1 μmol mL^{-1})
6. $[Ir(ppy)_3]$ (0.1 μmol mL^{-1})
7. $[Ir(dF\text{-}ppy)_3]$ (0.1 μmol mL^{-1})
8. $[Ir(ppy)_2(dtbpy)]PF_6$ (0.1 μmol mL^{-1})
9. $[Ir(dF(Me)ppy)_2(dtbbpy)]PF_6$ (0.1 μmol mL^{-1})
10. $[Ir(dF(CF_3)ppy)_2(dtbpy)]PF_6$ (**5**) (0.1 μmol mL^{-1})

These solutions were then further diluted with MeCN (374 μL) to bring the total volume to 428 μL for a concentration of **1** of 1.25 mM (relative to the diselenide dimer). These solutions were used to dissolve the PPh_3 (0.56 mg, 2.14 μmol, 4 eq.)** and the resulting reaction mixtures were irradiated with 450 nm light for the following time points: 0, 1, 2, 4, 8 and 16 min. At each time point, a 5 μL aliquot of the reaction mixture was taken and diluted 5-fold into H_2O (0.1 vol% FA). Reaction analysis was performed on these aliquots using HPLC, UPLC and UPLC-MS on gradients of 0-20% B over 5 min or 1-20% B over 30 min (λ = 214 nm).

Partial conversions of the starting material diselenide **1** to selenoether **3** were observed over the 16 min time course for reactions employing Eosin Y, $[Ir(dF(Me)ppy)_2(dtbbpy)]PF_6$, and $[Ir(dF(CF_3)ppy)_2(dtbpy)]PF_6$ (**5**).

* 5 vol% formic acid was added to aid peptide solubility in MeCN

** PPh_3 used as an alternative phosphine to TCEP due to poor solubility of TCEP in MeCN

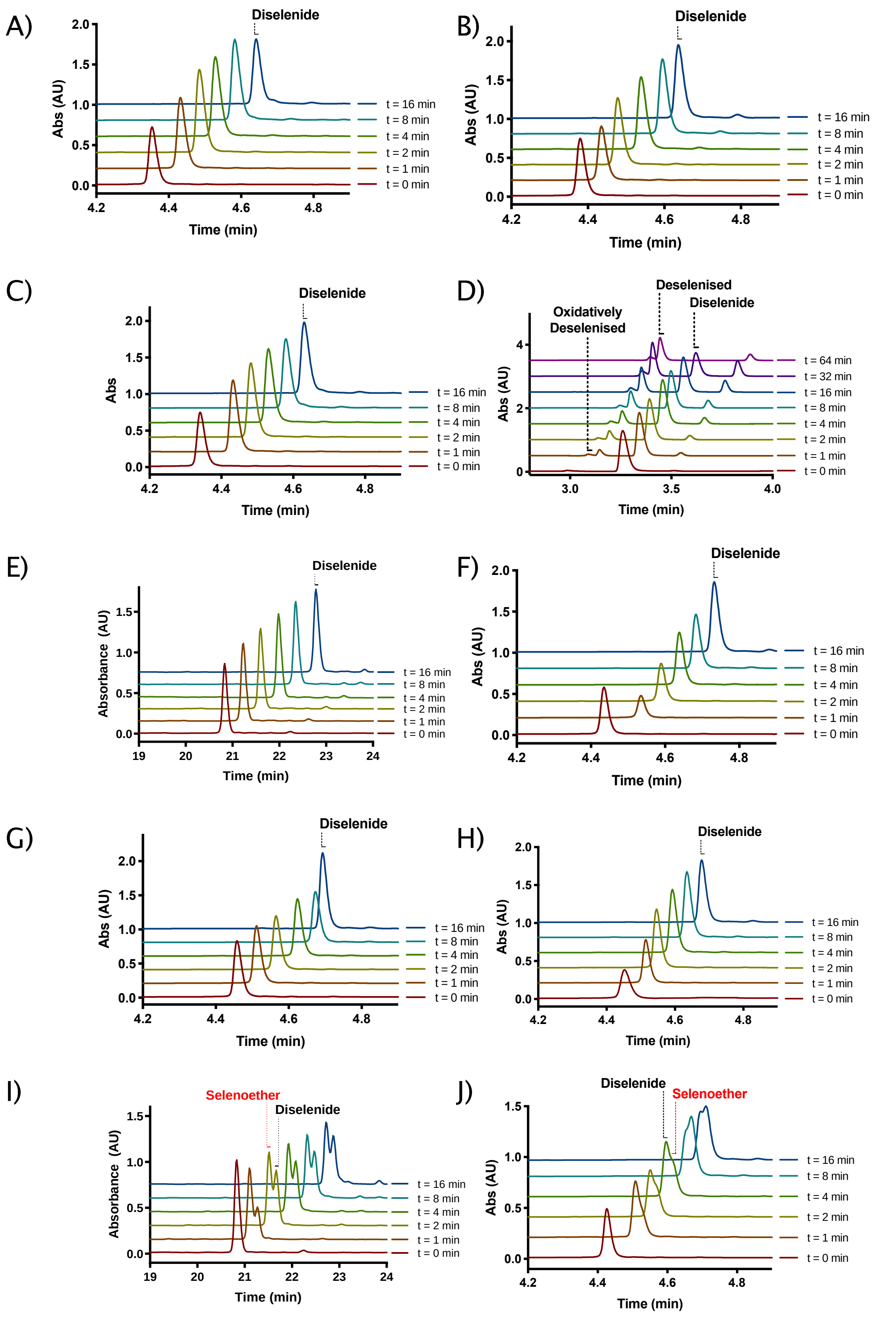

Supplementary Figure 4. UPLC/HPLC analysis of the dimerization of a model peptide diselenide [H_2N-USPGYS-NH_2]$_2$ (**1**) employing PPh_3 (4 eq.) and the photocatalyst (1 mol%): **A)** [Ru(bpy)$_3$]Cl_2, **B)** [Ru(bpz)$_3$](PF_6)$_2$, **C)** [Ru(phen)$_3$](PF_6)$_2$, **D)** Eosin Y, **E)** 4-CzIPN **F)** [Ir(ppy)$_3$], **G)** [Ir(dFppy)$_3$], **H)** [Ir(ppy)$_2$(dtbpy)]PF_6, **I)** [Ir(dF(Me)ppy)$_2$(dtbbpy)]PF_6 and **J)** [Ir(dF(CF_3)ppy)$_2$(dtbpy)]PF_6 (**5**). Note: a gradient of 0-20% B over 5 min was used in all cases except for **E)** and **I)**, which used a gradient of 1-20% B over 30 min. λ = 214 nm.

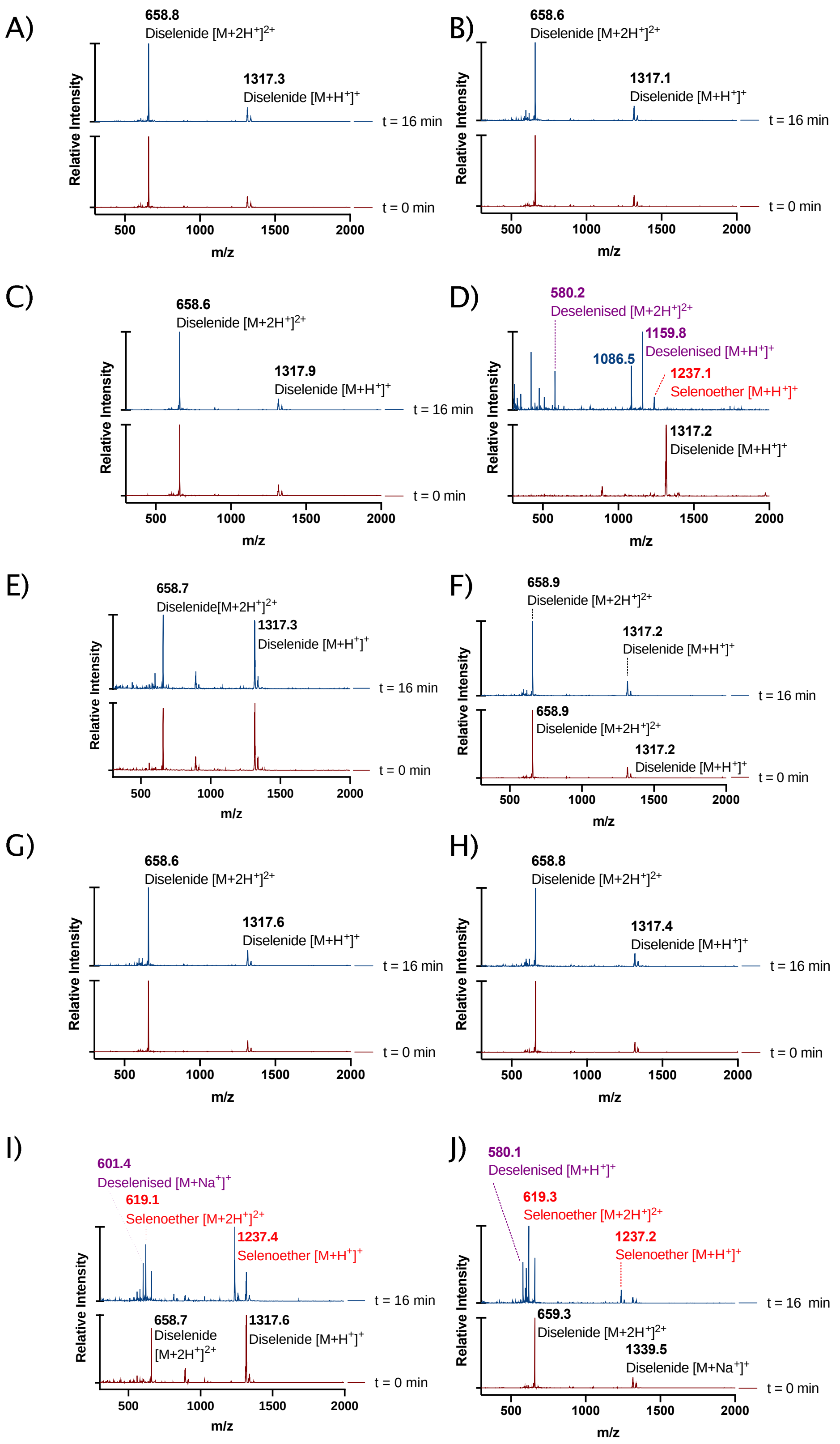

Supplementary Figure 5. Overlaid associated mass spectra of the 0 min (red) and 16 min (blue) time points taken from the dimerization of a model peptide diselenide $[H_2N\text{-USPGYS-NH}_2]_2$ (**1**) employing PPh_3 (4 eq.) and the photocatalyst (1 mol%): **A)** $[Ru(bpy)_3]Cl_2$, **B)** $[Ru(bpz)_3](PF_6)_2$, **C)** $[Ru(phen)_3](PF_6)_2$, **D)** Eosin Y, **E)** 4-CzIPN **F)** $[Ir(ppy)_3]$, **G)** $[Ir(dFppy)_3]$, **H)** $[Ir(ppy)_2(dtbpy)]PF_6$, **I)** $[Ir(dF(Me)ppy)_2(dtbbpy)]PF_6$ and **J)** $[Ir(dF(CF_3)ppy)_2(dtbpy)]PF_6$ (**5**).

Supplementary Table 1. Quantified results from the photocatalyst screen for the generation of selenoether **3** with PPh_3 (4 equiv.) in MeCN (2.5 mM) and under 450 nm irradiation. Conversions calculated by TIC integration of $[H_2N\text{-USPGYS-}NH_2]_2$ diselenide **1**, selenoether **3** and $H_2N\text{-ASPGYS-}NH_2$ (deselenized) **2** species, and division of a single species by the integration of total peptidic material.

Photocatalyst	Phos phine	Phosphine Equiv.	Solvent	Conc.[§]	Conversion
**Eosin Y**	TCEP	4	1:1 v/v H_2O:MeCN	1.25 mM	**Quant.** *10% selenoether* *90% deselenized*
**4-CzIPN**	PPh_3	4	MeCN	1.25 mM	**No Conversion**
**$[Ir(ppy)_3]$**	PPh_3	4	MeCN	1.25 mM	**No Conversion**
**$[Ir(dF(CF_3)ppy)_2(dtbpy)]PF_6$ (5)**	PPh_3	4	MeCN	1.25 mM	**73%** *69% selenoether* *31% deselenized*
**$[Ir(dF(Me)ppy)_2(dtbbpy)]PF_6$**	PPh_3	4	MeCN	1.25 mM	**78%** *56% selenoether* *44% deselenized*
**$[Ir(dFppy)_3]$**	PPh_3	4	MeCN	1.25 mM	**No Conversion**
**$[Ir(ppy)_2(dtbpy)]PF_6$**	PPh_3	4	MeCN	1.25 mM	**No Conversion**
**$[Ru(bpy)_3]Cl_2$**	PPh_3	4	MeCN	1.25 mM	**No Conversion**
**$[Ru(phen)_3](PF_6)_2$**	PPh_3	4	MeCN	1.25 mM	**No Conversion**
**$[Ru(bpz)_3](PF_6)_2$**	PPh_3	4	MeCN	1.25 mM	**No Conversion**

[§] All concentrations are quoted with respect to $[H_2N\text{-USPGYS-}NH_2]_2$ diselenide dimer **1**

Phosphine Screen

[H_2N-USPGYS-NH_2]$_2$ diselenide (**1**) (0.825 mg, 0.535 μmol) was dissolved in either 5 vol% formic acid in MeCN* (54 μL) or 1:1 v/v H_2O:MeCN (54 μL) containing [Ir(dF(CF_3)ppy)$_2$(dtbpy)]PF_6 (**5**) (0.1 μmol mL^{-1}) for a photocatalyst loading of 1 mol%. These solutions were then further diluted with either 5 vol% formic acid in MeCN (374 μL) or H_2O (374 μL) to bring the total volume to 428 μL for a concentration of **1** of 1.25 mM (relative to the diselenide dimer). All solutions were used to solvate one of the following phosphines:

1. PPh_3 (0.56 mg, 2.14 μmol, 4 eq.)
2. tri(*o*-tolyl)phosphine (TTP) (0.65 mg, 2.14 μmol, 4 eq.)
3. *tris*(pentafluorophenyl)phosphine (TPFPP) (1.14 mg, 2.14 μmol, 4 eq.)
4. *tris*(4-methoxyphenyl)phosphine (TMPP) (0.75 mg, 2.14 μmol, 4 eq.)
5. *tris*(2,4,6-trimethoxyphenyl)phosphine (TTMPP) (1.14 mg, 2.14 μmol, 4 eq.)
6. 3,3',3''-phosphinetriyltribenzenesulfonate trisodium salt (TPPTS) (1.22 mg, 2.14 μmol, 4 eq.)
7. *tris*(2-furyl)phosphine (TFP) (0.50 mg, 2.14 μmol, 4 eq.)
8. 1,3,5-triaza-7-phosphaadamantane (PTA) (**4**) (0.34 mg, 2.14 μmol, 4 eq.)

The resulting reaction mixtures were irradiated with 450 nm light for the following time points: 0, 1, 2, 4, 8 and 16 min. At each time point, a 5 μL aliquot of the reaction mixture was taken and diluted 5-fold into H_2O (0.1 vol% formic acid). Reaction analysis was performed on these aliquots using UPLC and UPLC-MS on gradients of 0-20% B over 5 min (λ = 214 nm).

Partial conversions of the starting material diselenide **1** to selenoether **3** were observed over the 16 min time course for reactions employing either PPh_3, and near-quantitative conversions were observed for reactions employing TPPTS or PTA (**4**).

* 5 vol% formic acid was added to aid peptide solubility in MeCN

See next pages for analytical data.

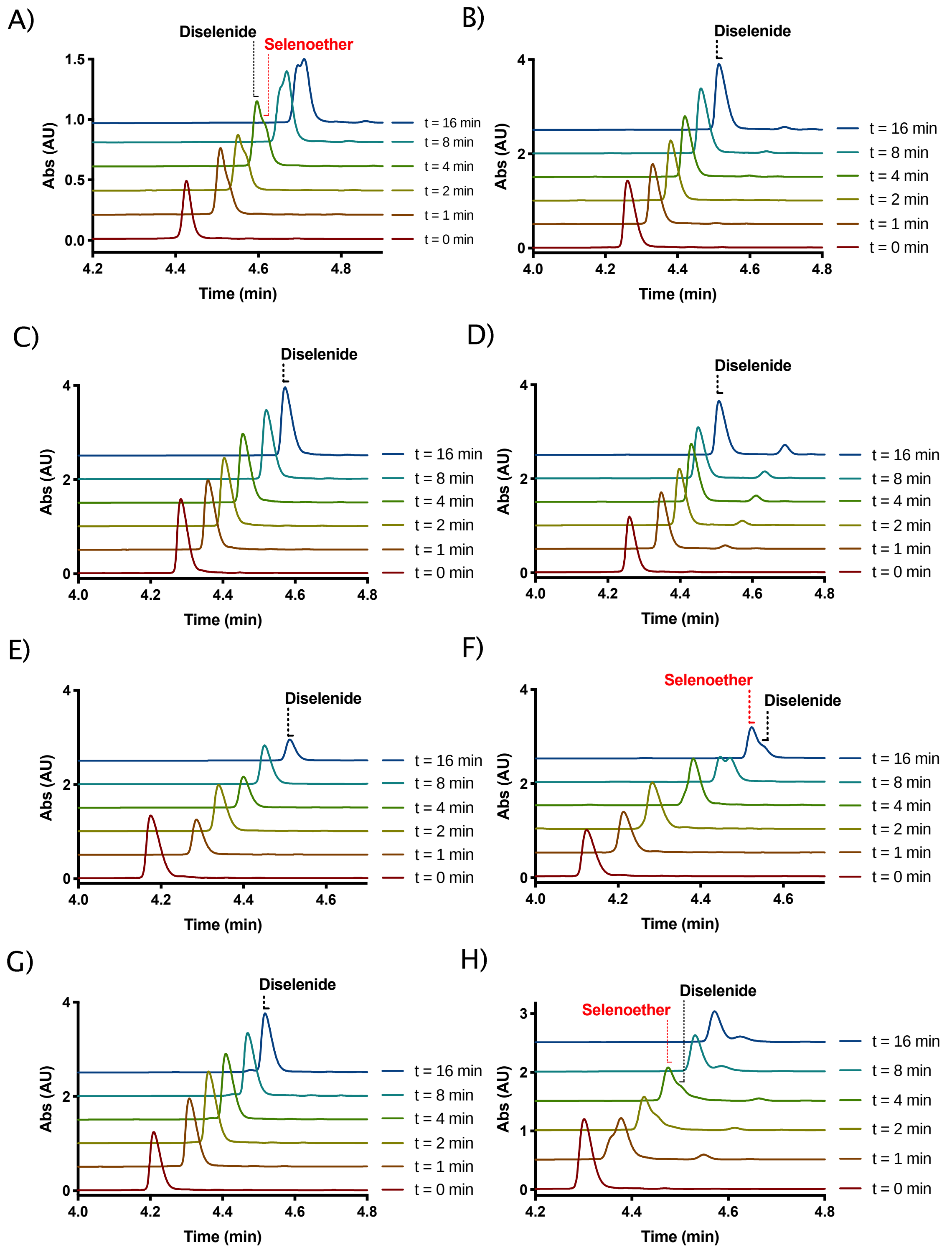

Supplementary Figure 6. UPLC analysis (0-20% B over 5 min, Waters Acquity BEH300 C18 1.7 μm, 2.1 x 50 mm, λ = 214 nm) of the dimerization of a model peptide diselenide [H_2N-USPGYS-NH_2]$_2$ (**1**) employing the photocatalyst [Ir(dF(CF_3)ppy)$_2$(dtbpy)]PF_6 (**5**) (1 mol%) and the phosphine: **A)** PPh_3, **B)** TTP, **C)** TPFPP, **D)** TMPP, **E)** TTMPP, **F)** TPPTS, **G)** TFP, and **H)** PTA (**4**).

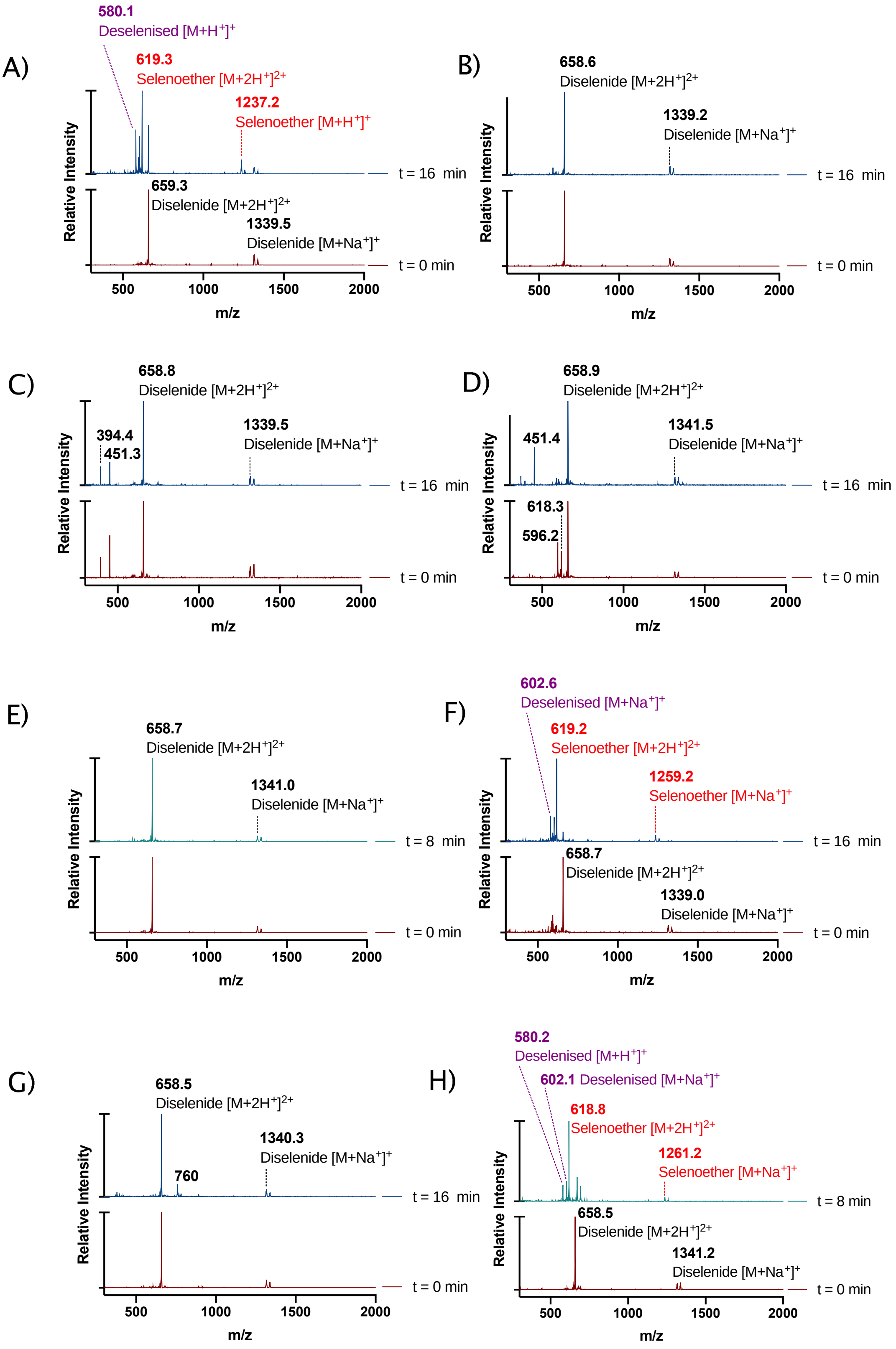

Supplementary Figure 7. Overlaid associated mass spectra of the 0 min (red) and 16 min (blue) time points taken from the dimerization of a model peptide diselenide [H_2N-USPGYS-NH_2]$_2$ (**1**) employing the photocatalyst [Ir(dF(CF_3)ppy)$_2$(dtbpy)]PF_6 (**5**) (1 mol%) and the phosphine: **A)** PPh_3, **B)** TTP, **C)** TPFPP, **D)** TMPP, **E)** TTMPP, **F)** TPPTS, **G)** TFP, and **H)** PTA (**4**).

Supplementary Table 2. Quantified results from the optimization of phosphine additive for the generation of selenoether **3** with $[Ir(dF(CF_3)ppy)_2(dtbpy)]^{3+}$ (**5**) photocatalyst in either MeCN or 1:1 v/v H_2O:MeCN (2.5 mM) and under 450 nm irradiation. Conversions calculated by TIC integration of $[H_2N\text{-}USPGYS\text{-}NH_2]_2$ diselenide **1**, selenoether **3** and H_2N-ASPGYS-NH_2 (deselenized) **2** species, and division of a single species by the integration of total peptidic material.

Phosphine	Photocatalyst	Phosphine Equiv.	Solvent	Conc. §	Conversion
**PPh_3**	$[Ir(dF(CF_3)ppy)_2(dtbpy)]$ PF_6 (**5**)	4	MeCN	1.25 mM	**73%** *69% selenoether* *31% deselenized*
**TTP**	$[Ir(dF(CF_3)ppy)_2(dtbpy)]$ PF_6 (**5**)	4	MeCN	1.25 mM	**No Conversion**
**TPFPP**	$[Ir(dF(CF_3)ppy)_2(dtbpy)]$ PF_6 (**5**)	4	MeCN	1.25 mM	**No Conversion**
**TMPP**	$[Ir(dF(CF_3)ppy)_2(dtbpy)]$ PF_6 (**5**)	4	MeCN	1.25 mM	**No Conversion**
**TTMPP**	$[Ir(dF(CF_3)ppy)_2(dtbpy)]$ PF_6 (**5**)	4	1:1 v/v H_2O:MeCN	1.25 mM	**No Conversion**
**TPPTS**	$[Ir(dF(CF_3)ppy)_2(dtbpy)]$ PF_6 (**5**)	4	1:1 v/v H_2O:MeCN	1.25 mM	**92%** *78% selenoether* *22% deselenized*
**TFP**	$[Ir(dF(CF_3)ppy)_2(dtbpy)]$ PF_6 (**5**)	4	1:1 v/v H_2O:MeCN	1.25 mM	**No Conversion**
**PTA (4)**	$[Ir(dF(CF_3)ppy)_2(dtbpy)]$ PF_6 (**5**)	4	1:1 v/v H_2O:MeCN	1.25 mM	**Quant.** *69% selenoether* *31% deselenized*

§ All concentrations are quoted with respect to $[USPGYS]_2$ diselenide dimer **1**

Phosphine Equivalents Screen

$[H_2N\text{-}USPGYS\text{-}NH_2]_2$ diselenide (**1**) (0.825 mg, 0.535 μmol) was dissolved in 1:1 v/v H_2O:MeCN (54 μL) containing $[Ir(dF(CF_3)ppy)_2(dtbpy)]PF_6$ (**5**) (0.1 μmol mL^{-1}) for a photocatalyst loading of 1 mol%. These solutions were then further diluted with H_2O (374 μL) to bring the total volume to 428 μL for a concentration of **1** of 1.25 mM (relative to the diselenide dimer). All solutions were used to solvate one of the following PTA (**4**) in the following quantities:

1. 0.085 mg, 0.535 μmol, 1 eq.
2. 0.17 mg, 1.07 μmol, 2 eq.
3. 0.34 mg, 2.14 μmol, 4 eq.
4. 0.68 mg, 4.28 μmol, 8 eq.

The resulting reaction mixtures were irradiated with 450 nm light for the following time points: 0, 1, 2, 4, 8 and 16 min. At each time point, a 5 μL aliquot of the reaction mixture was taken and diluted 5-fold into H_2O (0.1 vol% formic acid). Reaction analysis was performed on these aliquots using UPLC and UPLC-MS on gradients of 0-20% B over 5 min (λ = 214 nm).

No conversion of diselenide **1** to selenoether **3** was observed for reactions employing a single equivalent of PTA (**4**). Partial conversions (~50%) were observed for reactions employing 2 equivalents of PTA (**4**). Quantitative conversions were observed for reactions employing either 4 or 8 equivalents of PTA (**4**), albeit with significantly more deselenization in the case of the 8 equivalent reactions.

See next pages for analytical data.

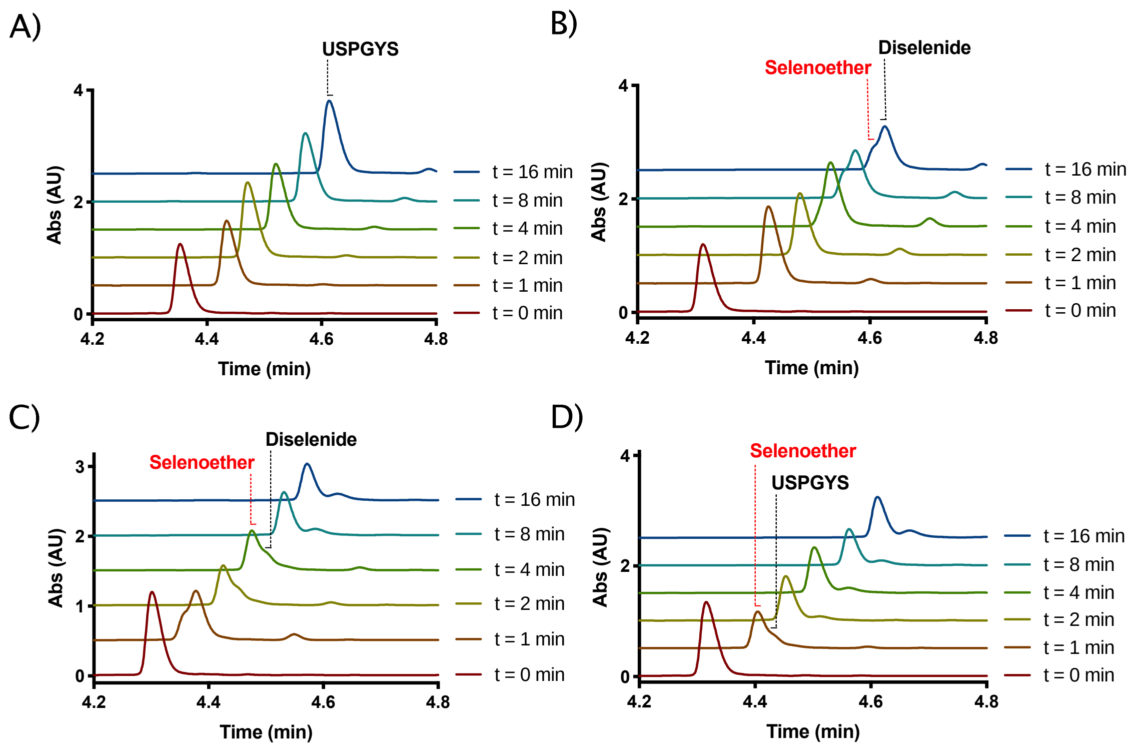

Supplementary Figure 8. UPLC analysis (0-20% B over 5 min, Waters Acquity BEH300 C18 1.7 µm, 2.1 x 50 mm, λ = 214 nm) of the dimerization of a model peptide diselenide [H_2N-USPGYS-NH_2]$_2$ (**1**) employing the photocatalyst [Ir(dF(CF_3)ppy)$_2$(dtbpy)]PF_6 (**5**) (1 mol%) and PTA (**4**) in the quantities: **A)** 1 eq., **B)** 2 eq., **C)** 4 eq., **D)** 8 eq.

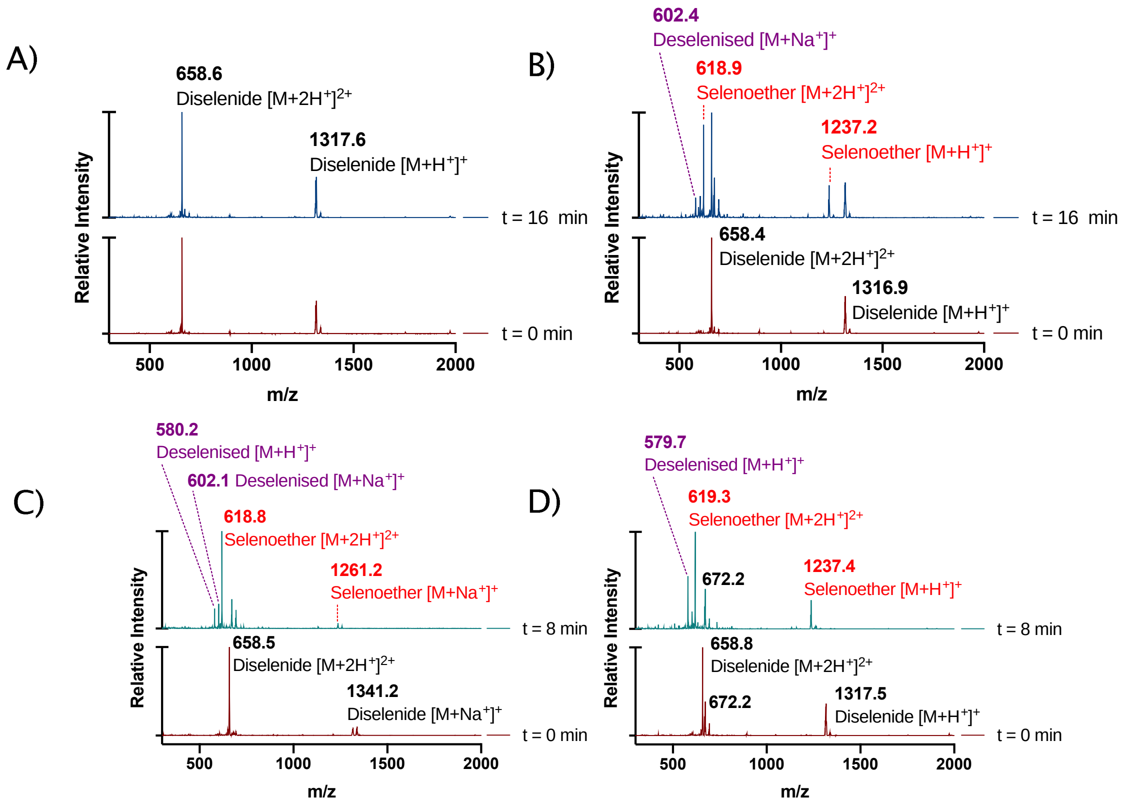

Supplementary Figure 9. Overlaid associated mass spectra of the 0 min (red) and 16 min (blue) time points taken from the dimerization of a model peptide diselenide $[H_2N\text{-USPGYS-}NH_2]_2$ (**1**) employing the photocatalyst $[Ir(dF(CF_3)ppy)_2(dtbpy)]PF_6$ (**5**) (1 mol%) and PTA (**4**) in the quantities: **A)** 1 eq., **B)** 2 eq., **C)** 4 eq., **D)** 8 eq.

Supplementary Table 3. Quantified results from the optimization of phosphine equivalents for the generation of selenoether **3** with $[Ir(dF(CF_3)ppy)_2(dtbpy)]PF_6$ (**5**) photocatalyst in 1:1 v/v H_2O:MeCN (2.5 mM) under 450 nm irradiation. Conversions calculated by TIC integration of $[H_2N\text{-USPGYS-}NH_2]_2$ diselenide **1**, selenoether **3** and $H_2N\text{-ASPGYS-}NH_2$ (deselenized) **2**, and division of a single species by the integration of total peptidic material.

Phosphine	Photocatalyst	4 Equiv.	Solvent	Conc. §	Conversion
PTA (**4**)	$[Ir(dF(CF_3)ppy)_2(dtbpy)]PF_6$ (**5**)	**1**	1:1 v/v H_2O:MeCN	1.25 mM	**No Conversion**
PTA (**4**)	$[Ir(dF(CF_3)ppy)_2(dtbpy)]PF_6$ (**5**)	**2**	1:1 v/v H_2O:MeCN	1.25 mM	**51%** *85% selenoether* *15% deselenized*
PTA (**4**)	$[Ir(dF(CF_3)ppy)_2(dtbpy)]PF_6$ (**5**)	**4**	1:1 v/v H_2O:MeCN	1.25 mM	**Quant.** *69% selenoether* *31% deselenized*
PTA (**4**)	$[Ir(dF(CF_3)ppy)_2(dtbpy)]PF_6$ (**5**)	**8**	1:1 v/v H_2O:MeCN	1.25 mM	**Quant.** *70% selenoether* *30% deselenized*

§ All concentrations are quoted with respect to $[USPGYS]_2$ diselenide dimer **1**

Solvent Screen

[H_2N-USPGYS-NH_2]$_2$ diselenide (**1**) (0.825 mg, 0.535 μmol) was dissolved in 54 μL of a solution of [Ir(dF(CF_3)ppy)$_2$(dtbpy)]PF_6 (**5**) (0.1 μmol mL^{-1}) in one of the following solvent systems:

1. 1:1 v/v H_2O:MeCN
2. 1:1 v/v aqueous denaturing buffer (6 M Gn.HCl, 0.1 M Na_2HPO_4, pH 7.2):MeCN
3. 1:1 v/v H_2O:DMSO
4. 9:1 v/v H_2O:DMSO

These solutions were then further diluted with their respective solvent systems (374 μL) to bring the total volume to 428 μL for a concentration of **1** of 1.25 mM (relative to the diselenide dimer). All solutions were used to solvate PTA (**4**) (0.34 mg, 2.14 μmol, 4 eq.) and the resulting reaction mixtures were irradiated with 450 nm light for the following time points: 0, 1, 2, 4, 8 and 16 min. At each time point, a 5 μL aliquot of the reaction mixture was taken and diluted 5-fold into H_2O (0.1 vol% formic acid). Reaction analysis was performed on these aliquots using UPLC and UPLC-MS on gradients of 0-20% B over 5 min (λ = 214 nm).

Quantitative conversion of diselenide **1** to selenoether **3** was observed for reactions conducted in 1:1 v/v H_2O:MeCN and 1:1 v/v aqueous denaturing buffer (6 M Gn.HCl, 0.1 M Na_2HPO_4, pH 7.2):MeCN, albeit with higher levels of deselenization in the latter. Similar conversion was observed for reactions in 1:1 v/v H_2O:DMSO, albeit with a greater number of by-products forming throughout the reaction. No conversion was observed for reactions in 9:1 v/v H_2O:DMSO.

See next pages for analytical data.

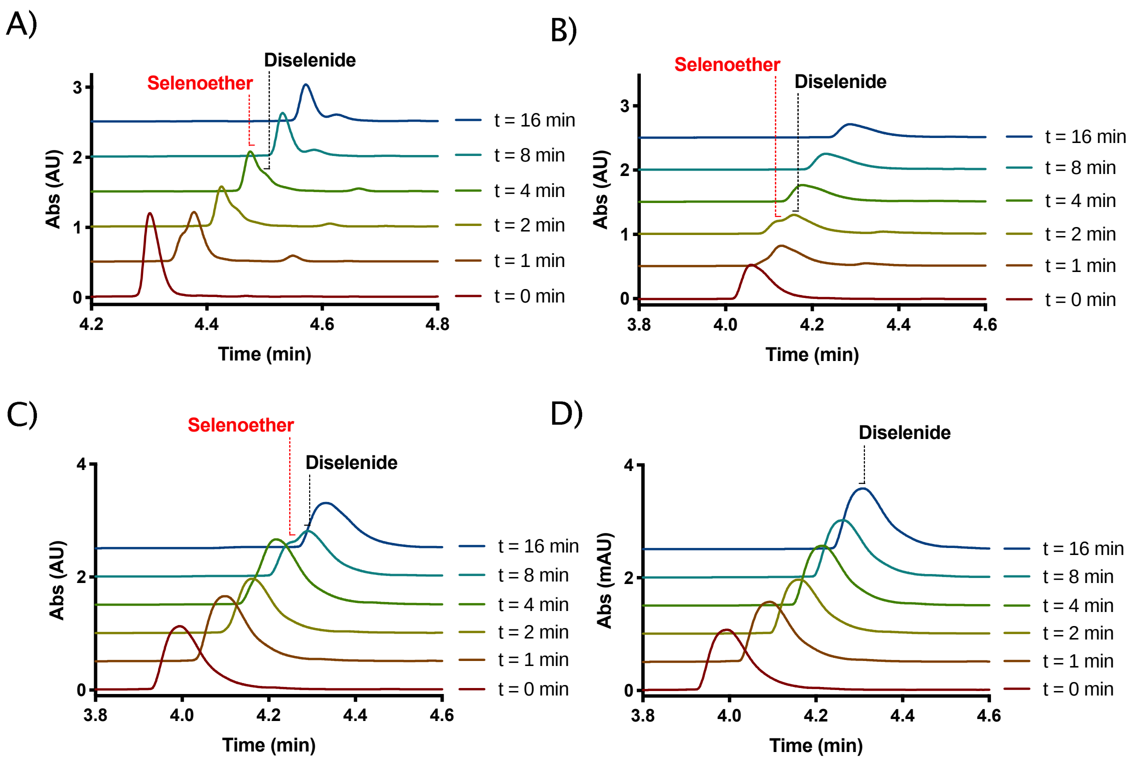

Supplementary Figure 10. UPLC analysis (0-20% B over 5 min, Waters Acquity BEH300 C18 1.7 µm, 2.1 x 50 mm, λ = 214 nm) of the dimerization of a model peptide diselenide [H_2N-USPGYS-NH_2]$_2$ (**1**) employing the photocatalyst [Ir(dF(CF_3)ppy)$_2$(dtbpy)]PF_6 (**5**) (1 mol%) and PTA (**4**) (4 eq.) in the following solvent systems: **A)** 1:1 v/v H_2O:MeCN, **B)** 1:1 v/v denaturing buffer (6 M Gn.HCl + 0.1 M Na_2HPO_4, pH 7.2):MeCN, **C)** 1:1 v/v H_2O:DMSO, and **D)** 9:1 v/v H_2O:DMSO.

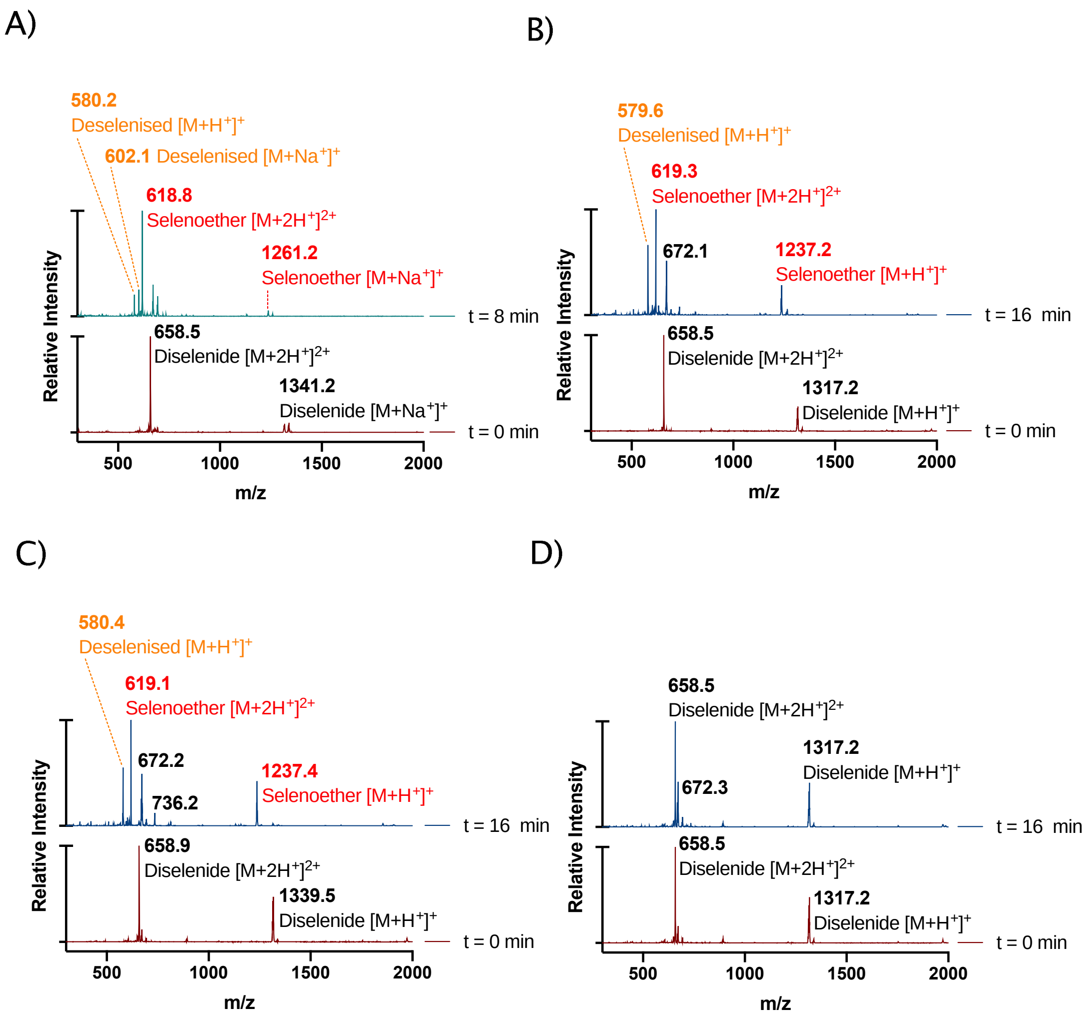

Supplementary Figure 11. Overlaid associated mass spectra of the 0 min (red) and 16 min (blue) time points taken from the dimerization of a model peptide diselenide [H_2N-USPGYS-NH_2]$_2$ (**1**) employing the photocatalyst [Ir(dF(CF_3)ppy)$_2$(dtbpy)]PF_6 (**5**) (1 mol%) and PTA (**4**) (4 eq.) in the following solvent systems: **A)** 1:1 v/v H_2O:MeCN, **B)** 1:1 v/v denaturing buffer (6 M Gn.HCl + 0.1 M Na_2HPO_4, pH 7.2):MeCN, **C)** 1:1 v/v H_2O:DMSO, and **D)** 9:1 v/v H_2O:DMSO.

Concentration Screen

[H_2N-USPGYS-NH_2]$_2$ diselenide (**1**) (0.825 mg, 0.535 μmol) was dissolved in 1:1 v/v H_2O:MeCN (54 μL) containing [Ir(dF(CF_3)ppy)$_2$(dtbpy)]PF_6 (**5**) (0.1 μmol mL^{-1}). Solutions were then further diluted with the following amounts of 1:1 v/v H_2O:MeCN to achieve set concentrations of **1** as follows:

1. 53 μL (for 5 mM [H_2N-USPGYS-NH_2]$_2$ diselenide (**1**))
2. 160 μL (for 2.5 mM [H_2N-USPGYS-NH_2]$_2$ diselenide (**1**))
3. 374 μL (for 1.25 mM [H_2N-USPGYS-NH_2]$_2$ diselenide (**1**))
4. 1.02 mL (for 0.5 mM [H_2N-USPGYS-NH_2]$_2$ diselenide (**1**))

All solutions were used to solvate PTA (**4**) (0.34 mg, 2.14 μmol, 4 eq.) and the resulting reaction mixtures were irradiated with 450 nm light for the following time points: 0, 1, 2, 4, 8 and 16 min. At each time point, aliquots of the reaction mixture were taken and diluted into H_2O (0.1 vol% FA) to achieve comparable concentrations for analysis across the dilution series. Reaction analysis was performed on these aliquots using UPLC and UPLC-MS on gradients of 0-20% B over 5 min (λ = 214 nm).

All reactions showed conversion of diselenide **1** to selenoether **3** over the course of the experiment, with more concentrated samples reaching quantitative conversions more rapidly.

See next pages for analytical data.

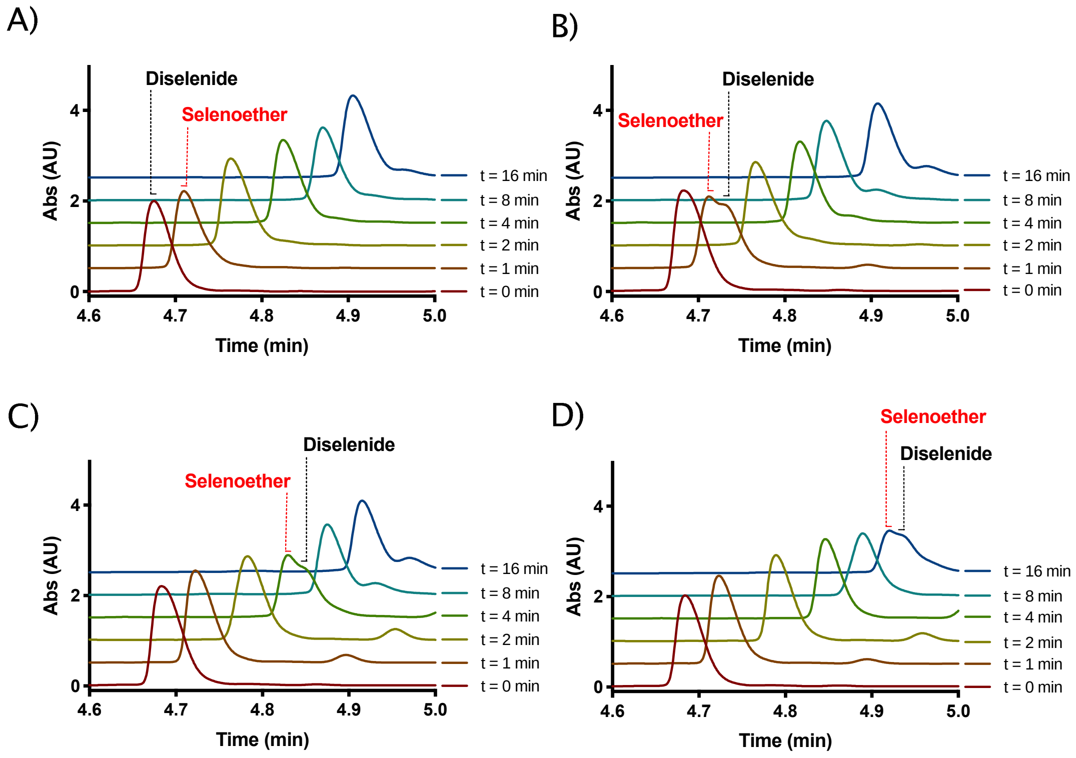

Supplementary Figure 12. UPLC analysis (0-20% B over 5 min, Waters Acquity BEH300 C18 1.7 μm, 2.1 x 50 mm, λ = 214 nm) of the dimerization of a model peptide diselenide [H_2N-USPGYS-NH_2]$_2$ (**1**) employing the photocatalyst [Ir(dF(CF_3)ppy)$_2$(dtbpy)]PF_6 (**5**) (1 mol%) and PTA (**4**) (4 eq.) in 1:1 v/v H_2O:MeCN at the following **1** concentrations: **A)** 5 mM, **B)** 2.5 mM, **C)** 1.25 mM, and **D)** 0.5 mM.

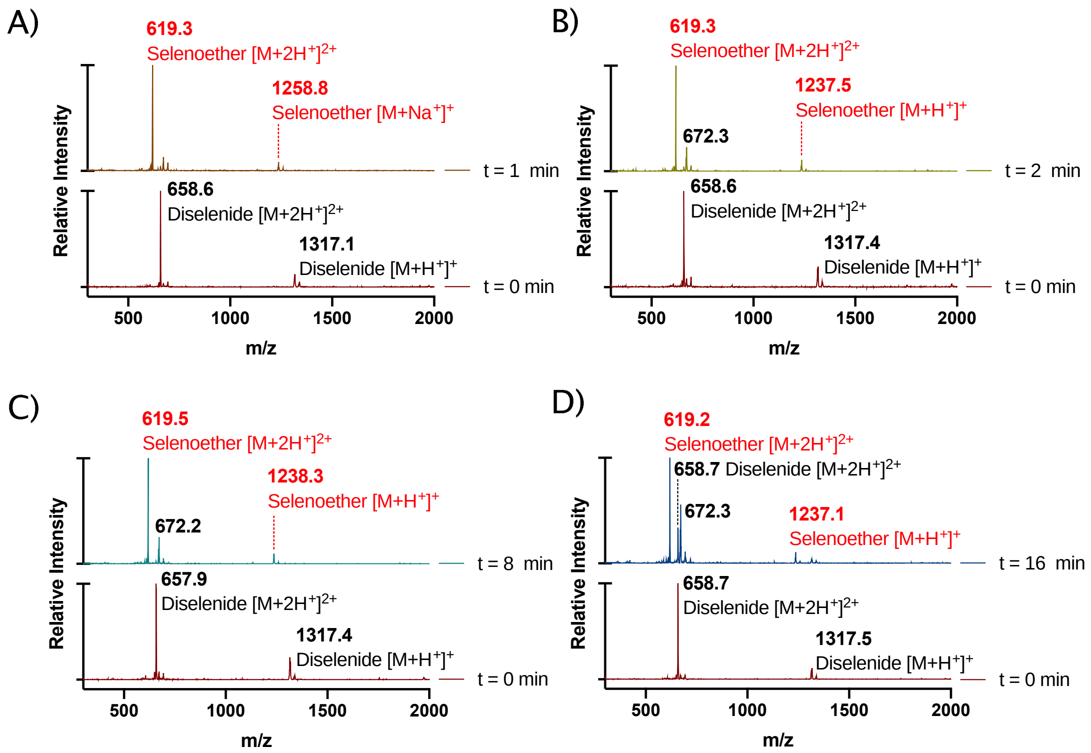

Supplementary Figure 13. Overlaid associated mass spectra of the 0 min (red) and 16 min (blue) time points taken from the dimerization of a model peptide diselenide [H_2N-USPGYS-NH_2]$_2$ (**1**) employing the photocatalyst [Ir(dF(CF_3)ppy)$_2$(dtbpy)]PF_6 (**5**) (1 mol%) and PTA (**4**) (4 eq.) in 1:1 v/v H_2O:MeCN at the following **1** concentrations: **A)** 5 mM, **B)** 2.5 mM, **C)** 1.25 mM, and **D)** 0.5 mM.

Supplementary Table 4. Quantified results from the optimization of phosphine equivalents for the generation of selenoether **3** with a $[Ir(dF(CF_3)ppy)_2(dtbpy)]PF_6$ (**5**) photocatalyst in varying solvent systems at varying concentrations and under 450 nm irradiation. Conversions calculated by TIC integration of $[H_2N\text{-USPGYS-}NH_2]_2$ diselenide **1**, selenoether **3** and $H_2N\text{-ASPGYS-}NH_2$ (deselenized) **2**, and division of a single species by the integration of total peptidic material.

**Phosphine**	**Photocatalyst**	**Phosphine Equiv.**	**Solvent**	**Conc.§**	**Conversion**
PTA (**4**)	$[Ir(dF(CF_3)ppy)_2(dtbpy)]PF_6$ (**5**)	4	1:1 v/v H_2O:MeCN	**5.0 mM**	**Quant. *(1 min)*** *100% selenoether*
PTA (**4**)	$[Ir(dF(CF_3)ppy)_2(dtbpy)]PF_6$ (**5**)	4	1:1 v/v H_2O:MeCN	**2.5 mM**	**Quant. *(2 min)*** *100% selenoether*
PTA (**4**)	$[Ir(dF(CF_3)ppy)_2(dtbpy)]PF_6$ (**5**)	4	1:1 v/v H_2O:MeCN	**1.25 mM**	**Quant. *(8 min)*** *100% selenoether*
PTA (**4**)	$[Ir(dF(CF_3)ppy)_2(dtbpy)]PF_6$ (**5**)	4	1:1 v/v H_2O:MeCN	**0.5 mM**	**77% *(16 min)*** *100% selenoether*
PTA (**4**)	$[Ir(dF(CF_3)ppy)_2(dtbpy)]PF_6$ (**5**)	4	1:1 v/v H_2O:MeCN	**0.05 mM**	**No Conversion *(16 min)***
PTA (**4**)	$[Ir(dF(CF_3)ppy)_2(dtbpy)]PF_6$ (**5**)	4	**1:1 v/v Gn_{aq}:MeCN**	1.25 mM	**Quant.** *66% selenoether* *34% deselenized*
PTA (**4**)	$[Ir(dF(CF_3)ppy)_2(dtbpy)]PF_6$ (**5**)	4	**1:1 v/v H_2O:DMSO**	1.25 mM	**Quant.** *72% selenoether* *28% deselenized*
PTA (**4**)	$[Ir(dF(CF_3)ppy)_2(dtbpy)]PF_6$ (**5**)	4	**9:1 v/v H_2O:DMSO**	1.25 mM	**No Conversion**

§ All concentrations are quoted with respect to $[USPGYS]_2$ diselenide dimer **1**

‘No Phosphine’ Control PDC Dimerization Reaction

[H_2N-USPGYS-NH_2]$_2$ diselenide **1** (0.825 mg, 0.535 μmol) was dissolved in 5 vol% formic acid in MeCN (54 μL) containing [Ir(dF(CF_3)ppy)$_2$(dtbpy)]PF_6 (**5**) (0.1 μmol mL^{-1}). The solution was then further diluted with 5 vol% formic acid in MeCN (374 μL). The solution was then irradiated with 450 nm light for 16 min. Aliquots of the reaction mixture were taken at t = 0 min and t = 16 min and diluted 5-fold into H_2O (0.1 vol% formic acid). Reaction analysis was performed on these aliquots using UPLC-MS on gradients of 0-20% B over 5 min (λ = 214 nm).

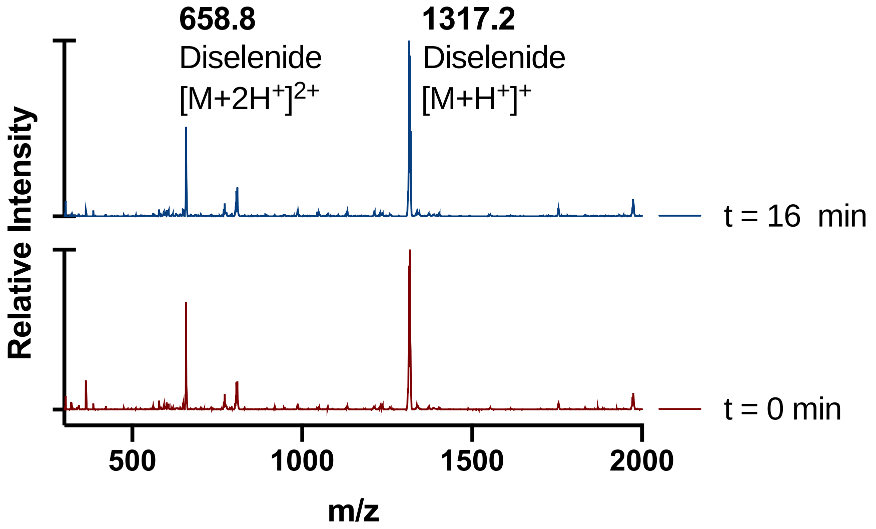

Supplementary Figure 14. Overlaid associated mass spectra of the 0 min (red) and 16 min (blue) time points taken from the negative control dimerization of a model peptide diselenide [H_2N-USPGYS-NH_2]$_2$ (**1**) employing only the photocatalyst [Ir(dF(CF_3)ppy)$_2$(dtbpy)]PF_6 (**5**) (1 mol%) in 5 vol% formic acid in MeCN.

‘No Photocatalyst’ Control PDC Dimerization Reaction

$[H_2N\text{-USPGYS-}NH_2]_2$ diselenide **1** (0.70 mg, 0.45 μmol) was dissolved in 5 vol% formic acid in MeCN (453 μL). The solution was then used to solvate PTA (0.29 mg, 1.8 μmol, 4 eq.) and the resulting reaction mixture was irradiated with 450 nm light for 16 min. Aliquots of the reaction mixture were taken at t = 0 min and t = 16 min and diluted 5-fold into H_2O (0.1 vol% formic acid). Reaction analysis was performed on these aliquots using UPLC-MS on a gradient of 0-20% B over 5 min (λ = 214 nm).

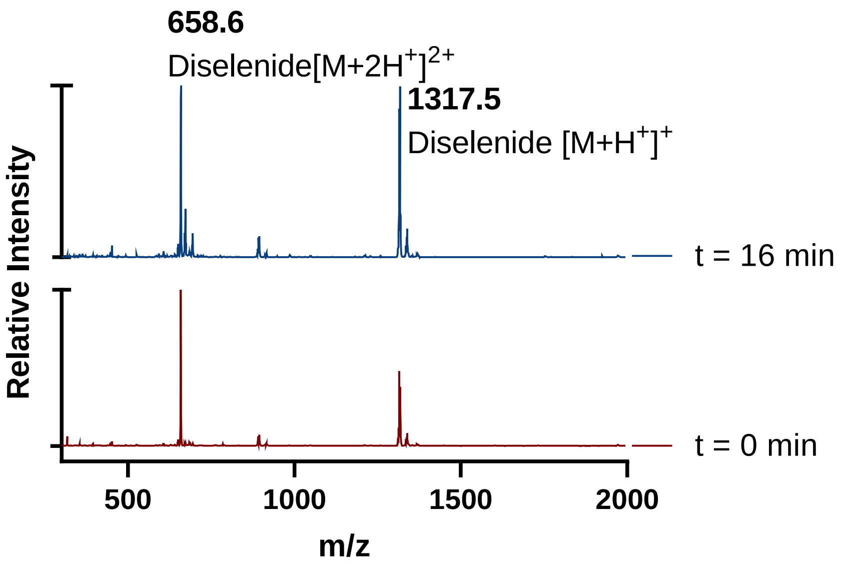

Supplementary Figure 15. Overlaid associated mass spectra of the 0 min (red) and 16 min (blue) time points taken from the negative control dimerization of a model peptide diselenide $[H_2N\text{-USPGYS-}NH_2]_2$ (**1**) employing only the phosphine PTA (4 eq.) in 5 vol% formic acid in MeCN.

‘No Light’ Control PDC Dimerization Reaction

$[H_2N\text{-USPGYS-}NH_2]_2$ diselenide **1** (0.70 mg, 0.45 μmol) was dissolved in 5 vol% formic acid in MeCN (46 μL) containing $[Ir(dF(CF_3)ppy)_2(dtbpy)]PF_6$ (**5**) (0.1 μmol mL^{-1}). The solution was then further diluted with 5 vol% formic acid in MeCN (180 μL). The solution was then used to solvate PTA (0.29 mg, 1.8 μmol, 4 eq.) and the resulting reaction mixture was left in the dark for 16 min. Aliquots of the reaction mixture were taken at t = 0 min and t = 16 min and diluted 5-fold into H_2O (0.1 vol% formic acid). Reaction analysis was performed on these aliquots using UPLC-MS on a gradient of 0-20% B over 5 min (λ = 214 nm).

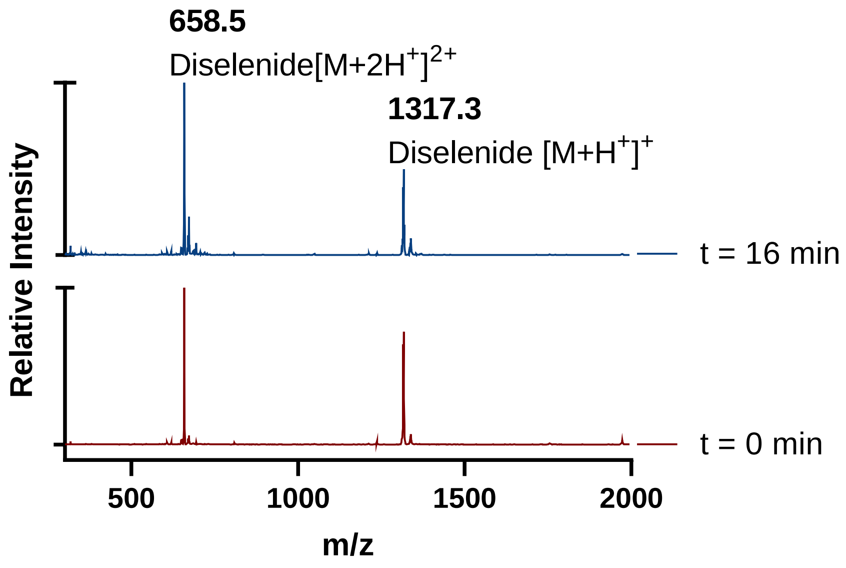

Supplementary Figure 16. Overlaid associated mass spectra of the 0 min (red) and 16 min (blue) time points taken from the negative control dimerization of a model peptide diselenide $[H_2N\text{-USPGYS-}NH_2]_2$ (**1**) employing $[Ir(dF(CF_3)ppy)_2(dtbpy)]PF_6$ (**5**) (1 mol%) and PTA (4 eq.) without 450 nm irradiation in 5 vol% formic acid in MeCN.

Mass Spectrometric and NMR Analysis of Model Selenoether Dimers

High-Resolution Mass Spectrometric Analysis of Selenoether Dimer 3

[H_2N-USPGYS-NH_2]$_2$ diselenide (**1**) (3.09 mg, 2.00 μmol) was dissolved in 200 μL of 1:1 v/v H_2O:MeCN containing [Ir(dF(CF_3)ppy)$_2$(dtbpy)]PF_6 (**5**) (0.1 μmol mL^{-1}) for a photocatalyst loading of 1 mol%. The solution was further diluted with 200 μL of 1:1 v/v H_2O:MeCN to bring the peptide concentration to 5 mM. This solution was then used to dissolve PTA (**4**) (1.28 mg, 8.00 μmol, 4 eq.) and the resulting reaction mixture was irradiated with 450 nm light for 1 min. After irradiation, UPLC-MS analysis showed complete conversion of the diselenide **1** to selenoether **3**, with minimal deselenization by-product **2** (*Supplementary Figure 17*). The reaction mixture was then diluted to ~ 5 mL with H_2O (0.1 vol% TFA) and purified by RP-HPLC (0-20% B over 45 min at 4 mL min^{-1}, Waters Sunfire C18 5 μm, 10 x 250 mm) to afford purified selenoether **3** (2.24 mg, 1.53 μmol) in 76% isolated yield (*Supplementary Figure 18*), with concurrent isolation of deselenised by-product H_2N-ASPGYS-NH_2 (**2**) (0.23 mg, 0.33 μmol, 17%).

This purified selenoether **3** was then analyzed by HRMS on a solariX XR mass spectrometer, injected in 1:1 v/v H_2O:MeCN. This analysis showed an exact match of the purified selenoether **3** to its calculated mass pattern within 0.1 ppm, also displaying the characteristic isotope pattern for Se (found: 619.22307 *m/z* $[M+2H^+]^{2+}$, calculated: 619.22300 *m/z* $[M+2H^+]^{2+}$) (*Supplementary Figures 19, 20*).

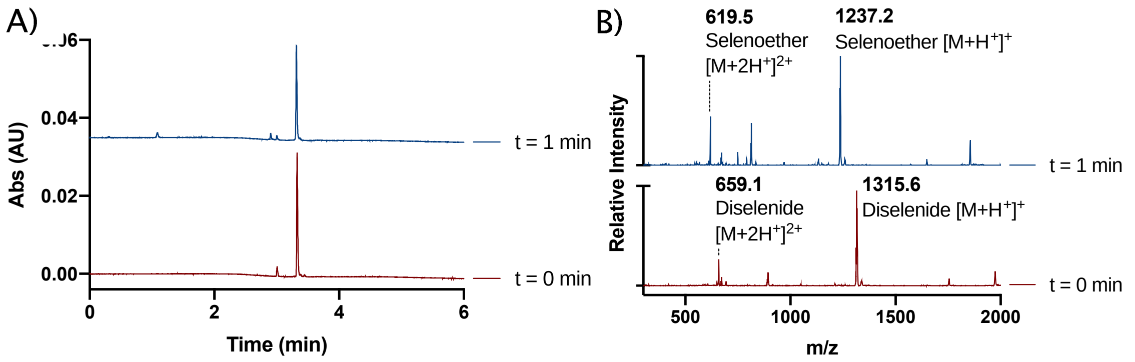

Supplementary Figure 17. A) Overlaid start (0 min, red) and end (1 min, blue) point UPLC analysis (0-20%B over 5 min) of the photocatalytic diselenide contraction of [H_2N-USPGYS-NH_2]$_2$ diselenide (**1**) with PTA (**4**) (4 eq.) and [Ir(dF(CF_3)ppy)$_2$(dtbpy)]PF_6 (**5**) (1 mol%) under 450 nm irradiation. **B)** Corresponding overlaid start (0 min, red) and end (1 min, blue) point MS (ESI+) analysis.

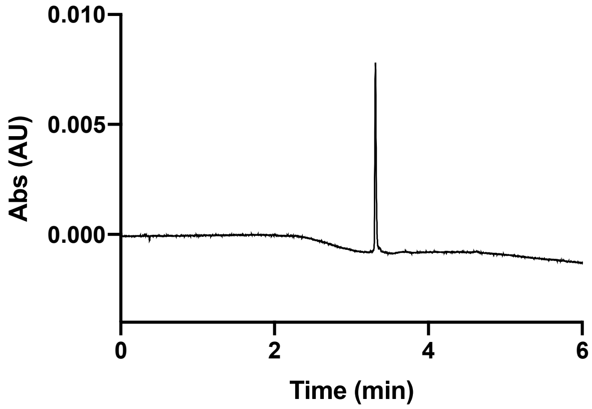

Supplementary Figure 18. UPLC analysis (0-20%B over 5 min at 0.6 mL min^{-1}, Waters Acquity BEH300 1.7 μm, 2.1 x 50 mm (C18), λ = 214 nm) of purified selenoether **3**.

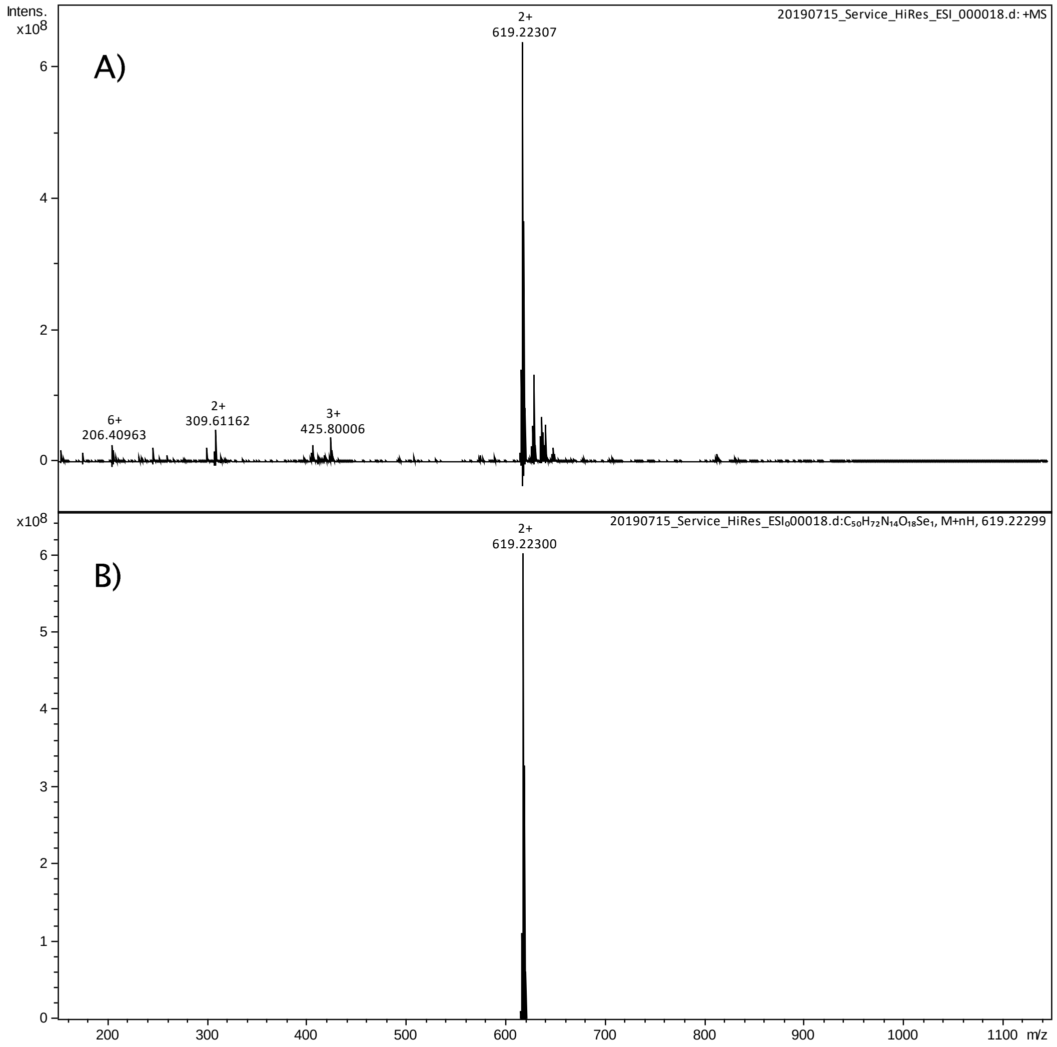

Supplementary Figure 19. A) Observed and **B)** calculated HRMS (ESI+) spectra of purified selenoether **3** as performed on a solariX XR FTICR mass spectrometer and injected in 1:1 v/v H_2O:MeCN (found: 619.22307 *m/z* $[M+2H^+]^{2+}$, calculated: 619.22300 *m/z* $[M+2H^+]^{2+}$, 0.1 ppm).

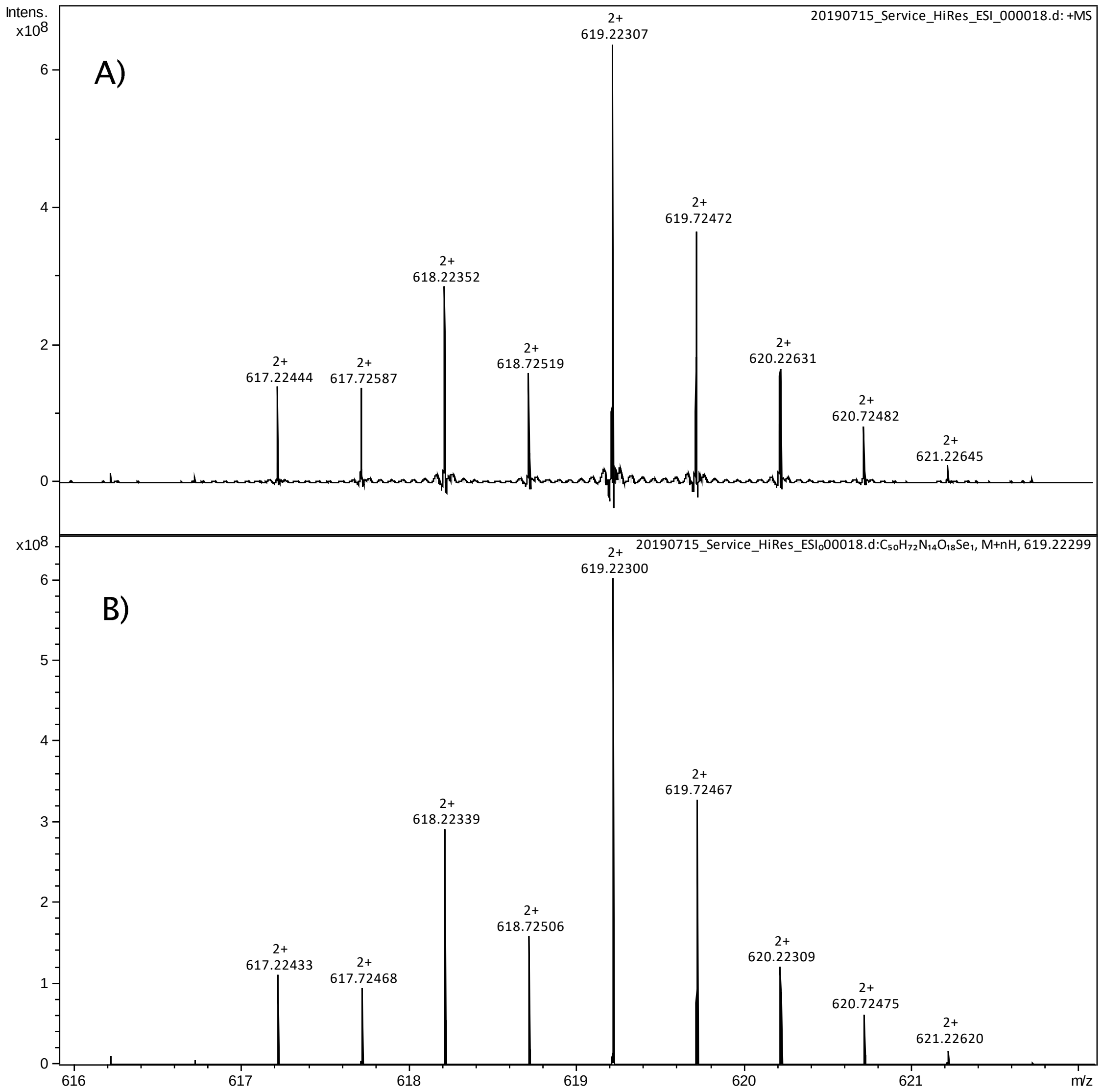

Supplementary Figure 20. Isotope pattern of **A)** observed and **B)** calculated HRMS (ESI+) spectra of purified selenoether **3** as performed on a solariX XR FTICR mass spectrometer and injected in 1:1 v/v H_2O:MeCN (found: 619.22307 *m/z* $[M+2H^+]^{2+}$, calculated: 619.22300 *m/z* $[M+2H^+]^{2+}$, 0.1 ppm).

Synthesis of [Boc-Sec-Phe-OMe]$_2$ diselenide

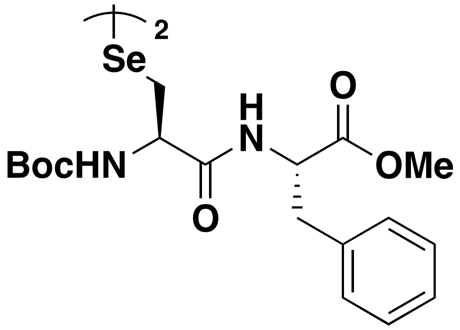

[Boc-Sec-Phe-OMe]$_2$ diselenide

[Boc-Sec-OH]$_2$ diselenide (535 mg, 1.00 mmol) and HOAt (544 mg, 4.00 mmol, 2 eq.) were dissolved in DMF (2 mL) and to this solution was added DIC (626 μL, 4.00 mmol, 2 eq.) and the resulting pre-activation mixture was stirred at room temperature for 3 min. A solution of H_2N-Phe-OMe (647 mg, 3.00 μmol, 1.5 eq.) in DMF (3 mL) was then added to the pre-activated mixture and the resulting coupling mixture was left to stir at room temperature for 16 h. The mixture was then poured over aqueous NH_4Cl and extracted with EtOAc (x 3). The organic layers were combined and washed with water (x 5) and saturated aqueous NaCl (x 1), then dried over Na_2SO_4, filtered and concentrated *in vacuo* to afford pure [Boc-Sec-Phe-OMe]$_2$ diselenide (800 mg, 0.934 mmol, 93%).

^{1}H NMR: (400 MHz, $CDCl_3$) δ 7.75 – 7.60 (m, 1H), 7.33 – 7.10 (m, 5H), 5.52 (d, *J* = 9.3 Hz, 1H), 4.87 (td, *J* = 8.2, 6.1 Hz, 1H), 4.75 – 4.65 (m, 1H), 3.69 (s, 3H), 3.32 – 3.16 (m, 3H), 3.06 (dd, *J* = 13.9, 8.1 Hz, 1H), 1.46 (s, 9H). **^{77}Se NMR:** (76 MHz, $CDCl_3$) δ 328.30. **LRMS (ESI+):** *m/z* 851.5 $[M+H^+]^+$.

Synthesis of a Boc-Sec-Phe-OMe Selenoether by PDC Dimerization

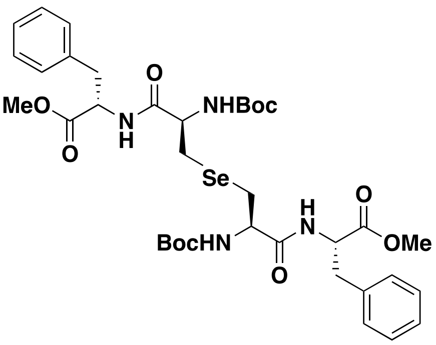

$[Boc\text{-}Sec\text{-}Phe\text{-}OMe]_2$ diselenide (50 mg, 58 µmol) was dissolved in MeCN (2 mL) and this solution was used to solvate $[Ir(dF(CF_3)ppy)_2(dtbpy)]PF_6$ (**5**) (0.65 mg, 0.58 µmol, 1 mol%) and PPh_3 (46 mg, 175 umol, 3 eq.). The combined reaction mixture was irradiated with 450 nm light for 15 min then concentrated under a stream of N_2 gas. The crude solid was re-dissolved in 10 vol% EtOAc in hexane and purified by flash chromatography (10-50 vol% EtOAc in hexane) to afford pure Boc-Sec-Phe-OMe selenoether (30 mg, 39 µmol, 67%).

^{1}H NMR: (400 MHz, $CDCl_3$) δ 7.58 – 7.44 (m, 1H), 7.36 – 7.17 (m, 5H), 5.67 (d, J = 8.1 Hz, 1H), 4.84 (td, J = 8.0, 6.0 Hz, 1H), 4.62 (s, 1H), 3.69 (s, 3H), 3.25 – 3.15 (m, 1H), 3.07 (dd, J = 13.9, 7.8 Hz, 1H), 2.90 (m, 2H), 1.46 (s, 9H). **^{13}C NMR:** (101 MHz, $CDCl_3$) δ 171.63, 170.42, 155.68, 129.14, 128.58, 127.02, 80.13, 54.61, 53.65, 52.28, 37.84, 28.36, 28.23. **^{77}Se NMR:** (76 MHz, $CDCl_3$) δ 140.10. **LRMS (ESI+):** *m/z* 779.2 $[M+H^+]^+$.

NMR Characterization of Boc-Sec-Phe-OMe Selenoether Dimer

Purified Boc-Sec-Phe-OMe selenoether was analyzed by ^{1}H, ^{13}C, ^{77}Se, ^{1}H-^{1}H COSY and ^{1}H-^{13}C HSQC NMR spectroscopy in $CDCl_3$ at frequencies of 400 MHz (^{1}H), 100 MHz (^{13}C) and 76 MHz (^{77}Se). COSY and HSQC spectra were used to assign ^{1}H peaks (*Supplementary Figure 21*). ^{1}H and ^{77}Se spectra were overlaid to show peak shifts upon diselenide contraction (*Supplementary Figures 22, 23*).

See next pages for experimental data.

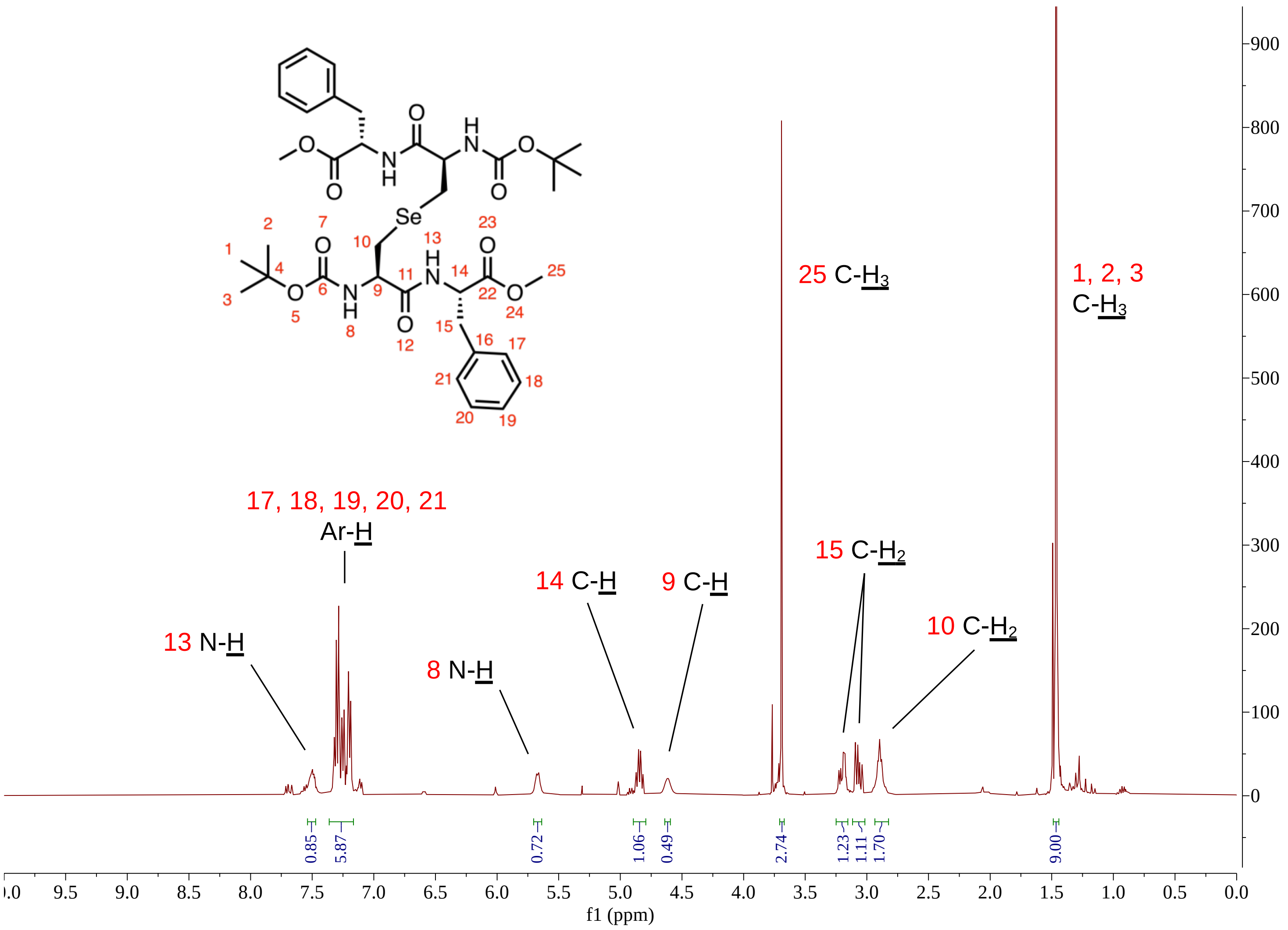

Supplementary Figure 21. ^{1}H NMR spectrum (400 MHz, $CDCl_3$) of Boc-Sec-Phe-OMe selenoether with signals assigned.

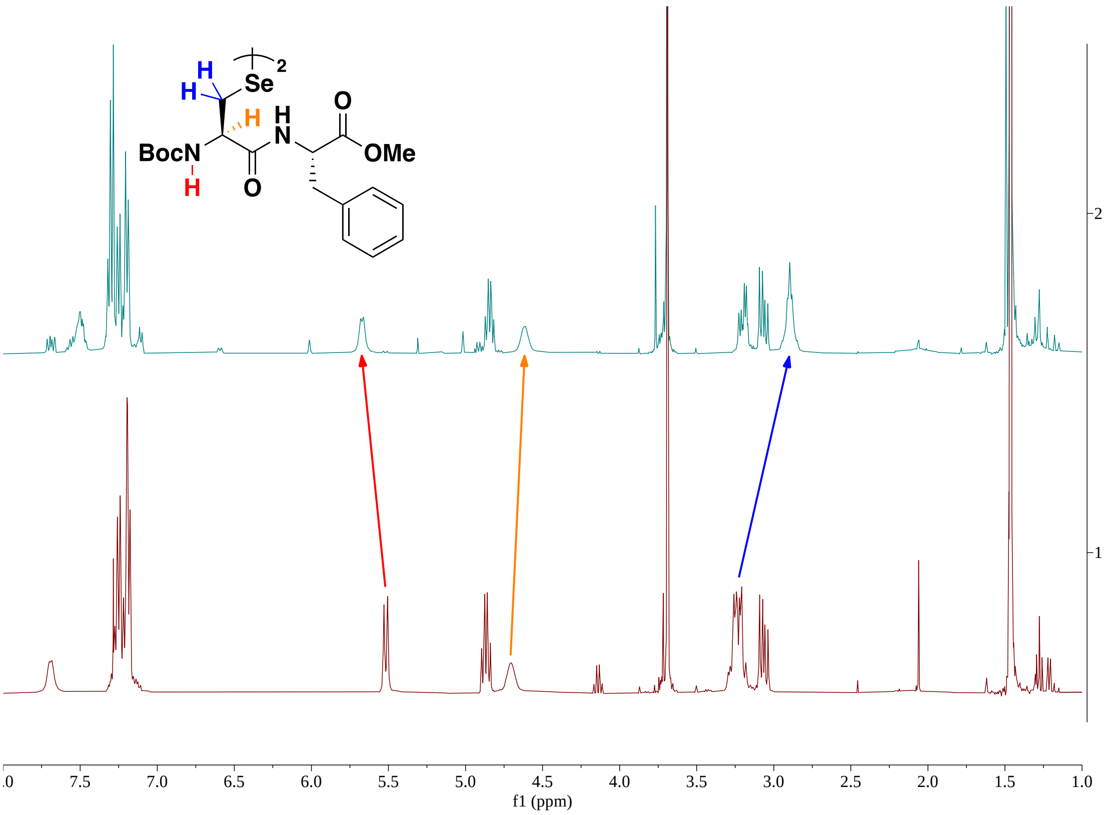

Supplementary Figure 22. Overlaid ^{1}H NMR spectra (400 MHz, $CDCl_3$) of **1)** Boc-Sec-Phe-OMe diselenide and **2)** selenoether displaying key Sec peak shifts: α-N<u>H</u> (red), α-C<u>H</u> (orange) and β-C<u>H</u>$_2$ (blue).

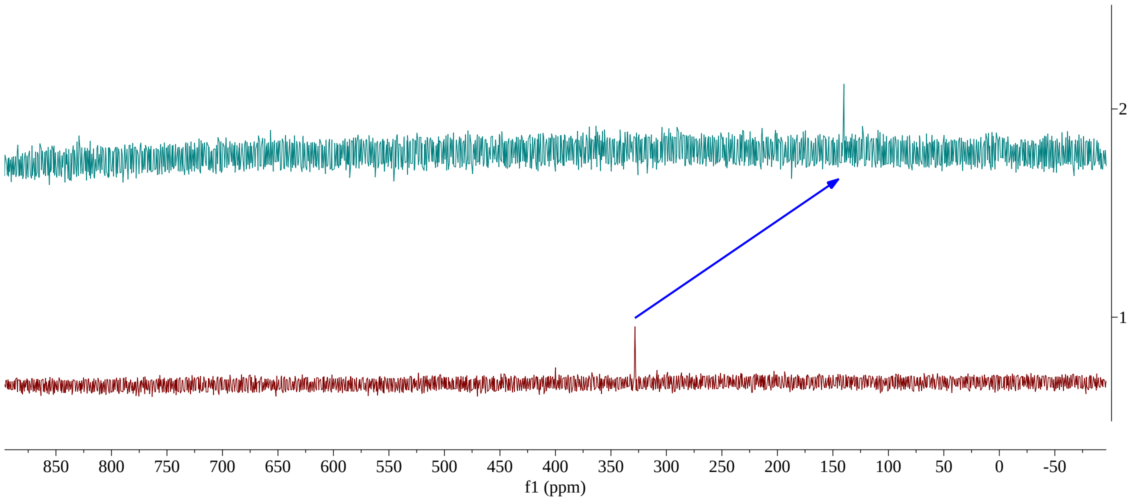

Supplementary Figure 23. Overlaid ^{77}Se NMR spectra (76 MHz, $CDCl_3$) of **1)** $[\text{Boc-Sec-Phe-OMe}]_2$ diselenide and **2)** selenoether displaying a key shift in the ^{77}Se peak (blue).

Stability Studies on Model Selenoether Dimer

Stability Studies on Selenoether Dimer 3 under Acidic, Basic, and Reducing Conditions

Purified selenoether dimer (**3**) (0.50 mg, 0.32 μmol) was dissolved in 40 μL of Milli-Q water to generate a 10 mM stock solution. 4 μL aliquots of this solution were then taken and diluted to 120 μL with PBS buffer (10 mM Na_2HPO_4, 137 mM NaCl, 2.7 mM KCl, 1.8 mM KH_2PO_4), 5 mM dithiothreitol (DTT) in PBS buffer, or 5 mM reduced glutathione (GSH) in PBS buffer. The pH of each solution was adjusted to give the following series of conditions:

1. PBS buffer, pH 2
2. PBS buffer, pH 4
3. PBS buffer, pH 10
4. PBS buffer, pH 12
5. 5 mM DTT in PBS buffer, pH 7.3
6. 5 mM GSH in PBS buffer, pH 7.3

These solutions were incubated at room temperature for 12 h with time points taken after 0, 6, and 12 h. At each time point, a 25 μL aliquot was taken and analysed using UPLC-MS on a gradient of 0-20% B over 5 min and analytical HPLC using a gradient of 1-20% B over 30 min (λ = 214 nm).

See next pages for analytical data.

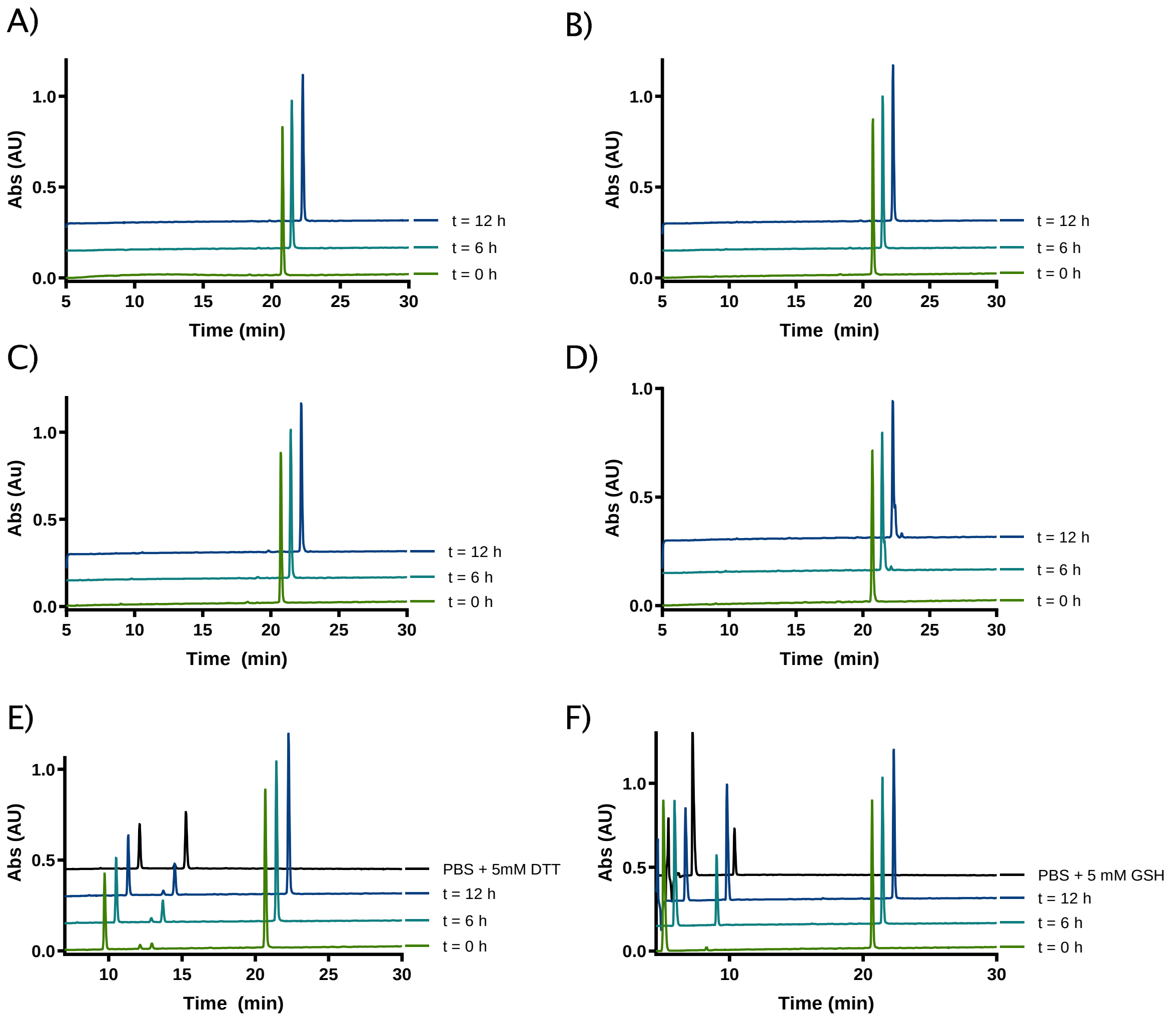

Supplementary Figure 24. Analytical HPLC analysis (1-20% B over 30 min, λ = 214 nm) of the stability of selenoether dimer (**3**) under the following conditions: **A)** PBS buffer, pH 2, **B)** PBS buffer, pH 4, **C)** PBS buffer, pH 10, **D)** PBS buffer, pH 12, **E)** 5 mM DTT in PBS buffer, pH 7.3, and **F)** 5 mM GSH in PBS buffer, pH 7.3.

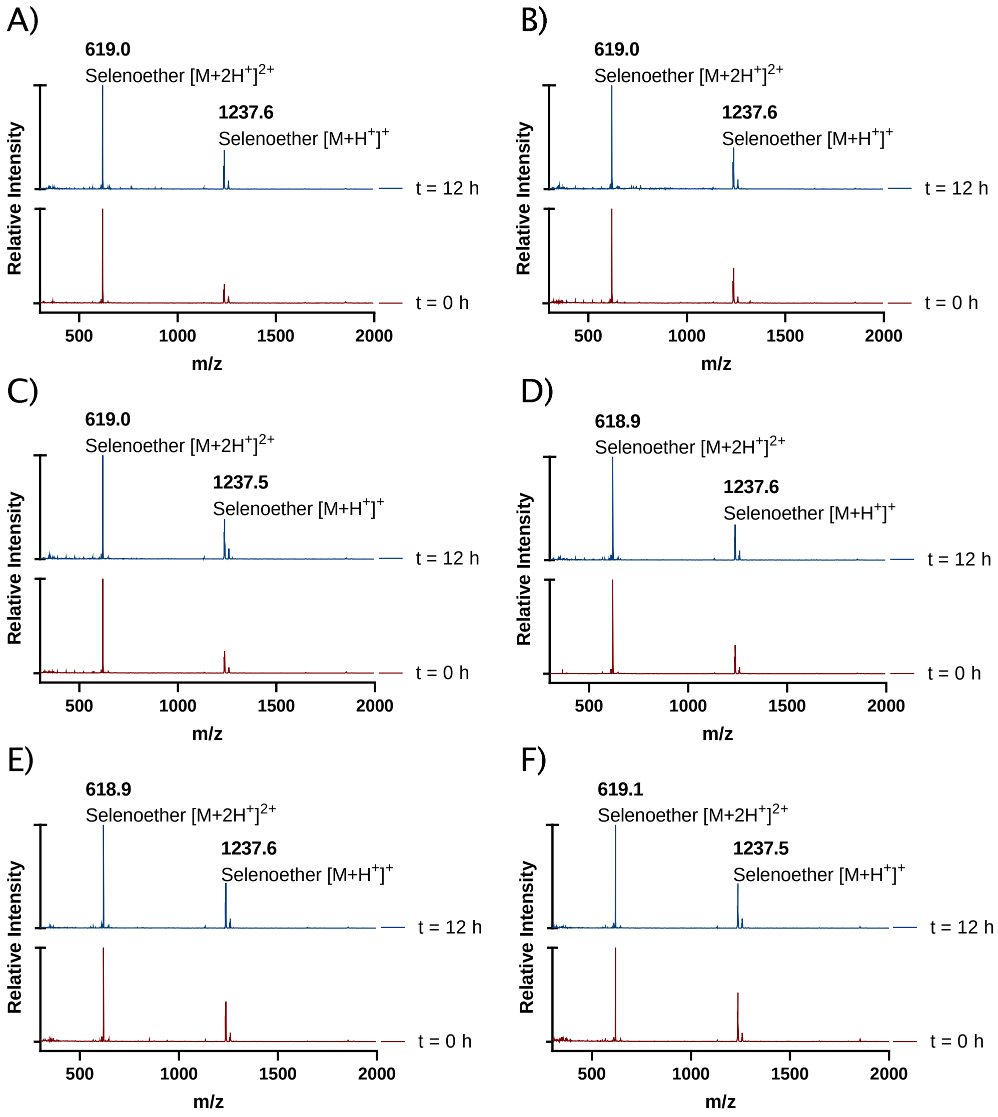

Supplementary Figure 25. Overlaid associated mass spectra of the 0 h (red) and 12 h (blue) time points taken from stability studies on selenoether dimer (**3**) under the following conditions: **A)** PBS buffer, pH 2, **B)** PBS buffer, pH 4, **C)** PBS buffer, pH 10, **D)** PBS buffer, pH 12, **E)** 5 mM DTT in PBS buffer, pH 7.3, and **F)** 5 mM GSH in PBS buffer, pH 7.3.

Stability Studies on Selenoether Dimer 3 in Human Plasma

The plasma stability of selenoether dimer (**3**) was determined using a method previously described by Ford *et al*.[3]

Selenoether dimer (**3**) (10 mM stock in MilliQ H_2O) was added to 200 µL of fresh human plasma to a final concentration of 200 µM and the resulting sample was incubated at 37 °C for 1, 4, or 24 hours. For each time point, 50 µL aliquots were removed and the plasma proteins were precipitated by the addition of 150 µL of 1:1 v/v MeOH:MeCN. After subjecting the samples to centrifugation at 19360 g for 15 minutes, the supernatants (20 µL) were analysed by UPLC-MS on a gradient of 0-40% B over 5 min.

Note: All procedures involving the collection of blood from healthy donors were approved by the University of Sydney Human Research Ethics Committee (HREC, Project 2014/244).

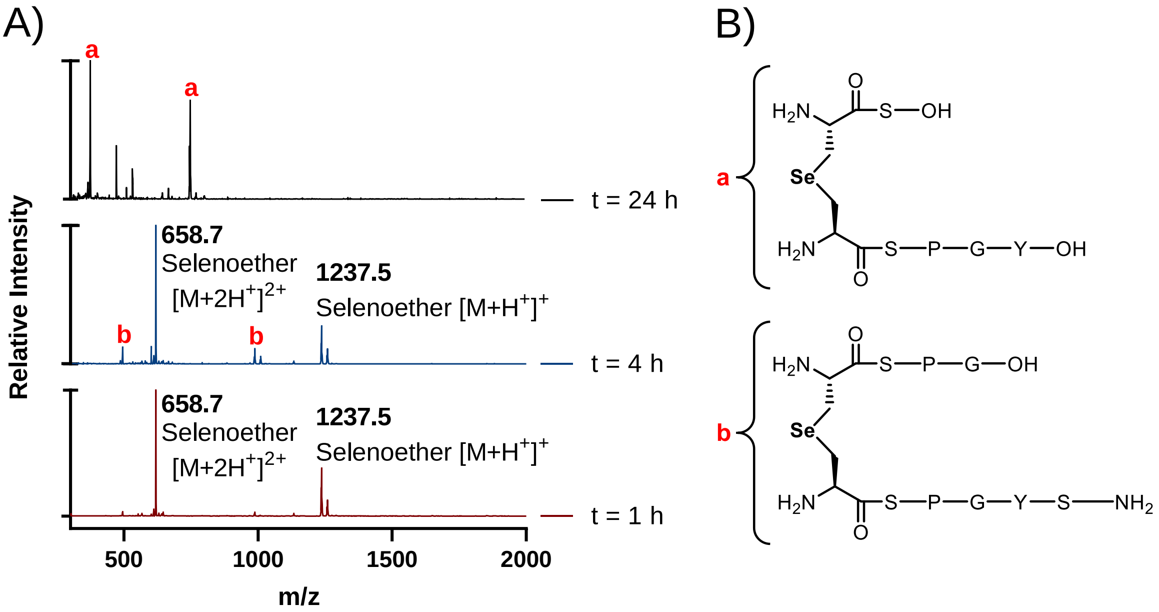

Supplementary Figure 26. Stability studies on selenoether dimer (**3**) in human plasma. **A)** Mass spectra of the 1 h, 4 h, and 24 h time points and **B)** structures of the major cleavage products, **a**: *m/z* 747.3 $[M+H^+]^+$, *m/z* 374.1 $[M+2H^+]^{2+}$, **b**: *m/z* 988.4 $[M+H^+]^+$, *m/z* 494.7 $[M+2H^+]^{2+}$.

Synthesis of diselenide dimer peptides 9-11

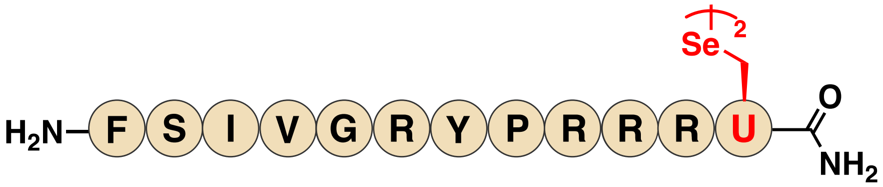

Gephyrin Binding Peptide 3a Diselenide (9)

Fmoc-Sec(Bn)-OH (1.5 eq.) was loaded to Rink amide resin (102 mg, 0.49 mmol g^{-1} loading) with DIC (1.5 eq.) and HOAt (3 eq.) at room temperature over 16 h for a loading of 50 μmol. The loaded resin was then transferred to a Biotage Syro I automated peptide synthesiser for automated coupling of the F1-R11 fragment with single 50 °C couplings (*see General Procedures)* with a final Fmoc-deprotection. The peptide was cleaved from resin with an acidic cleavage cocktail (3 mL, 90:5:5 v/v/v TFA:iPr$_3$SiH:H_2O) at room temperature over 2 h, then transferred to an empty 50 mL centrifuge tube, concentrated under a gentle stream of N_2 gas and precipitated by addition of cold Et_2O (40 mL). The resulting precipitate was pelleted by centrifugation (4000 x g, 5 min, 4 °C), the ether supernatant decanted and the remaining peptide pellet allowed to dry under a gentle stream of N_2 gas. The crude peptide was then re-dissolved in 2 vol% thioanisole in TFA (13.2 mL) to an estimated peptide concentration of 3.8 mM (based on resin loading). To this solution was added 2,2-dithiobis(5-nitropyridine) (DTNP) (310 mg, 1.00 mmol, 20 eq.) and the resulting reaction mixture was incubated at 50 °C for 1 h, then cooled to room temperature and incubated for a further 1 h. The mixture was then concentrated under a gentle stream of N_2 then precipitated in cold Et_2O (3 x 40 mL) as above. Pelleting of this peptide precipitate by centrifugation (4000 x g, 5 min, 4 °C) allowed the crude peptide to be dried under a stream of N_2. The crude peptide pellet was then re-dissolved in 20 vol% MeCN in H_2O (0.1% TFA) (5 mL) for purification. Immediately before purification, a spatula tip of sodium ascorbate followed by TCEP was added to the solution to effect reduction of the asymmetric DTNP-selenylsulfide. RP-HPLC purification (0-40% B over 30 min) then afforded peptide diselenide **9** (15.9 mg, 1.17 μmol, 2% yield from 50 μmol resin loading) as a white solid following lyophilization.

See next page for analytical data.

A)

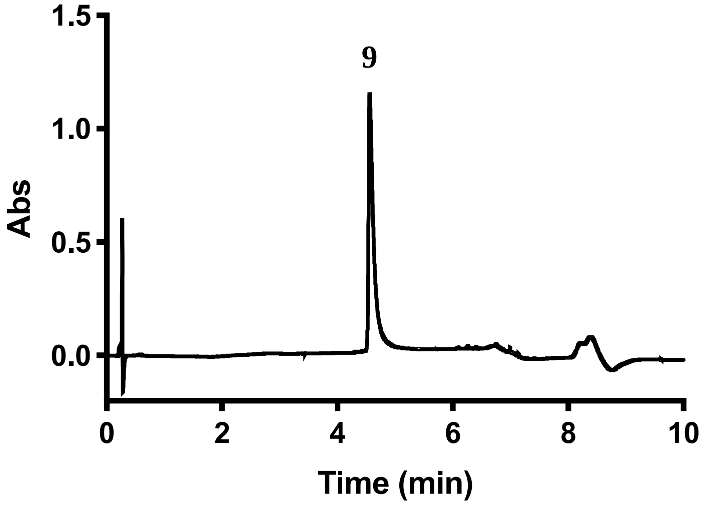

B)

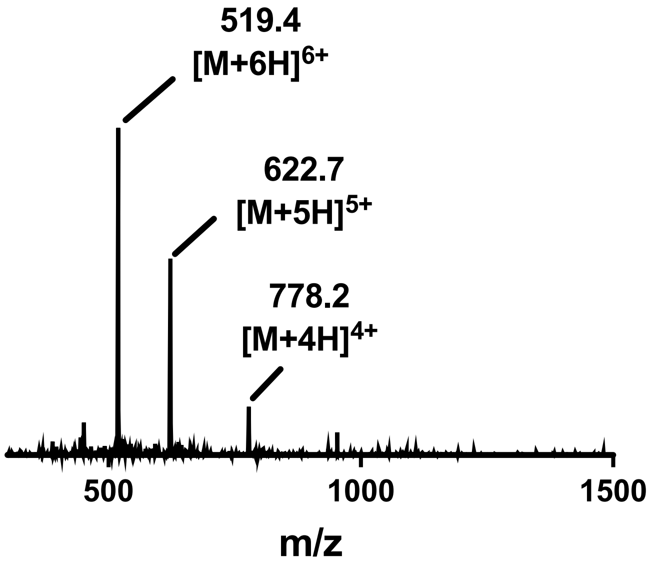

Supplementary Figure 27. A) UPLC-MS analysis (15-35% B over 5 min at 0.6 mL min^{-1}, Waters Acquity BEH300 1.7 µm, 2.1 x 50 mm (C18), λ = 214 nm) of purified Gephyrin Binding Peptide 3a diselenide (**9**). **B)** MS (ESI+) spectrum of purified Gephyrin Binding Peptide 3a diselenide (**9**).

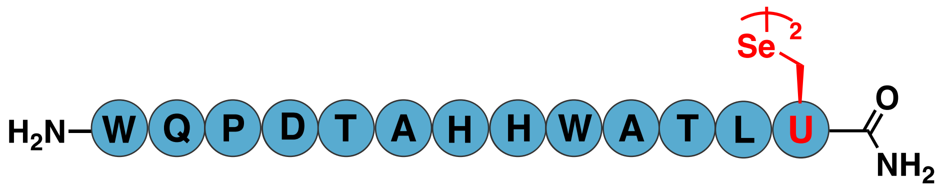

PSMA Binding Peptide Diselenide (10)

Fmoc-Sec(Bn)-OH (1.5 eq.) was loaded to Rink amide resin (102 mg, 0.49 mmol g^{-1} loading) with DIC (1.5 eq.) and HOAt (3 eq.) at room temperature over 16 h for a loading of 50 μmol. The loaded resin was then transferred to a Biotage Syro I automated peptide synthesizer for automated synthesis of the W1-L12 fragment with single 50 °C couplings (*see General Procedures*) with a final Fmoc-deprotection. The peptide was cleaved from resin with an acidic cleavage cocktail (3 mL, 90:5:5 v/v/v TFA:$i$$Pr_3SiH$:$H_2O$) at room temperature over 2 h, then transferred to an empty 50 mL centrifuge tube, concentrated under a gentle stream of N_2 and precipitated by addition of cold Et_2O (40 mL). The resulting precipitate was pelleted by centrifugation (4000 x g, 5 min, 4 °C), the ether supernatant decanted and the remaining peptide pellet allowed to dry under a gentle stream of N_2. The crude peptide was then re-dissolved in 2 vol% thioanisole in TFA (13.2 mL) to an estimated peptide concentration of 3.8 mM (based on resin loading). To this solution was added DTNP (310 mg, 1.00 mmol, 20 eq.) and the resulting reaction mixture was incubated at 50 °C for 1 h, then cooled to room temperature and incubated for a further 1 h. The mixture was then concentrated under a gentle stream of N_2 then precipitated in cold Et_2O (3 x 40 mL) as above. Pelleting of this peptide precipitate by centrifugation (4000 x g, 5 min, 4 °C) allowed the crude peptide to be dried under a stream of N_2. The crude peptide pellet was then re-dissolved in 20 vol% MeCN in H_2O (0.1% TFA) (5 mL) for purification. Immediately before purification, a spatula tip of sodium ascorbate followed by TCEP was added to the solution to effect reduction of the asymmetric DTNP-selenylsulfide. RP-HPLC purification (20-40% B over 45 min) then afforded peptide diselenide **10** (8.70 mg, 2.23 μmol, 5% isolated yield from 50 μmol resin loading) as a white solid following lyophilization.

See next page for analytical data.

A)

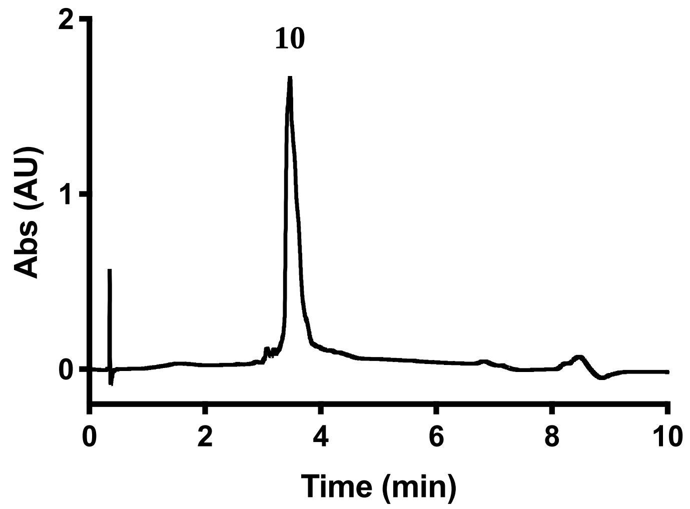

B)

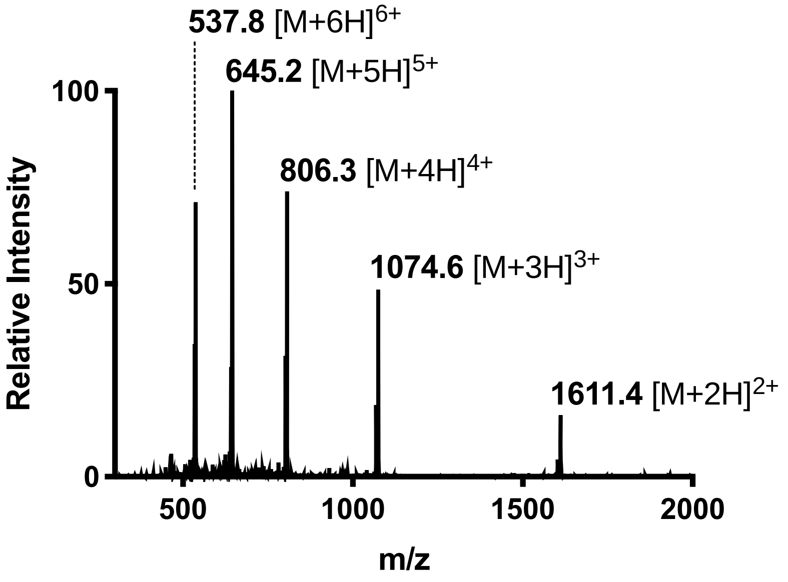

Supplementary Figure 28. A) UPLC-MS analysis (20-40% B over 5 min at 0.6 mL min^{-1}, Waters Acquity BEH300 1.7 µm, 2.1 x 50 mm (C18), λ = 214 nm) of purified PSMA binding peptide diselenide (**10**). **B)** MS (ESI+) spectrum of purified PSMA binding peptide diselenide (**10**).

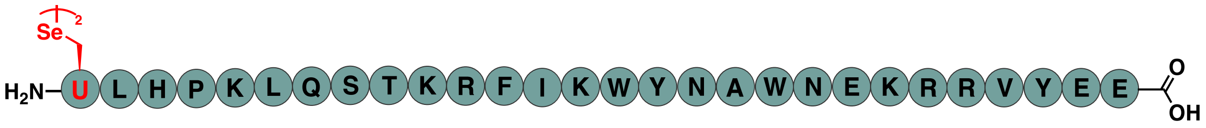

[CXCL14$_{51\text{-}77}$]$_2$ Diselenide (11)

Fmoc-Glu(*t*Bu)-OH (5 eq.) was loaded to 2-CTC resin (1.14 mmol g^{-1} loading) with *i*Pr_2NEt (10 eq.) in CH_2Cl_2 (*c* = 0.1 M) at room temperature for 16 h according to standard protocols (*see General Procedures*). The loaded resin was transferred to a Biotage Syro I automated peptide synthesizer for automated synthesis of the L2-E27 fragment (*see General Procedures*) with room temperature couplings and double coupling of residues L6-I13, Y16, N17, W19, N20, and K22-V25. After final Fmoc-deprotection of L2, coupling of [Boc-Sec-OH]$_2$ (0.5 eq.) was carried out using DIC (1 eq.) and HOAt (2 eq.) at room temperature over 16 h. The peptide was then cleaved from resin with an acidic cleavage cocktail (3 mL, 90:5:5 v/v/v TFA:*i*Pr_3SiH:H_2O) at room temperature over 2 h, then transferred to an empty 50 mL centrifuge tube, concentrated under a gentle stream of N_2 and precipitated by addition of cold Et_2O (40 mL). The resulting precipitate was pelleted by centrifugation (2000 x g, 5 min, 4 °C), the ether supernatant decanted and the remaining peptide pellet allowed to dry under a gentle stream of N_2. The crude peptide was then re-dissolved in 20 vol% MeCN in H_2O (0.1 vol% TFA) (10 mL) for purification by RP-HPLC (0-40% B over 40 min, Waters XBridge C18 30 x 150 mm) to afford pure [CXCL14$_{51\text{-}77}$]$_2$ diselenide (**11**) (14.5 mg, 1.58 µmol, 3% isolated yield from initial 103 µmol loading) as a white solid following lyophilization.

See next page for analytical data.

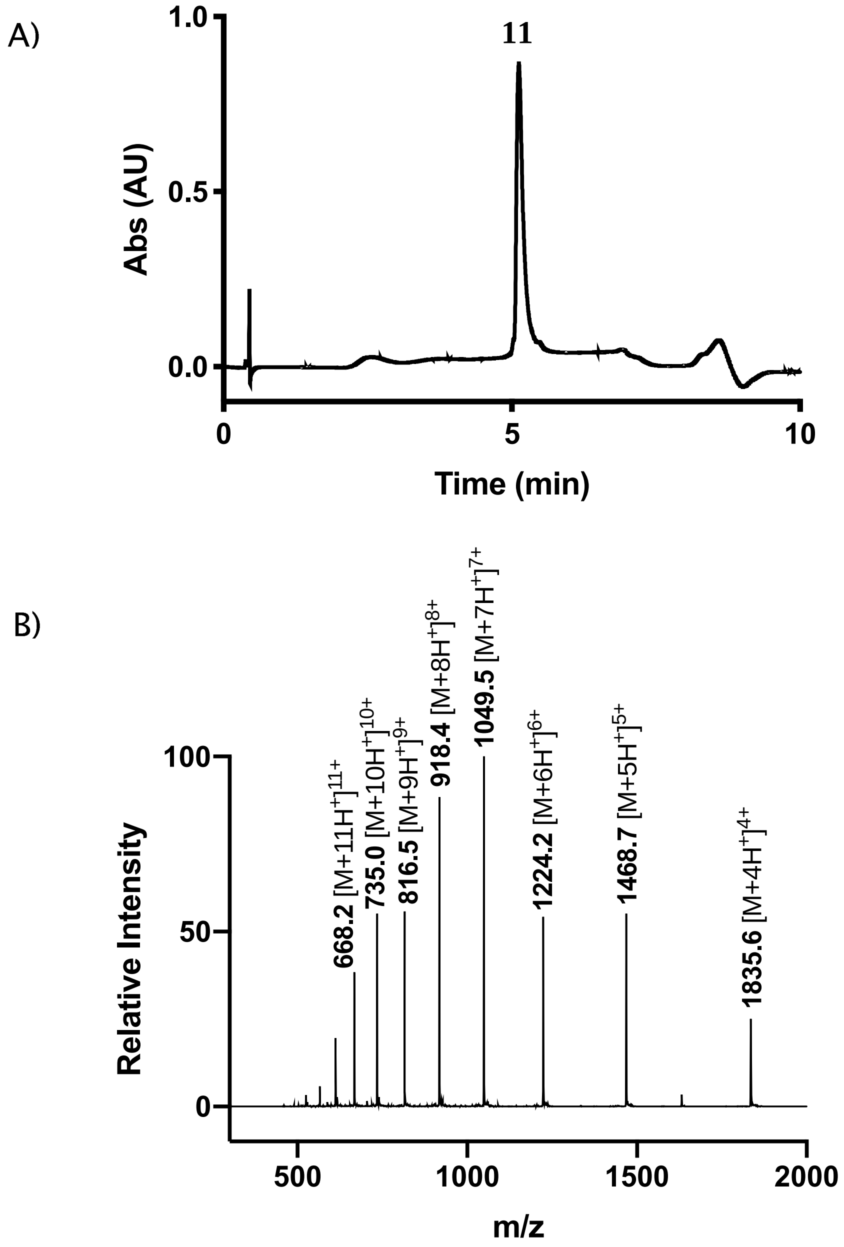

Supplementary Figure 29. A) UPLC analysis (0% B for 1 min then 0-40% B over 5 min at 0.6 mL min^{-1}, Waters Acquity BEH300 1.7 μm, 2.1 x 50 mm (C18), λ = 214 nm) of pure [CXCL14$_{51\text{-}77}$]$_2$ diselenide (**11**). **B)** MS (ESI+) spectrum of pure [CXCL14$_{51\text{-}77}$]$_2$ diselenide (**11**).

PDC Dimerization of Peptide Targets

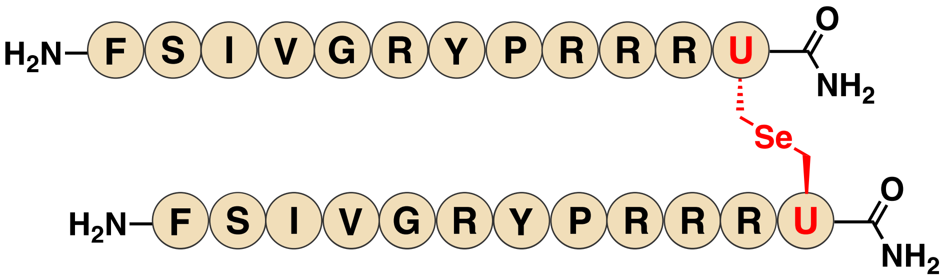

Gephyrin Binding Peptide 3a (GBP3a) Selenoether (6)

Gephyrin Binding Peptide 3a diselenide (**9**) (6.21 mg, 2.00 μmol) was dissolved in 0.8 mL of a solution of $[Ir(dF(CF_3)ppy)_2(dtbpy)]PF_6$ (**5**) (0.025 μmol mL^{-1}) in 1:1 v/v aqueous denaturing buffer (1 M Gn.HCl, 0.02 M Na_2HPO_4, pH 7.2):MeCN. The solution was used to dissolve PTA (**4**) (1.26 mg, 8.00 μmol) and the resulting reaction mixture was quickly vortexed, briefly centrifuged then irradiated with 450 nm light for 4 min. 10 μL aliquots were taken at 0, 2 and 4 min, diluted 5-fold in H_2O (0.1 vol% TFA), and analysed by UPLC-MS (15-35% B over 5 min, Waters Acquity BEH300 1.7 μm, 2.1 x 50 mm (C18), λ = 214 nm) and by UPLC (15-35% B over 5 min, Waters Acquity BEH300 1.7 μm, 2.1 x 50 mm (C18), λ = 214 nm). After completion, the crude reaction mixture was diluted to ~ 4 mL with H_2O (0.1 vol% TFA) and purified by RP-HPLC (15-35% B over 30 min) to afford the purified selenoether **6** (3.95 mg, 1.30 μmol, 65% isolated yield) as a white solid following lyophilization.

See next page for analytical data.

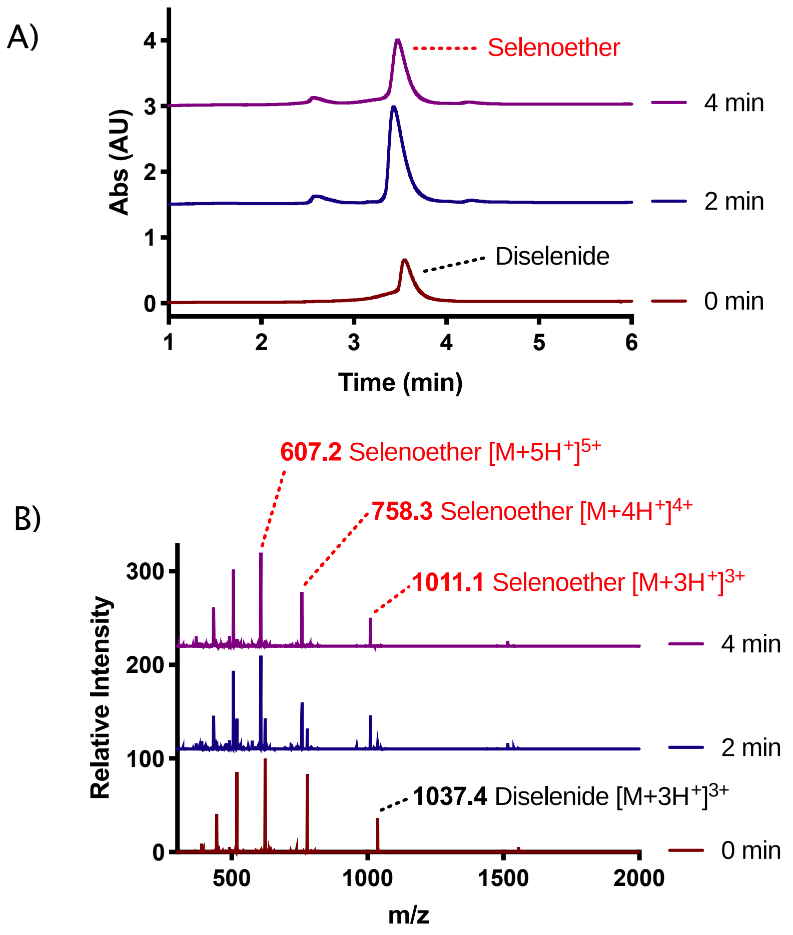

Supplementary Figure 30. A) Conversion of Gephyrin binding peptide 3a diselenide **9** to selenoether **6** by photocatalytic diselenide contraction as monitored by UPLC (15-35% B over 5 min at 0.6 mL min^{-1}, λ = 214 nm). **B)** Associated overlaid MS (ESI+) spectra of the conversion of diselenide **9** to selenoether **6**.

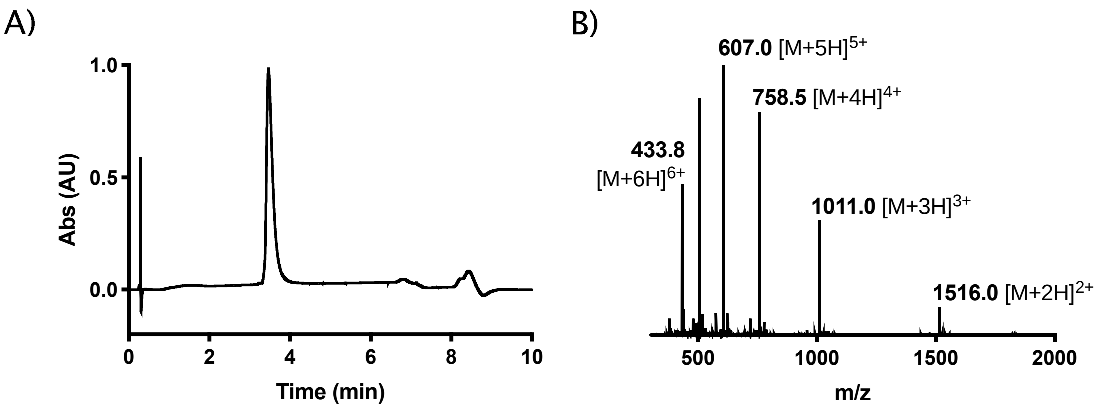

Supplementary Figure 31. A) UPLC (15-35% B over 5 min at 0.6 mL min^{-1}, λ = 214 nm) of purified Gephyrin Binding Peptide 3a selenoether **6**. **B)** Associated MS (ESI+) spectrum of purified selenoether **6**.

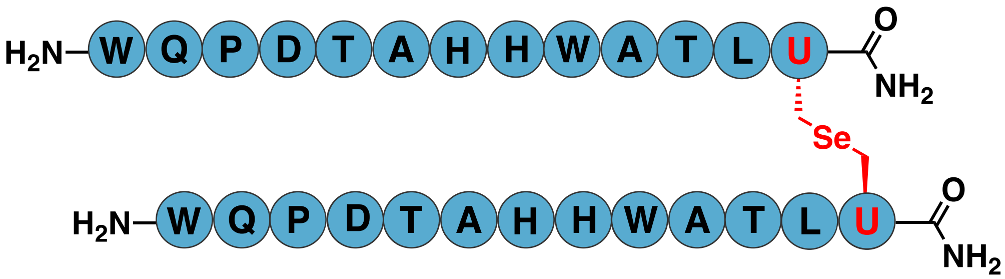

Prostate Specific Membrane Antigen Binding Peptide (PSMABP) Selenoether (7)

PSMA Binding Peptide diselenide (**10**) (4.68 mg, 1.20 µmol) was dissolved in 0.485 mL of solution of [Ir(dF(CF$_3$)ppy)$_2$(dtbpy)]PF$_6$ (**5**) (0.025 µmol mL^{-1}) in 1:1 v/v aqueous denaturing buffer (1 M Gn.HCl, 0.02 M Na$_2$HPO$_4$, pH 7.2):MeCN. The solution was used to dissolve PTA (**4**) (0.76 mg, 4.8 µmol) and the solution was quickly vortexed, briefly centrifuged then irradiated with 450 nm light for 6 min. 10 µL aliquots were taken at 0, 4 and 6 min, diluted 5-fold in H_2O (0.1 vol% TFA), and analysed by UPLC-MS (0-40% B over 5 min) and UPLC (20-40% B over 5 min). After completion, the crude reaction mixture was diluted to ~ 4 mL with H_2O (0.1 vol% TFA) and purified by RP-HPLC (20-40% B over 45 min) to afford purified selenoether **7** (2.35 mg, 0.61 µmol, 51% isolated yield) as a white solid following lyophilization.

See next page for analytical data.

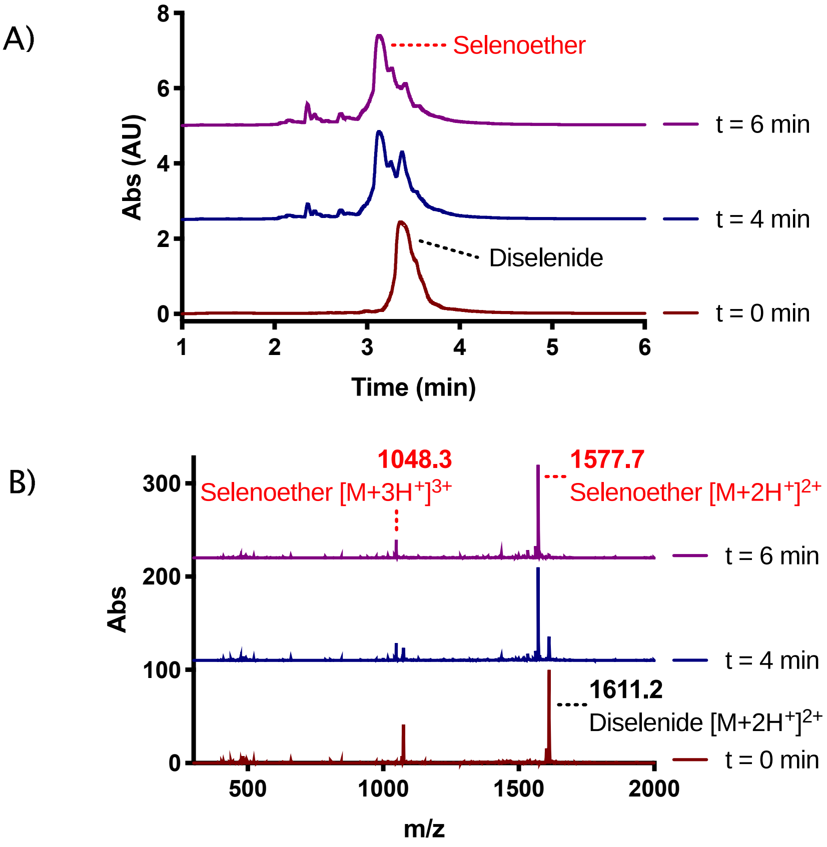

Supplementary Figure 32. A) Conversion of PSMA binding peptide diselenide (**10**) to selenoether **7** by photocatalytic diselenide contraction as monitored by UPLC (20-40% B over 5 min at 0.6 mL min^{-1}, Waters Acquity BEH300 1.7 μm, 2.1 x 50 mm (C18), λ = 214 nm). **B)** Associated overlaid MS (ESI+) spectra of the conversion of diselenide **10** to selenoether **7**.

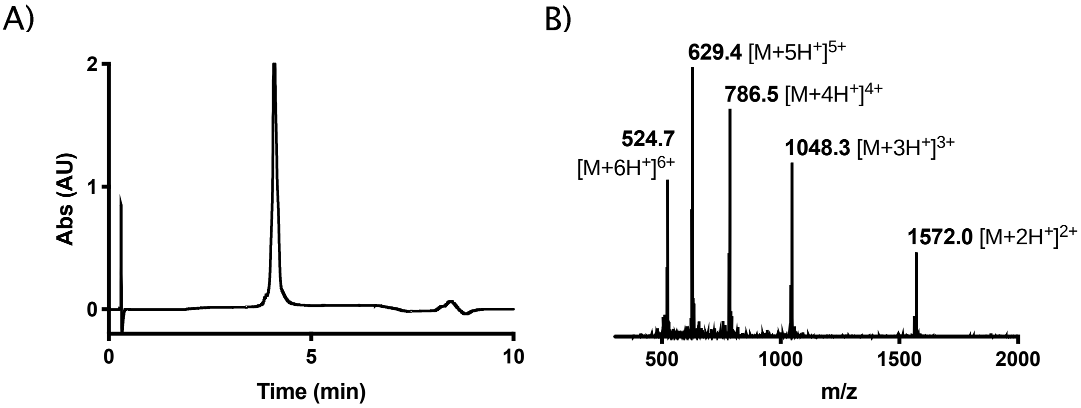

Supplementary Figure 33. A) UPLC (20-40% B over 5 min at 0.6 mL min^{-1}, Waters Acquity BEH300 1.7 μm, 2.1 x 50 mm (C18), λ = 214 nm) of purified PSMABP selenoether (**7**). **B)** Associated MS (ESI+) spectrum of purified selenoether **7**.

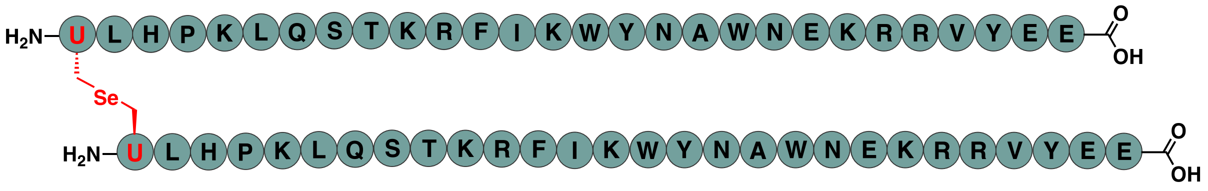

CXC Chemokine Ligand 14 (51-77) ($CXCL14_{51\text{-}77}$) Selenoether (8)

$[CXCL14_{51\text{-}77}]_2$ diselenide (**11**) (5.08 mg, 0.55 μmol) was dissolved in 55 μL of a solution of $[Ir(dF(CF_3)ppy)_2(dtbpy)]PF_6$ (**5**) (0.1 μmol mL^{-1}) and PTA (**4**) (6.29 mg mL^{-1}, 40 μmol mL^{-1}) in 1:1 v/v H_2O:MeCN for photocatalyst loading of 1 mol%, 4 eq. of PTA (**4**) (2.2 umol) and a concentration of **11** of 10 mM. The resulting reaction mixture was quickly vortexed, briefly centrifuged then irradiated with 450 nm light (PennOC M1 photoreactor) for 5 min. 1 μL aliquots were taken at 0 and 5 min and diluted 30-fold with H_2O (0.1 vol% TFA) for analysis by UPLC-MS (0-40% B over 5 min) and UPLC (0-40% B over 5 min). After completion of the reaction, the crude reaction mixture was diluted to ~ 4 mL with H_2O (0.1 vol% TFA) and purified by RP-HPLC (0-50% B over 40 min, Waters XBridge C18 10 x 250 mm) to afford the purified selenoether **8** (2.69 mg, 0.296 μmol) in 54% isolated yield as a white powder after lyophilization.

See next page for analytical data.

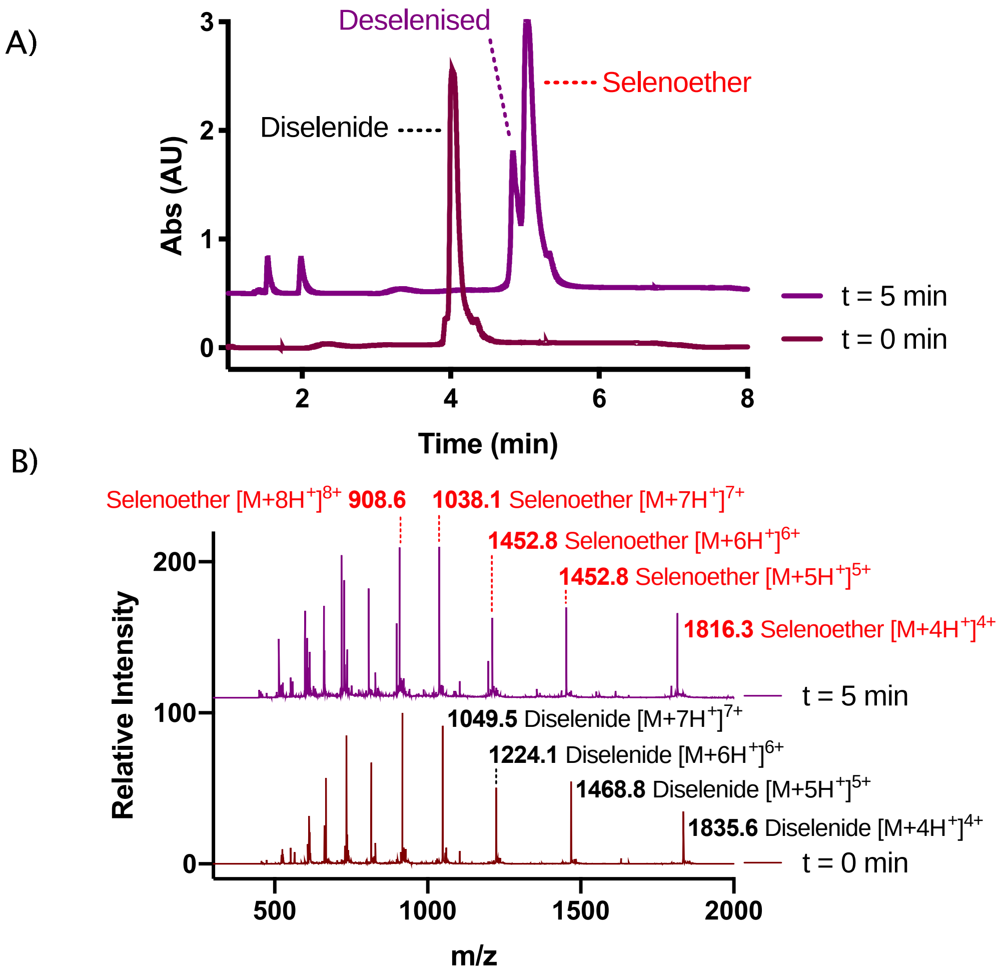

Supplementary Figure 34. A) Conversion of $[CXCL14_{51\text{-}77}]_2$ diselenide (**11**) to selenoether **8** by photocatalytic diselenide contraction as monitored by UPLC (0-40% B over 5 min at 0.6 mL min^{-1}, Waters Acquity BEH300 1.7 μm, 2.1 x 50 mm (C18), λ = 214 nm). **B)** Associated overlaid MS (ESI+) spectra for the conversion of $[CXCL14_{51\text{-}77}]_2$ diselenide (**11**) to selenoether **8**.

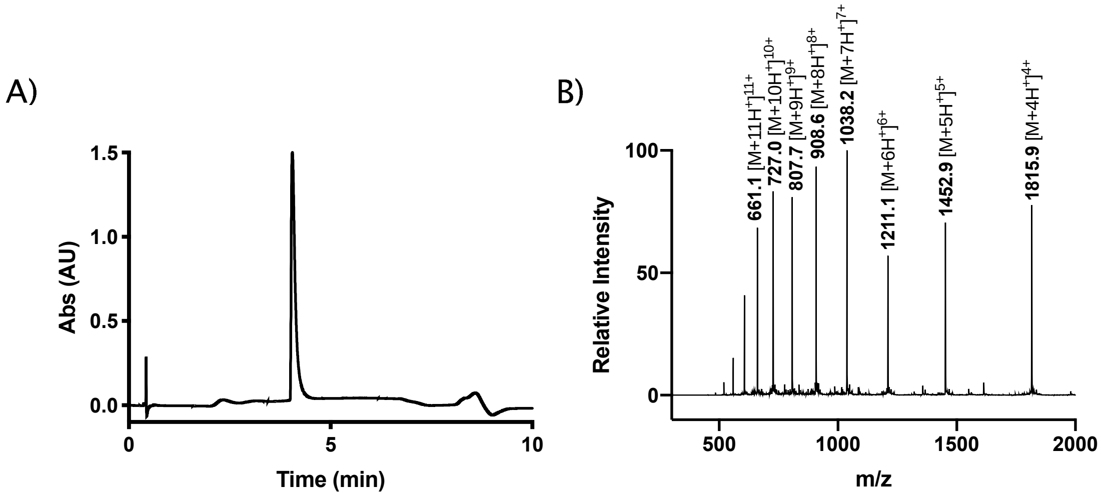

Supplementary Figure 35. A) UPLC (0-40% B over 5 min at 0.6 mL min^{-1}, Waters Acquity BEH300 1.7 μm, 2.1 x 50 mm (C18), λ = 214 nm) of purified selenoether (**8**). **B)** Associated MS (ESI+) spectrum of purified selenoether **8**.

Bacterial Expression of CaM (K148U) Diselenide 12

The DNA sequence encoding a His_6-MBP-TEV cassette and CaM (K148U) [with a TAG amber stop codon at position 148] were PCR amplified (Q5 polymerase, 25 cycles) to bear overhanging regions with each other and with the backbone of the pUC-PT7 plasmid, using the following primers: Fwd_His_6-MBP-TEV and Rvs_His_6-MBP-TEV (T_a = 65 ^oC), and Fwd_CaM and Rvs_CaM (T_a = 60 ^oC), respectively (*see Primer Oligonucleotide DNA Sequences*). The pUC-PT7 plasmid was amplified and linearized by PCR (Q5 polymerase, 25 cycles) to bear complementary overhangs with the 5' region of the His_6-MBP-TEV cassette and the 3' region of the CaM (K148U) cassette, using the primers: Fwd_pUC-PT7 and Rvs_pUC-PT7 (T_a = 60 ^oC) (*see Primer Oligonucleotide DNA Sequences*). Amplified DNA was purified using a Monarch Gel Purification Kit and the desired expression plasmid encoding His_6-MBP-TEV-CaM (K148U) (*Supplementary Figure 36*) was assembled from this pure DNA using a 3-fragment Gibson Assembly (New England Biolabs, MA, USA) reaction as per manufacturer's instructions. Positive transformants from Gibson Assembly were transformed into commercial NEB 5-α cells (50 μL) by heat shock at 42 ^oC for 30 s and selected on LB-agar plates supplemented with chloramphenicol (33 μg mL^{-1}). Successful transformants were used to inoculate 5 mL LB cultures [supplemented with chloramphenicol (33 μg mL^{-1})] to purify plasmid DNA using a Monarch Plasmid Miniprep Kit.

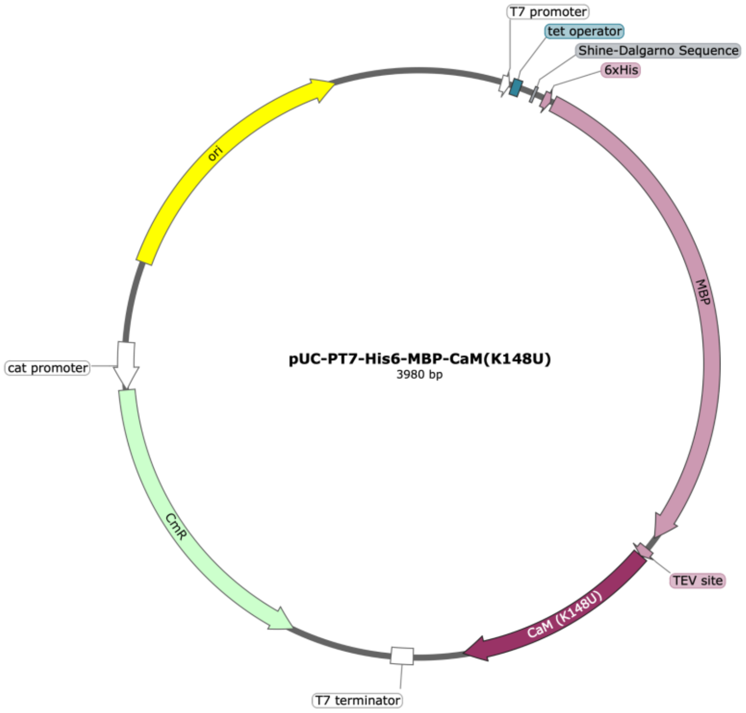

Supplementary Figure 36. Plasmid map of pUC-PT7-His_6-MBP-TEV-CaM (K148U).

A 1 μL aliquot of a pUC-PT7 plasmid encoding a His_6-MBP-TEV-tagged CaM (K148U) was transformed into a 50 μL aliquot of amber-suppressing electrocompetent *E. coli* β_UU3-T7 cells[4] through electroporation (2500 V, 25 μF, 200 Ω), after which cells were recovered for 1 h at 37 °C in SOC medium (950 μL) and grown on an LB-agar plate containing chloramphenicol (33 μg mL^{-1}), carbenicillin (500 μg mL^{-1}) and Na_2SeO_3 (10 μM).

A single colony from this transformation was used to inoculate LB medium (24 x 10 mL) containing chloramphenicol (33 μg mL^{-1}), carbenicillin (500 μg mL^{-1}) and Na_2SeO_3 (10 μM). This starter culture was grown to saturation at 37 °C over 16 h with orbital shaking (150 rpm). The starter cultures were then diluted 100-fold into TB medium (24 x 1 L) containing chloramphenicol (33 μg mL^{-1}), carbenicillin (500 μg mL^{-1}) and Na_2SeO_3 (25 μM), and the resulting expression cultures were grown in baffled flasks to mid-log phase at 37 °C for 4 h and with orbital shaking (150 rpm). Upon reaching mid-log phase, protein expression was induced with addition of anhydrotetracycline (200 ng μL^{-1}) and the resulting cultures were incubated at a temperature of 25 °C for 20 h. Cells were then harvested by centrifugation (8000 x g, 10 min, 4 °C) and re-suspended in 24 x 25 mL of lysis buffer (50 mM K_2HPO_4, 300 mM NaCl, 10 vol% glycerol, pH 8.0) containing cOmplete protease inhibitor cocktail (EDTA-free) (Roche, Basel, Switzerland) and lysozyme (0.5 mg mL^{-1}). The re-suspended cells were then lysed by homogenization (10000-15000 psi, 4 passes) and the resulting cell lysate was clarified by centrifugation (30000 x g, 60 min, 4 °C).

The supernatant was collected and subjected directly to dextrin affinity FPLC. The sample was loaded *via* sample pump onto a 5 mL MBPTrap FF cartridge (GE Healthcare, IL, USA) affixed to an ÄKTA Pure FPLC system (GE Healthcare, IL, USA) which had been pre-equilibrated in 6 column volumes (CV) of binding buffer (50 mM Tris, 150 mM NaCl, pH 8.0). The cartridge was then washed isocratically with 10 CV of binding buffer, then MBP-tagged protein was eluted from the cartridge into 96-well deep plates with 10 CV of elution buffer (50 mM Tris, 150 mM NaCl, 10 mM maltose, pH 8.0). SDS-PAGE analysis (*Supplementary Figure 37*) showed successful purification of His_6-MBP-TEV-CaM (K148U).

Purified His_6-MBP-TEV-CaM (K148U) was then directly subjected to TEV protease cleavage of the His_6-MBP tag. The purified construct was dosed with SuperTEV protease (~3 mg) and immediately dialyzed into 50 mM Tris.HCl, 300 mM NaCl, 5 mM 2-mercaptoethanol, 0.5 mM EDTA, pH 7.5 at 4 °C for 16 h to effect TEV cleavage. SDS-PAGE analysis showed successful cleavage of the His_6-MBP tag (*Supplementary Figure 38*) and the TEV cleavage solution was

then directly subjected to Ni-NTA affinity chromatography using a 5 mL HisTrap FF column (GE Healthcare, IL, USA) affixed to an ÄKTA Pure FPLC system (GE Healthcare, IL, USA) which had been pre-equilibrated in 6 column volumes (CV) of binding buffer (50 mM Tris, 150 mM NaCl, pH 8.0). The column flow through, containing (non-His_6-tagged) CaM (K148U) was collected and SDS-PAGE analysis showed successful purification of CaM (K148U) as its asymmetric 2-mercaptoethanol–CaM (K148U) selanyl sulfide from cleaved His_6-MBP by-product.

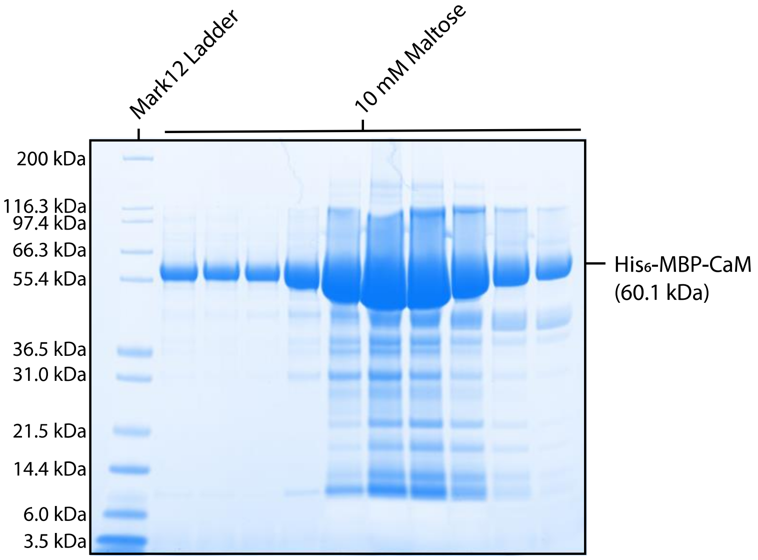

Supplementary Figure 37. Representative SDS-PAGE analysis (Bolt 4-12%, MES running buffer, 165 V, 400 mA, 35 min, Coomassie G-250) of MBPTrap FF (1 mL) FPLC-purified His_6-MBP-TEV-CaM (K148U) from a single large scale expression.

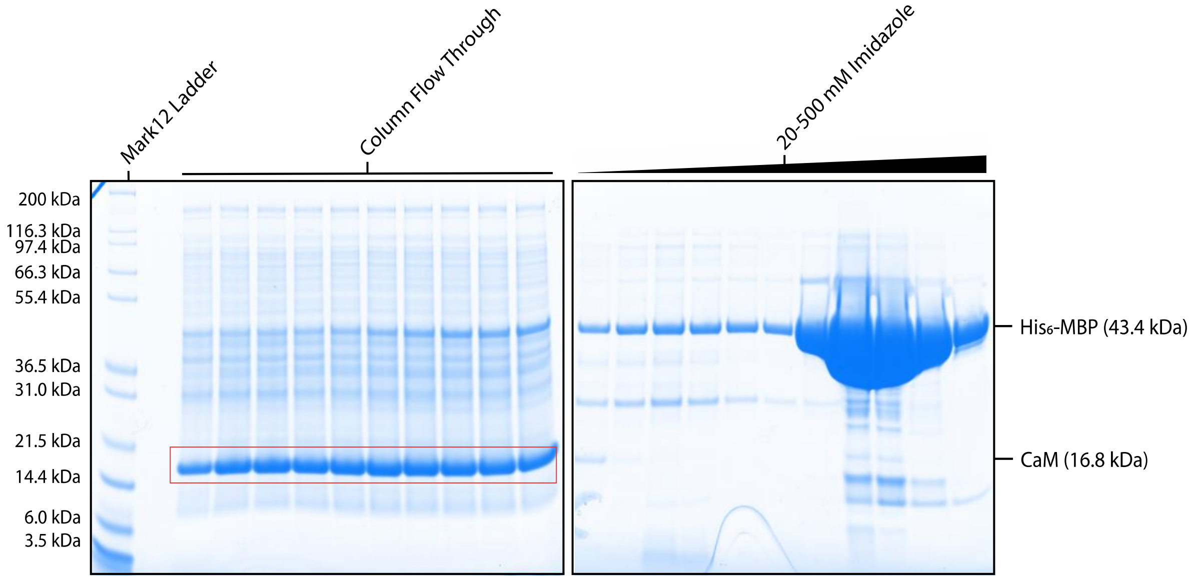

Supplementary Figure 38. Representative SDS-PAGE analysis (Bolt 4-12%, MES running buffer, 165 V, 400 mA, 35 min, Coomassie G-250) of TEV protease cleavage after 18 h on the substrate His_6-MBP-TEV-CaM (K148U) from a single large scale expression.

The 2-mercaptoethanol–CaM (K148U) selanyl sulfide (directly from reverse Ni-NTA affinity purification) was reduced through addition of a spatula tip of TCEP and vortex mixing for approximately 1 min. The solution was then immediately purified by RP-HPLC on a Waters Symmetry C4 (300 Å, 5 μm, 19 × 150 mm) column over a gradient of 30-50% B over 60 min and a flow rate of 16 mL min^{-1}, then lyophilized to afford pure [CaM (K148U)]$_2$ diselenide **12** (13.8 mg, 0.411 μmol) as a white solid (*Supplementary Figure 39*).

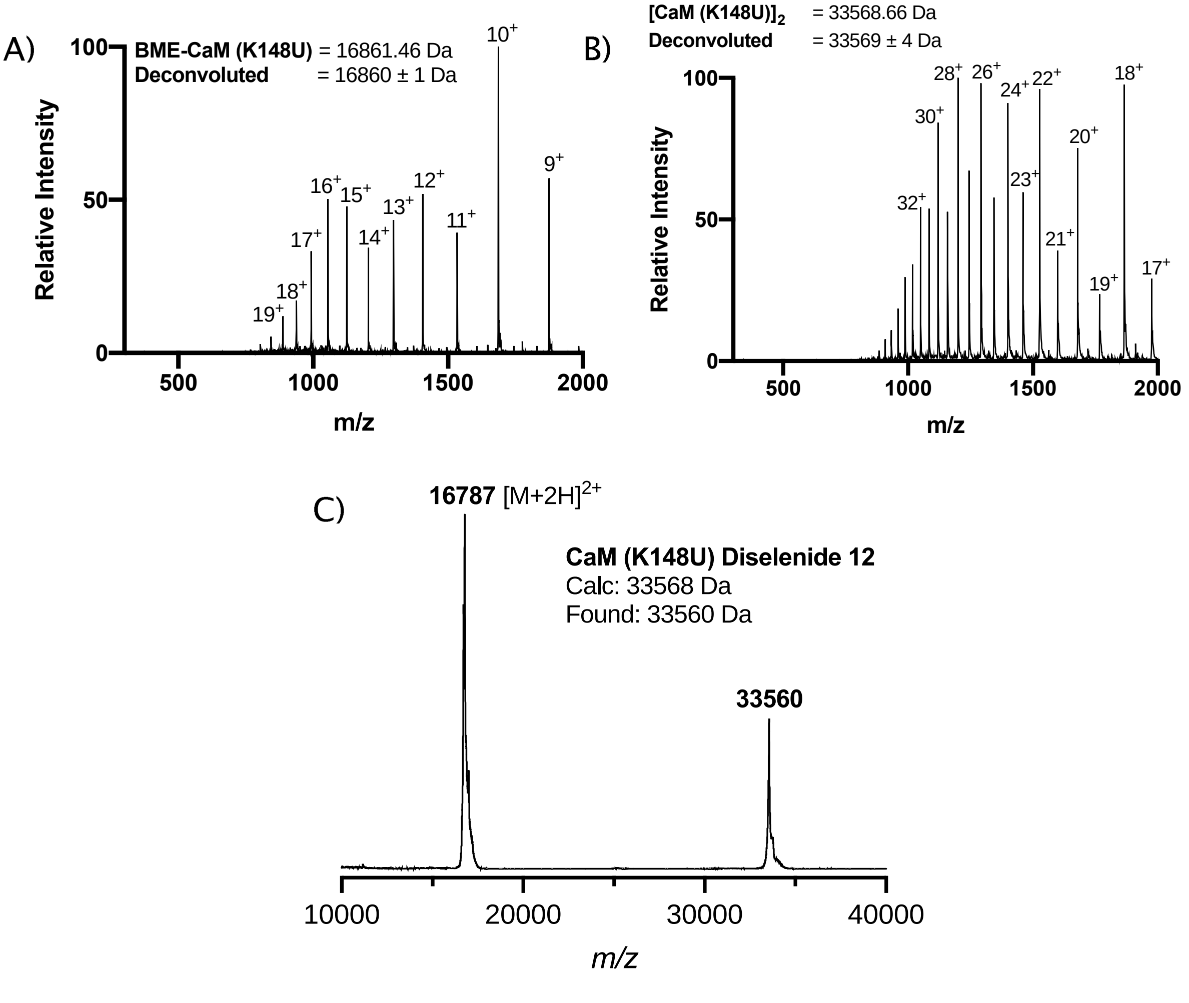

Supplementary Figure 39. LRMS (ESI+) spectrum and deconvolution of **A)** BME-CaM (K148U) selenylsulfide adduct, and **B)** reduced and desalted [CaM (K148U)]$_2$ diselenide **12**. **C)** MALDI-TOF MS spectrum of purified [CaM (K148U)]$_2$ diselenide **12**.

Attempted PDC Dimerization of CaM (K148U) Diselenide 12

In a 1.5 mL Protein LoBind (Eppendorf, Hamburg, Germany) microcentrifuge tube, CaM (K148U) diselenide **12** (1.15 mg, 30 nmol), PTA (**4**) (0.019 mg, 120 nmol, 4 eq.) [from a stock solution in 1:1 v/v MeCN:H_2O] and [Ir(dF(CF_3)ppy)$_2$(dtbpy)]PF_6 (**5**) (2 mol% relative to **12**) [2 μL from a 0.3 μmol mL^{-1} stock in 1:1 v/v MeCN:H_2O] were dissolved in 60 μL of 50 vol% MeCN in 1 M Gn.HCl, 0.02 M NaP_i, pH 7.0 buffer under a stream of N_2 to achieve a protein diselenide **12** concentration of 0.5 mM. The reaction tube was capped and immediately irradiated with 450 nm LED light (PennOC M1 photoreactor) at 37 °C for 5 min.

Two 5 μL aliquots were taken at 0 min and 5 min time points and individually diluted 4-fold in 6 M Gn.HCl, 0.1 M NaP_i, pH 7.0 buffer (15 μL). One aliquot was desalted using a C18 ZipTip (Merck, Darmstadt, Germany) and analyzed MALDI-TOF MS (*see General Procedures*). The other diluted aliquot was directly analyzed by LC-HRMS (*see General Procedures*). Reaction conversion was calculated through averaging integrations of HRMS-derived extracted ion chromatograms of the $[M+11H]^{11+}$, $[M+10H]^{10+}$ and $[M+9H]^{9+}$ charge states. Conversion errors are reported as the standard deviation of these three ion peak integrations for a single experiment.

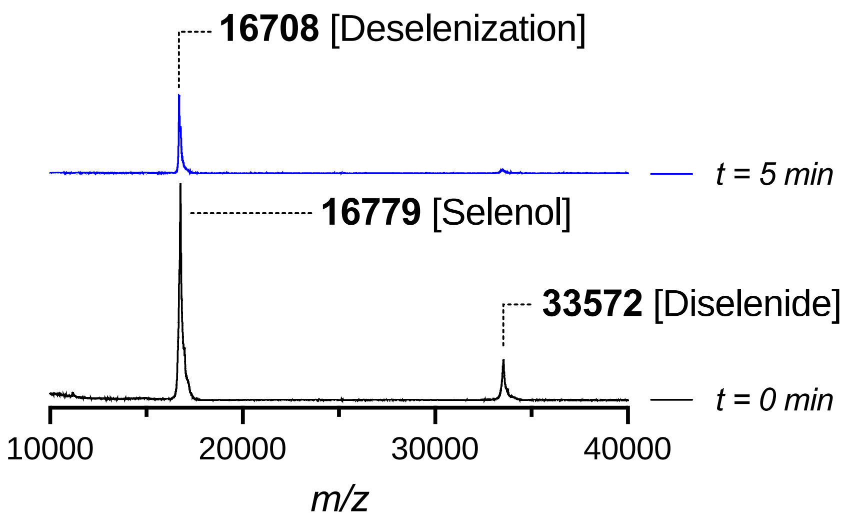

Supplementary Figure 40. MALDI-TOF MS spectra of the reaction mixture of the attempted PDC dimerization of CaM (K148U) diselenide **12** at 0 min (black) and 5 min (blue) irradiation.

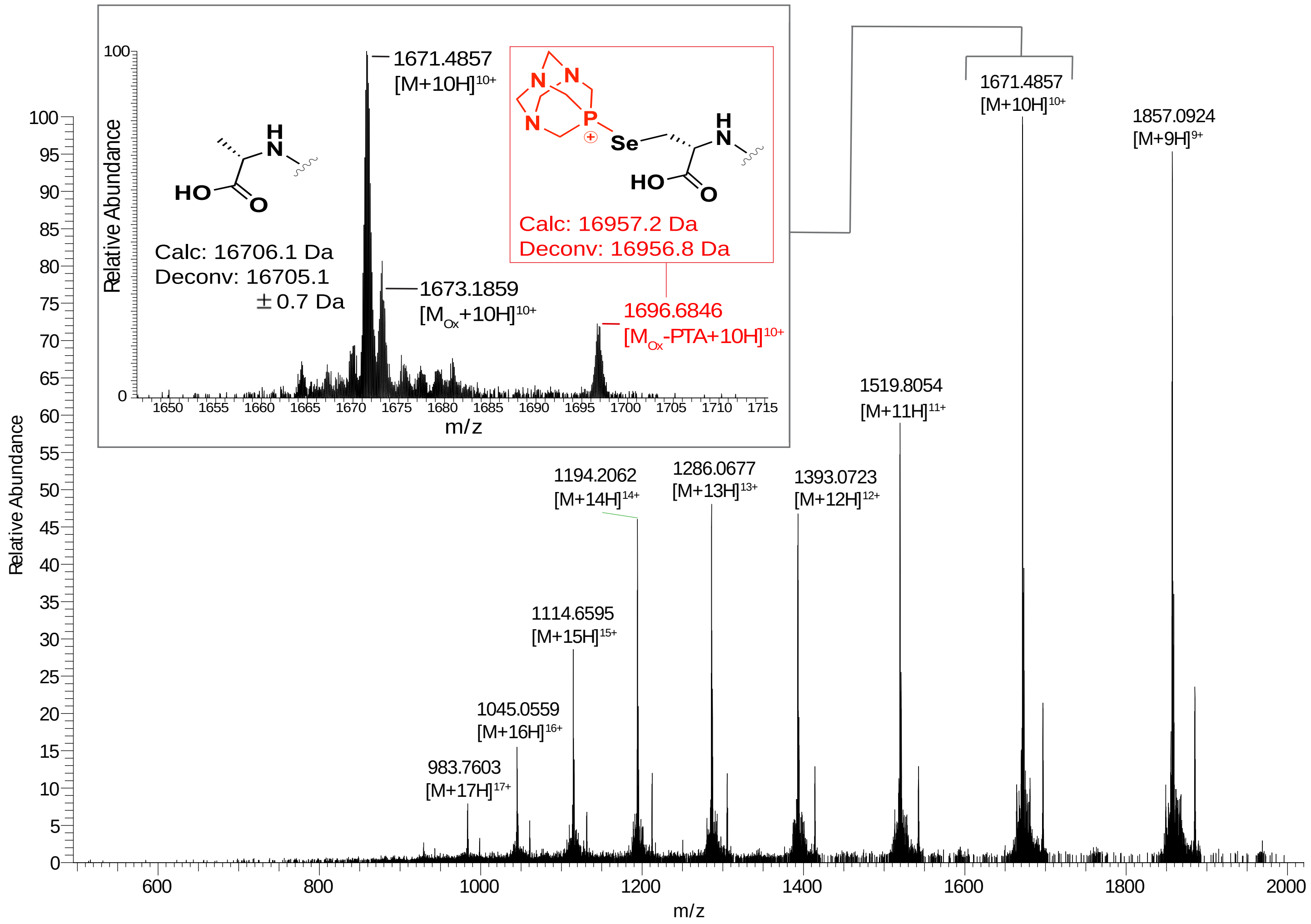

Supplementary Figure 41. HRMS spectrum of the reaction mixture of the attempted PDC dimerization of CaM (K148U) diselenide **12** after 5 min irradiation with expanded $[M+10H^{+}]^{10+}$ ion displaying peaks for the deselenization by-product, CaM (A148) **13** (1671.4857 *m/z*), and the observed selanylphosphonium reaction intermediate **14** (1696.6846 *m/z*).

Photoluminescence (PL) Spectroscopy and Cyclic Voltammetry (CV)

Photoluminescence (PL) Spectroscopy

Time correlated single photon counting (TSCPC) experiments were performed using a lifetime fluorimeter (DeltaFlex, Horiba). For measurements involving [Ir(dF(CF$_3$)ppy)$_2$(dtbpy)]PF$_6$ (**5**) and PTA (**4**), **5** was excited by selecting the output of SuperK extreme/fianium series laser (λ = 500 nm, bandwidth 10 nm, power 4.0 mW, repetition rate 304 kHz, pulse width 150 ps, spot size ~ 1 cm^2).

For experiments involving a TAMRA-labelled diselenide **15**, the excitation was provided by selecting the output of SuperK extreme/fianium series laser (λ = 500 nm, bandwidth 10 nm, power 4.0 mW, repetition rate 304 kHz, pulse width 150 ps, spot size ~ 1 cm^2). The sample was continuously irradiated with a 450 nm light source from a PennOC M1 photoreactor. Emission was collected using a plano-convex lens (focal length: 25 mm) and further collimated using a biconvex lens (focal length:100 mm). An emission cut-off filter (580 nm) was used to remove contributions from stray excitation light. Time-resolved emission spectra were collected by scanning the emission monochromator from 580 nm to 750 nm (step size: 15nm) where emission decay was monitored at each wavelength for 60 secs.

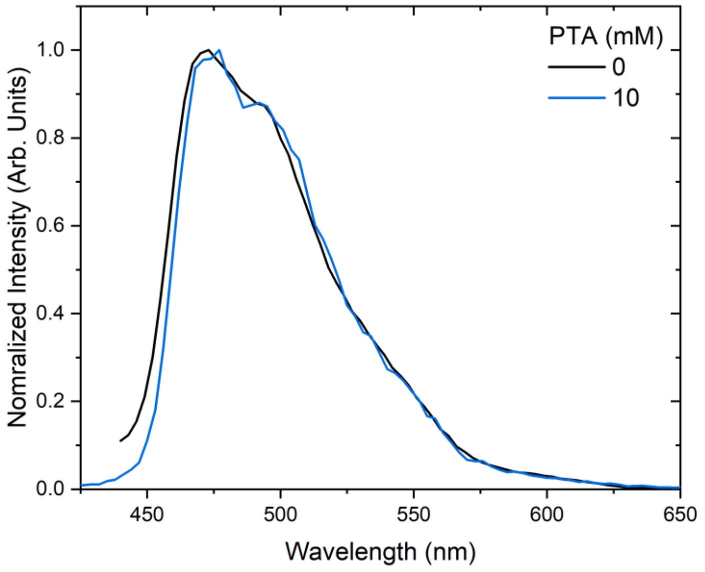

Supplementary Figure 42. Steady state photoluminescence (PL) spectra of an Argon-sparged 1:1 v/v H_2O:MeCN (as per typical PDC reaction conditions) solution of [Ir(dF(CF_3)ppy)$_2$(dtbpy)]PF_6 (**5**) with 0 mM (black line) and 10 mM (blue line) PTA (**4**) added. The excitation wavelength was 415 nm. No significant changes in the PL shape is observed with the addition of PTA to [Ir(dF(CF_3)ppy)$_2$(dtbpy)]PF_6. Arb. Units on the y-axis = arbitrary units.

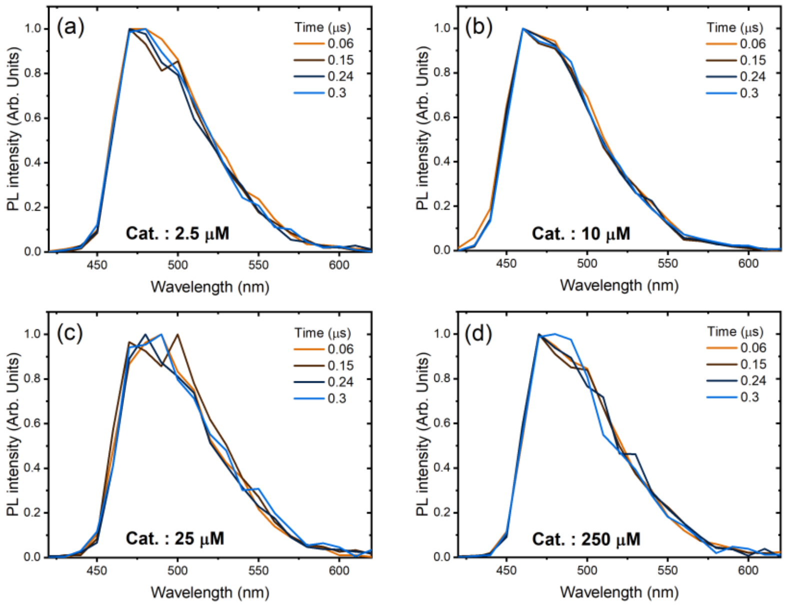

Supplementary Figure 43. Time resolved photoluminescence (PL) spectra of Argon-sparged 1:1 v/v H_2O:MeCN (as per typical PDC reaction conditions) solutions of [Ir(dF(CF_3)ppy)$_2$(dtbpy)]PF_6 (**5**) (Cat) with 10 mM PTA (**4**) added for (a) 2.5 µM, (b) 10 µM, (c) 25 µM and (d) 250 µM concentration of Cat. The excitation wavelength was 415 nm. No significant changes in the PL shapes are observed throughout the lifetime of Cat in presence of PTA. Arb. Units on the y-axis = arbitrary units.

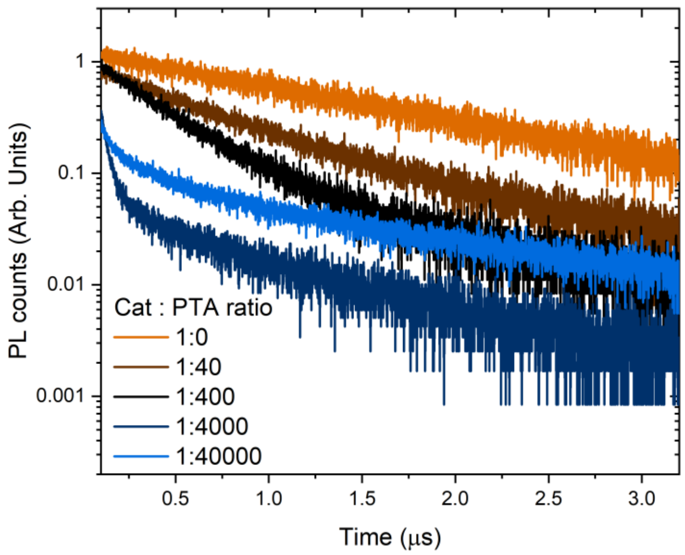

Supplementary Figure 44. Photoluminescence (PL) counts of an Argon-sparged 1:1 v/v H_2O:MeCN (as per typical PDC reaction conditions) solution of [Ir(dF(CF_3)ppy)$_2$(dtbpy)]PF_6 catalyst (Cat) (**5**) monitored at 470 nm with different molar ratios of PTA (**4**) added. The excitation wavelength was 415 nm. With an increasing ratio of PTA to Cat, a decrease in the lifetime of Cat is observed suggesting the emergence of additional decay pathways of the excitation. Arb. Units on the y-axis = arbitrary units.

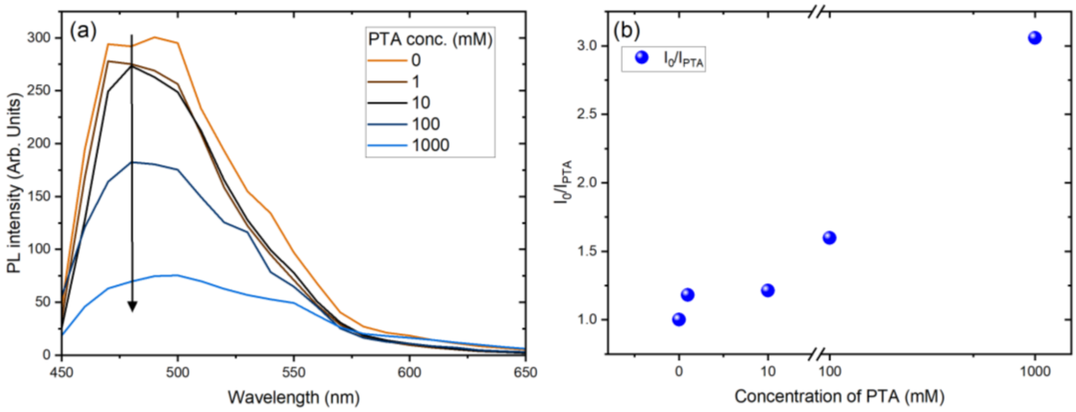

Supplementary Figure 45. **A)** Photoluminescence (PL) spectra of 25 μM solutions of Ir photocatalyst (**5**) monitored with different concentrations of PTA added. **B)** Stern-Volmer plot for the quenching of **5** due to PTA obtained by dividing the integrated PL intensity of 5 with (I_{PTA}) and without (I_0) PTA added. Excitation wavelength = 415 nm. Arb. Units on the y-axis = arbitrary units.

Cyclic Voltammetry (CV)

Cyclic voltammetry experiments were performed using a BASi-Epsilon potentiostat and an undivided electrochemical cell containing a glassy carbon working electrode, platinum wire counter electrode and silver wire pseudoreference electrode. Ferrocene was used as a reference standard. Experiments were conducted at a scan rate of 0.1 $V.s^{-1}$ in 1:1 v/v MeCN:H_2O with 0.1 M NaCl. Immediately prior to collecting each voltammogram, 0.6 mL of 1:1 v/v MeCN:H_2O with 0.1 M NaCl was sparged with Ar for 10 min and then used to solvate one of the following:

1. PTA (**4**) (0.94 mg, 6.00 µmol)
2. PEG_6 diselenide (**16**) (4.13 mg, 6.00 µmol)
3. Succinyl diselenide (2.68 mg, 6.00 µmol)

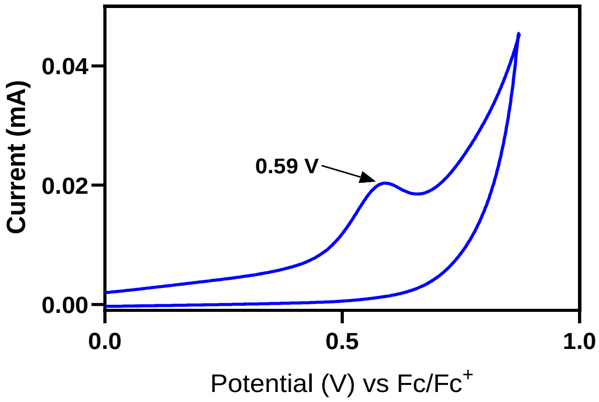

Supplementary Figure 46. Cyclic voltametric profile of PTA (**4**) in 1:1 v/v MeCN:H_2O with 0.1 M NaCl. Scan rate: 0.1 $V.s^{-1}$.

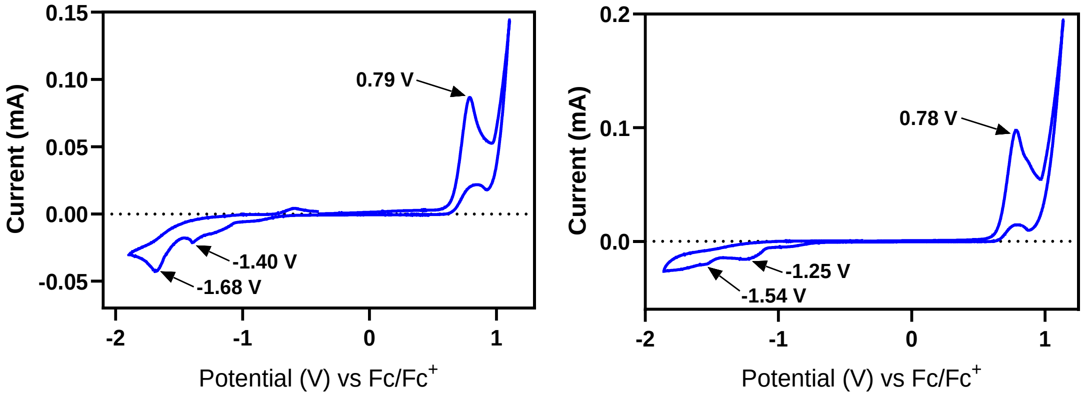

Supplementary Figure 47. Cyclic voltametric profile in 1:1 v/v MeCN:H_2O with 0.1 M NaCl of **A)** PEG_6 diselenide **16** and **B)** succinyl diselenide. Scan rate: 0.1 $V.s^{-1}$.

Additional PL experiments

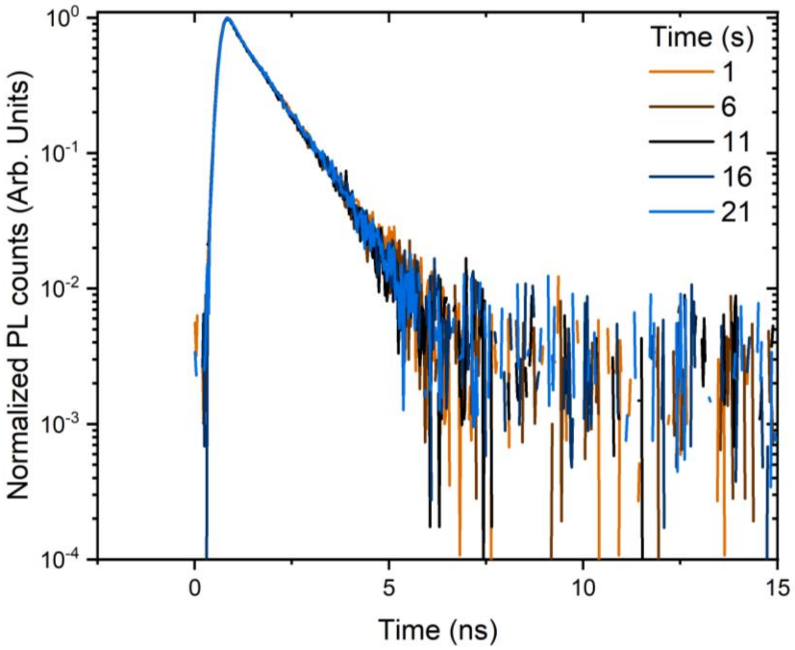

Supplementary Figure 48. Photoluminescence (PL) counts of an Argon-sparged 1:1 v/v H_2O:MeCN (as per typical PDC reaction conditions) solution of diselenide derivatised 5-carboxytetramethylrhodamine (TAMRA) (**15**) in the presence of $[Ir(dF(CF_3)ppy)_2(dtbpy)]PF_6$ (**5**) and PTA (**4**) monitored at 615 nm under constant UV exposure. The excitation wavelength was 415 nm. Arb. Units on the y-axis = arbitrary units.

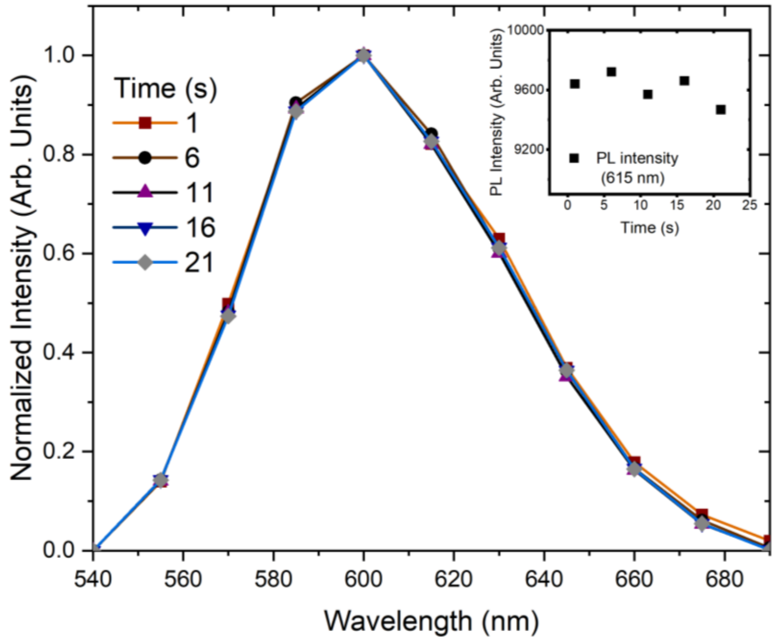

Supplementary Figure 49. Normalized photoluminescence (PL) spectra of an Argon-sparged 1:1 v/v H_2O:MeCN (as per typical PDC reaction conditions) solution of diselenide derivatised 5-carboxytetramethylrhodamine (TAMRA) (**15**) and 25 µM [Ir(dF(CF_3)ppy)$_2$(dtbpy)]PF_6 (**5**) in the absence of PTA (**4**). The excitation wavelength was 550 nm. The spectra are monitored at different times under constant exposure to UV light. The inset figure shows the changes in PL intensity monitored at 615 nm at different times under constant exposure to UV light. Arb. Units on the y-axis = arbitrary units.

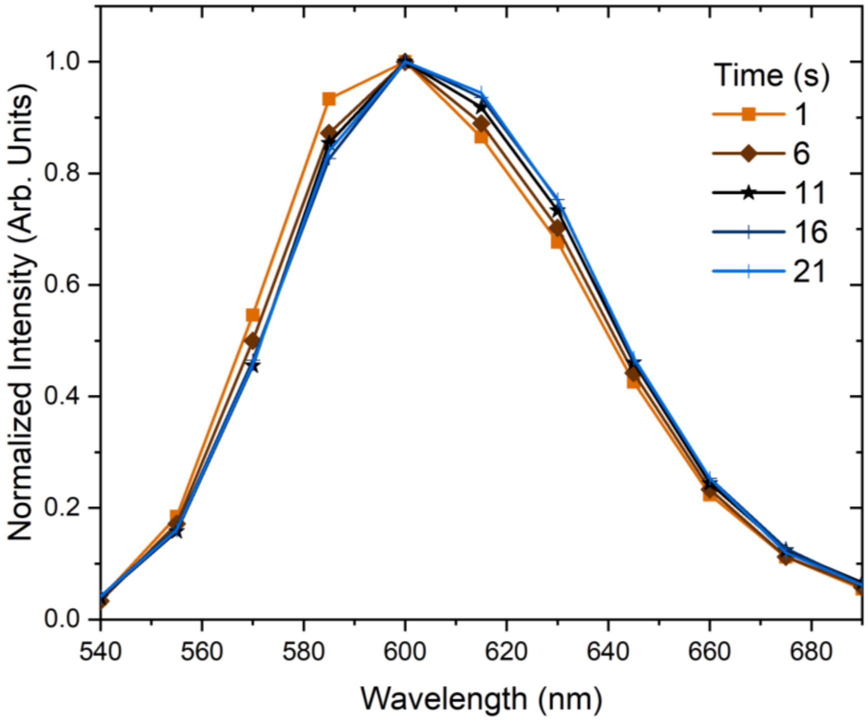

Supplementary Figure 50. Normalized photoluminescence (PL) spectra of an Argon-sparged 1:1 v/v H_2O:MeCN (as per typical PDC reaction conditions) solution of diselenide derivatised 5-carboxytetramethylrhodamine (**15**) and 25 µM [Ir(dF(CF_3)ppy)$_2$(dtbpy)]PF_6 (**5**) with 10 mM PTA (**4**) added. The excitation wavelength was 550 nm. The spectra are monitored at different times under constant exposure to UV light. Arb. Units on the y-axis = arbitrary units.

^{31}P NMR Spectroscopy Studies on $[H_2N\text{-USPGYS-}NH_2]_2$

$[H_2N\text{-USPGYS-}NH_2]_2$ diselenide (**1**) (1.70 mg, 1.10 μmol) was dissolved in 110 μL of 1:1 v/v D_2O:MeCN-d3 containing $[Ir(dF(CF_3)ppy)_2(dtbpy)]PF_6$ (**5**) (0.1 μmol mL^{-1}) for a photocatalyst loading of 1 mol%. The solution was further diluted with 110 μL of 1:1 v/v D_2O:MeCN-d3 to bring the peptide concentration to 5 mM. This solution was then used to dissolve PTA (**4**) (0.69 mg, 4.40 μmol, 4 eq.) and the resulting reaction mixture was irradiated with 450 nm light for 1 min. After irradiation, UPLC-MS analysis showed complete conversion of the diselenide **1** to selenoether **3**, with minimal deselenization by-product **2**. The reaction mixture was then diluted to 0.5 mL with 1:1 v/v D_2O:MeCN-d3. This crude reaction mixture was then analyzed by analytical HPLC using a gradient of 1-20% B over 30 min (λ = 214 nm), and by ^{31}P NMR spectroscopy with KPF_6 (0.920 mg, 5.00 μmol) used an internal reference standard (*Supplementary Figure 51*). The observed ^{31}P peaks at -97.15 ppm and -32.85 ppm were assigned as PTA and PTA selenide, respectively, in accordance previously reported values.[5]

Separately, $[H_2N\text{-USPGYS-}NH_2]_2$ diselenide (**1**) (3.86 mg, 2.50 μmol) and PTA (**4**) (1.57 mg, 10.0 μmol) were dissolved in 500 μL of D_2O and analyzed by ^{31}P NMR spectroscopy over the course of 60 min, with spectra recorded at t = 0, 5, 10, 30 and 60 min. KPF_6 (0.920 mg, 5.00 μmol) was used an internal reference standard. ^{31}P spectra from each timepoint were overlaid to provide insight into potential background reactions occurring between the PTA and $[H_2N\text{-USPGYS-}NH_2]_2$ diselenide (**1**) (*Supplementary Figure 52*). The only observed change over the course of 60 min was the appearance of a peak at -2.84 ppm after 30 min, which was assigned as PTA oxide.[5] This contrasts with the ^{31}P spectrum when $[H_2N\text{-USPGYS-}NH_2]_2$ diselenide (**1**) was subjected to optimised PDC reaction conditions, which contains a peak at -32.85 ppm (*Supplementary Figure 51*). As mentioned above, this peak was assigned as PTA selenide, a by-product of the PDC reaction which was also observed by UPLC-MS and analytical HPLC (*Supplementary Figure 51*).

See next pages for experimental data.

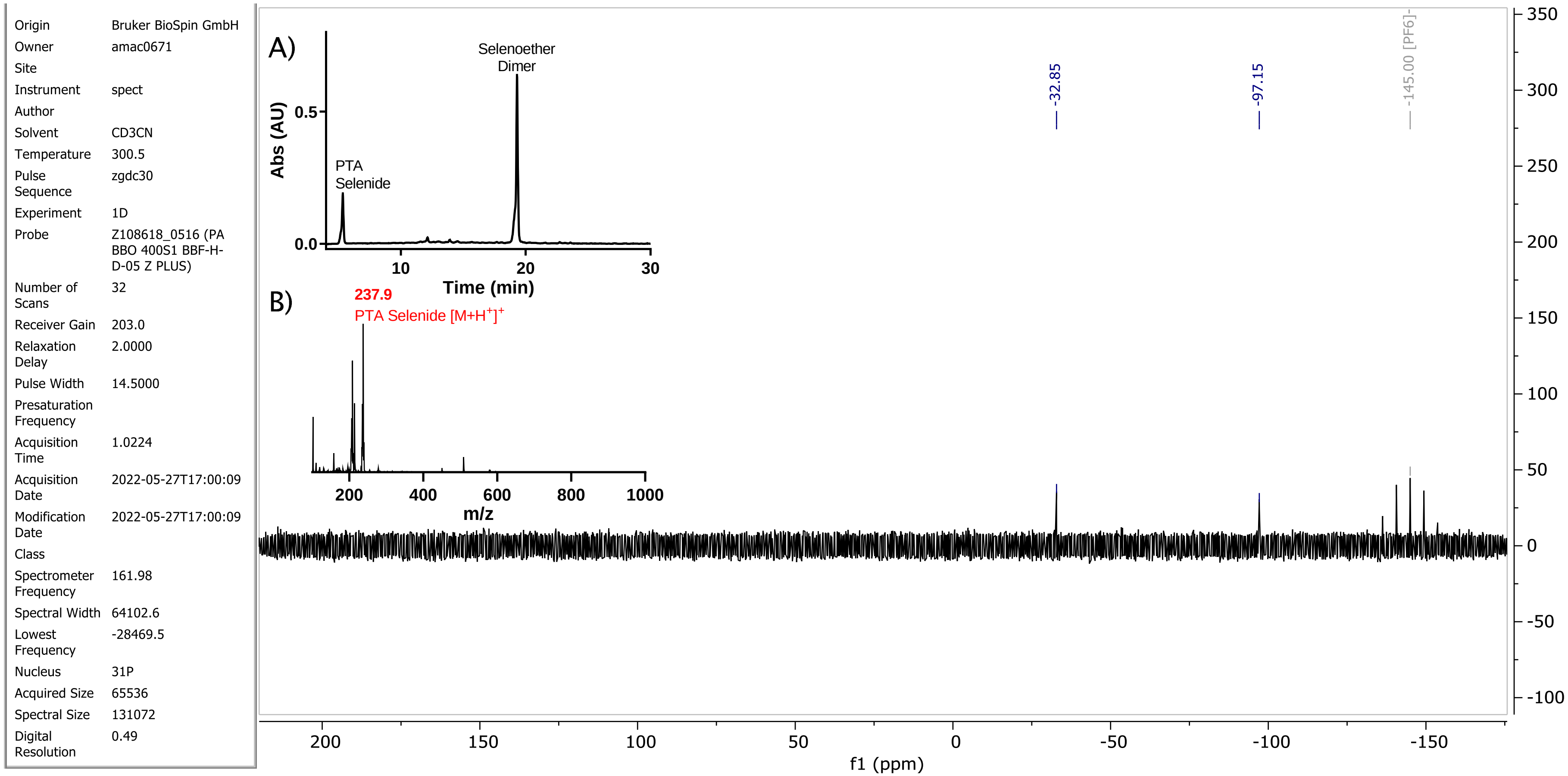

Supplementary Figure 51. ^{31}P NMR spectra (162 MHz, 1:1 D_2O:MeCN-d3) after $[H_2N\text{-USPGYS-}NH_2]_2$ diselenide (**1**) was subjected to optimised PDC reaction conditions for 1 min, displaying key ^{31}P peak shifts: PTA (-97.15 ppm) and PTA selenide (-32.85 ppm). KPF_6 used as an internal reference standard ($[PF_6]^-$ ^{31}P shift at -145 ppm). Inset: **A)** analytical HPLC analysis (1-20% B over 30 min, λ = 214 nm) of the crude reaction mixture after $[H_2N\text{-USPGYS-}NH_2]_2$ diselenide (**1**) was subjected to optimised PDC reaction conditions for 1 min, and **B)** associated mass spectrum averaged over the analytical HPLC peak assigned as PTA selenide.

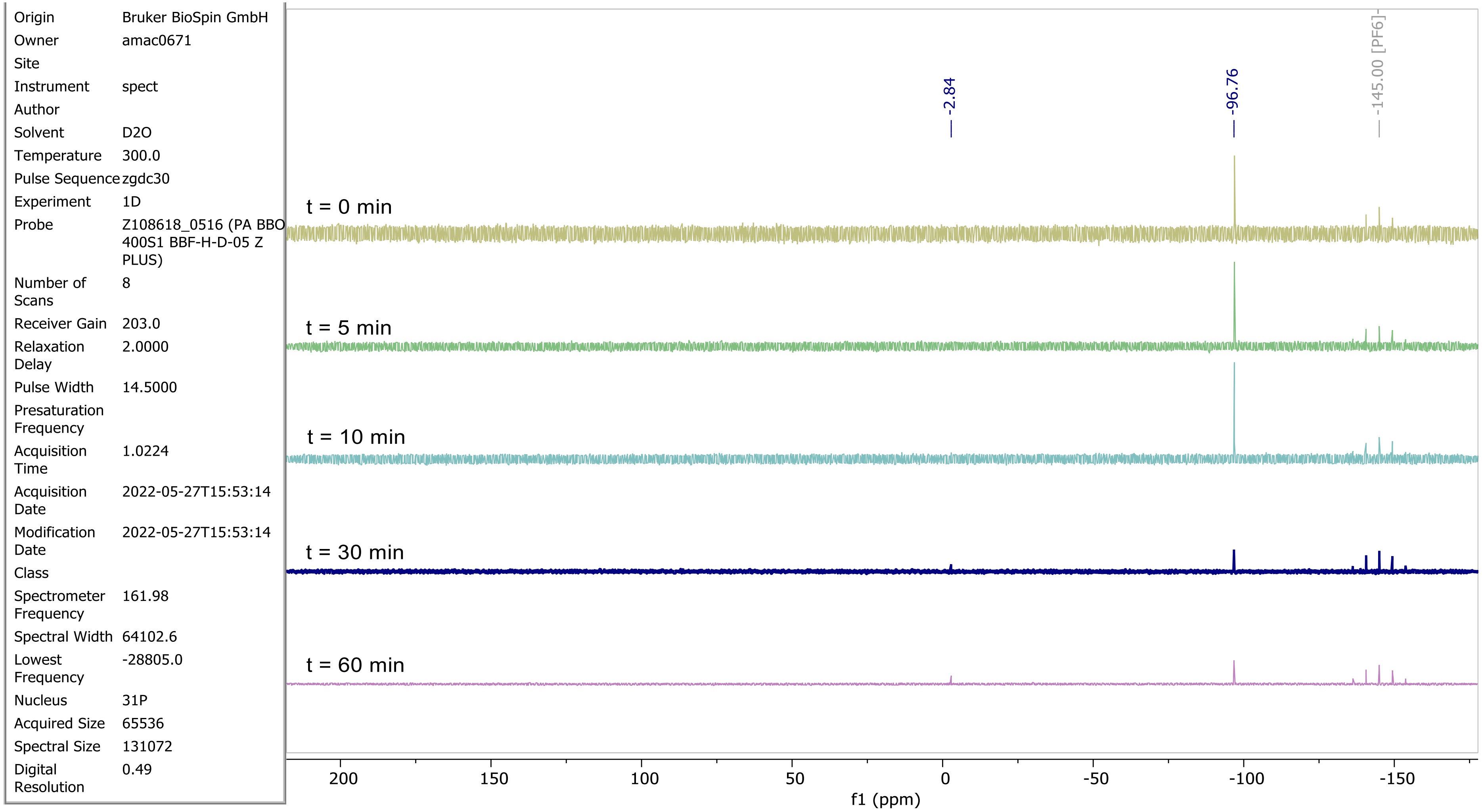

Supplementary Figure 52. Overlaid ^{31}P NMR spectra (162 MHz, D_2O) of $[H_2N\text{-}USPGYS\text{-}NH_2]_2$ diselenide (**1**) and PTA (**4**) after 0, 5, 10, 30 and 60 min, displaying key ^{31}P peak shifts: PTA (-96.76 ppm) and PTA oxide (-2.84 ppm). KPF_6 used as an internal reference standard ($[PF_6]^-$ ^{31}P shift at -145 ppm).

Computational Mechanistic Studies

Computational Methods

The range-separated dispersion-corrected B3PW91 density functional[6-8] and the 6-31+G(d,p)[9-13] basis set (def2-SVP[14,15] for Ir atoms) were used with Grimme's D3 correction[16] (employing the Becke-Johnson damping function)[17] to optimize the geometries of all stationary points. Single-point energy corrections were included at the B3PW91-GD3(BJ)/6-311+G(2df,p) (def2-TZVPP for Ir atoms) level to refine the calculated electronic energy. Similar levels of theory have proven to be accurate when modelling molecules bearing different types of Se atoms.[18,19] All calculations included the SMD[20] variation of the IEFPCM[21-25] solvation model (solvent = acetonitrile) to account for solvent effects. We evaluated the ability of our chosen level of theory in optimizing structures containing different types of Se atoms by comparison against a structure that has been characterized by X-ray crystallography (*Supplementary Figure 53*). Considering that we used gas phase in this optimization calculation, which is of different polarity to the bulk crystal, and allowing for the possibility of crystal packing effects in the X-ray structures, the level of agreement between theory and experiment is high: Se-Se and Se-C bond distances differ by less than 1.0% (*Supplementary Figure 53, left*), as well as Se=P and P-C bond distances by 1.5% or less (*Supplementary Figure 53, right*). All angles and dihedrals are also well reproduced.

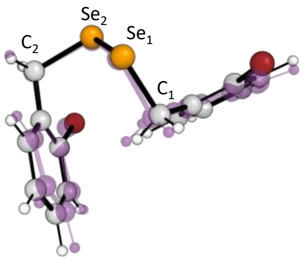

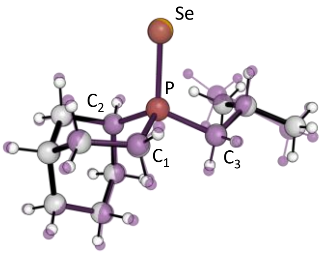

Bond	Bond length (Å) Crystal	Bond length (Å) DFT	Variation (%)
Se-Se	2.303	2.295	-0.3
Se_1-C_1	1.988	2.001	0.7
Se_2-C_2	1.986	1.984	-0.1

Bond	Bond length (Å) Crystal	Bond length (Å) DFT	Variation (%)
Se=P	2.136	2.109	-1.3
P-C_1	1.834	1.833	-0.1
P-C_2	1.822	1.850	1.5
P-C_3	1.825	1.835	0.5

Supplementary Figure 53. Overlay of the X-ray structure of 1,2-bis(2-bromobenzyl)diselane (Crystallography Open Database, COD,[26,27] identifier: 2227239)[28] (left) and 2-isobutyl-2-phosphabicyclo[3.3.1]nonane 2-selenide (COD identifier: 2221079)[29] (right). Crystal structures are represented with standard colors and B3PW91-

GD3(BJ)/6-31+G(d,p) (gas phase) optimized geometries are shown in purple. Tabulation of multiple bond lengths determined experimentally and computationally is also included.

The AQME program[30] was used in the conformational sampling of ground state structures (workflow available in Zenodo[31]). Transition states (TSs) were found by using all the ground state conformations that could connect to the TSs. During the conformational search, we found a great number of unique conformers for each reaction step (i.e. more than 275 structures for the model cysteine derivative, see attached *Thermochemistry.dat* file for more information). To enumerate the different conformers, we used different letters at the end of each name (*i.e.* a, b, c, *etc.*). Representations in the main text and supporting information refer to the most stable rotameric conformation found for each step. G values of all the energy profiles correspond to the Boltzmann weighted G of all the conformers found in each step (G_{av}) (see section *Thermochemical Data Calculation with GoodVibes*).

Vibrational frequency calculations were used to confirm that stationary points were either minima or first-order saddle points on the potential energy surface and to obtain frequencies used to calculate thermochemistry values with the *GoodVibes* program.[32] For more information about calculating thermodata, see the *Thermochemical Data Calculation with GoodVibes* section. Furthermore, intrinsic reaction coordinate (IRC) calculations[33] were carried out to ensure that the intermediates (**Int**) of the different pathways connected to their corresponding transition structure (**TS**).

Gaussian 16[34] was employed for all density functional theory (DFT) calculations, using the default "ultrafine" pruned (99,590) grid for numerical integration of the exchange-correlation functional and its derivatives. Our display settings have been made openly accessible.[35]

Thermochemical Data Calculation with *GoodVibes*

After all geometry optimizations were completed, all duplicated geometries were excluded from the study using the *GoodVibes* program (duplicate detection with the "--check" option). With the same software, quasi-harmonic (QHA) corrections were introduced to the computed vibrational entropies using a frequency cut-off value of 100.0 cm^{-1}, following the model proposed by Grimme[36] at 298.15 K. Also, a correction for the change in standard state from gas phase at 1 atm to a 1 M solution was introduced (option "-c 1").[37] A few of the calculations showed undesired persistent low imaginary frequencies lower than 50 cm^{-1}. These imaginary

frequencies were detected (option "--imag") and inverted manually from the output files to their respective positive values before the QHA entropic corrections were computed as seen in previous examples.[38] Entropy corrections due to entropy of symmetry (option "--ssym"), mixing, and multi-structural effects (option "--pes") were also included.[39-43] Boltzmann weighted G (G_{av}) were also calculated with *GoodVibes* (option "--pes").

All the thermochemical data including absolute energies, zero-point energies (ZPE) and T·S, among other parameters, at the B3PW91-GD3(BJ)/6-31+G(d,p) level, as well as the absolute energies, corrected final G and relative G obtained with B3PW91-GD3(BJ)/6-311+G(2df,p), were generated in an automated way using *GoodVibes* and tabulated in a separate file included in the Supplementary Information. Additionally, the "--check" option was employed to detect any potential input errors in the calculations (i.e. always using same level of theory, program version and solvation model, geometries of single-point corrections match their corresponding optimizations, etc). This process for creating G profiles in an automated manner provides a useful method to avoid errors related to human manipulation of the data. The keyword input line used in *GoodVibes* is also included in the document uploaded as additional Supplementary Information.

Example Input Files

Gaussian 16 optimization (keywords line):

B3PW91/6-31+G(d,p) empiricaldispersion=GD3BJ scrf=(smd,solvent=acetonitrile) opt freq=noraman

Gaussian 16 single-point calculation (keywords line):

B3PW91/6-311+G(2df,p) empiricaldispersion=GD3BJ scrf=(smd,solvent=acetonitrile)

Complete PES and Representations of the Most Stable Conformers

(BnSe)$_2$ model substrate

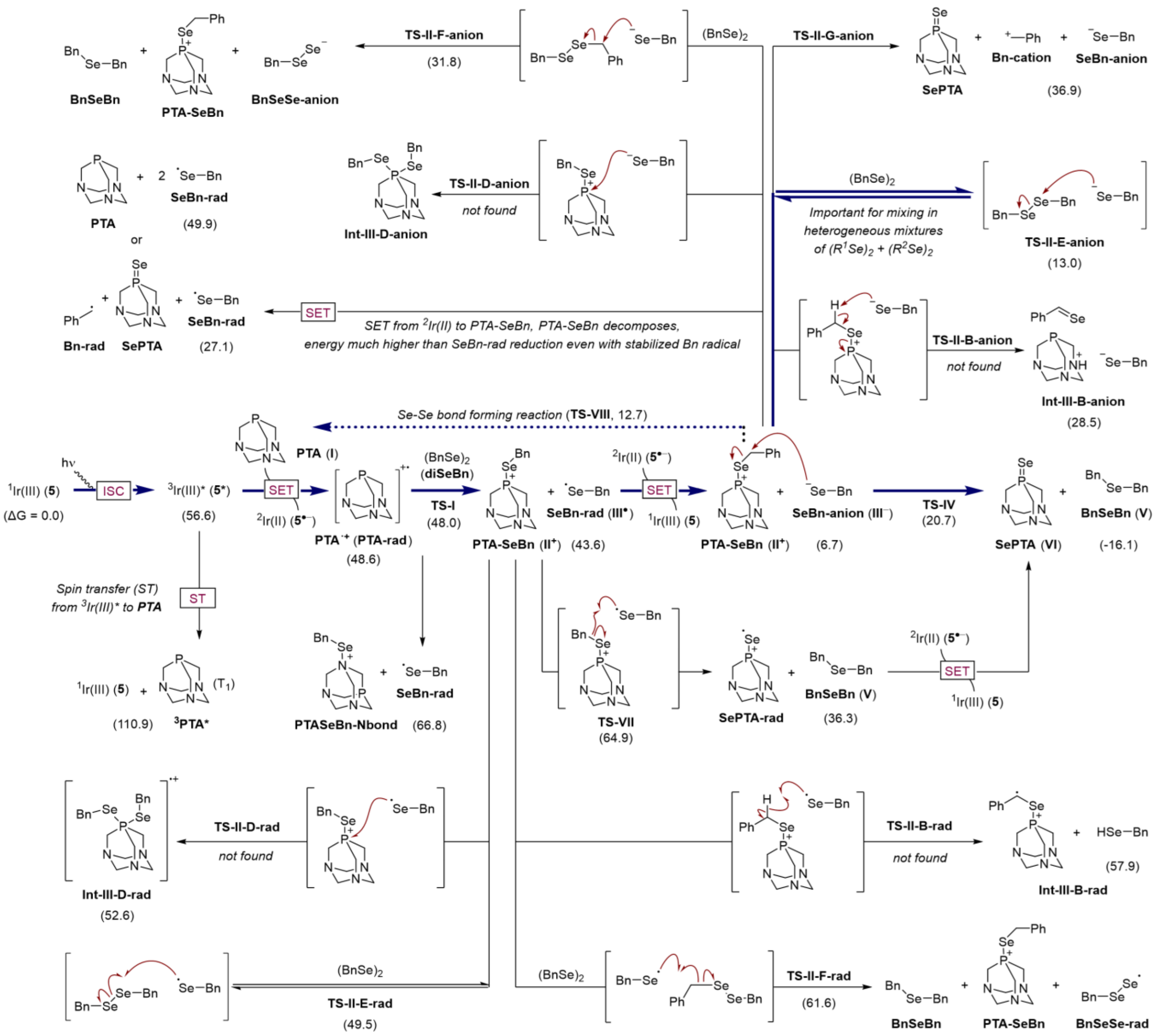

Supplementary Figure 54. Preliminary mechanistic exploration using (BnSe)$_2$ as the model substrate. This model was used to select viable pathways for further studies (marked with thick blue arrows). Prefix "SeBn-Model" was included in the names of intermediates and TSs in the raw data contained in the separated thermochemistry and molecular coordinates files included with the ESI (i.e. **SeBn-Model-TS-IV-anion** for the **TS-IV** step).

Selenocysteine derivative

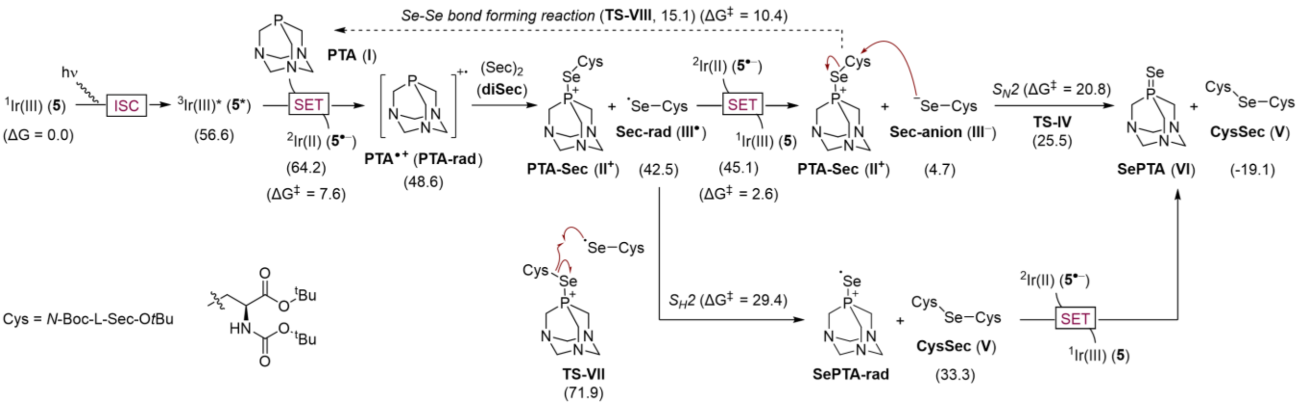

Supplementary Figure 55. G profile of the model selenocysteine derivative, including relative G values (ΔG in kcal·mol^{-1}) of all the steps in parenthesis and activation barriers (ΔG‡) of TSs with respect to their corresponding resting states. Prefix "Sec" was included in the names of intermediates and TSs in the raw data contained in the separated thermochemistry and molecular coordinates files included with the ESI (i.e. **Sec-TS-IV-anion** for the **TS-IV** step).

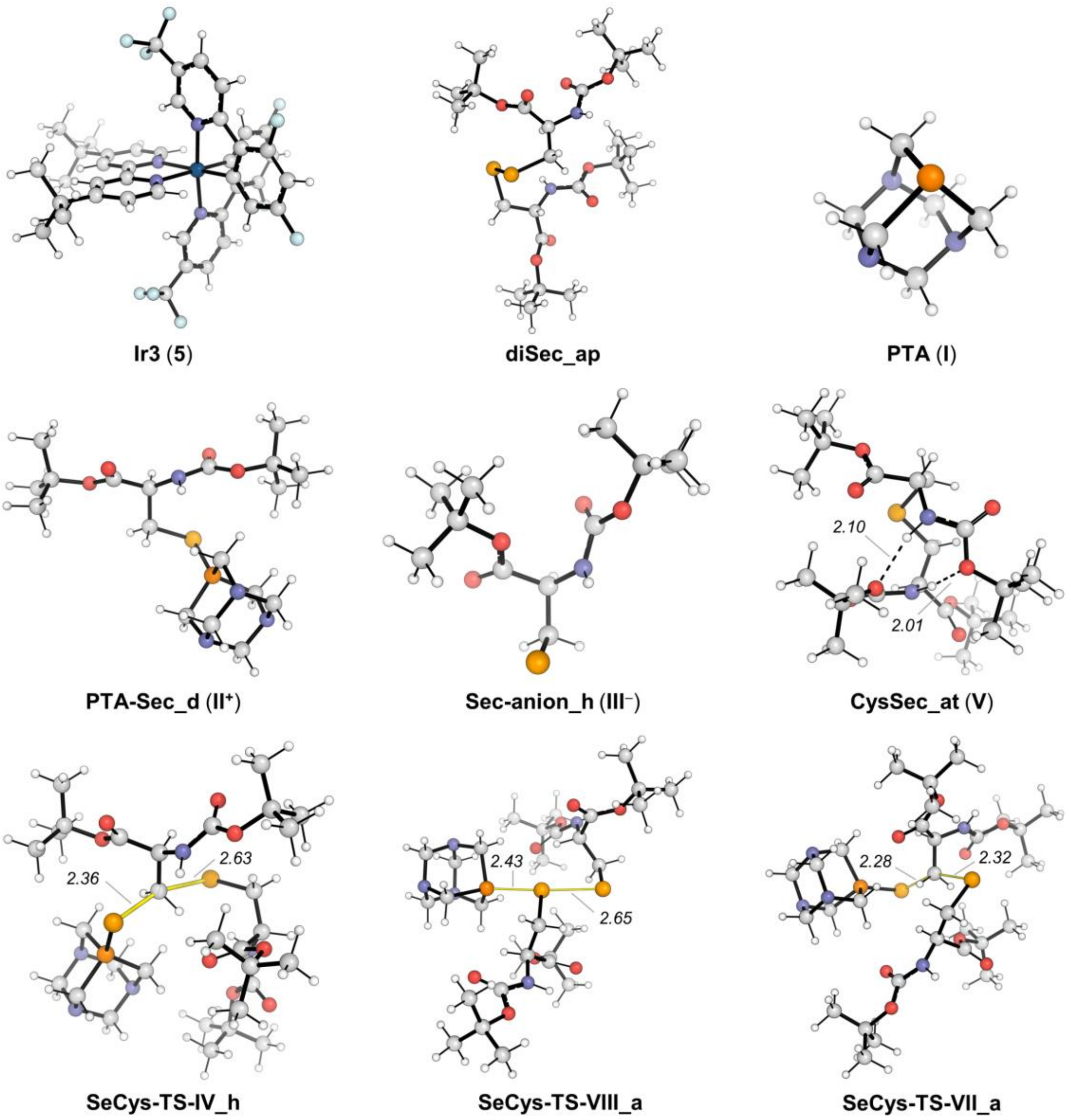

Supplementary Figure 56. Representations of the most stable conformers of selected reaction steps (including conformer suffix and name used in Figure 2) for the model selenocysteine derivative. Yellow thin lines represent bonds involved in the TSs. Black dashed lines correspond to noncovalent interactions. Distances shown in Å.

<u>(C_6F_5-CH_2-Se)$_2$ substrate</u>

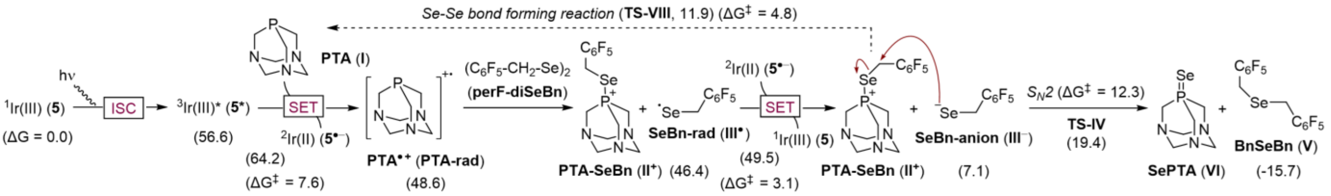

Supplementary Figure 57. Main and competitive anionic reaction pathways of the (C_6F_5-CH_2-Se)$_2$ substrate. Prefix "perF" was included in the names of intermediates and TSs in the raw data contained in the separated thermochemistry and molecular coordinates files included with as separate Supplementary Information files (i.e. **perF-TS-IV-** for the **TS-IV** step).

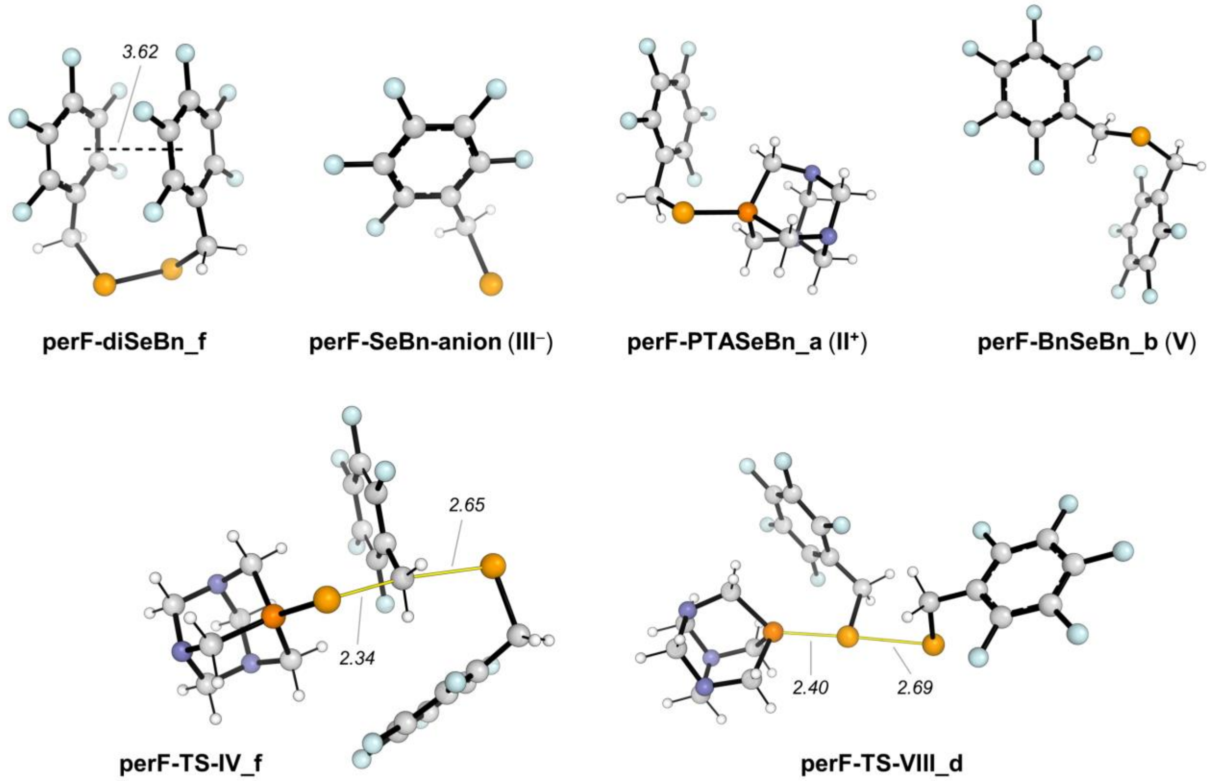

Supplementary Figure 58. Representations of the most stable conformers of selected reaction steps (including conformer suffix and name according to Figure 2) for the (C_6F_5-CH_2-Se)$_2$ substrate. Yellow thin lines represent bonds involved in the TSs. Black dashed lines correspond to noncovalent interactions. Distances shown in Å.

(Alkyl-Se)$_2$ derivatives

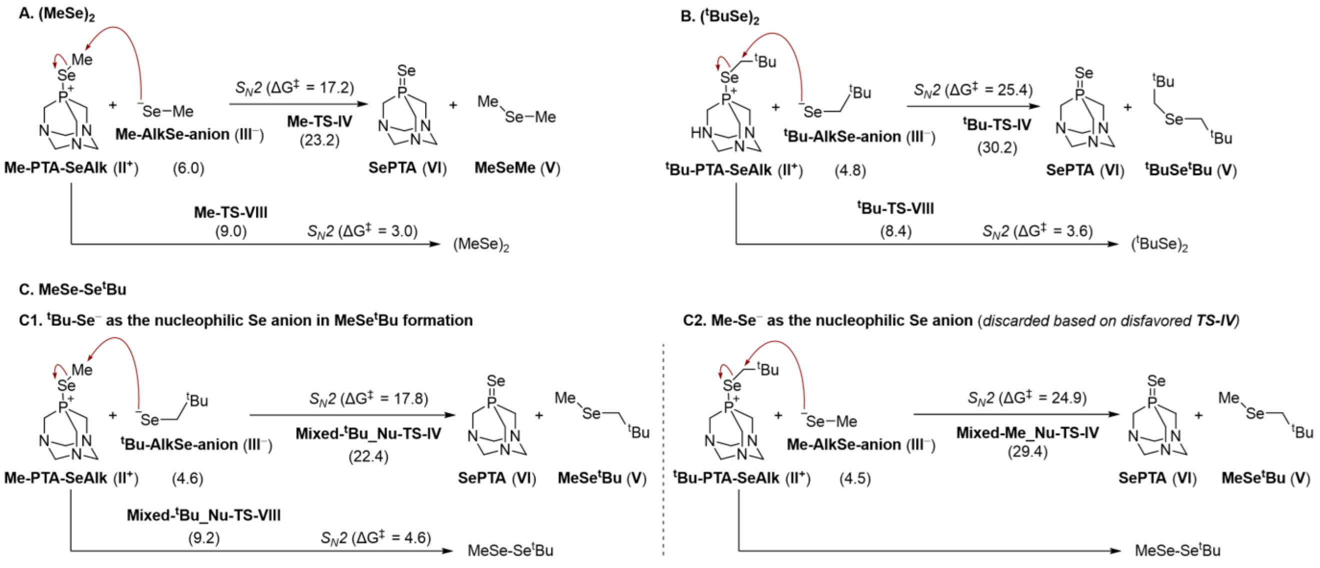

Supplementary Figure 59. Steric studies using (Alkyl-Se)$_2$ derivatives. The names shown in the figure are analogous to the names of the raw data from the separated thermochemistry and molecular coordinates files included as separate Supplementary Information files.

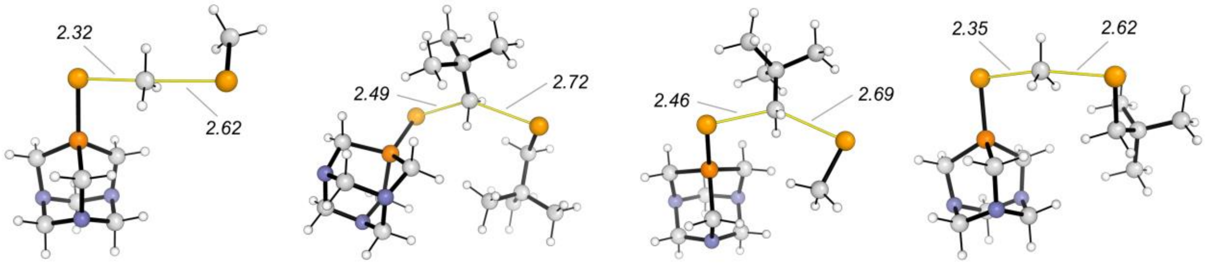

Me-TS-IV_a **^{t}Bu-TS-IV_d** **Mixed-Me_Nu-TS-IV_b** **Mixed-^{t}Bu_Nu-TS-IV_a**

Supplementary Figure 60. Representations of the most stable conformers of TSs (including conformer suffix) for the (Alkyl-Se)$_2$ derivatives. Yellow thin lines represent bonds involved in the TSs. Distances shown in Å.

SET Calculations

Barriers for electron-transfer were estimated using Marcus theory[44,45] with protocols from Houk and Buchwald,[46] Vaissier,[47] and an extended approach of the Houk-Buchwald method. All the methods led to similar conclusions (*Supplementary Figure 61*). The barriers shown in the figures of the manuscript and Supplementary Information, as well as the barriers used in the kinetic models, correspond to the averaged values of the extended Houk-Buchwald's and the Vaissier methods (*Supplementary Table 5*, for more details see the sections below). We did not use the original Houk-Buchwald method to calculate averaged values since nuclear reorganization energies (λ_i) are not significantly smaller than solvent reorganization energies (λ_0) and, therefore, cannot be neglected (*Supplementary Table 7, vide infra*).

Supplementary Table 5. SET energy barriers calculated with different methods. Sec- and perF- prefixes indicate *N*-Boc-L-Sec-O*t*Bu diselenide and (C_6F_5-CH_2-Se)$_2$ derivatives, respectively.

SET process	$\Delta G_{SET}^{\ddagger}$ (Houk-Buchwald) (kcal·mol^{-1})	$\Delta G_{SET}^{\ddagger}$ (Vaissier) (kcal·mol^{-1})	$\Delta G_{SET}^{\ddagger}$ (extended Houk-Buchwald) (kcal·mol^{-1})	$\Delta G_{SET}^{\ddagger}$ (average Vaissier and extended Houk-Buchwald) (kcal·mol^{-1})
**5*** + **I** → **5**$^{\cdot-}$ + **I**$^{\cdot+}$	2.5	10.2	5.0	**7.6**
**Sec-III**· + **5**$^{\cdot-}$ → **Sec-III**$^{-}$ + **5**	15.4	1.7	3.5	**2.6**
**perF-III**· + **5**$^{\cdot-}$ → **perF-III**$^{-}$ + **5**	12.3	1.4	4.7	**3.1**

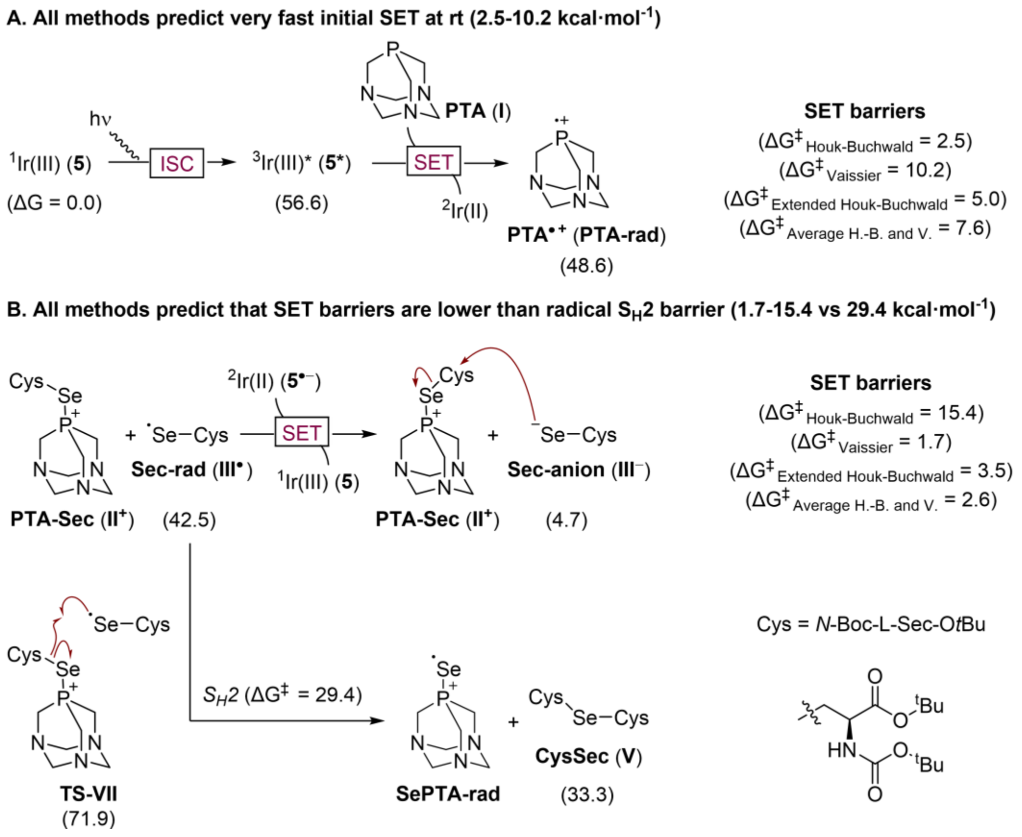

Supplementary Figure 61. Relevant conclusions obtained after the calculation of SET energy barriers.

Houk-Buchwald method

The original formula was employed to calculate SET barriers ($\Delta G_{SET}^{\ddagger}$) as follows:

$$\Delta G_{SET}^{\ddagger} = \Delta G_0^{\ddagger}\left(1 + \frac{\Delta G_r}{4\Delta G_0^{\ddagger}}\right)^2 \qquad (1)$$

where $\Delta G_0^{\ddagger}$ corresponds to the intrinsic barrier (i.e. the activation energy at zero driving force) for the outer-sphere electron transfer and ΔG_r is the reaction energy (G difference between the steps before and after the SET). $\Delta G_0^{\ddagger}$ is calculated using the reorganization energy (λ) using the equation:

$$\Delta G_0^{\ddagger} = \frac{\lambda}{4} = \frac{\lambda_0 + \lambda_i}{4} \qquad (2)$$

where λ_0 is the solvent reorganization energy and λ_i is the inner reorganization energy for the reactants. As in the original paper, we estimate $\lambda_i \approx 0$ in this approach, since the inner reorganization energies in stepwise electron transfer reactions are usually small:

$$\Delta G_0^{\ddagger} = \frac{\lambda}{4} = \frac{\lambda_0}{4} \qquad (3)$$

However, it is worth to mention that λ_i was found to be significant using DFT when following Vaissier's approach and, therefore, a variation of the Houk-Buchwald method including DFT-calculated λ_i was also considered (see below). λ_0 was calculated using the formula:[45]

$$\lambda_0 = (\Delta e)^2 \left(\frac{1}{2a_1} + \frac{1}{2a_2} - \frac{1}{R}\right)\left(\frac{1}{\varepsilon_{op}} - \frac{1}{\varepsilon}\right) \quad (4)$$

where a_1 and a_2 are the radii of the molecules involved in the SET process (calculated in the DFT study), R = a_1 + a_2 (used as an estimation for the center-to-center separation distance of the reactants as proposed originally by Marcus[44]) ε_{op} is the optical dielectric constant (calculated as the square of the refractive index of acetonitrile at 20 °C, $1.344^2 = 1.806$),[48] ε is the dielectric constant of acetonitrile (36.64)[49] and Δe is the charge transferred from one reactant to the other (1 e in this case). The formula was adapted in the original publication to kcal·Å·mol^{-1} using Coulomb's constant (332 kcal·Å·mol^{-1}·e^{-2}):

$$\lambda_0 = 332\ (kcal \cdot \text{Å} \cdot \text{mol}^{-1}) \left(\frac{1}{2a_1} + \frac{1}{2a_2} - \frac{1}{R}\right)\left(\frac{1}{\varepsilon_{op}} - \frac{1}{\varepsilon}\right) \quad (5)$$

Then, combining equations (3) and (5):

$$\Delta G_0^{\ddagger} = \frac{\lambda_0}{4} = \frac{332\ (kcal \cdot \text{Å} \cdot \text{mol}^{-1}) \left(\frac{1}{2a_1} + \frac{1}{2a_2} - \frac{1}{R}\right)\left(\frac{1}{1.806} - \frac{1}{36.64}\right)}{4} \quad (6)$$

Using equations (1) and (6), $\Delta G_{SET}^{\ddagger}$ barriers were calculated for the reactions with the selenocysteine derivative and the $(C_6F_5\text{-}CH_2\text{-}Se)_2$ substrate (*Supplementary Table 6*).

Supplementary Table 6. SET energy barriers and parameters used in their calculation. Sec- and perF- prefixes indicate *N*-Boc-L-Sec-O*t*Bu diselenide and $(C_6F_5\text{-}CH_2\text{-}Se)_2$ derivatives, respectively.

SET process	ΔG_r (DFT) (kcal·mol^{-1})	a_1 (Å)	a_2 (Å)	R (Å)	λ_0 (kcal·mol^{-1})	$\Delta G_0^{\ddagger}$ (eq. 6) (kcal·mol^{-1})	$\Delta G_{SET}^{\ddagger}$ (eq. 1) (kcal·mol^{-1})
**5*** + **I** → **5**$^{\cdot-}$ + **I**$^{\cdot+}$	-8.0	8.28 (**5***)	3.75 (**I**)	12.03	19.3	4.8	**2.5**
**Sec-III·** + **5**$^{\cdot-}$ → **Sec-III**$^{-}$ + **5**	-37.8	8.28 (**5***)	6.20 (**Sec-III·**)	14.48	12.6	3.1	**15.4**
**perF-III·** + **5**$^{\cdot-}$ → **perF-III**$^{-}$ + **5**	-39.3	8.28 (**5***)	4.99 (**perF-III·**)	13.27	14.9	3.7	**12.3**

In this approach, the reorganization parameter λ is assumed to be the same in the forward and backward reactions (assuming two parabolas having the same curvature, *Supplementary Figure 62A*). However, this is not always the most precise treatment and averaging multiple λ values

might increase the agreement with experimental results as seen previously (*Supplementary Figure 62B*).[50]

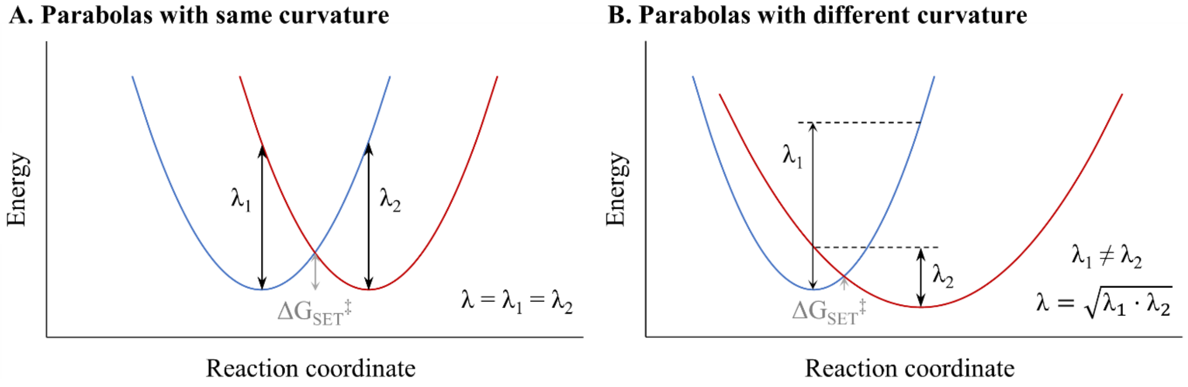

Supplementary Figure 62. Representation of pairs of parabolas with **A)** same and **B)** different curvature.

Vaissier method

In this method, DFT calculations (B3PW91-GD3(BJ)/6-31+G(d,p) level) are employed to measure λ_i (inner reorganization energies) and λ_0 (solvent reorganization energy). The total λ values ($\lambda_i + \lambda_0$) are obtained with single point energy calculations (instead of Gibbs free energy) of the reagents into the geometries of the products in non-equilibrium solvent phase. In order to generate meaningful results about solvent reorganization, the calculations when changing electronic states require the NonEq=write (original state) and NonEq=read (final state) keywords in Gaussian 16 to store and read the solvent cages (using Link 1 jobs). An example of input file is given below. For example, in the SET:

$$\mathbf{5^* + I \rightarrow 5^{\cdot -} + I^{\cdot +}}$$

$\lambda_{i,1}$ is obtained through four single-point energy calculations in gas phase:

Initial substrates before SET
(1) **5*** in its geometry, with multiplicity = 3, charge = 1 and using gas phase
(2) **I** in its geometry, with multiplicity = 1, charge = 0 and using gas
Initial substrates after SET
(3) **5*** in the geometry of $\mathbf{5}^{\cdot -}$, with multiplicity = 3, charge = 1 and using gas phase
(4) **I** in the geometry of $\mathbf{I}^{\cdot +}$, with multiplicity = 1, charge = 0 and using gas phase

Analogously, $\lambda_{i,2}$ is obtained with:

Products before SET

(1) **5**$^{\cdot-}$ in its geometry, with multiplicity = 2, charge = 0 and using gas phase

(2) **I**$^{\cdot+}$ in its geometry, with multiplicity = 2, charge = 1 and using gas phase

Products after SET

(3) **5**$^{\cdot-}$ in the geometry of **5***, with multiplicity = 2, charge = 0 and using gas phase

(4) **I**$^{\cdot+}$ in the geometry of **I**, with multiplicity = 2, charge = 1 and using gas phase

The final λ values ($\lambda_0 + \lambda_i$) and SET barriers are calculated in *Supplementary Tables 7* and *8*, respectively. It is worth to mention that, in all the cases, the contribution from λ_i is significant compared to λ_0.

Supplementary Table 7. SET energy barriers and parameters used in their calculation. Sec- and perF- prefixes indicate *N*-Boc-L-Sec-O*t*Bu diselenide and $(C_6F_5\text{-}CH_2\text{-}Se)_2$ derivatives, respectively.

SET process	ΔG_r (DFT) (kcal·mol^{-1})	λ_0 (kcal·mol^{-1})	$\lambda_{i,1}$ (kcal·mol^{-1})	$\lambda_{i,2}$ (kcal·mol^{-1})	λ_1 (kcal·mol^{-1})	λ_2 (kcal·mol^{-1})
**5*** + **I** → **5**$^{\cdot-}$ + **I**$^{\cdot+}$	-8.0	19.3	14.6	14.8	33.9	34.1
**Sec-III·** + **5**$^{\cdot-}$ → **Sec-III**$^{-}$ + **5**	-37.8	12.6	8.4	7.8	21.0	20.4
**perF-III·** + **5**$^{\cdot-}$ → **perF-III**$^{-}$ + **5**	-39.3	14.9	4.2	5.6	19.1	20.5

Supplementary Table 8. SET energy barriers and parameters used in their calculation. Sec- and perF- prefixes indicate *N*-Boc-L-Sec-O*t*Bu diselenide and $(C_6F_5\text{-}CH_2\text{-}Se)_2$ derivatives, respectively.

SET process	ΔG_r (DFT) (kcal·mol^{-1})	λ_1 (kcal·mol^{-1})	λ_2 (kcal·mol^{-1})	λ_{av} (kcal·mol^{-1})	$\Delta G_0^‡$ (eq. 2) (kcal·mol^{-1})	$\Delta G_{SET}^‡$ (eq. 1) (kcal·mol^{-1})
**5*** + **I** → **5**$^{\cdot-}$ + **I**$^{\cdot+}$	-8.0	33.9	34.1	34.0	8.5	**5.0**
**Sec-III·** + **5**$^{\cdot-}$ → **Sec-III**$^{-}$ + **5**	-37.8	21.0	20.4	20.7	5.2	**3.5**
**perF-III·** + **5**$^{\cdot-}$ → **perF-III**$^{-}$ + **5**	-39.3	19.1	20.5	19.8	5.0	**4.7**

Berkeley Madonna Kinetic Simulations Using $(C_6F_5\text{-}CH_2\text{-}Se)_2$

The inputs employed to run Berkeley Madonna[51] kinetic simulations are shown below. A slight adjustment in the energy value of **TS-IV** leads to a theoretical model that reproduces experimental yields quantitatively, as seen in previous studies.[52,53] The energy changes are included as comments in the scripts below. In all the cases, the energy variations applied were considerably small (-0.4 kcal·mol^{-1} and +1.2 kcal·mol^{-1} in **TS-IV** for the photocatalyzed and uncatalyzed reactions, respectively).

Photocatalyzed reaction

Experimental result: 95% in 1 min

*A high concentration of photons is included to make the initial Ir excitation very fast as seen experimentally.

METHOD RK4; RK4 is not used, the method is Rosenbrock (stiff), but it has to be specified manually when running the program

STARTTIME = 0
STOPTIME=100; in seconds
DTMIN = 1E-12

T = 298.15 ; reaction temperature
kb = 1.38064852*10^-23 ; Boltzmann constant in seconds
h = 6.62607004*10^-34 ; Planck constant
Na = 6.02214086*10^23 ; Avogadro constant
kcal_to_J = -4184 ; convert kcal/mol to J/mol
kT = kb * T
RT = Na * kb * T
prefac = kb * T / h ; Erying pre-exponential factor in seconds
visc = 3.44*10^-4 ; solvent viscosity of MeCN at 298.15K in Pa s-1 (from the Dortmund Data Bank, http://www.ddbst.com/en/EED/PCP/VIS_C3.php)

; initial concentrations in mmol
INIT Photon = 1000000000000000000000000000000000000000000; we used a big concentration of protons just to emulate experimental observation (very fast **Ir3** (**5**) --> **Ir3-T1** (**5***) excitation)
INIT Ir_cat_3 = 0.0005 ; Ir catalyst with oxidation state = +3, calc name = **Ir3** (**5**)
INIT Ir_cat_3_T1 = 0 ; Ir catalyst with oxidation state = +3, triplet state, calc name = **Ir3-T1** (**5***)
INIT Ir_cat_2 = 0 ; Ir catalyst with oxidation state = +2, calc name = **Ir2** ($\mathbf{5}^{\cdot-}$)
INIT PTA = 0.02 ; PTA phosphine, calc name = PTA (**I**)
INIT PTA_rad = 0 ; PTA phosphine radical, calc name = **PTA-rad** ($\mathbf{I}^{\cdot+}$)
INIT diSeBn = 0.005 ; initial diselenide, calc name = **perF-diSeBn**
INIT PTA_SeBn = 0 ; PTA-SeBn intermediate, calc name = **perF-PTA-SeBn** ($\mathbf{II}^{+}$)
INIT SeBn_rad = 0 ; SeBn radical, calc name = **perF-SeBn-rad** ($\mathbf{III}^{\cdot}$)
INIT SeBn_anion = 0 ; SeBn anion, calc name = **perF-SeBn-anion** ($\mathbf{III}^{-}$)
INIT Se_PTA = 0 ; PTA=Se byproduct, calc name = **SePTA** (**VI**)
INIT Product = 0 ; BnSeBn product, calc name = **perF-BnSeBn** (**V**)

; calculated relative G energies of the reaction steps in kcal/mol
G_Ir_3_initial = 0
G_PTA_diSeBn = 56.6
G_SET_PTA_Ir_cat_3_T1 = 64.2
G_PTA_rad_diSeBn = 48.6
G_PTA_SeBn_SeBn_rad = 46.4
G_SET_SeBn_rad_Ir_cat_2 = 49.5
G_PTA_SeBn_SeBn_anion = 7.1
G_TS_IV = 19.0 ; -0.4 kcal/mol compared to original DFT result
G_TS_VIII = 11.9

G_Se_PTA_Product = -15.7

; automated dG of TS in kcal/mol
G0f = G_PTA_diSeBn - G_Ir_3_initial
G0r = 0 ; diffusion controlled
G1f = G_SET_PTA_Ir_cat_3_T1 - G_PTA_diSeBn
G1r = G_SET_PTA_Ir_cat_3_T1 - G_PTA_rad_diSeBn
G2f = 0 ; diffusion controlled
G2r = G_PTA_rad_diSeBn - G_PTA_SeBn_SeBn_rad ; diffusion controlled
G3f = G_SET_SeBn_rad_Ir_cat_2 - G_PTA_SeBn_SeBn_rad
G3r = G_SET_SeBn_rad_Ir_cat_2 - G_PTA_SeBn_SeBn_anion
G4f = G_TS_VIII - G_PTA_SeBn_SeBn_anion
G4r = G_TS_VIII - G_Ir_3_initial
G5f = G_TS_IV - G_PTA_SeBn_SeBn_anion
G5r = G_TS_IV - G_Se_PTA_Product

{ 0: Ir_cat_3 + Photon <--> Ir_cat_3_T1 } ; photoexcitation, very fast process (diffusion controlled)
RXN0 = K0f*Ir_cat_3*Photon - K0r*Ir_cat_3_T1
K0f = (8*RT)/(3*visc)*exp(G0f*kcal_to_J/RT)
K0r = (8*RT)/(3*visc)*exp(G0r*kcal_to_J/RT)
d/dt(Ir_cat_3) = -RXN0+RXN3
d/dt(Photon) = -RXN0
d/dt(Ir_cat_3_T1) = -RXN1+RXN0

{ 1: Ir_cat_3_T1 + PTA <--> Ir_cat_2 + PTA_rad } ; SET process
RXN1 = K1f*Ir_cat_3_T1*PTA - K1r*Ir_cat_2*PTA_rad
K1f = prefac*exp(G1f*kcal_to_J/RT)
K1r = prefac*exp(G1r*kcal_to_J/RT)
d/dt(PTA) = -RXN1+RXN4
d/dt(Ir_cat_2) = +RXN1-RXN3
d/dt(PTA_rad) = +RXN1-RXN2

{ 2: PTA_rad + diSeBn <--> PTA_SeBn + SeBn_rad } ; PTA radical cleaves the initial diselenium compound forming a SeBn radical, very fast process (diffusion controlled)
RXN2 = K2f*PTA_rad*diSeBn - K2r*PTA_SeBn*SeBn_rad
K2f = (8*RT)/(3*visc)*exp(G2f*kcal_to_J/RT)
K2r = (8*RT)/(3*visc)*exp(G2r*kcal_to_J/RT)
d/dt(diSeBn) = -RXN2+RXN4
d/dt(PTA_SeBn) = +RXN2-RXN4-RXN5
d/dt(SeBn_rad) = +RXN2-RXN3

{ 3: SeBn_rad + Ir_cat_2 <--> SeBn_anion + Ir_cat_3 } ; SET process
RXN3 = K3f*SeBn_rad*Ir_cat_2 - K3r*SeBn_anion*Ir_cat_3
K3f = prefac*exp(G3f*kcal_to_J/RT)
K3r = prefac*exp(G3r*kcal_to_J/RT)
d/dt(SeBn_anion) = +RXN3-RXN4-RXN5

{ 4: PTA_SeBn + SeBn_anion <--> PTA + diSeBn } ; inhibitory retro-reaction that goes back to the starting material (in the reverse direction, this is also a pathway that can trigger SeBn_anion and product formation without photoexcitation, but it is considerably slower than the main photocatalytic pathway)
RXN4 = K4f*PTA_SeBn*SeBn_anion - K4r*PTA*diSeBn
K4f = prefac*exp(G4f*kcal_to_J/RT)
K4r = prefac*exp(G4r*kcal_to_J/RT)

{ 5: PTA_SeBn + SeBn_anion <--> Product + Se_PTA } ; productive reaction leading to the product and Se=PTA
RXN5 = K5f*PTA_SeBn*SeBn_anion - K5r*Product*Se_PTA
K5f = prefac*exp(G5f*kcal_to_J/RT)
K5r = prefac*exp(G5r*kcal_to_J/RT)
d/dt(Product) = +RXN5
d/dt(Se_PTA) = +RXN5

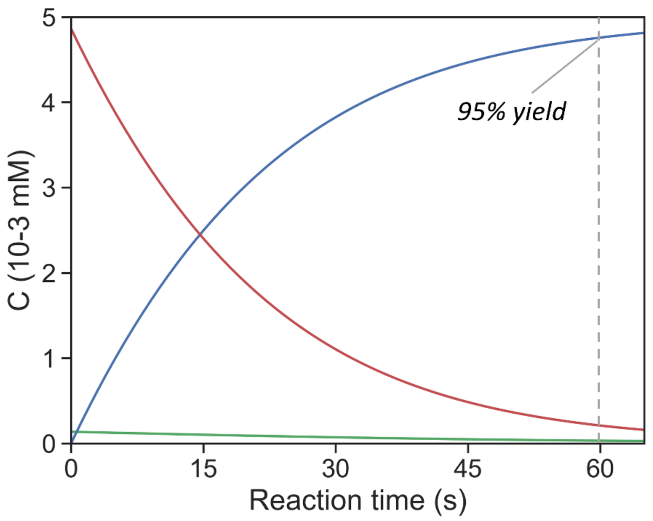

Supplementary Figure 63. Simulated concentration profile of the photocatalytic diselenide contraction of the $(C_6F_5\text{-}CH_2\text{-}Se)_2$ substrate. Concentrations: blue line = product; red line = starting $(C_6F_5\text{-}CH_2\text{-}Se)_2$; green line = PTA-SeBn ($\mathbf{II}^+$) intermediate.

Uncatalyzed reaction

Experimental result: 27% in 1 h

METHOD RK4; RK4 is not used, the method is Rosenbrock (stiff), but it has to be specified manually when running the program

STARTTIME = 0
STOPTIME=4000; in seconds
DTMIN = 1E-12

T	= 298.15 ; reaction temperature
kb	= 1.38064852*10^-23 ; Boltzmann constant in seconds
h	= 6.62607004*10^-34 ; Planck constant
Na	= 6.02214086*10^23 ; Avogadro constant
kcal_to_J	= -4184 ; convert kcal/mol to J/mol
kT	= kb * T
RT	= Na * kb * T
prefac	= kb * T / h ; Erying pre-exponential factor in seconds

visc = 3.44*10^-4 ; solvent viscosity of MeCN at 298.15K in Pa s-1 (from the Dortmund Data Bank, http://www.ddbst.com/en/EED/PCP/VIS_C3.php)

; initial concentrations in mmol
INIT PTA = 0.02 ; PTA phosphine, calc name = PTA (**I**)
INIT diSeBn = 0.005 ; initial diselenide, calc name = **perF-diSeBn**
INIT PTA_SeBn = 0 ; PTA-SeBn intermediate, calc name = **perF-PTA-SeBn** (**II**$^{+}$)
INIT SeBn_anion = 0 ; SeBn anion, calc name = **perF-SeBn-anion** (**III**$^{-}$)
INIT Se_PTA = 0 ; PTA=Se byproduct, calc name = **SePTA** (**VI**)
INIT Product = 0 ; BnSeBn product, calc name = **perF-BnSeBn** (**V**)

; relative G energies of the reaction steps in kcal/mol
G_Initial_reagents = 0
G_PTA_SeBn_SeBn_anion = 7.1
G_TS_VIII = 11.9
G_TS_IV = 20.6 ; +1.2 kcal/mol compared to original DFT result
G_Se_PTA_Product = -15.7

; automated dG of TS in kcal/mol
G0f = G_TS_VIII - G_Initial_reagents
G0r = G_TS_VIII - G_PTA_SeBn_SeBn_anion
G1f = G_TS_IV - G_PTA_SeBn_SeBn_anion
G1r = G_TS_IV - G_Se_PTA_Product

{ 0: PTA + diSeBn <--> PTA_SeBn + SeBn_anion } ; PTA cleaves the initial diselenium compound forming a SeBn anion
RXN0 = K0f*PTA*diSeBn - K0r*PTA_SeBn*SeBn_anion
K0f = prefac*exp(G0f*kcal_to_J/RT)
K0r = prefac*exp(G0r*kcal_to_J/RT)
d/dt(PTA) = -RXN0
d/dt(diSeBn) = -RXN0
d/dt(PTA_SeBn) = +RXN0-RXN1
d/dt(SeBn_anion) = +RXN0-RXN1

{ 1: PTA_SeBn + SeBn_anion <--> Product + Se_PTA } ; productive reaction leading to the product and Se=PTA
RXN1 = K1f*PTA_SeBn*SeBn_anion - K1r*Product*Se_PTA
K1f = prefac*exp(G1f*kcal_to_J/RT)
K1r = prefac*exp(G1r*kcal_to_J/RT)
d/dt(Product) = +RXN1
d/dt(Se_PTA) = +RXN1

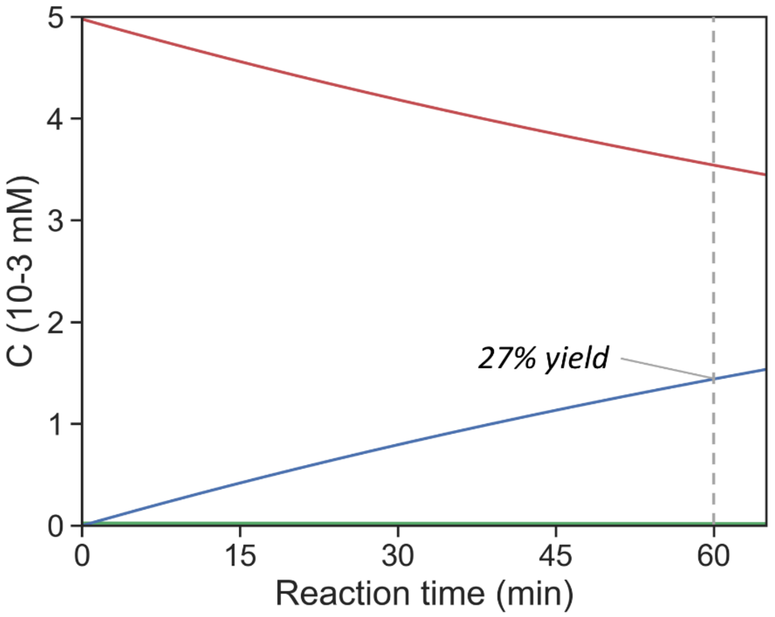

Supplementary Figure 64. Simulated concentration profile of the uncatalyzed diselenide contraction of the $(C_6F_5\text{-}CH_2\text{-}Se)_2$ substrate. Concentrations: blue line = product; red line = starting $(C_6F_5\text{-}CH_2\text{-}Se)_2$; green line = PTA-SeBn (**II**$^{+}$) intermediate.

Molecular Coordinates

An xyz file containing all the geometries studied is provided as a separate Supplementary Information file. The creation of the xyz files was automated with the *GoodVibes* software (option “--xyz”).

Optimization of PDC Functionalization on a Model Selenopeptide System: Mucin-1 (MUC1) (U11)

Synthesis of Model Selenopeptide MUC1 (U11):

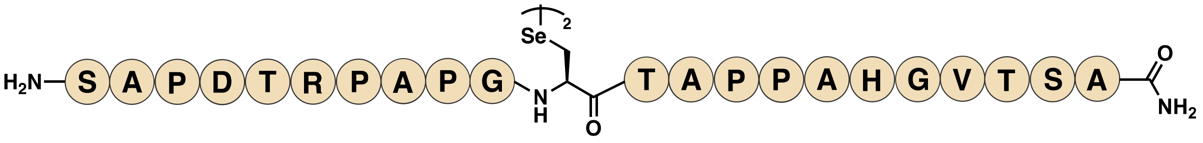

[MUC1 (U11)]$_2$ Diselenide

Fmoc-Ala-OH was loaded onto Rink amide resin and the peptide was extended (100 µmol) using standard Fmoc-SPPS techniques as outlined in the general methods section. Fmoc-Sec(Bn)-OH was coupled manually using DIC/Oxyma coupling conditions [Fmoc-Sec(Bn)-OH (1.2 eq.), DIC (1.2 eq.) and Oxyma (3 eq.) in DMF (0.1 M) for 16 h at room temperature]. Removal of the acid labile protecting groups, with concomitant cleavage from resin, was achieved *via* treatment with a solution of TFA/iPr$_3$SiH/ /H_2O (90:5:5 v/v/v, 5 mL) for 2 h at room temperature. After filtering off the resin, the deprotection solution was concentrated under nitrogen flow and the crude peptide was precipitated from ice-cold Et_2O. The crude peptide (50 µmol) was dissolved in a 2 vol% solution of thioanisole in TFA. DTNP (20 eq.) was added and the mixture heated at 50 ºC for 1 h and then room temperature for 1 h. The solution was then concentrated under nitrogen flow and the crude peptide was precipitated from ice-cold Et_2O. The crude peptide (100 µmol) was treated with sodium ascorbate and TCEP and immediately purified by RP-HPLC (C18 X-bridge column, 5 µm, 30 × 150 mm, 0 to 30%B over 50 min, 0.1 vol% TFA) to afford the pure target peptide diselenide as a white fluffy solid after lyophilization (24.2 mg, 11% yield).

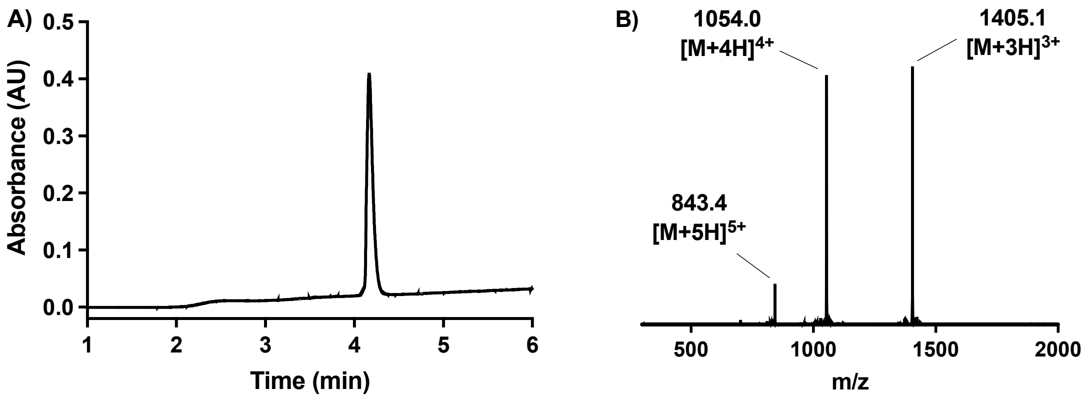

Supplementary Figure 65. A) Analytical HPLC trace of purified [MUC1 (U11)]$_2$ diselenide, R_t 4.1 min (0 to 30% B over 5 min, 0.1 vol% TFA, λ = 214 nm); **B)** ESI-MS of pure [MUC1 (U11)]$_2$ diselenide; Calculated Mass: $[M+3H]^{3+}$: 1405.1; $[M+4H]^{4+}$: 1054.1; $[M+5H]^{5+}$: 843.5. Mass Found (ESI+) 1405.1 $[M+3H]^{3+}$; 1054.0 $[M+4H]^{4+}$; 843.4 $[M+5H]^{5+}$. ESI-MS data was collected over the entire gradient and wash cycle of the UPLC-MS.

Small Molecule Equivalents Screen: PTA (4) Scaling = 4 Equiv.

[MUC1 (U11)]$_2$ diselenide (1.22 mg, 0.250 μmol) was dissolved in 1:1 v/v H_2O:MeCN (50 μL) containing $[Ir(dF(CF_3)ppy)_2(dtbpy)]PF_6$ (**5**) (0.1 μmol mL^{-1}) for a photocatalyst loading of 2 mol% and a concentration of [MUC1 (U11)]$_2$ diselenide of 5 mM. This solution was then used to dissolve [Se-PEG$_6$]$_2$ diselenide (**16**) in the following quantities:

1. 0.172 mg, 0.250 μmol, 1 eq.
2. 0.344 mg, 0.500 μmol, 2 eq.
3. 0.689 mg, 1.00 μmol, 4 eq.
4. 1.378 mg, 2.00 μmol, 8 eq.

The solution was then equilibrated for 30 min at room temperature. After incubation, the solution was used to dissolve PTA (**4**) in the following associated quantities (quoted equivalents are relative to [MUC1 (U11)]$_2$ diselenide) such as to maintain four equivalents of **4** relative to total diselenide ([MUC1 (U11)]$_2$ diselenide + **16**):

1. 0.314 mg, 2.00 μmol, 8 eq.
2. 0.471 mg, 3.00 μmol, 12 eq.
3. 0.785 mg, 5.00 μmol, 20 eq.
4. 1.413 mg, 9.00 μmol, 36 eq.

The resulting reaction mixture was irradiated with 450 nm light (PennOC M1 photoreactor) for 5 min. At 0, 0.5, 1, 2 and 5 min, aliquots (5 μL) of the reaction mixtures were taken and diluted 10-fold with H_2O (0.1 vol% TFA) (45 μL). Reaction analysis was performed on these aliquots using UPLC-HRMS on gradients of 0-100% B (Waters Acquity BEH300 C18 1.7 μm, 2.1 x 50 mm, λ = 214 nm). Conversions were calculated based on the integration of all peptide related masses in the HRMS TIC.

See next page for analytical data.

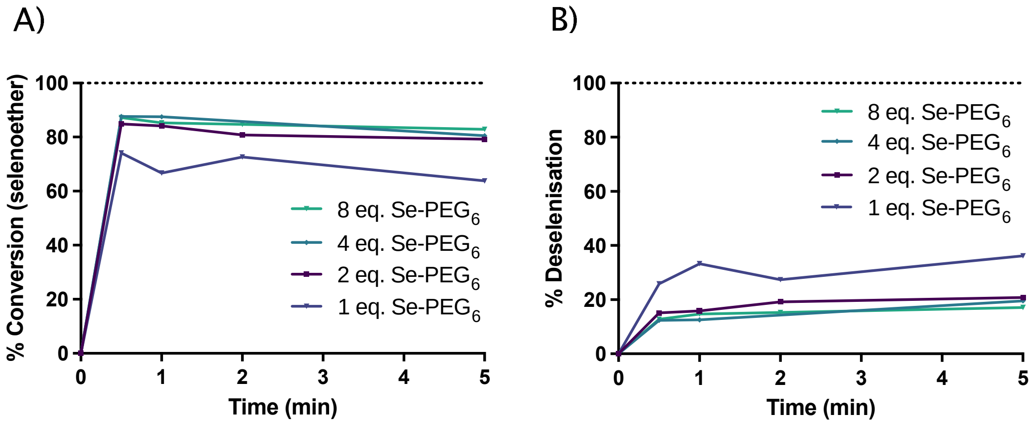

Supplementary Figure 66. Optimization of the functionalization of [MUC1 (U11)]$_2$ diselenide with [Se-PEG$_6$]$_2$ diselenide (**16**) *via* PDC employing PTA (**4**) at 4 eq. relative to total diselenide. **A)** Conversion to PEG$_6$-MUC1 (U11) asymmetric selenoether at differing concentrations of [Se-PEG$_6$]$_2$ diselenide (**16**) over 5 min of irradiation with 450 nm light. **B)** Associated conversion to deselenized MUC (A11) by-product at differing concentrations of [Se-PEG$_6$]$_2$ diselenide (**16**) over 5 min of irradiation at 450 nm. Conversions calculated through integration of all peptide-related TIC peaks.

Small Molecule Equivalents Screen: PTA (4) Scaling = 2 Equiv.

[MUC1 (U11)]$_2$ diselenide (0.61 mg, 0.125 μmol) was dissolved in 1:1 v/v H_2O:MeCN (25 μL) containing [Ir(dF(CF_3)ppy)$_2$(dtbpy)]PF_6 (**5**) (0.1 μmol mL^{-1}) for a photocatalyst loading of 2 mol% and a concentration of [MUC1 (U11)]$_2$ diselenide of 5 mM. This solution was then used to solvate [Se-PEG$_6$]$_2$ diselenide (**16**) in the following quantities:

1. 0.086 mg, 0.125 μmol, 1 eq.
2. 0.172 mg, 0.250 μmol, 2 eq.
3. 0.345 mg, 0.500 μmol, 4 eq.
4. 0.689 mg, 1.00 μmol, 8 eq.

The solution was then equilibrated for 30 min at room temperature. After incubation, the solution was used to solvate PTA (**4**) in the following associated quantities (quoted equivalents are relative to [MUC1 (U11)]$_2$ diselenide diselenide) such as to maintain four equivalents of **4** relative to total diselenide ([MUC1 (U11)]$_2$ diselenide + **16**):

1. 0.079 mg, 0.500 μmol, 4 eq.
2. 0.118 mg, 0.750 μmol, 6 eq.
3. 0.197 mg, 1.25 μmol, 10 eq.
4. 0.354 mg, 2.25 μmol, 18 eq.

The resulting reaction mixture was irradiated with 450 nm light (PennOC M1 photoreactor) for 5 min. At 0, 0.5, 1, 2 and 5 min, aliquots (2.5 μL) of the reaction mixtures were taken and diluted 10-fold with H_2O (0.1 vol% TFA) (22.5 μL). Reaction analysis was performed on these aliquots using UPLC-HRMS on gradients of 0-100% B over 5 min (Waters Acquity BEH300 C18 1.7 μm, 2.1 x 50 mm, λ = 214 nm). Conversions were calculated based on the integration of all peptide related masses in the MS TIC.

See next page for analytical data.

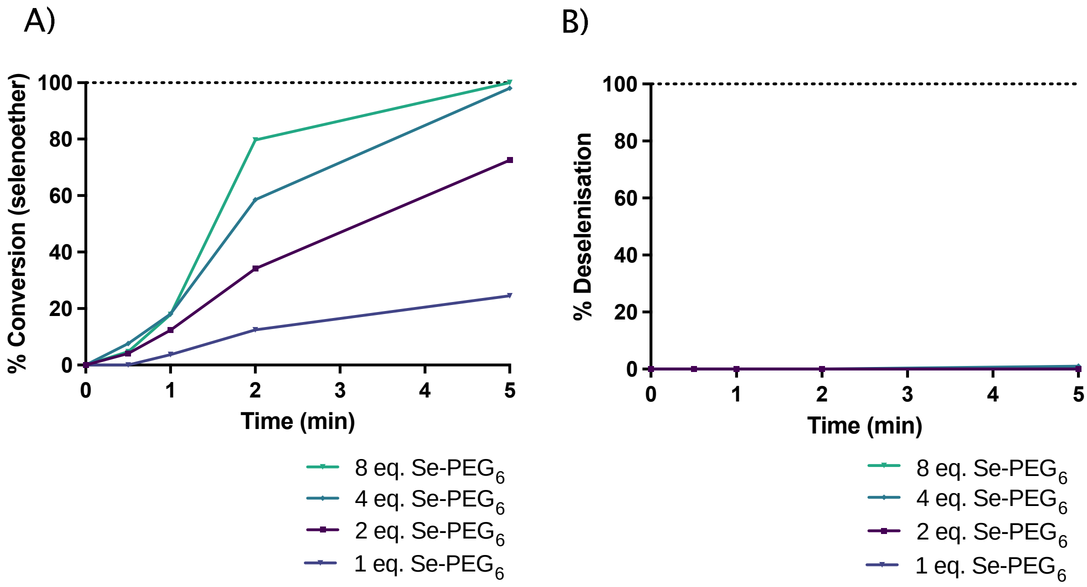

Supplementary Figure 67. Optimization of the functionalization of [MUC1 (U11)]$_2$ diselenide with [Se-PEG$_6$]$_2$ diselenide (**16**) *via* PDC employing PTA (**4**) at 2 eq. relative to total diselenide. **A)** Conversion to PEG$_6$-MUC1 (U11) asymmetric selenoether at differing concentrations of [Se-PEG$_6$]$_2$ diselenide (**16**) over 5 min of irradiation with 450 nm light. **B)** Associated conversion to deselenized MUC (A11) by-product at differing concentrations of [Se-PEG$_6$]$_2$ diselenide (**16**) over 5 min of irradiation at 450 nm. Conversions calculated through integration of all peptide-related TIC peaks.

Solvent Screen

[MUC1 (U11)]$_2$ diselenide (0.61 mg, 0.125 μmol) was dissolved in 25 μL of one of the following solvent systems containing [Ir(dF(CF$_3$)ppy)$_2$(dtbpy)]PF$_6$ (**5**) (0.1 μmol mL^{-1}) for a photocatalyst loading of 2 mol%:

1. 1:1 v/v H_2O:MeCN
2. 1:1 v/v denaturing buffer (6 M Gn.HCl, 0.02 M Na_2HPO_4, pH 7.0):MeCN
3. 1:1 v/v TBS (50 mM Tris, 300 mM NaCl, pH 8.0):MeCN
4. 7:3 v/v H_2O:trifluoroethanol (TFE)
5. 7:3 v/v denaturing buffer (6 M Gn.HCl, 0.02 M Na_2HPO_4, pH 7.0):TFE
6. 7:3 v/v TBS (50 mM Tris, 300 mM NaCl, pH 8.0):TFE

All solutions were further diluted with 225 μL of their respective solvent systems to bring the concentration of [MUC1 (U11)]$_2$ diselenide to 0.5 mM. These solutions were then used to dissolve [Se-PEG$_6$]$_2$ diselenide (**16**) (0.689 mg, 1.00 μmol, 8 eq.) and PTA (**4**) (0.354 mg, 2.25 μmol, 18 eq.) and the resulting reaction mixtures were irradiated with 450 nm light (PennOC M1 photoreactor) for 5 min. At 0, 2.5 and 5 min, aliquots of the reaction mixtures were taken directly analyzed by UPLC-HRMS on gradients of 0-100% B over 5 min (Waters Acquity BEH300 C18 1.7 μm, 2.1 x 50 mm, λ = 214 nm). Conversions were calculated based on the integration of all peptide related masses in the MS TIC.

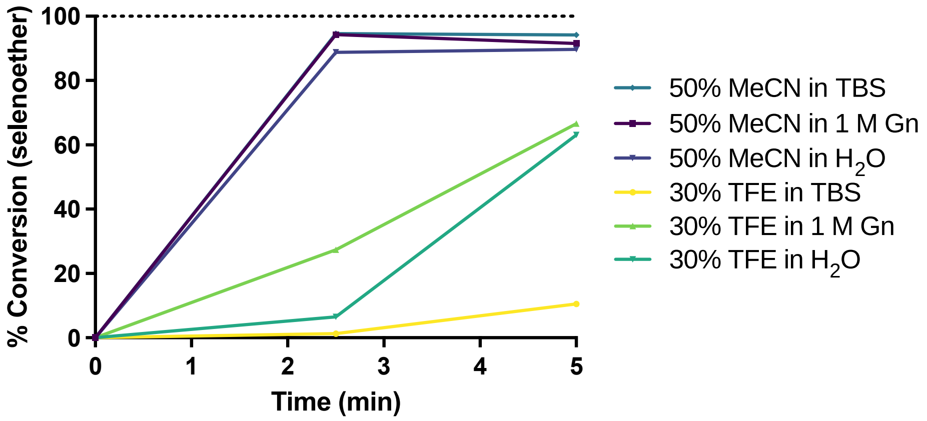

Supplementary Figure 68. Solvent compatibility screen for the functionalisation of [MUC1 (U11)]$_2$ diselenide with [Se-PEG$_6$]$_2$ diselenide (**16**) *via* photocatalytic diselenide contraction employing PTA (**4**) at 2 eq. relative to

diselenide and 5 min irradiation with 450 nm light. Conversions calculated through integration of all peptide-related TIC peaks.

Concentration Screen

[MUC1 (U11)]$_2$ diselenide (1.22 mg, 0.250 μmol) was dissolved in 50 μL of 1:1 v/v H_2O:MeCN containing [Ir(dF(CF_3)ppy)$_2$(dtbpy)]PF_6 (**5**) (0.1 μmol mL^{-1}) for a photocatalyst loading of 2 mol%. Solutions were used to solvate [Se-PEG$_6$]$_2$ diselenide (**16**) (1.378 mg, 2.00 μmol, 8 eq.) and PTA (**4**) (0.708 mg, 4.50 μmol, 18 eq.).

Aliquots (7.5 μL) of this solution were split into six different reaction vessels and then further diluted with an amount of 1:1 v/v H_2O:MeCN for reduced concentrations of [MUC1 (U11)]$_2$ diselenide as follows:

1. 0 μL ([MUC1 (U11)]$_2$ diselenide = 5.0 mM)
2. 7.5 μL ([MUC1 (U11)]$_2$ diselenide = 2.5 mM)
3. 30.0 μL ([MUC1 (U11)]$_2$ diselenide = 1.0 mM)
4. 67.5 μL ([MUC1 (U11)]$_2$ diselenide = 0.50 mM)
5. 142.5 μL ([MUC1 (U11)]$_2$ diselenide = 0.25 mM)
6. 367.5 μL ([MUC1 (U11)]$_2$ diselenide = 0.10 mM)

The resulting reaction mixtures were irradiated with 450 nm light (PennOC M1 photoreactor) for 5 min. At 0, 2.5 and 5 min, aliquots of the reaction mixtures were taken and directly analyzed by UPLC-HRMS using a gradient of 0-100% B over 5 min (Waters Acquity BEH300 1.7 μm, 2.1 x 50 mm (C18), λ = 214 nm). Conversions were calculated based on the integration of all peptide related masses in the MS TIC.

See next page for analytical data.

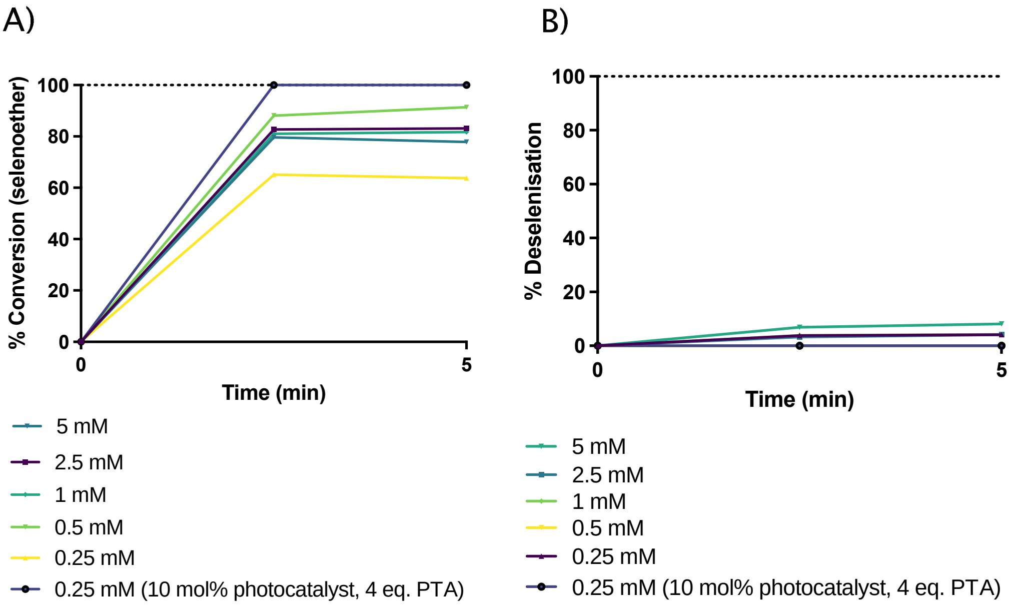

Supplementary Figure 69. Concentration screen for the functionalisation of [MUC1 (U11)]$_2$ diselenide with [Se-PEG$_6$]$_2$ diselenide (**16**) *via* photocatalytic diselenide contraction employing PTA (**4**) at 2 eq. relative to diselenide. **A)** Conversion to PEG$_6$-MUC1 (U11) asymmetric selenoether at various [MUC1 (U11)]$_2$ diselenide concentrations over 5 min of irradiation with 450 nm light. **B)** Associated conversion to deselenized MUC (A11) by-product at various [MUC1 (U11)]$_2$ concentrations over 5 min of irradiation at 450 nm. Conversions calculated through integration of all peptide-related TIC peaks.

Control Reactions

'No Phosphine'

[MUC1 (U11)]$_2$ diselenide (0.61 mg, 0.125 μmol) was dissolved in 1:1 v/ H_2O:MeCN (25 μL) containing [Ir(dF(CF_3)ppy)$_2$(dtbpy)]PF_6 (**5**) (0.1 μmol mL^{-1}) for a photocatalyst loading of 2 mol%. The solution was further diluted with 1:1 v/v H_2O:MeCN (225 μL) to achieve a concentration of [MUC1 (U11)]$_2$ diselenide of 0.5 mM. This solution was then used to dissolve [Se-PEG$_6$]$_2$ diselenide (**16**) (0.69 mg, 1.00 μmol, 8 eq.) and the resulting reaction mixture was irradiated with 450 nm light (PennOC M1 photoreactor) for 5 min. At 0 and 5 min, aliquots of the reaction mixtures were taken directly analyzed by UPLC-HRMS on gradients of 0-100% B over 5 min (Waters Acquity BEH300 1.7 μm, 2.1 x 50 mm (C18), λ = 214 nm).

'No Photocatalyst'

[MUC1 (U11)]$_2$ diselenide (0.61 mg, 0.125 μmol) was dissolved in 1:1 v/v H_2O:MeCN (250 μL) to achieve a concentration of 0.5 mM. This solution was then used to dissolve [Se-PEG$_6$]$_2$ diselenide (**16**) (0.69 mg, 1.00 μmol, 8 eq.) and PTA (**4**) (0.354 mg, 2.25 μmol, 18 eq.) and the resulting reaction mixture was irradiated with 450 nm light (PennOC M1 photoreactor) for 5 min. At 0 and 5 min, aliquots of the reaction mixtures were taken directly analyzed by UPLC-HRMS on gradients of 0-100% B over 5 min (Waters Acquity BEH300 1.7 μm, 2.1 x 50 mm (C18), λ = 214 nm).

'No [Se-PEG$_6$]$_2$'

[MUC1 (U11)]$_2$ diselenide (0.61 mg, 0.125 μmol) was dissolved in 1:1 v/v H_2O:MeCN (25 μL) containing [Ir(dF(CF_3)ppy)$_2$(dtbpy)]PF_6 (**5**) (0.1 μmol mL^{-1}) for a photocatalyst loading of 2 mol%. The solution was further diluted with 1:1 v/v H_2O:MeCN (225 μL) to achieve a concentration of [MUC1 (U11)]$_2$ diselenide of 0.5 mM. This solution was then used to dissolve PTA (**4**) (0.354 mg, 2.25 μmol, 18 eq.) and the resulting reaction mixture was irradiated with 450 nm light (PennOC M1 photoreactor) for 5 min. At 0 and 5 min, aliquots of the reaction mixtures were taken directly analyzed by UPLC-HRMS on gradients of 0-100% B over 5 min (Waters Acquity BEH300 1.7 μm, 2.1 x 50 mm (C18), λ = 214 nm).

As expected, no conversion of [MUC1 (U11)]2 diselenide to its asymmetric selenoether was observed in any of the control reactions. Only gradual conversion of [MUC1 (U11)]2 diselenide to its corresponding symmetric selenoether and deselenized by-product, MUC1 (A11), diselenide was observed in the case of the '*no [Se-PEG6]2*' control.

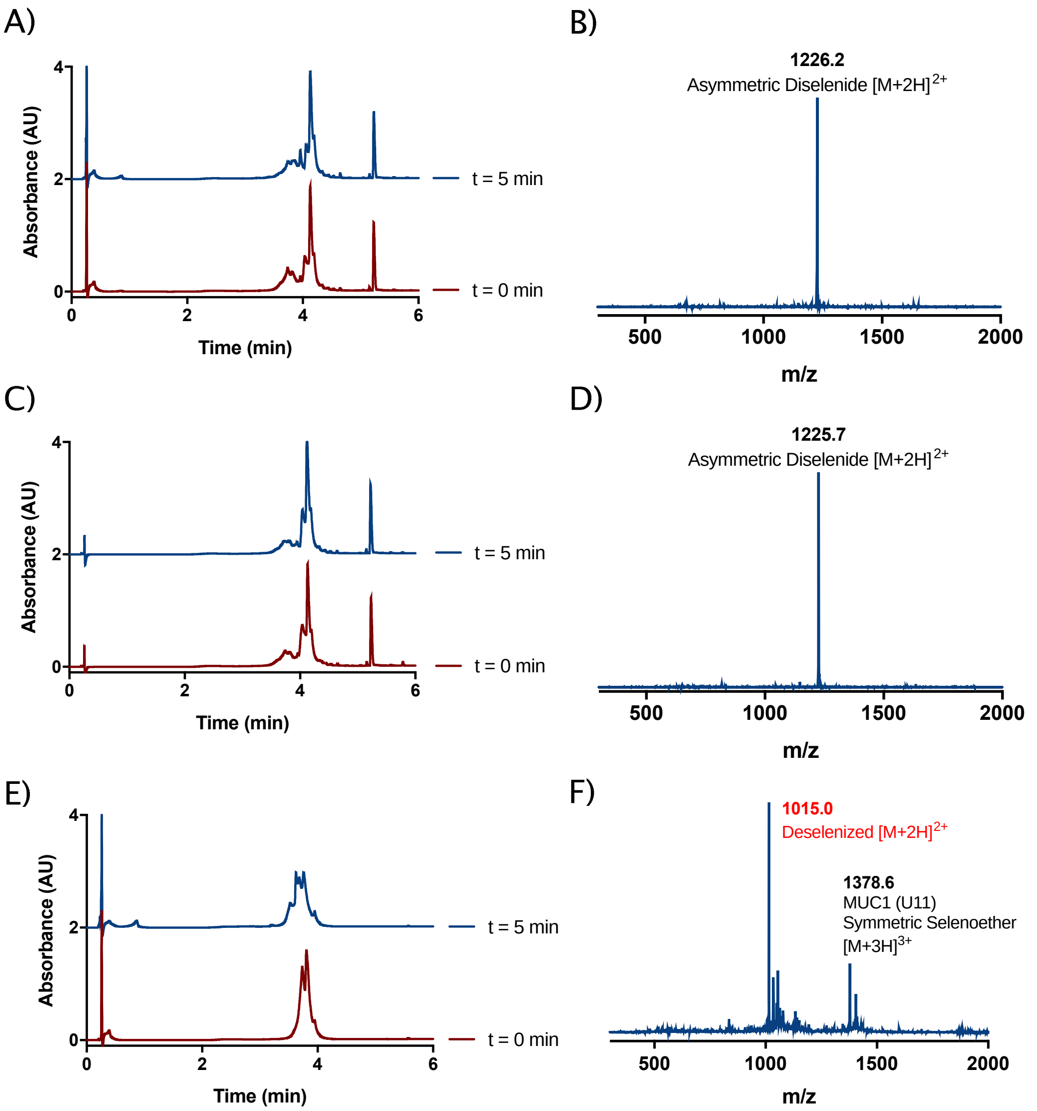

Supplementary Figure 70. Control reactions for the functionalization of [MUC1 (U11)]2 diselenide with [Se-PEG6]2 diselenide (**16**) employing PTA (**4**) at 2 eq. relative to diselenide at a peptide concentration of 5 mM in 1:1 v/v H_2O:MeCN as monitored by UPLC (0-40% B over 5 min, λ = 214 nm) and MS (ESI+). **A)** '*No phosphine*' control featuring removal of PTA (**4**). **B)** Associated MS (ESI+) spectrum for the '*no phosphine*' control. **C)** '*No photocatalyst*' control featuring removal of [Ir(dF(CF3)ppy)2(dtbpy)]PF6 (**5**). **D)** Associated MS (ESI+) spectrum for the '*no photocatalyst*' control. **E)** '*No [Se-PEG6]2*' control featuring removal of [Se-PEG6]2 (**16**). **F)** Associated MS (ESI+) spectrum for the '*No [Se-PEG6]2*' control.

Optimized PDC Functionalization of [MUC1 (U11)]$_2$ Diselenide

[MUC1 (U11)]$_2$ diselenide (0.61 mg, 0.125 μmol) was dissolved in 1:1 v/v H_2O:MeCN (25 μL) containing [Ir(dF(CF_3)ppy)$_2$(dtbpy)]PF_6 (**5**) (0.1 μmol mL^{-1}) for a photocatalyst loading of 2 mol% and then further diluted with 1:1 v/v H_2O:MeCN (225 μL) for a concentration of [MUC1 (U11)]$_2$ diselenide of 0.5 mM. This solution was then used to dissolve [Se-PEG$_6$]$_2$ diselenide (**16**) (0.689 mg, 1.00 μmol, 8 eq.), followed by PTA (**4**) (0.354 mg, 2.25 μmol, 18 eq.) such as to maintain 2 equivalents of **4** relative to total diselenide ([MUC1 (U11)]$_2$ diselenide + **16**). The reaction mixture was then split evenly into 13 aliquots of 20 μL and each of these aliquots was independently irradiated with 450 nm light (PennOC M1 photoreactor) for either 0, 10, 20, 30, 40, 50, 60, 70, 80, 90, 100, 110 or 120 s. After irradiation, each aliquot was directly analysed by UPLC-HRMS using a gradient of 0-100% B over 5 min (λ = 214 nm). Conversions were calculated based on the integration of all peptide related masses in the MS.

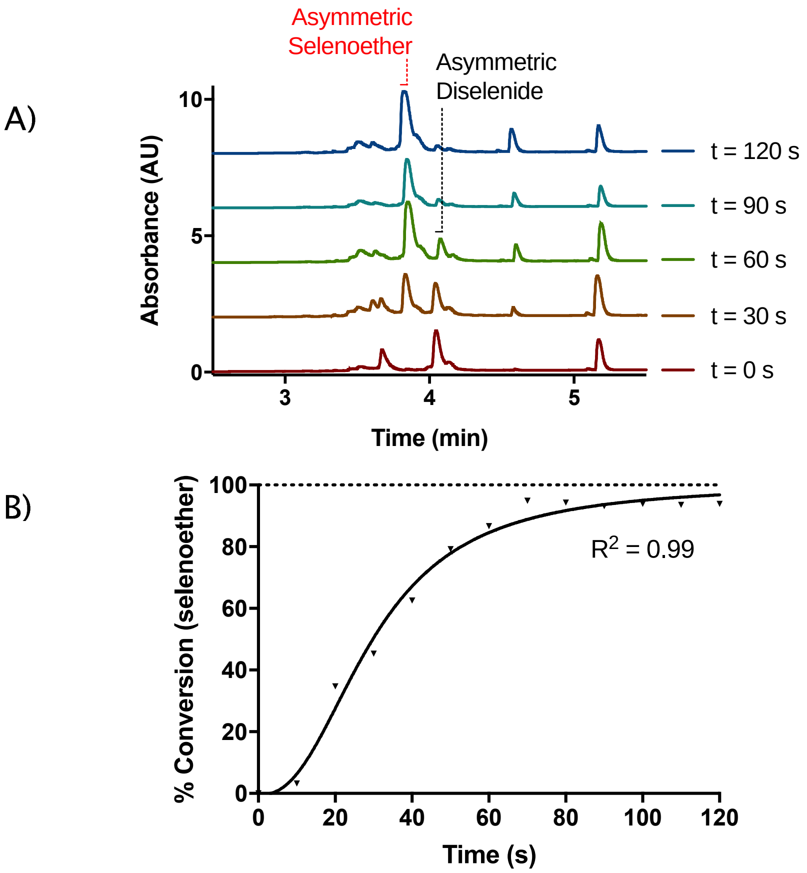

Supplementary Figure 71. A) Conversion of [MUC1 (U11)]$_2$ diselenide and [Se-PEG$_6$]$_2$ diselenide (**16**) to PEG$_6$-MUC1 (U11) selenoether *via* optimized PDC functionalization over 120 s as monitored by UPLC (0-100% B over 5 min, Waters Acquity BEH300 1.7 μm, 2.1 x 50 mm (C18), λ = 214 nm). **B)** Associated plotted conversion data with sigmoidal fit. Conversions calculated through integration of all peptide-related MS TIC peaks.

Chemoselective functionalization of model peptide H_2N-USPCYSC-NH_2 with PEG_6 diselenide (at Sec)

H_2N-USPCYSC-NH_2 (0.15 mg, 81.5 nmol) was dissolved in 1:1 v/v H_2O:MeCN (58 μL) followed by addition of 6.8 μL of [Ir(dF(CF_3)ppy)$_2$(dtbpy)]PF_6 (**5**) (0.3 μmol mL^{-1}) for a photocatalyst loading of 2.5 mol.% and then further diluted with a solution of [Se-PEG_6]$_2$ diselenide (**16**) (0.45 mg, 0.65 μmol, 8 eq.) in 1:1 v/v H_2O:MeCN (50 μL). Finally, a solution of PTA (**4**) (0.23 mg, 1.47 μmol, 18 eq.) in 1:1 v/v H_2O:MeCN (50 μL) was added for a 0.5 mM concentration of the peptide H_2N-USPCYSC-NH_2. The reaction mixture was then irradiated with 450 nm light (PennOC M1 photoreactor) for 5 and 10 min. After irradiation, a 5 μL aliquot was taken out and diluted to 50 μL with water (0.1 vol% TFA) and directly analysed by UPLC-MS using a gradient of 0-60% B over 5 min (λ = 214 nm).

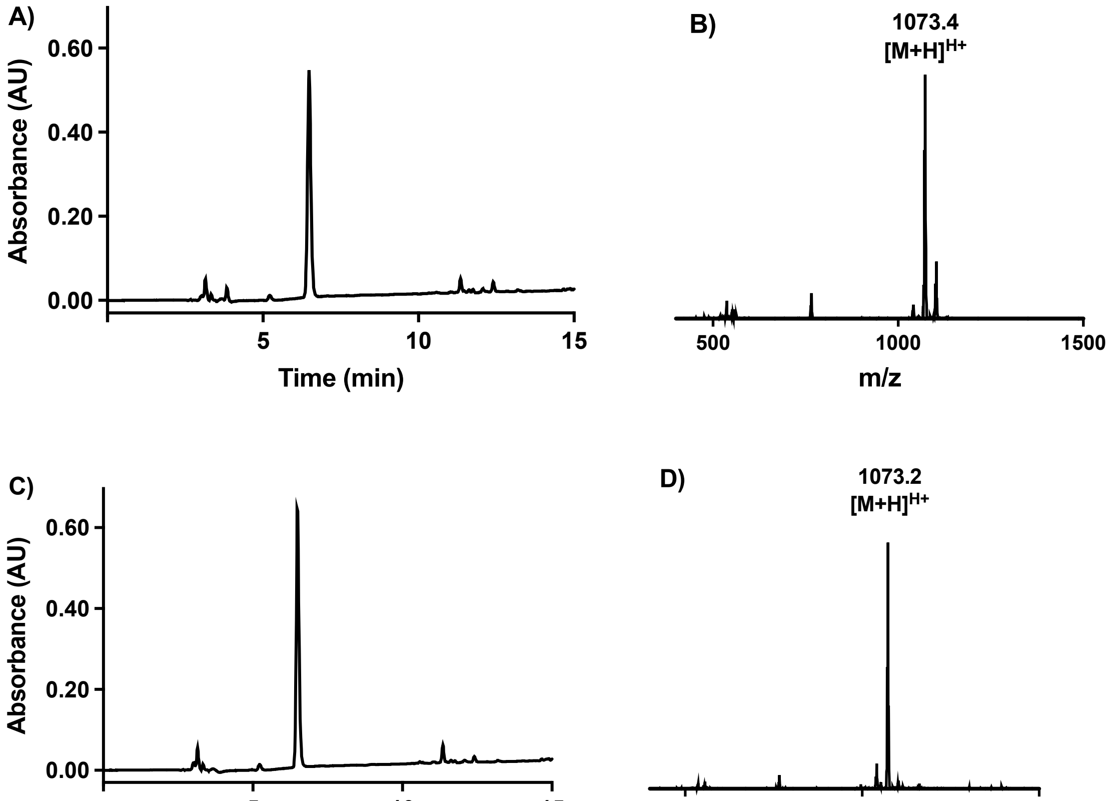

Supplementary Figure 72. A) and **C)** Crude analytical HPLC traces of PDC reaction between model peptide H_2N-USPCYSC-NH_2 and PEG_6 diselenide after 5 and 10 min, respectively; (1-50% B over 30 min, 0.1% v/v TFA, λ = 214 nm); **B)** and **D)** ESI-MS showing formation of USPCYSC-PEG_6 selenoether after 5 and 10 min; Calculated Mass: $[M+H]^+$: 1073.3; Mass Found (ESI+) 1073.2 $[M+H]^+$. ESI-MS data was collected over the entire gradient and wash cycle of the UPLC-MS.

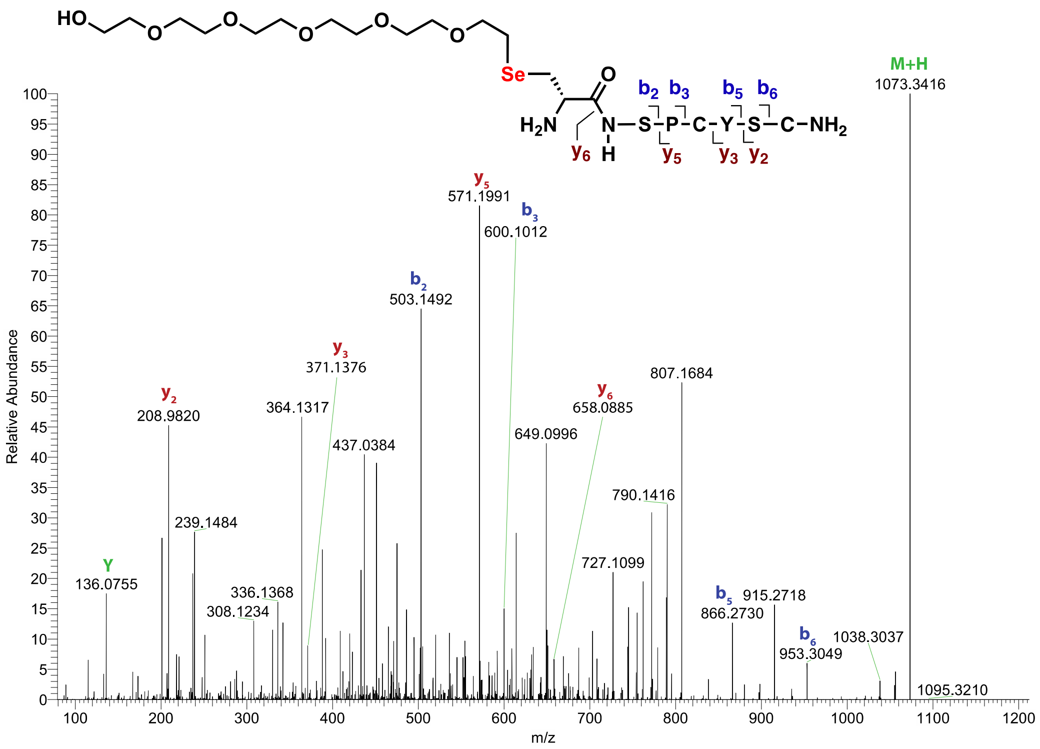

Supplementary Figure 73. MS-MS analysis of chemoselectively functionalized model peptide H_2N-USPCYSC-NH_2 with PEG_6 diselenide (at Sec).

Late-Stage PDC Functionalization of CaM (K148U) Diselenide 12

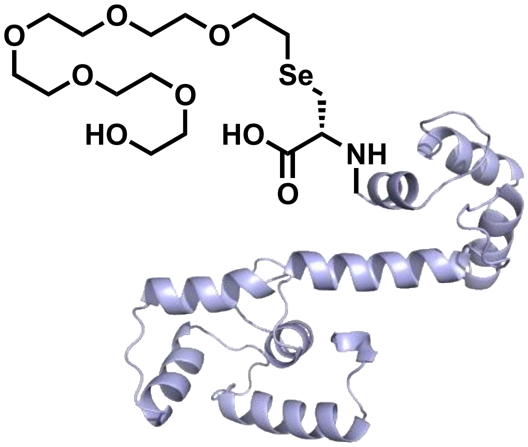

CaM (K148)-PEG$_6$ Selenoether 18

In a 1.5 mL Protein LoBind (Eppendorf, Hamburg, Germany) microcentrifuge tube, CaM (K148U) diselenide **12** (0.96 mg, 25 nmol), small molecule diselenide (0.80 µmol, 32 eq.),

PTA (**4**) (0.26 mg, 1.65 μmol, 66 eq.) and [Ir(dF(CF$_3$)ppy)$_2$(dtbpy)]PF$_6$ (**5**) (10 mol% relative to **12**) [8.4 μL from a 0.3 μmol mL^{-1} stock in 1:1 v/v MeCN:H$_2$O] were dissolved in 50 μL of 50 vol% MeCN in 1 M Gnd.HCl, 0.02 M NaP$_i$, pH 7.0 buffer under a stream of N$_2$ to achieve a protein diselenide **12** concentration of 0.5 mM. The reaction tube was capped and immediately irradiated with 450 nm LED light (PennOC M1 photoreactor) at 37 °C for 5 min.

Two 5 μL aliquots were taken at 0 min and 5 min time points and individually diluted 4-fold in 6 M Gnd.HCl, 0.1 M NaP$_i$, pH 7.0 buffer (15 μL). One aliquot was desalted using a C18 ZipTip (Merck, Darmstadt, Germany) and analyzed by MALDI-TOF MS (*see General Procedures*). The other diluted aliquot was directly analyzed by LC-HRMS (*see General Procedures*). The product **18** was afforded in 90.9 ± 0.6% conversion from the diselenide starting material **12**, as calculated through averaging integrations of HRMS-derived extracted ion chromatograms of the [M+11H]$^{11+}$, [M+10H]$^{10+}$ and [M+9H]$^{9+}$ charge states. Conversion errors are reported as the standard deviation of these three ion peak integrations for a single experiment.

See next pages for analytical data.

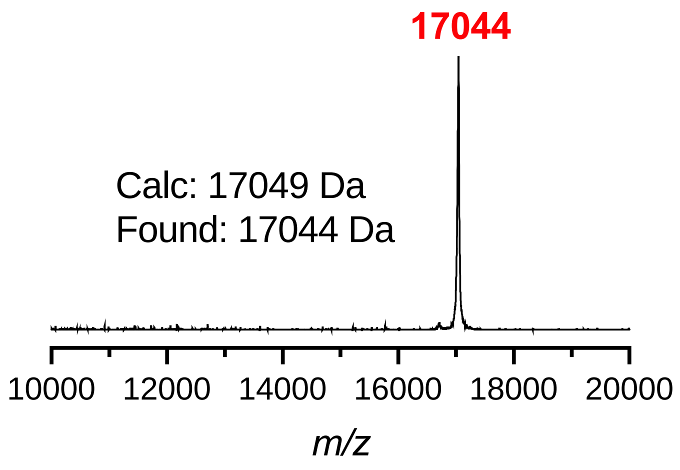

Supplementary Figure 74. MALDI-TOF MS spectrum of the crude reaction mixture of the PDC functionalization of CaM (K148U) diselenide **12** with [Se-PEG$_6$]$_2$ (**16**) after 5 min irradiation at 450 nm.

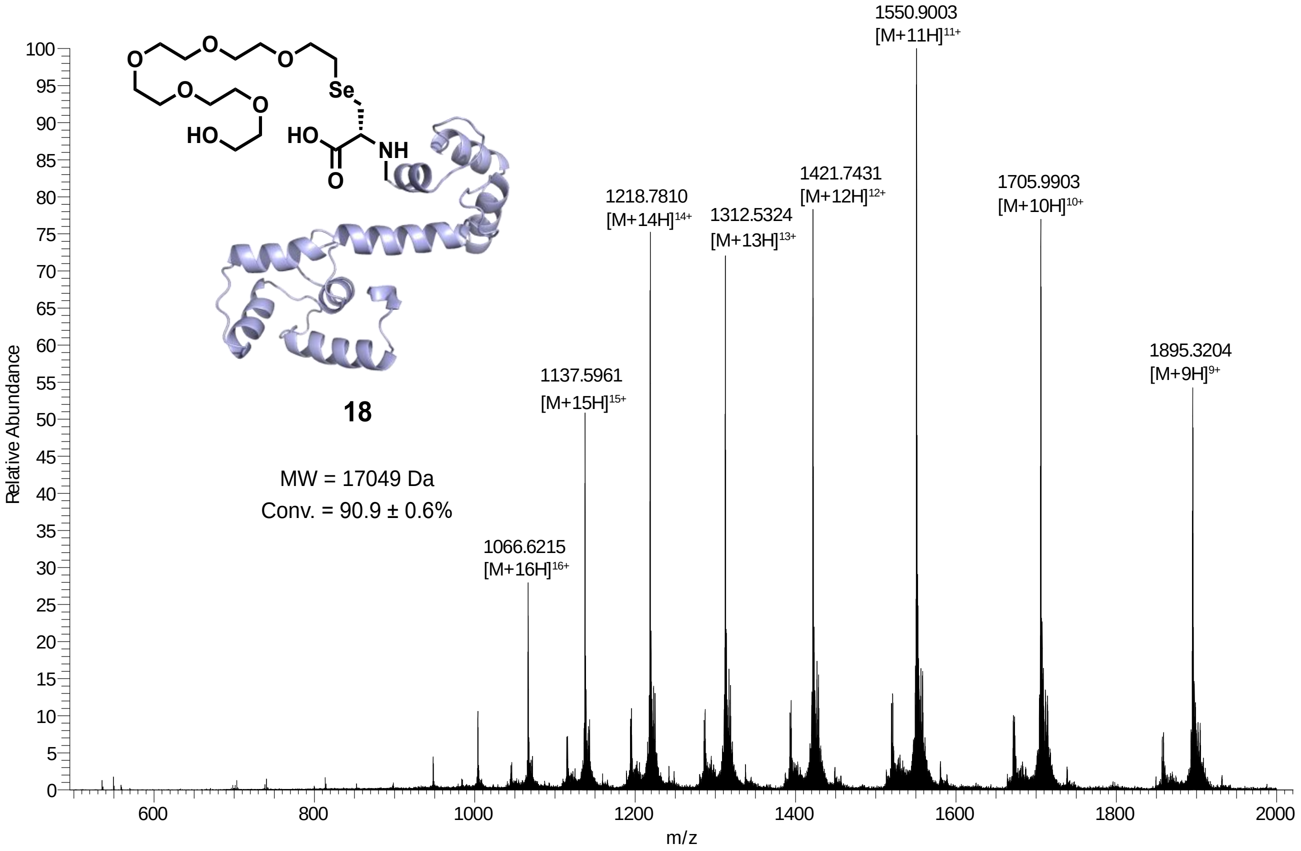

Supplementary Figure 75. HRMS spectrum of the crude reaction mixture of the PDC functionalization of CaM (K148U) diselenide **12** with [Se-PEG$_6$]$_2$ (**16**) after 5 min irradiation at 450 nm. Ion peaks for the CaM (K148U)-PEG$_6$ selenoether **18** product are labelled. Reaction conversions were calculated through averaging integrations of HRMS-derived extracted ion chromatograms of the $[M+11H]^{11+}$, $[M+10H]^{10+}$ and $[M+9H]^{9+}$ charge states and errors are reported as the standard deviation of the integration of these three ion peaks for a single experiment.

Crystallization Trials of PEG_6-CaM (K148U) Selenoether 18

Crystallization experiments on modified PEG_6-CaM (K148U) **18** were performed using a sitting-drop vapour-diffusion method. The modified protein **18** (243 µM concentration) was dialyzed into a buffer comprising 10 mM HEPES pH 7.4, 150 mM NaCl, 1 mM DTT, and 1 mM $CaCl_2$ in preparation for crystallography. Crystallization trials were performed using the commercial PACT and JCSG+ 96-well crystallization screens (Molecular Dimensions). PEG_6-CaM (K148U) **18** was dispensed into MRC two-drop chamber, 96-well crystallization plates using a Mosquito crystallization robot and mixed with each crystallization condition, maintaining a final drop volume of 300 nL. The plates were incubated at 18 °C and protein crystals appeared in a condition containing 0.2 M $MgCl_2$, 0.1 M Tris (pH 8.5) and 20% w/v PEG8000 after 7 days. The crystals were fished and plunge-frozen in liquid nitrogen following cryoprotection using 10 vol% glycerol in the mother liquid from which the crystals were derived.

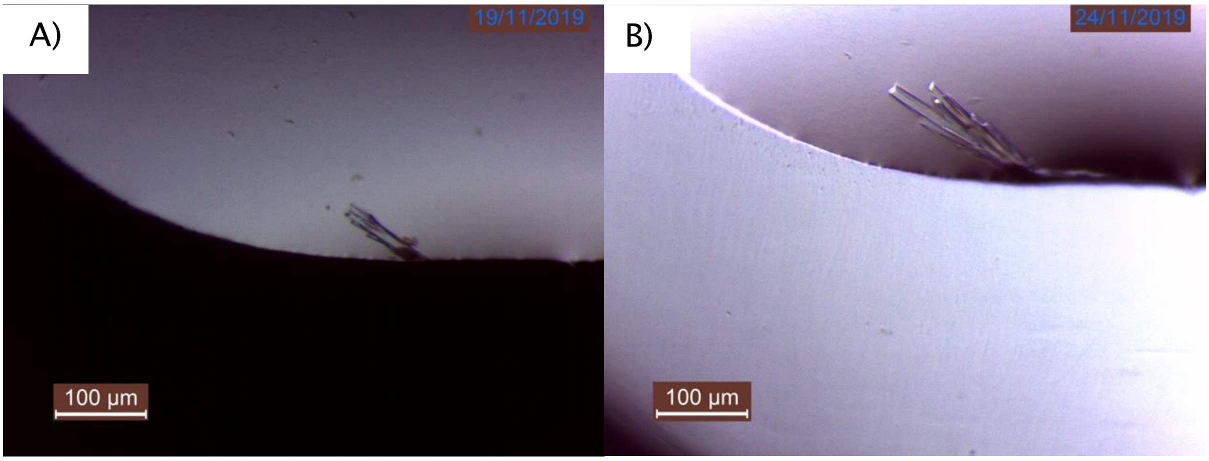

Supplementary Figure 76. Crystals of PEG_6-CaM (K148U) selenoether **18** observed after **A)** 7 days and **B)** 12 days, under the following conditions: 0.2 M $MgCl_2$, 0.1 M Tris (pH 8.5), 20% w/v PEG8000.

X-Ray Crystallography of PEG_6-CaM (K148U) Selenoether 18

This research was undertaken using the MX2 beamline at the Australian Synchrotron, part of ANSTO, and made use of the Australian Cancer Research Foundation (ACRF) Eiger 16M detector.

X-Ray diffraction data (*Supplementary Figure 77*) were collected from frozen crystals at the Australian Synchrotron using the Macromolecular Crystallography MX2 (microfocus) beamline at 100K and a wavelength of 0.9537 Å.[54] Data from a single crystal were integrated using XDS and scaled using AIMLESS from the CCP4i suite.[55,56] PhaserMR was used to calculate the initial phases using an existing structure of CaM as a molecular replacement model (PDB ID: 6EEB). The model was built by iterative rounds of manual model building using COOT followed by refinement using Phenix.[57,58] The quality of the final model was validated using the wwPBD server and deposited to the PDB (ID: 7T2Q). The data collection and refinement statistics for this structure are listed in *Supplementary Table 9*. Structure diagrams were generated using PyMOL.

Fluorescence excitation scans of single PEG_6-CaM (K148U) crystals were collected using the MX2 beamline synchrotron radiation to enable heavy-metal identification (*Supplementary Figure 78*. The excitation scans were performed at across an 0-20.5 keV energy range for ~1-5 seconds. The identity of the peaks in the resultant spectra were confirmed by matching to existing reference element fluorescence emission data.

Supplementary Table 9. Data collection and refinement statistics for the crystal structure of PEG_6-CaM (K148U) (PDB ID: 7T2Q). Values in parentheses are for highest-resolution shell. All data were collected from a single crystal.

	PDB ID: 7T2Q
**Data collection**	
Space group	C 2 2 21
Cell dimensions	
a, b, c (Å)	51.051, 56.347, 116.606
α, β, γ (°)	90.00, 90.00, 90.00
Resolution (Å)	38.87-1.95 (2.0-1.95)*
R_{merge}	0.126 (0.942)
$CC_{1/2}$	0.997 (0.815)
$I / \sigma I$	10.9 (2.0)
Completeness (%)	99.5 (100.0)
Redundancy	5.6 (5.8)
**Refinement**	
Resolution (Å)	37.83-1.95
No. reflections	12524
R_{work} / R_{free}	0.2208/0.2577
No. atoms	1266
Protein	1163
Ligand/ion	7
Water	96
Average B-factors	28.0

R.m.s. deviations	
Bond lengths (Å)	0.004
Bond angles (°)	0.684

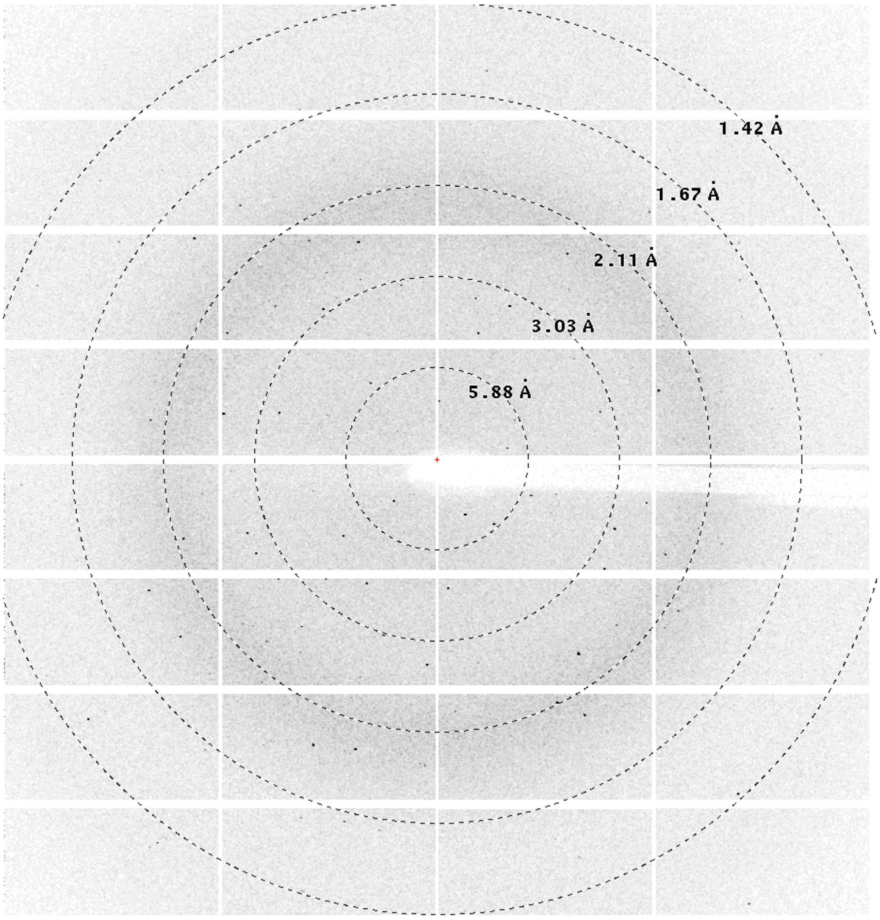

Supplementary Figure 77. 1.95 Å diffraction pattern obtained for a crystal of PEG$_6$-CaM (K148U) selenoether **18**.

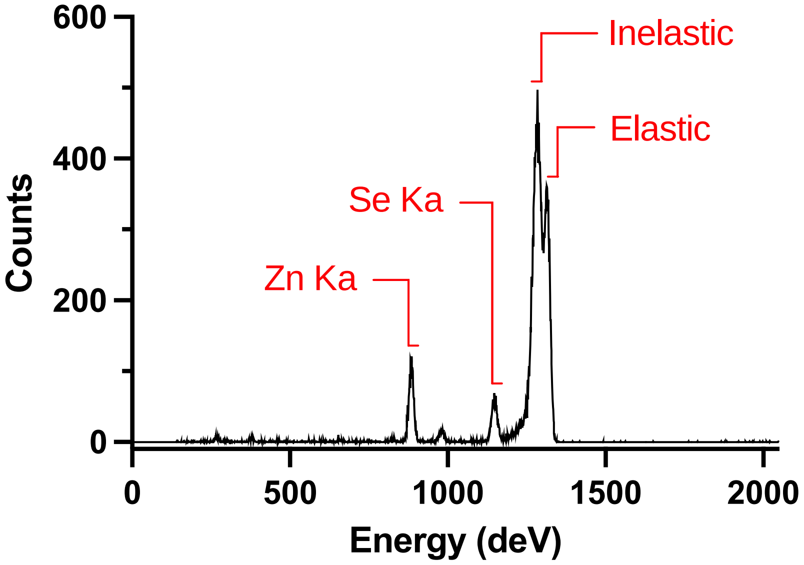

Supplementary Figure 78. Fluorescence excitation scan of PEG_6-CaM (K148U) selenoether **18** across 0-20.5 keV for 1-5 seconds. Selenium and zinc Kα fluorescence peaks, along with protein elastic and inelastic fluorescence peaks, are labelled in red.

NMR spectra

See next page(s) for NMR spectra.

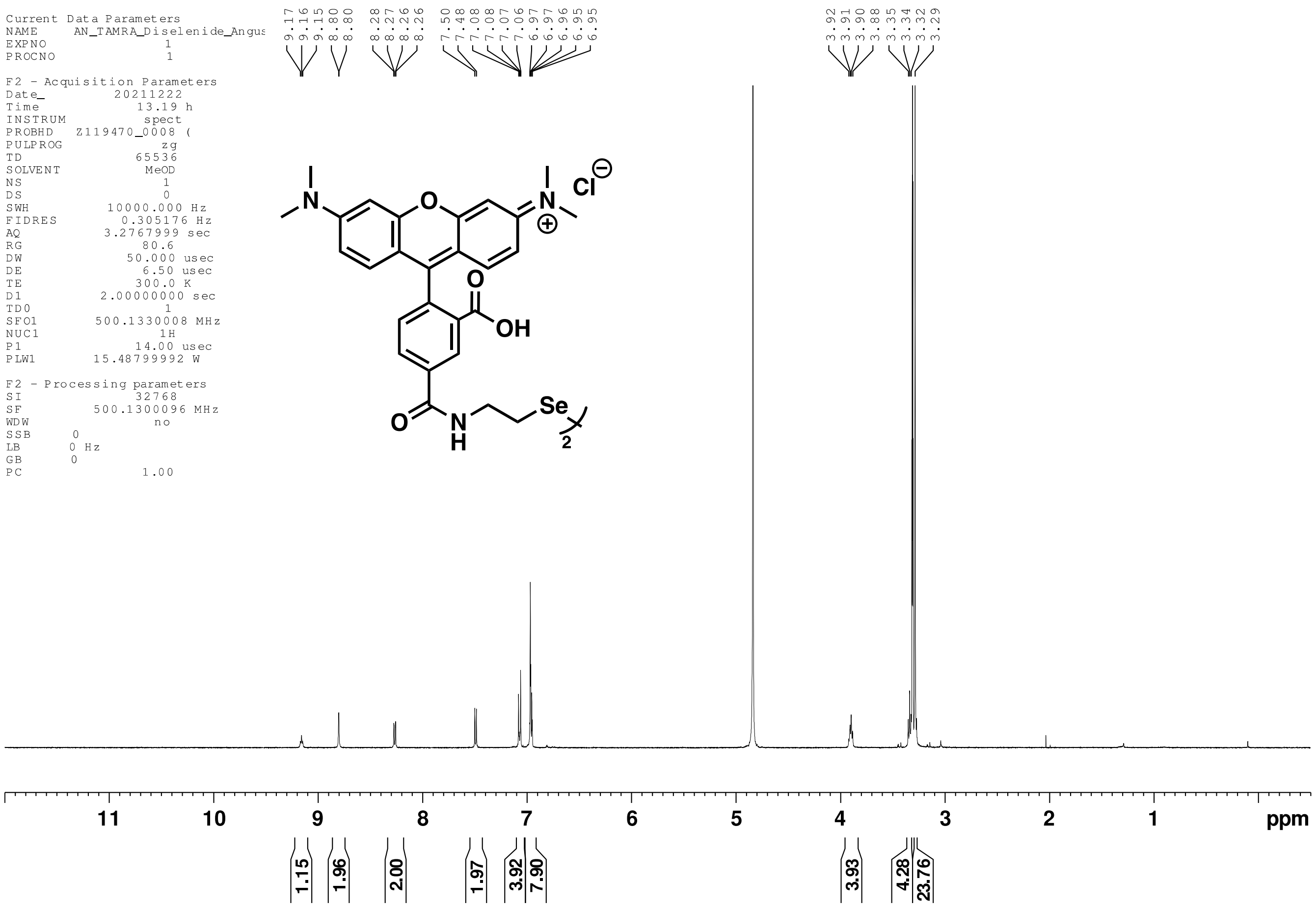

Supplementary Figure 79. ^{1}H NMR spectrum (500 MHz, CD_3OD) of compound **15**.

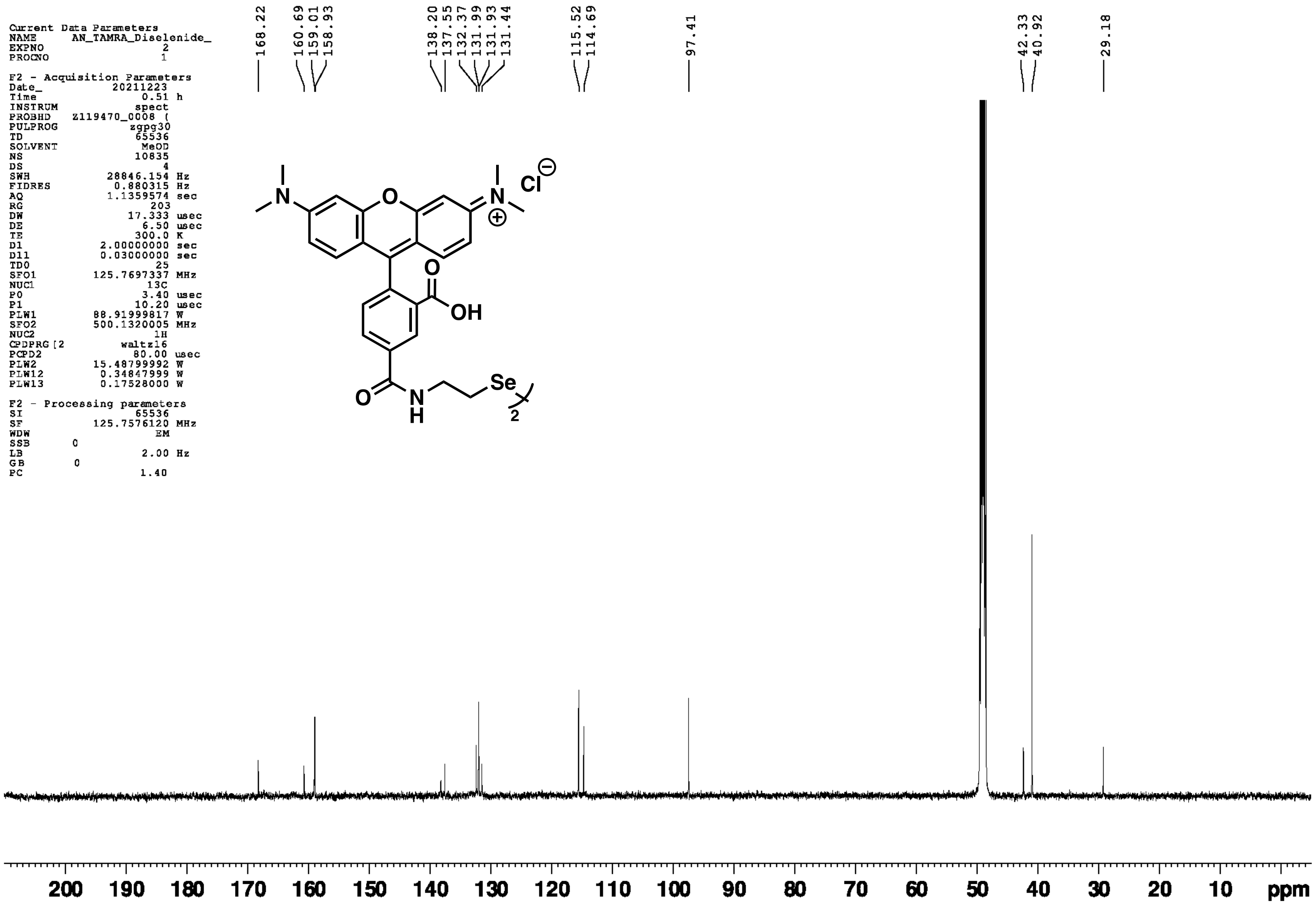

Supplementary Figure 80. ^{13}C NMR spectrum (126 MHz, CD_3OD) of compound **15**.

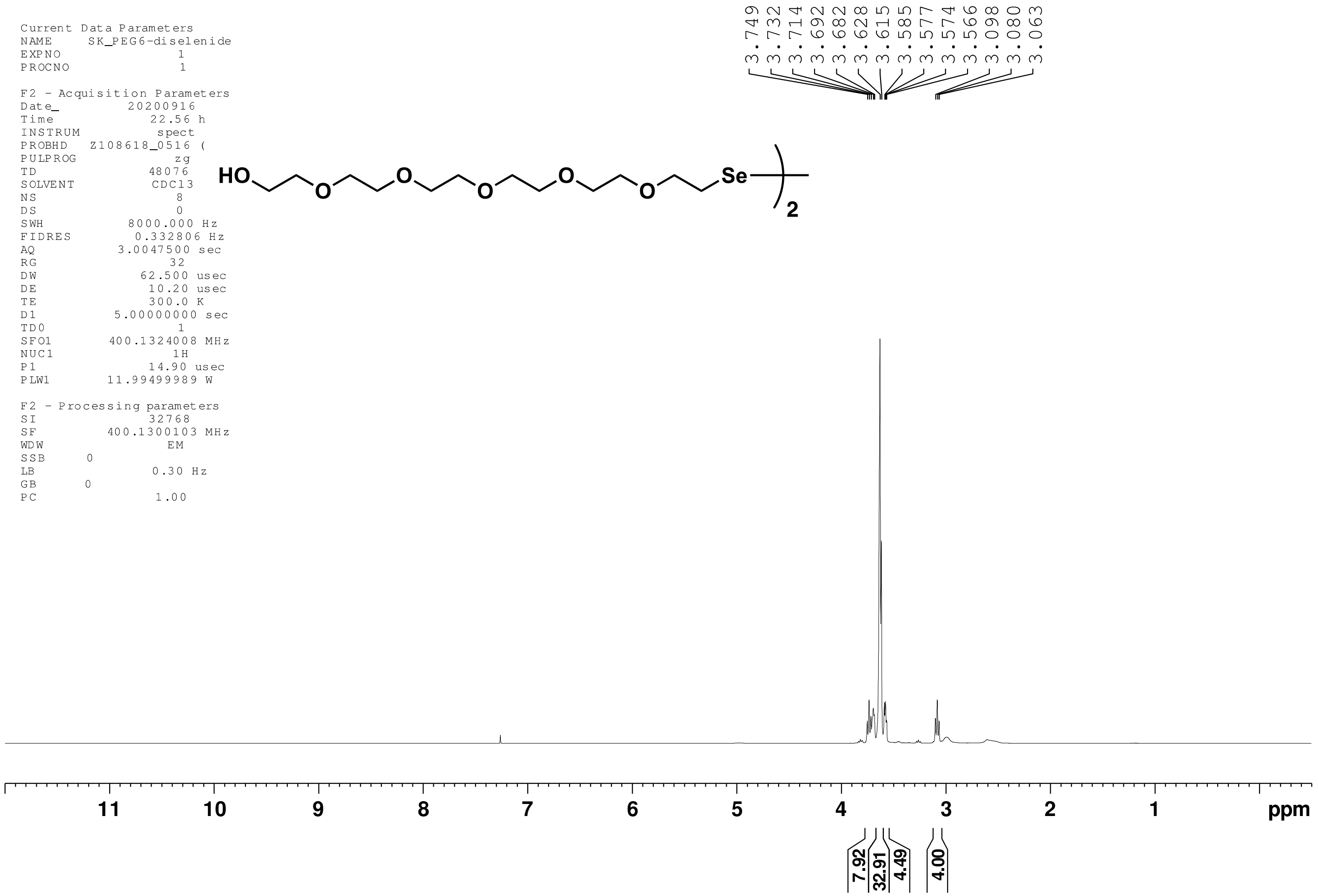

Supplementary Figure 81. ^{1}H NMR spectrum (400 MHz, $CDCl_3$) of compound **16**.

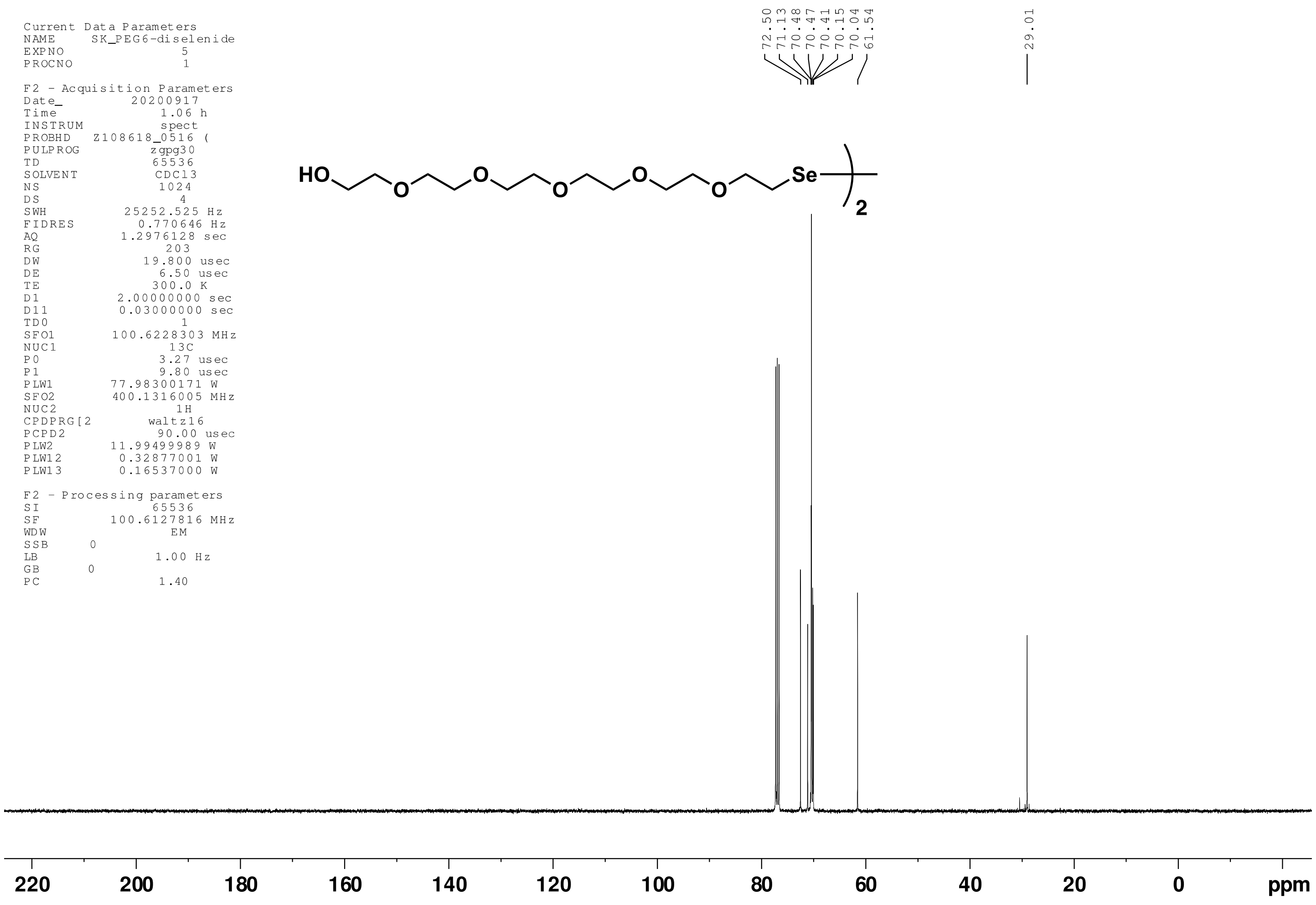

Supplementary Figure 82. ^{13}C NMR spectrum (101 MHz, $CDCl_3$) of compound **16**.

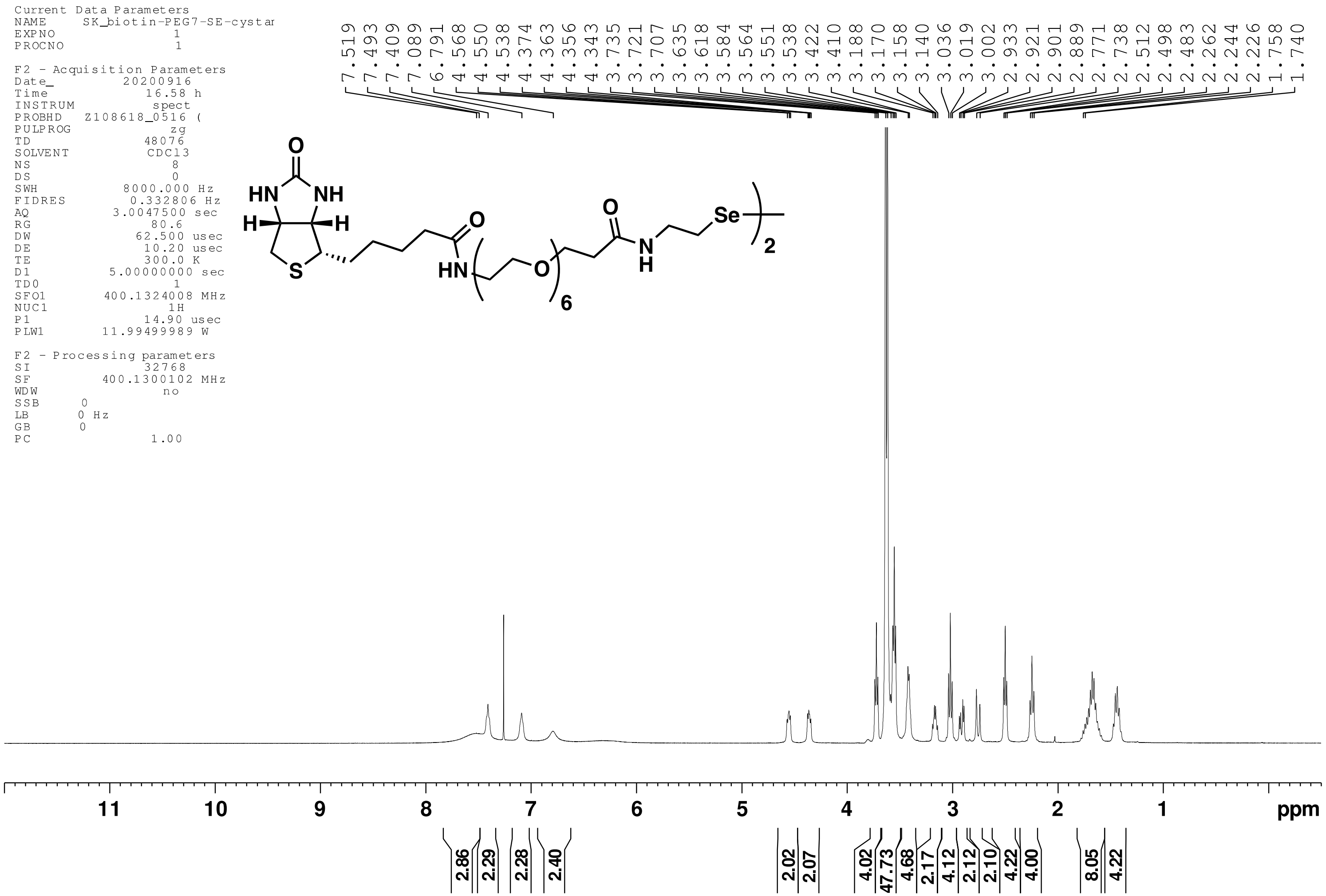

Supplementary Figure 83. ^{1}H NMR spectrum (400 MHz, $CDCl_3$) of Biotin-PEG$_5$ diselenide.

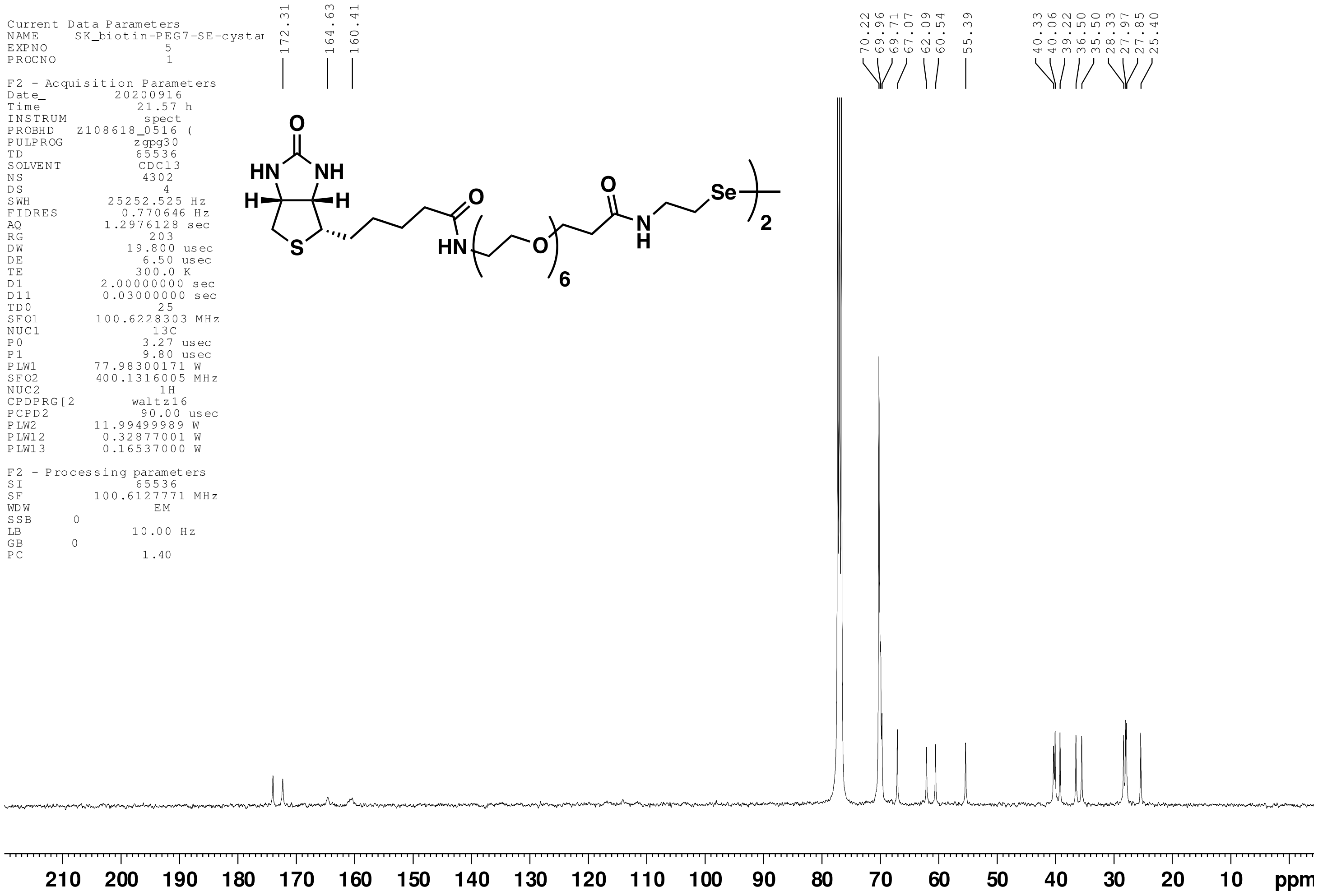

Supplementary Figure 84. ^{13}C NMR spectrum (101 MHz, $CDCl_3$) of Biotin-PEG$_5$ diselenide.

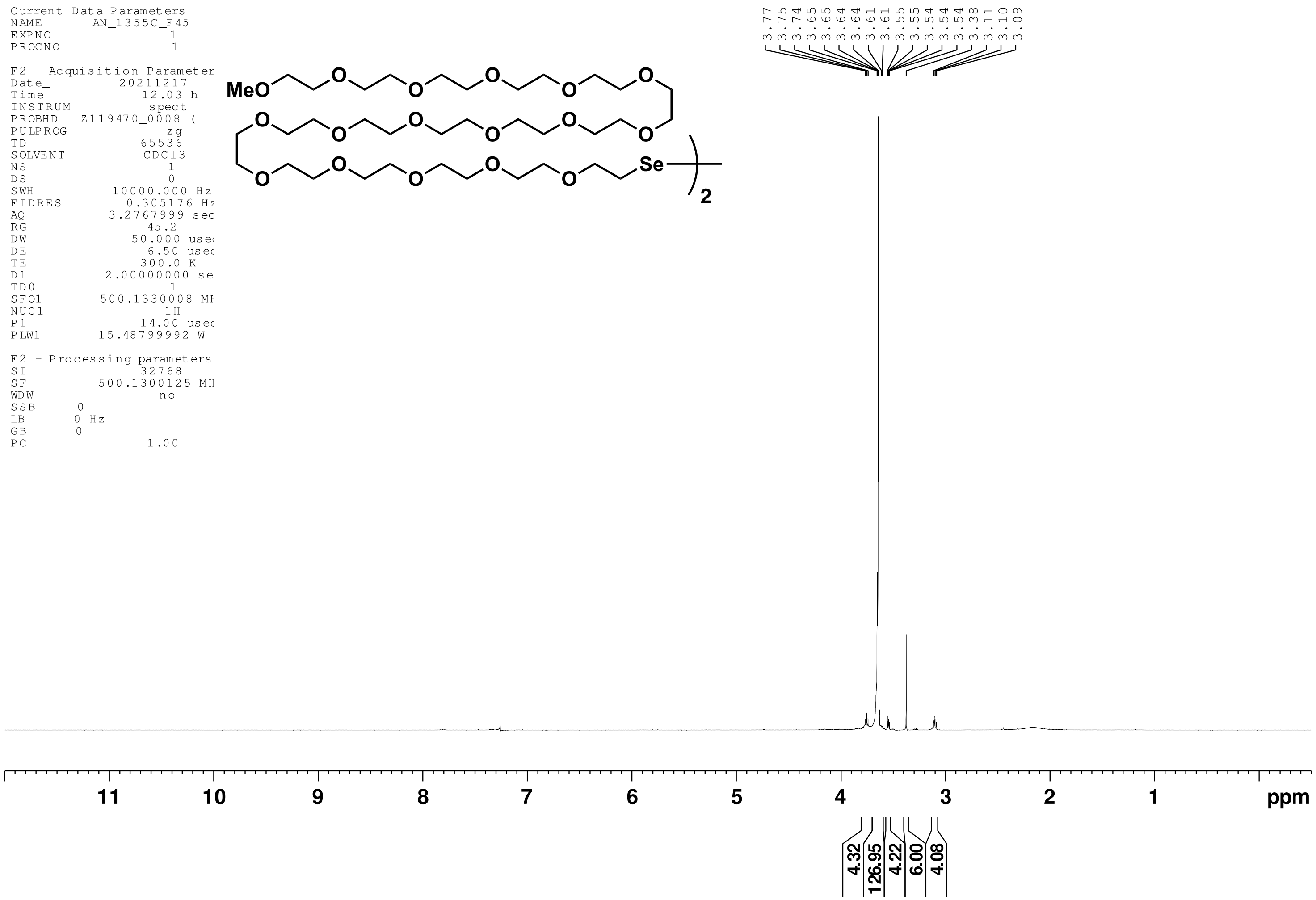

Supplementary Figure 85. ^{1}H NMR spectrum (500 MHz, $CDCl_3$) of $mPEG_{17}$ diselenide.

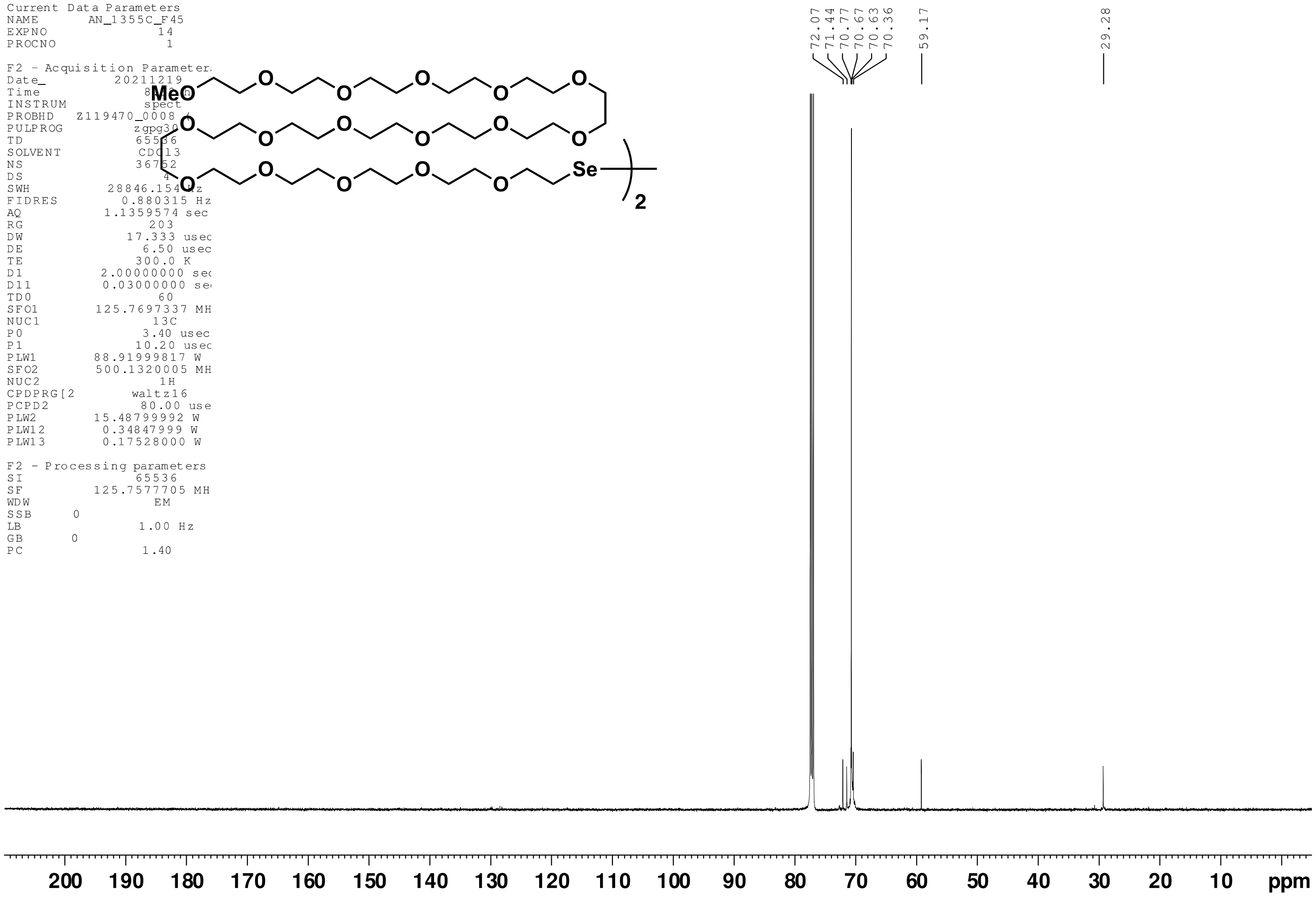

Supplementary Figure 86. ^{13}C NMR spectrum (126 MHz, $CDCl_3$) of $mPEG_{17}$ diselenide.

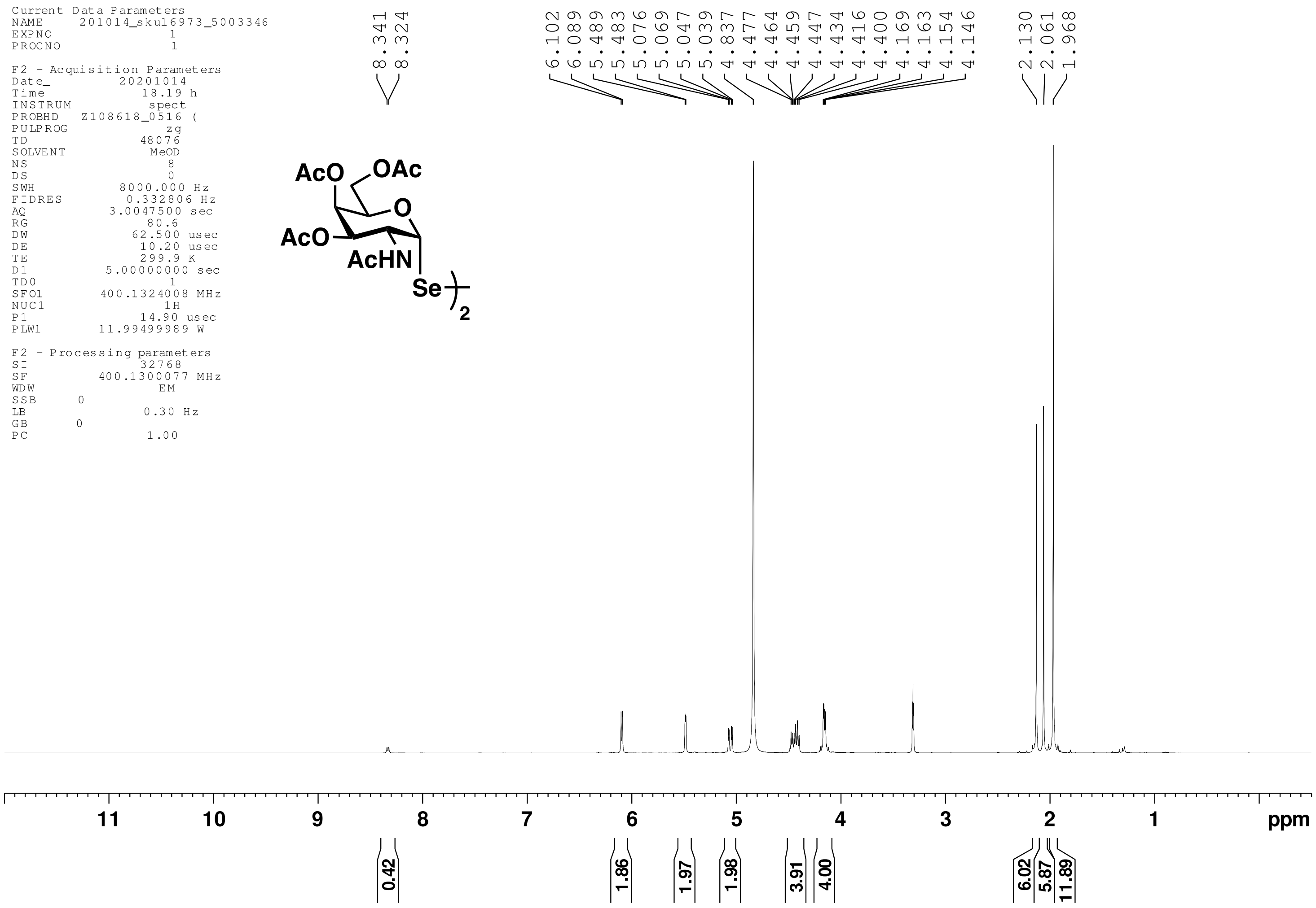

Supplementary Figure 87. ^{1}H NMR spectrum (400 MHz, CD_3OD) of α-GalNAc diselenide.

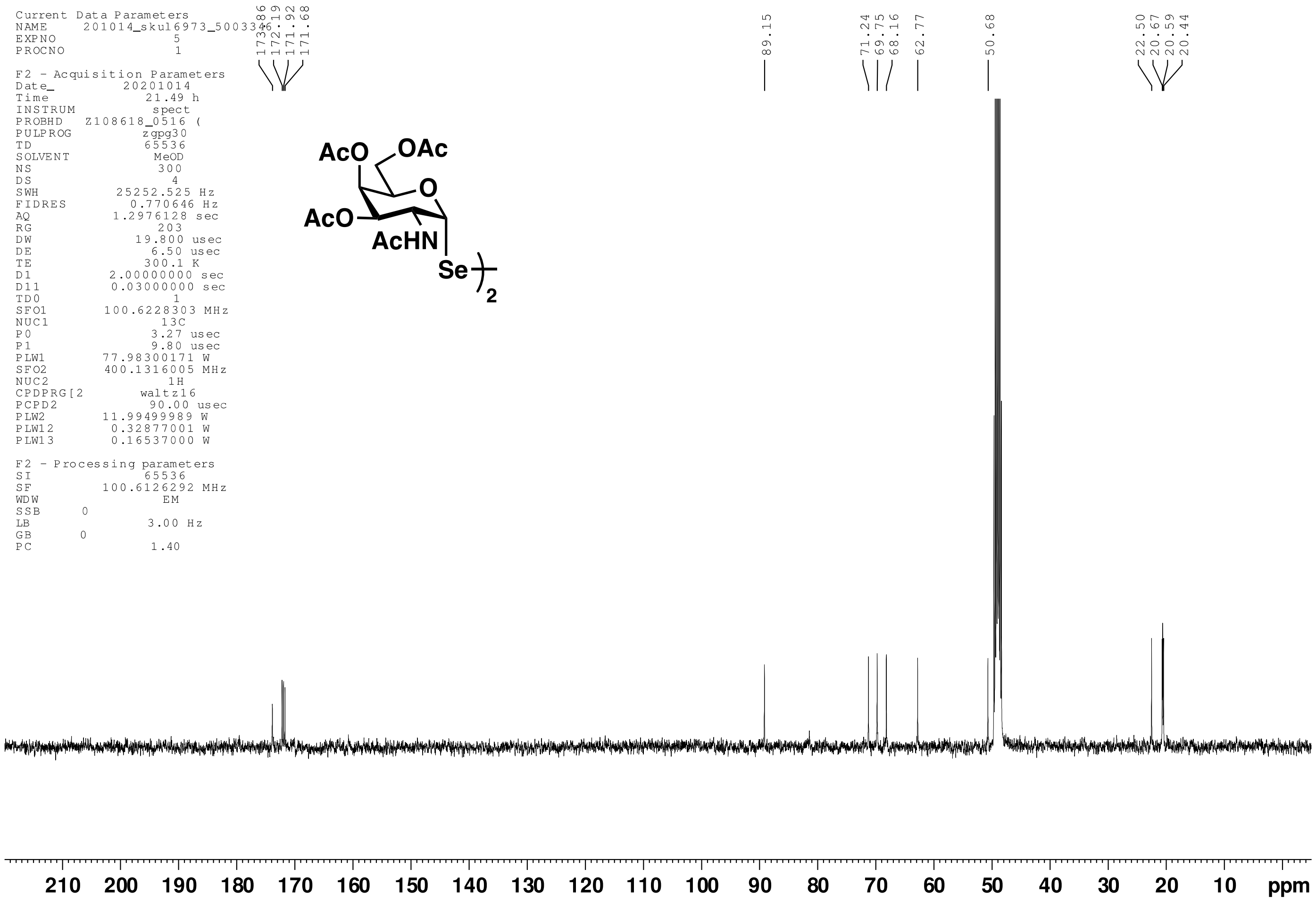

Supplementary Figure 88. ^{13}C NMR spectrum (101 MHz, CD_3OD) of α-GalNAc diselenide.

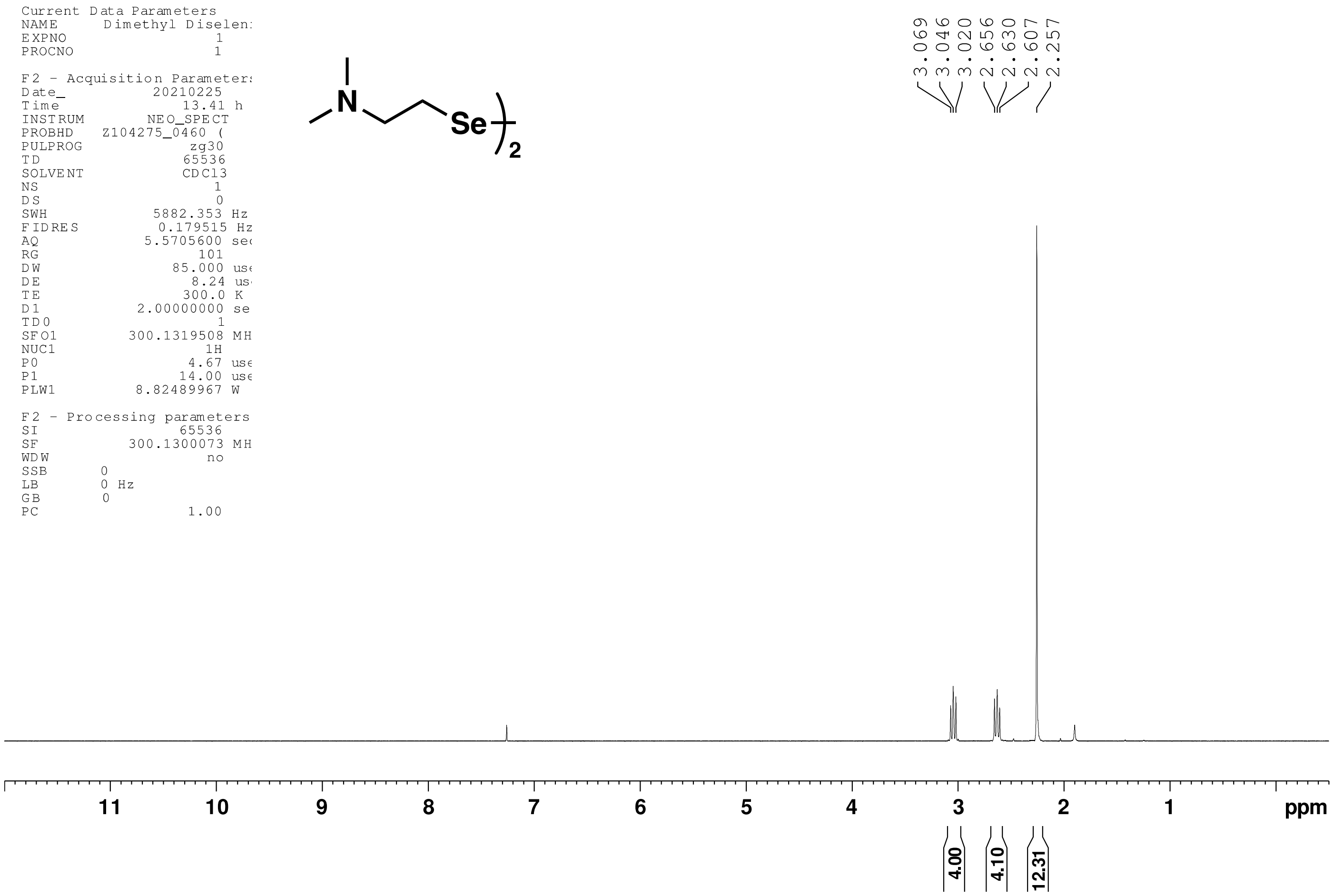

Supplementary Figure 89. ^{1}H NMR spectrum (400 MHz, $CDCl_3$) of 2,2'-diselanediylbis(*N*,*N*-dimethylethan-1-amine).

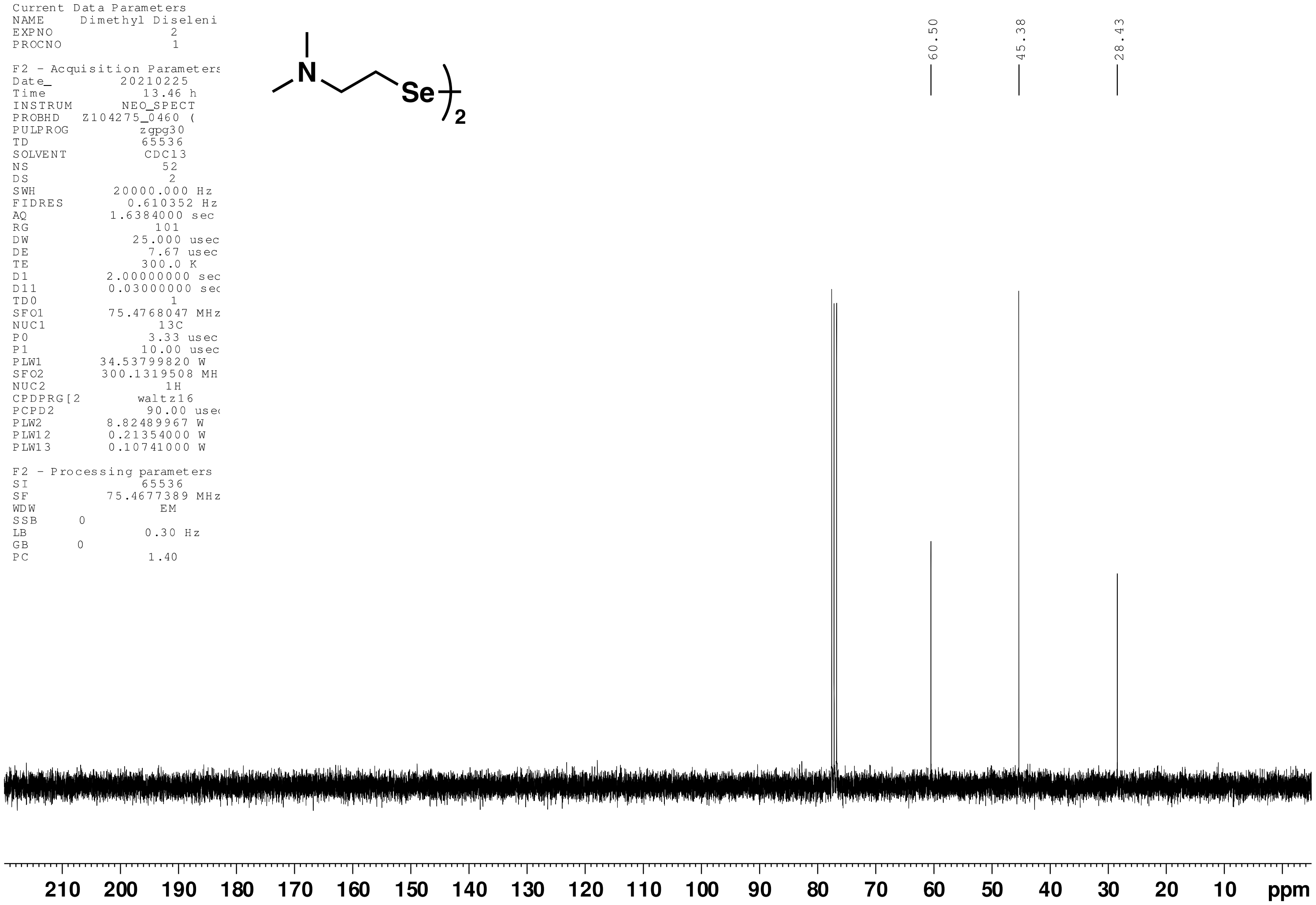

Supplementary Figure 90. ^{13}C NMR spectrum (101 MHz, $CDCl_3$) of 2,2'-diselanediylbis(*N*,*N*-dimethylethan-1-amine).

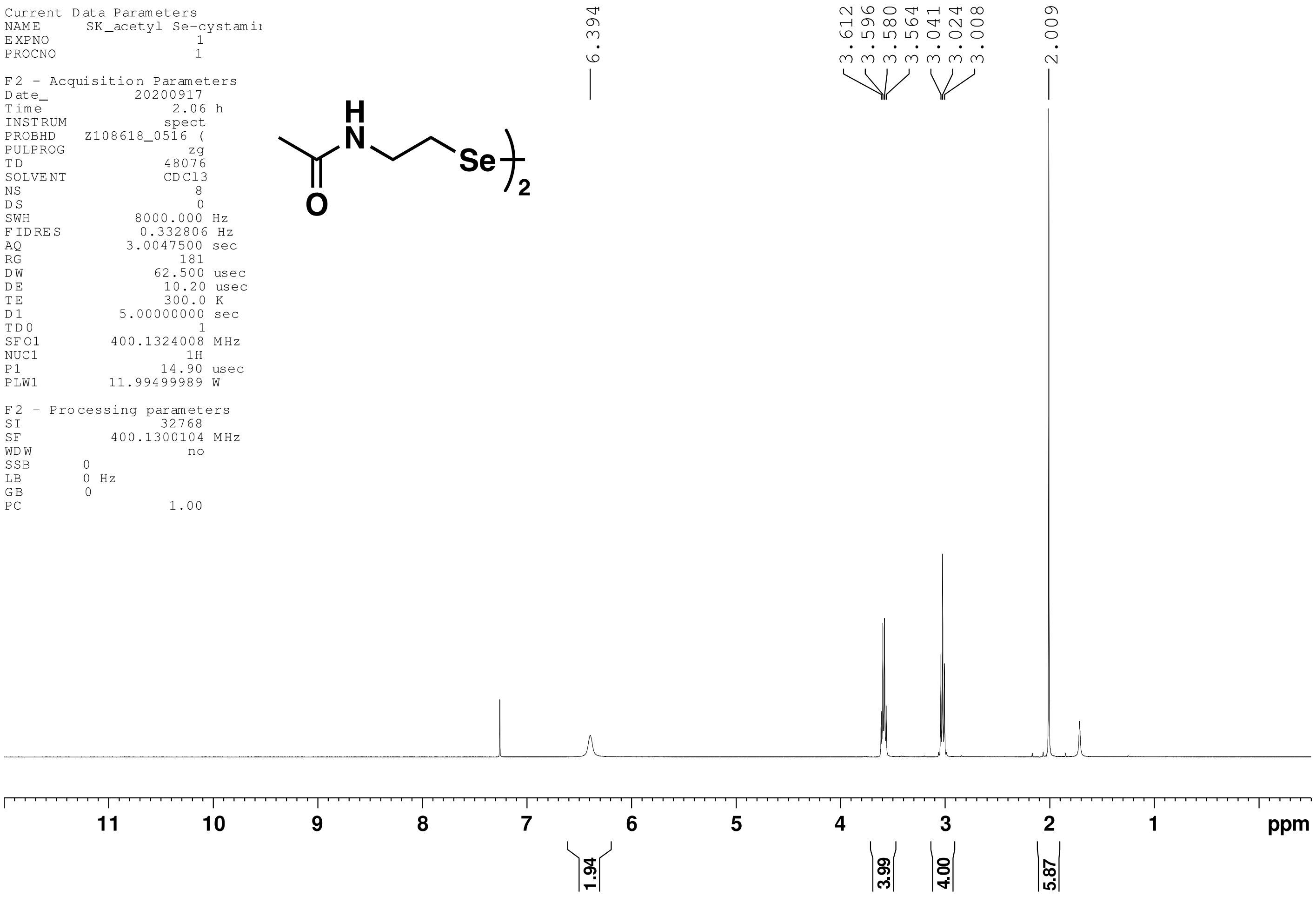

Supplementary Figure 91. ^{1}H NMR spectrum (400 MHz, $CDCl_3$) of *N*,*N*'-(diselanediylbis(ethane-2,1-diyl))diacetamide.

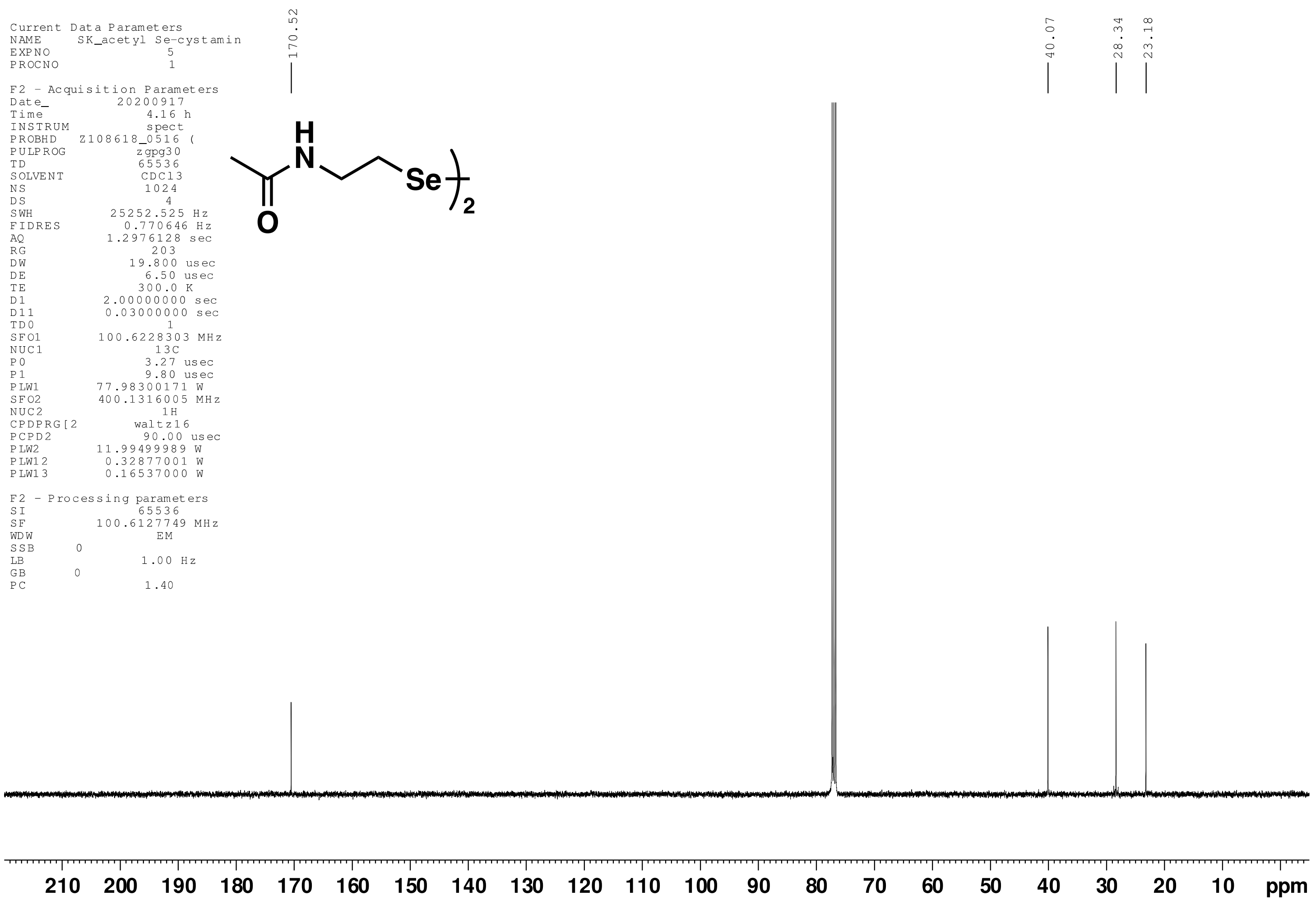

Supplementary Figure 92. ^{13}C NMR spectrum (101 MHz, $CDCl_3$) of *N*,*N'*-(diselanediylbis(ethane-2,1-diyl))diacetamide.

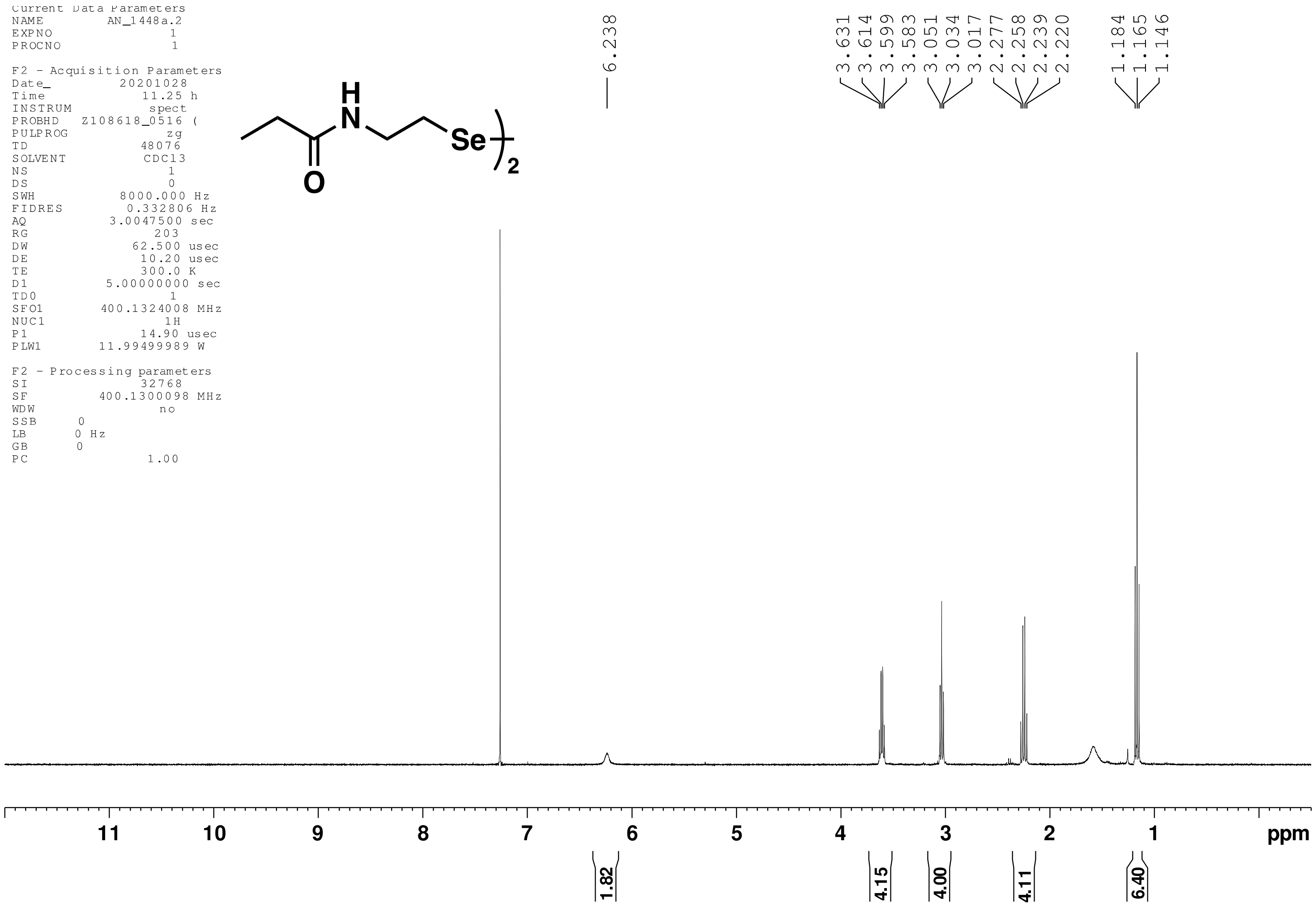

Supplementary Figure 93. ^{1}H NMR spectrum (400 MHz, $CDCl_3$) of *N*,*N*'-(diselanediylbis(ethane-2,1-diyl))dipropionamide.

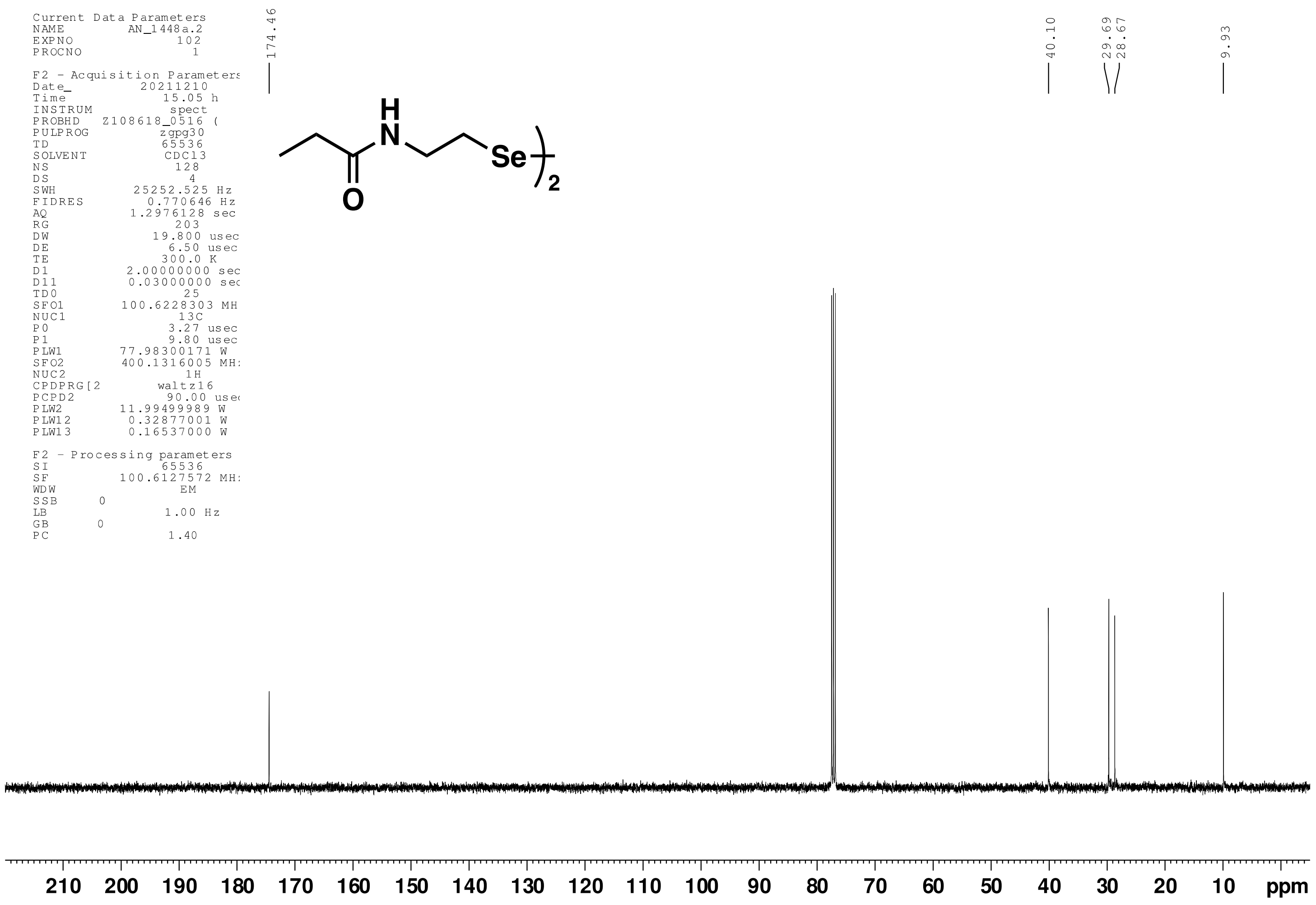

Supplementary Figure 94. ^{13}C NMR spectrum (101 MHz, $CDCl_3$) of *N*,*N*'-(diselanediylbis(ethane-2,1-diyl))dipropionamide.

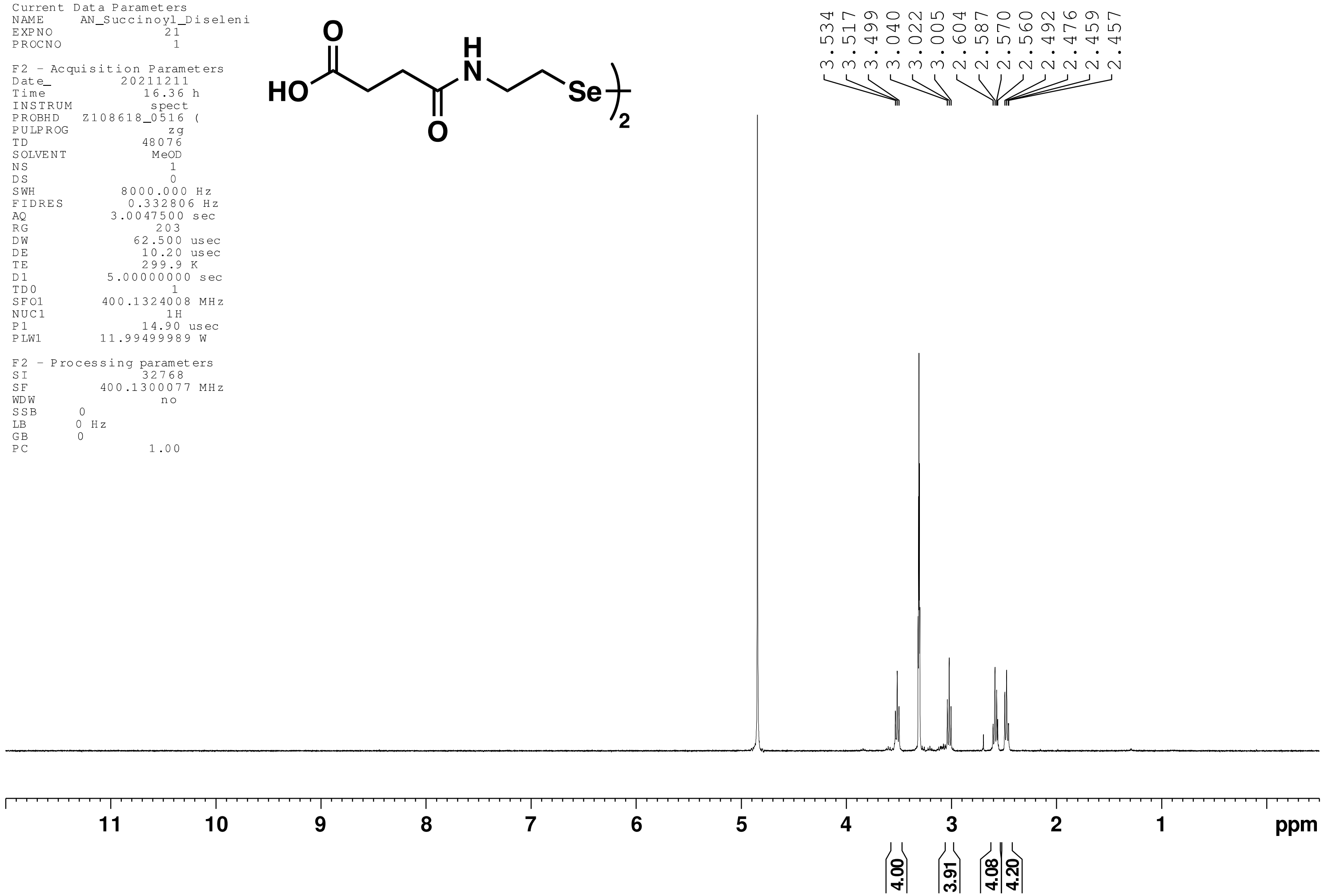

Supplementary Figure 95. ^{1}H NMR spectrum (400 MHz, CD_3OD) of 4,4'-((diselanediylbis(ethane-2,1-diyl))bis(azanediyl))bis(4-oxobutanoic acid).

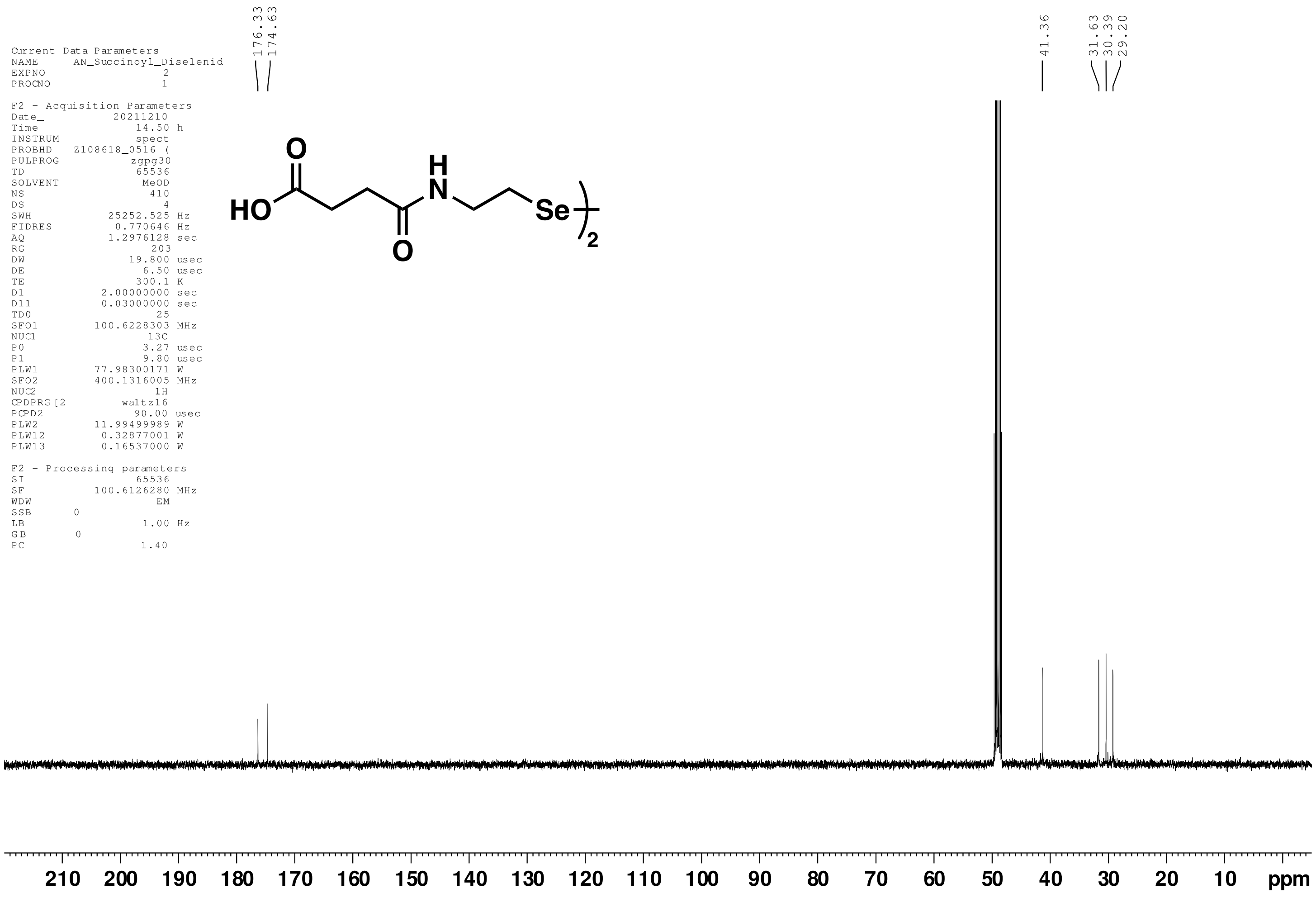

Supplementary Figure 96. ^{13}C NMR spectrum (101 MHz, CD_3OD) of 4,4'-((diselanediylbis(ethane-2,1-diyl))bis(azanediyl))bis(4-oxobutanoic acid).

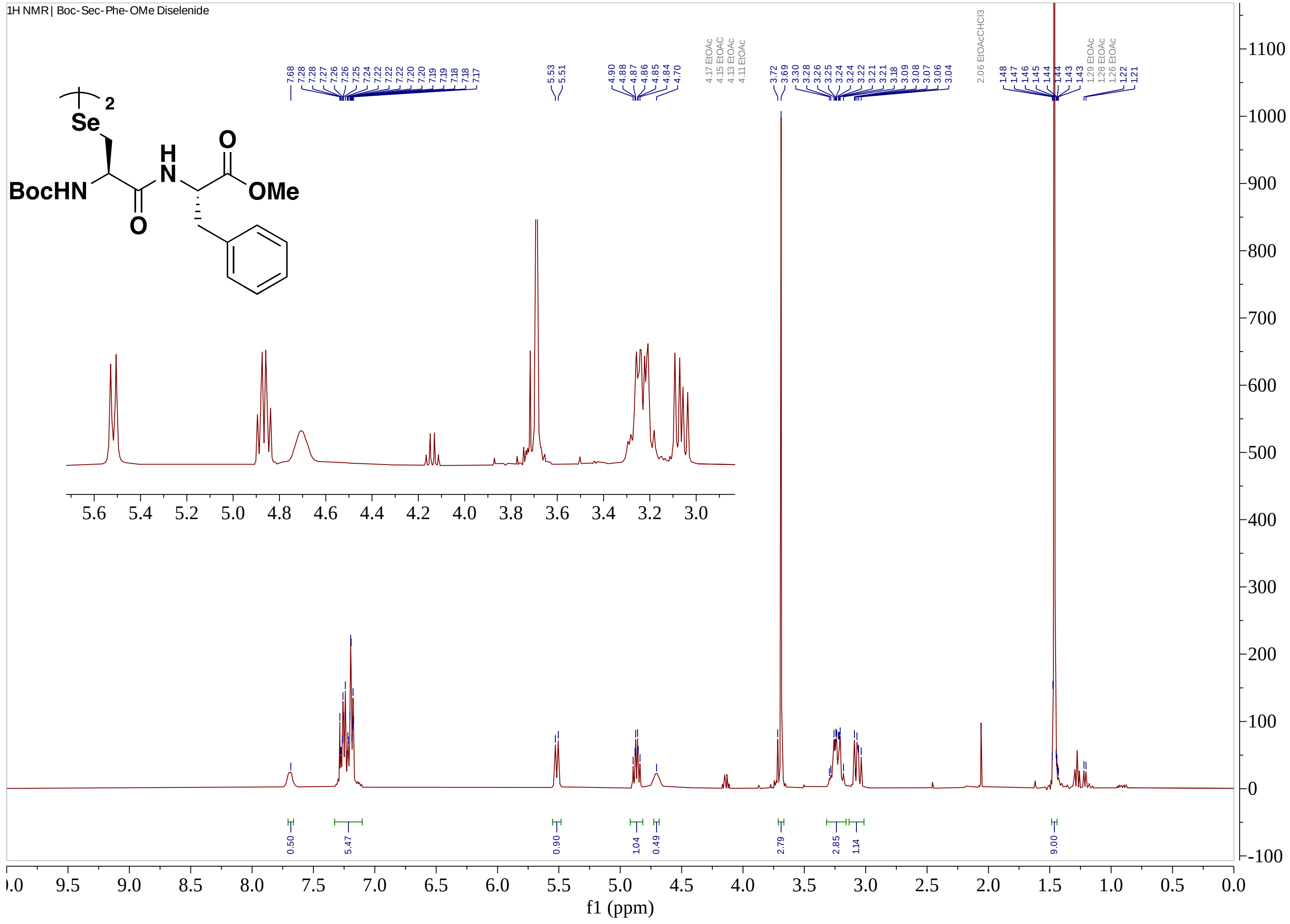

Supplementary Figure 97. ^{1}H NMR spectrum (400 MHz, $CDCl_3$) of $[\text{Boc-Sec-Phe-OMe}]_2$ diselenide

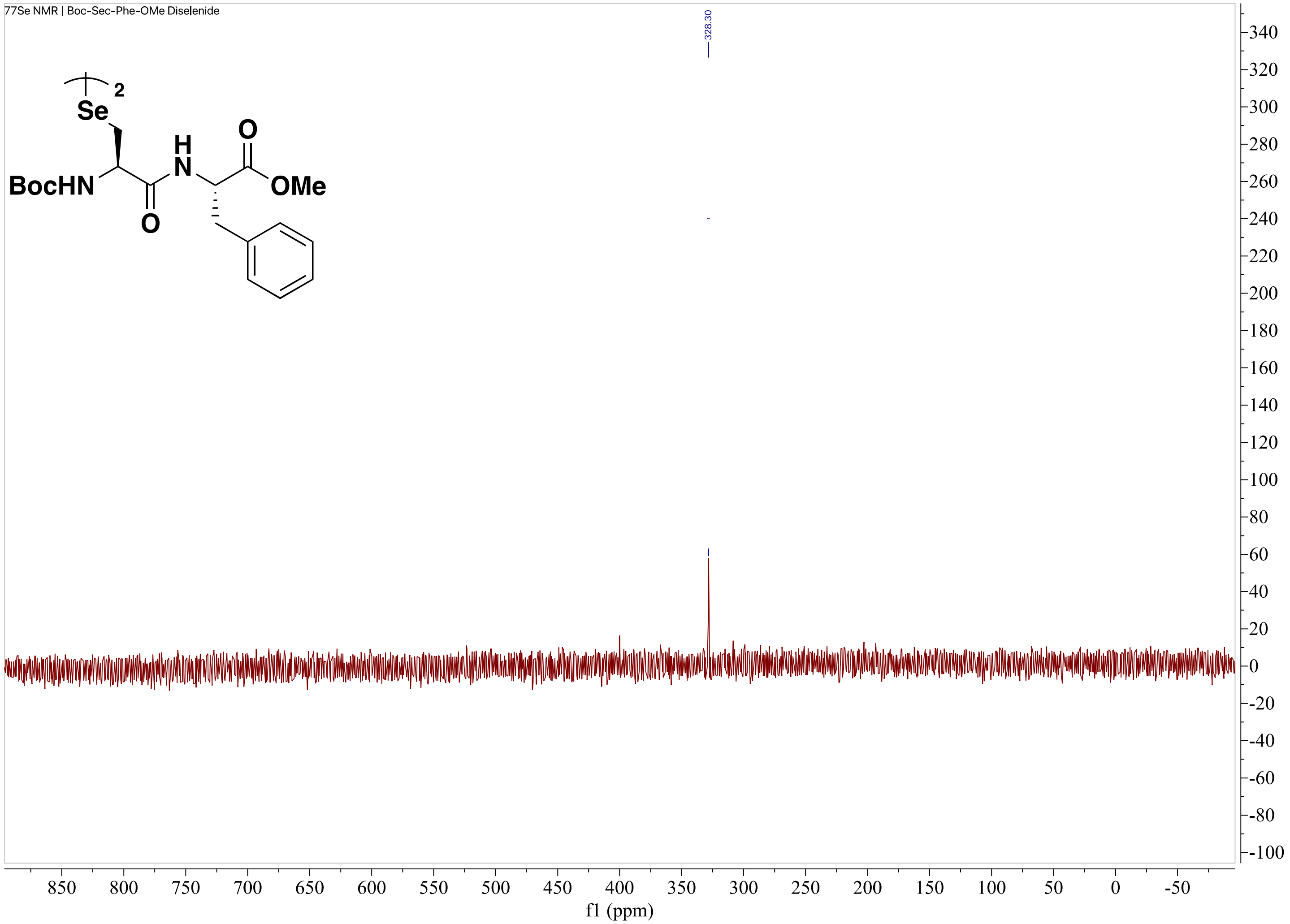

Supplementary Figure 98. ^{77}Se NMR spectrum (76 MHz, $CDCl_3$) of [Boc-Sec-Phe-OMe]$_2$ diselenide

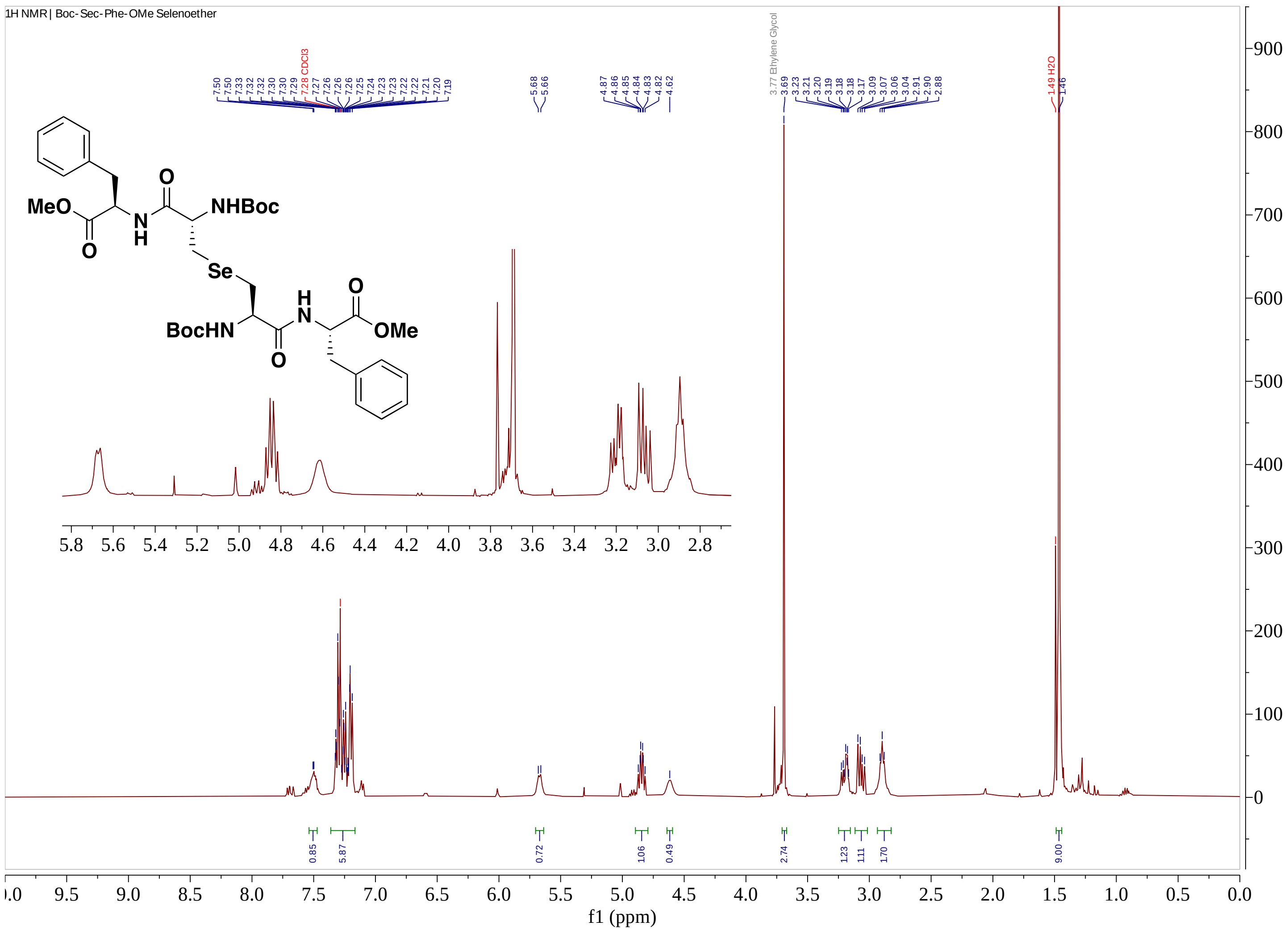

Supplementary Figure 99. ^{1}H NMR spectrum (400 MHz, $CDCl_3$) of Boc-Sec-Phe-OMe Selenoether

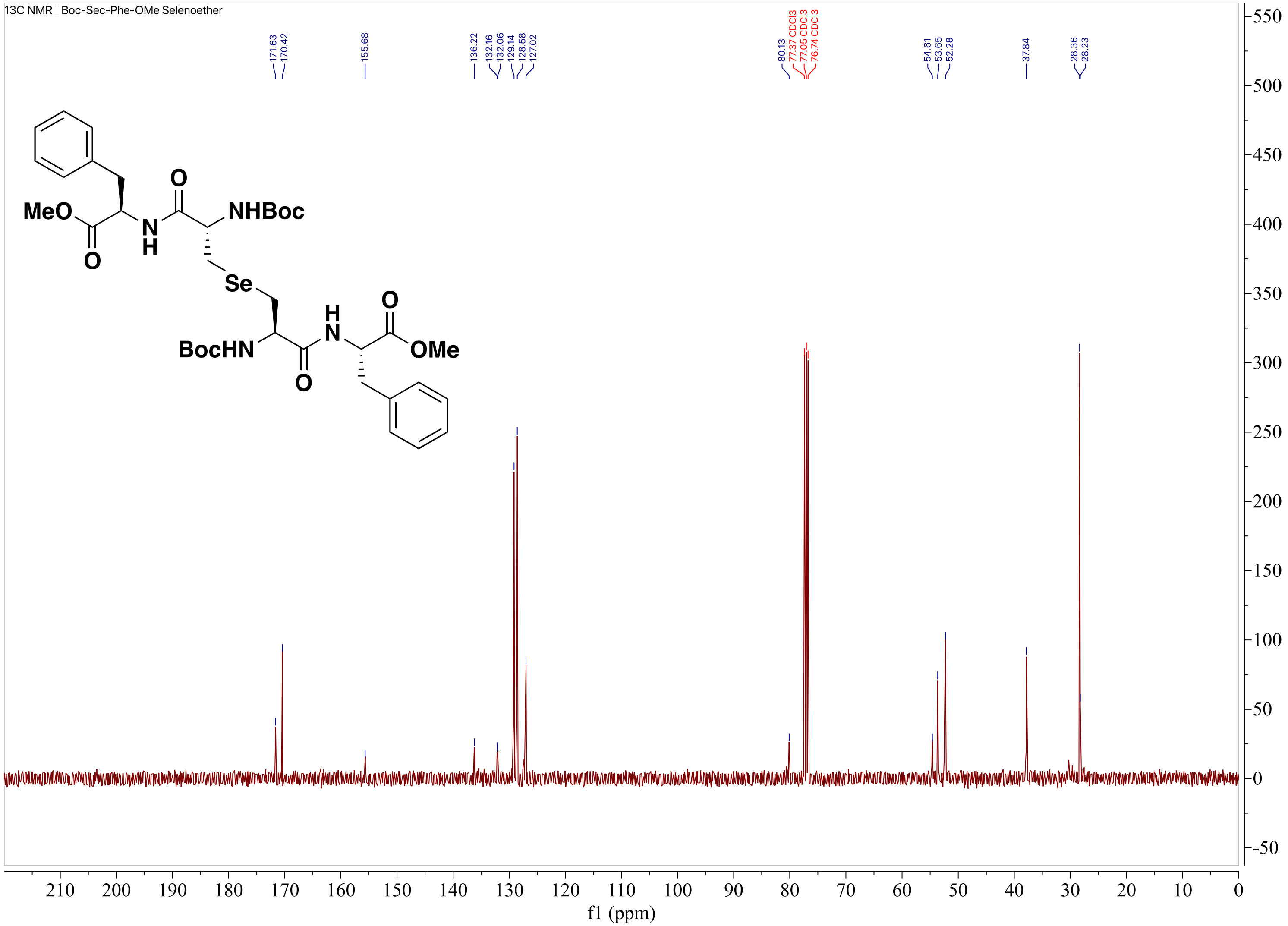

Supplementary Figure 100. ^{13}C NMR spectrum (101 MHz, $CDCl_3$) of Boc-Sec-Phe-OMe Selenoether

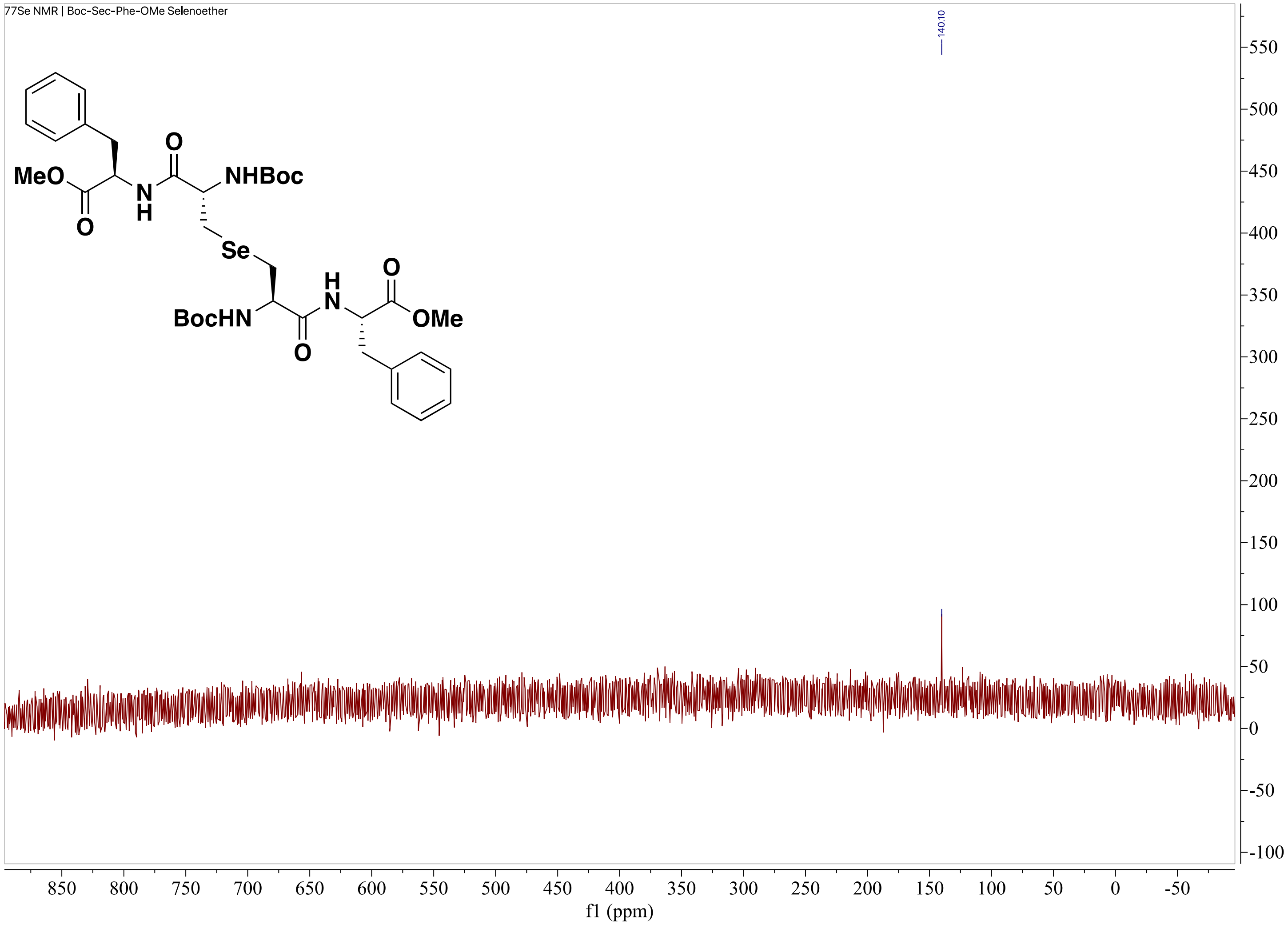

Supplementary Figure 101. ^{77}Se NMR spectrum (76 MHz, $CDCl_3$) of Boc-Sec-Phe-OMe Selenoether

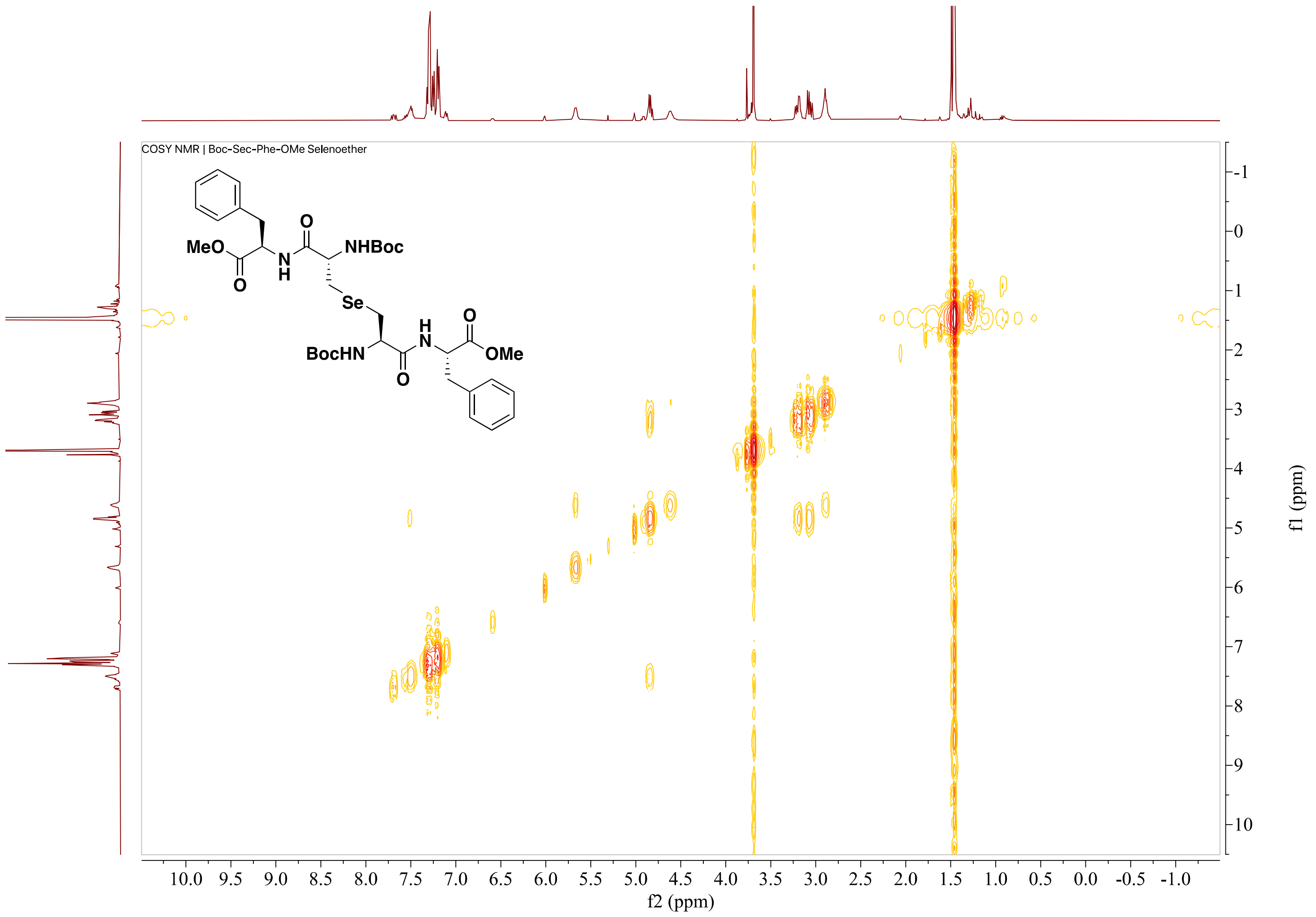

Supplementary Figure 102. COSY NMR spectrum of Boc-Sec-Phe-OMe Selenoether

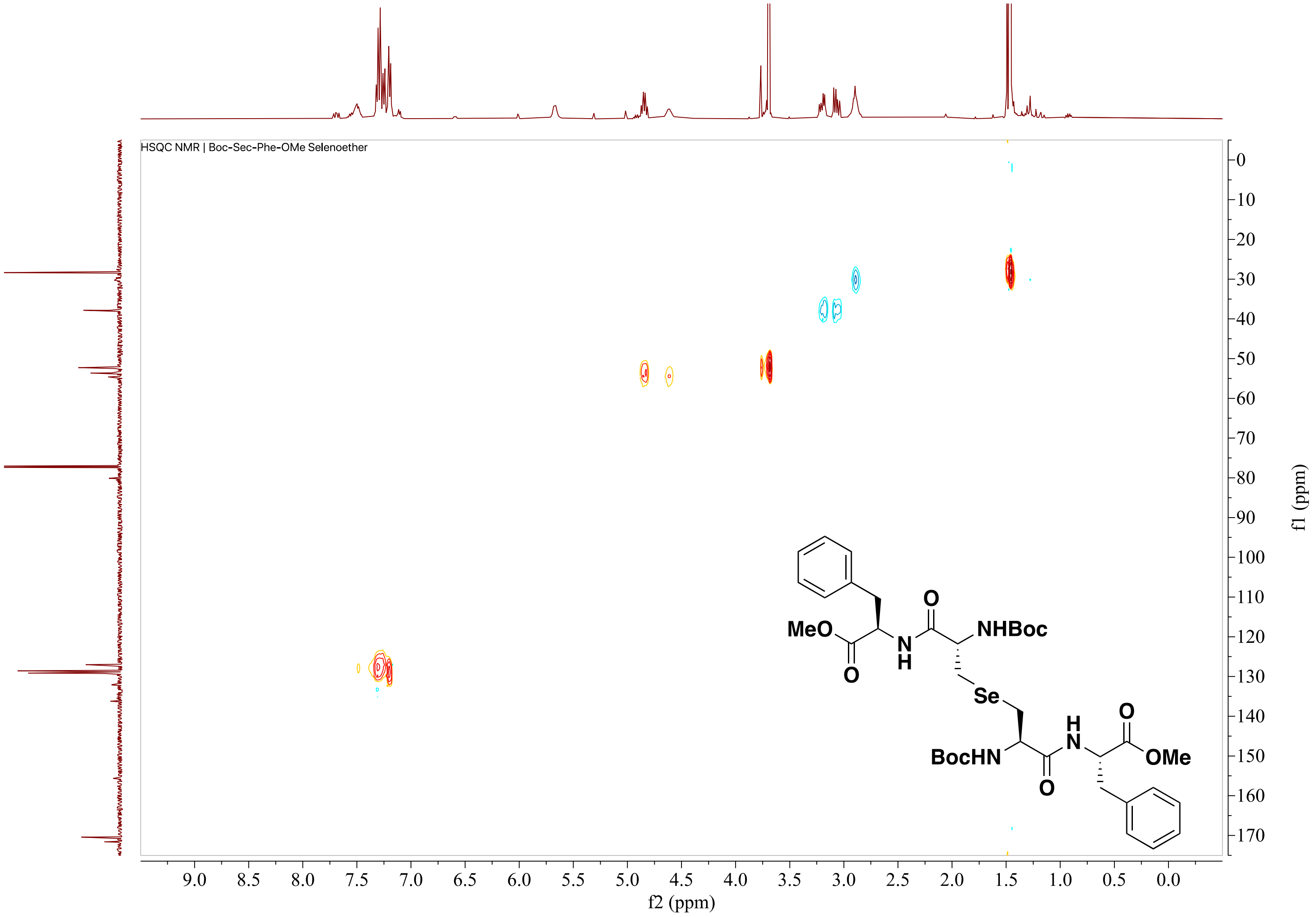

Supplementary Figure 103. HSQC NMR spectrum of Boc-Sec-Phe-OMe Selenoether

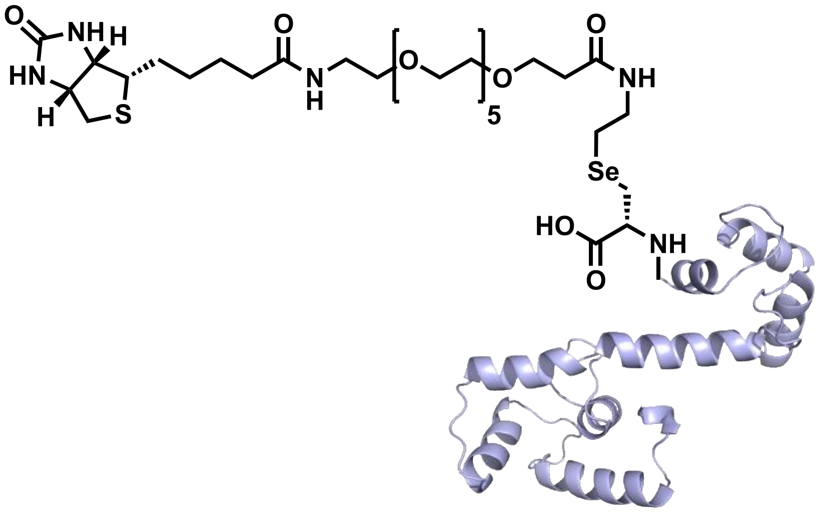

CaM (K148)-PEG$_5$-Biotin Selenoether 19

This PDC reaction was performed on CaM (K148U) diselenide **12** using conditions as described for the PEG$_6$ example above. The product **19** was afforded in 79.7 ± 0.4% conversion from the diselenide starting material **12**, as calculated through averaging integrations of HRMS-derived extracted ion chromatograms of the $[M+11H]^{11+}$, $[M+10H]^{10+}$ and $[M+9H]^{9+}$ charge states.

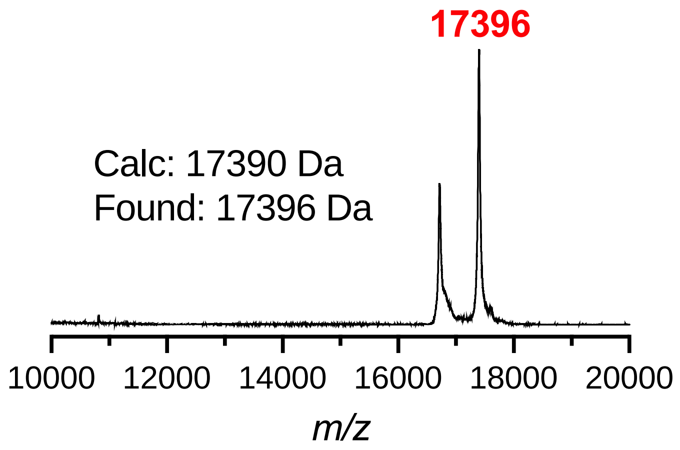

Supplementary Figure 104. MALDI-TOF MS spectrum of the crude reaction mixture of the PDC functionalization of CaM (K148U) diselenide **12** with [Se-PEG$_5$-Biotin]$_2$ after 5 min irradiation at 450 nm.

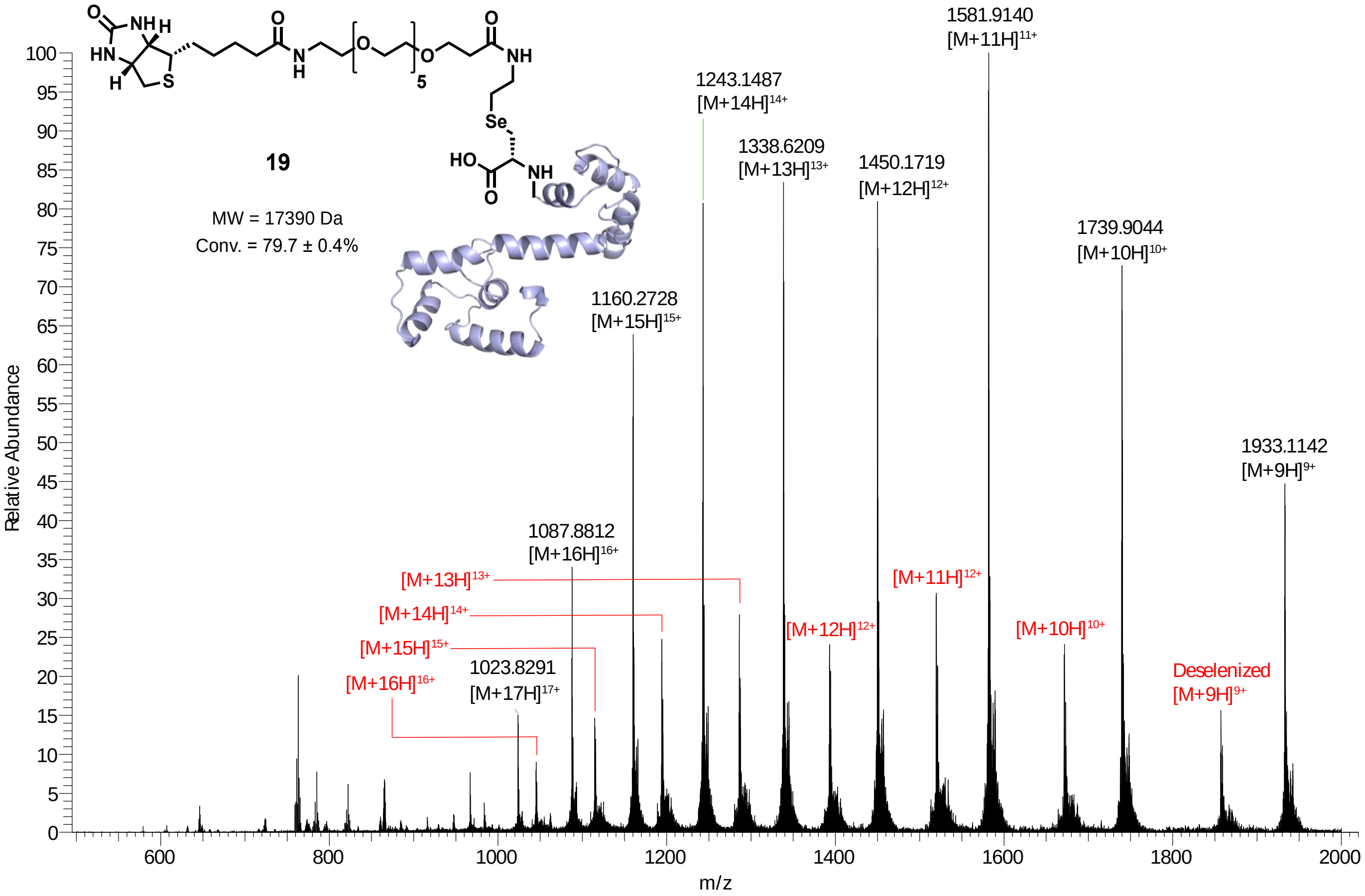

Supplementary Figure 105. HRMS spectrum of the crude reaction mixture of the PDC functionalization of CaM (K148U) diselenide **12** with [Se-PEG$_5$-Biotin]$_2$ after 5 min irradiation at 450 nm. Ion peaks for the CaM (K148U)-PEG$_5$-Biotin selenoether **19** product are labelled. Reaction conversions were calculated through averaging integrations of HRMS-derived extracted ion chromatograms of the [M+11H]$^{11+}$, [M+10H]$^{10+}$ and [M+9H]$^{9+}$ charge states and errors are reported as the standard deviation of the integration of these three ion peaks for a single experimen

.

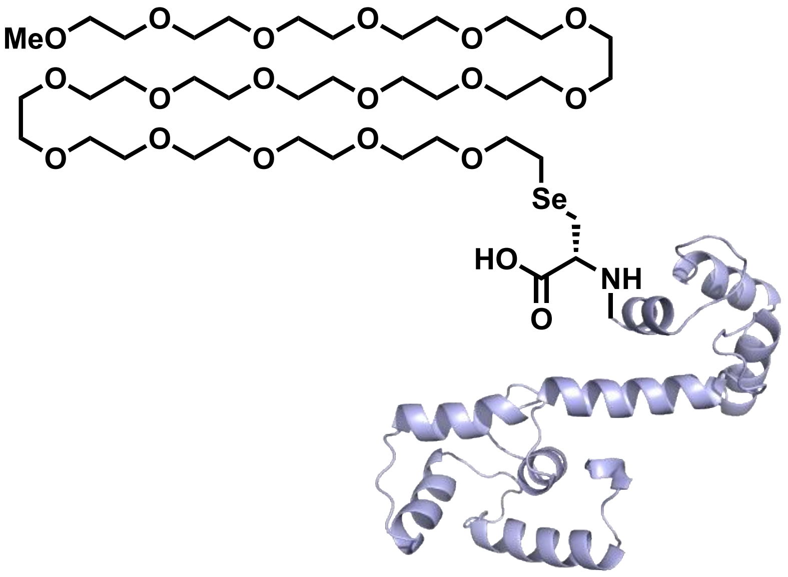

CaM (K148)-mPEG$_{17}$ Selenoether 20

This PDC reaction was performed on CaM (K148U) diselenide **12** using conditions as described for the PEG$_6$ example above. The product **20** was afforded in 84.0 ± 0.7% conversion from the diselenide starting material **12**, as calculated through averaging integrations of HRMS-derived extracted ion chromatograms of the $[M+11H]^{11+}$, $[M+10H]^{10+}$ and $[M+9H]^{9+}$ charge states.

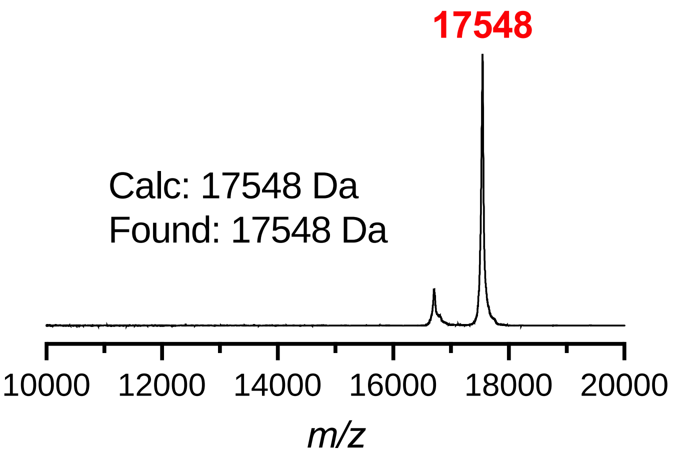

Supplementary Figure 106. MALDI-TOF MS spectrum of the crude reaction mixture of the PDC functionalization of CaM (K148U) diselenide **12** with [Se-mPEG$_{17}$]$_2$ after 5 min irradiation at 450 nm.

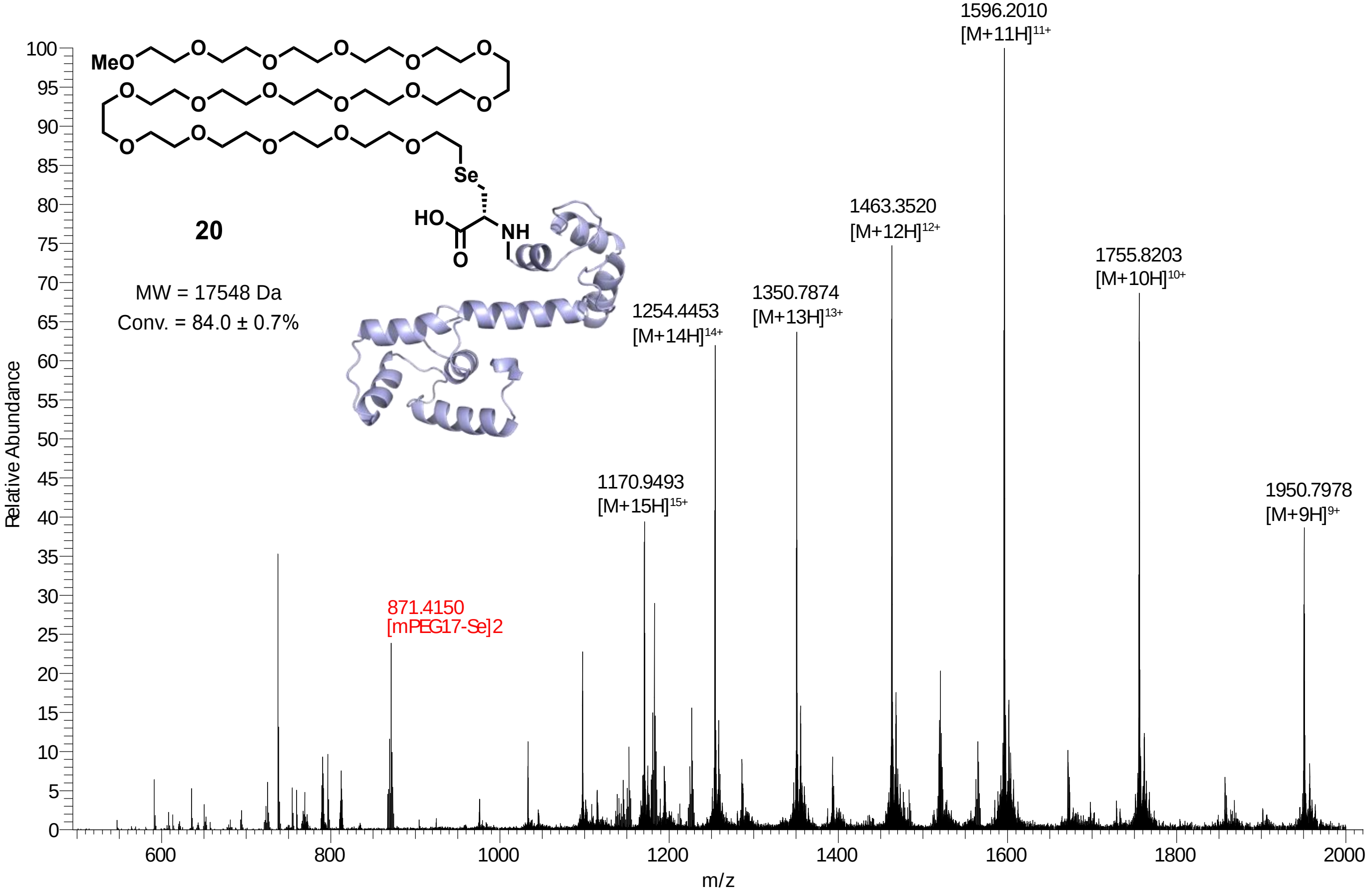

Supplementary Figure 107. HRMS spectrum of the crude reaction mixture of the PDC functionalization of CaM (K148U) diselenide **12** with [Se-mPEG$_{17}$]$_2$ after 5 min irradiation at 450 nm. Ion peaks for the CaM (K148U)-mPEG$_{17}$ selenoether **20** product are labelled. Reaction conversions were calculated through averaging integrations of HRMS-derived extracted ion chromatograms of the [M+11H]$^{11+}$, [M+10H]$^{10+}$ and [M+9H]$^{9+}$ charge states and errors are reported as the standard deviation of the integration of these three ion peaks for a single experiment.

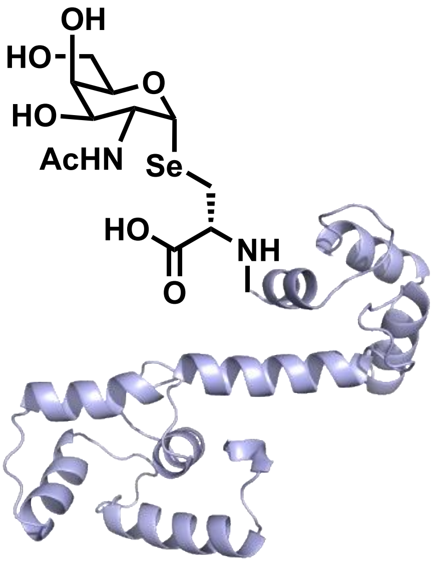

CaM (K148)-GalNAc Selenoether 21

This PDC reaction was performed on CaM (K148U) diselenide **12** using conditions as described for the PEG_6 example above. The product **21** was afforded in 90 ± 1% conversion from the diselenide starting material **12**, as calculated through averaging integrations of HRMS-derived extracted ion chromatograms of the $[M+11H]^{11+}$, $[M+10H]^{10+}$ and $[M+9H]^{9+}$ charge states.

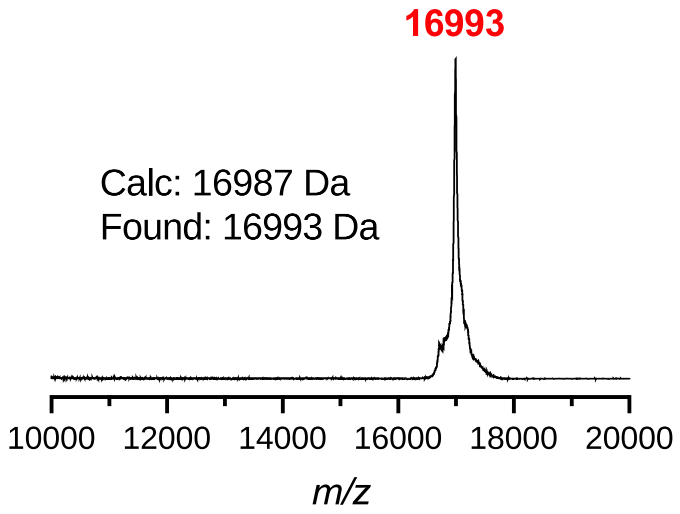

Supplementary Figure 108. MALDI-TOF MS spectrum of the crude reaction mixture of the PDC functionalization of CaM (K148U) diselenide **12** with $[Se\text{-}GalNAc]_2$ after 5 min irradiation at 450 nm.

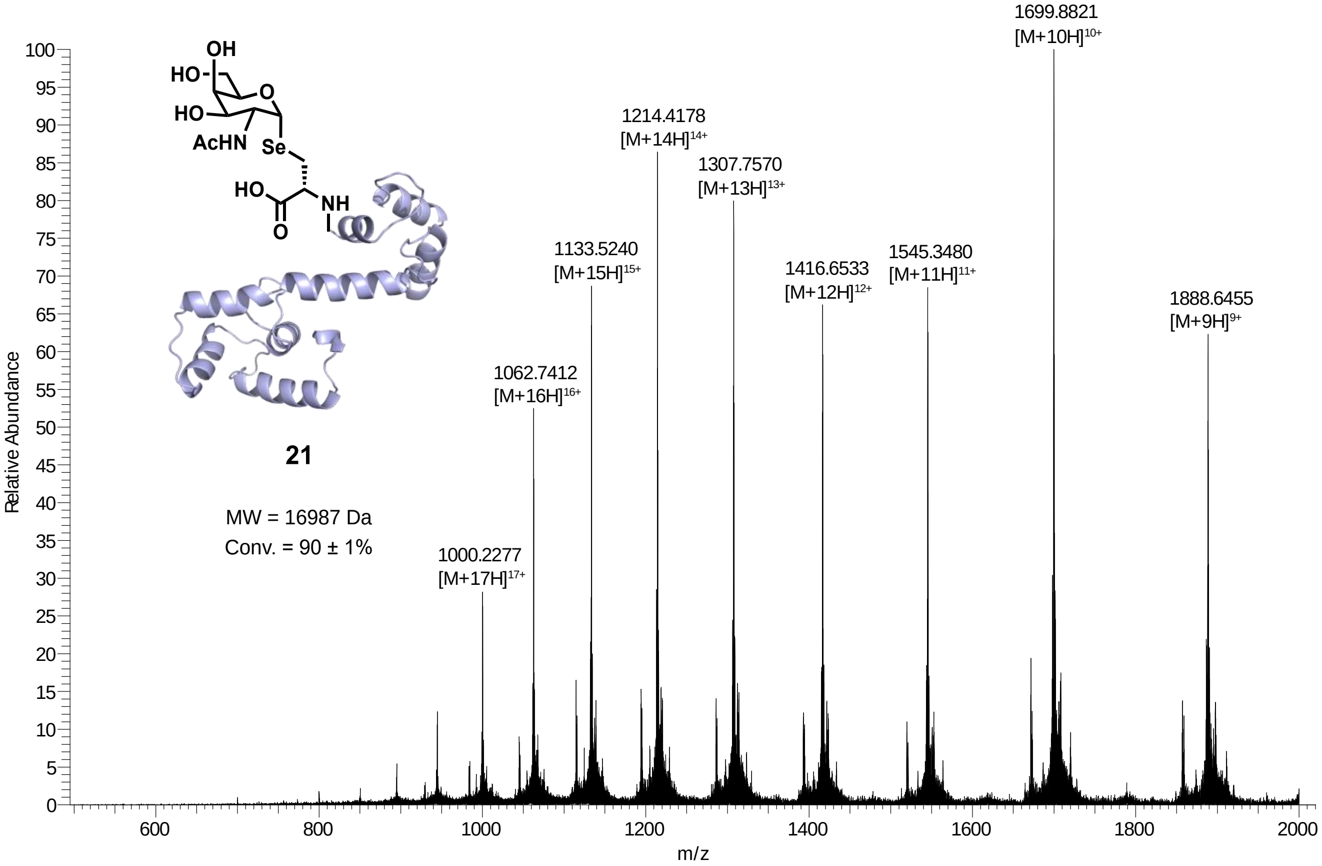

Supplementary Figure 109. HRMS spectrum of the crude reaction mixture of the PDC functionalization of CaM (K148U) diselenide **12** with [Se-GalNAc]$_2$ after 5 min irradiation at 450 nm. Ion peaks for the CaM (K148U)-GalNAc selenoether **21** product are labelled. Reaction conversions were calculated through averaging integrations of HRMS-derived extracted ion chromatograms of the $[M+11H]^{11+}$, $[M+10H]^{10+}$ and $[M+9H]^{9+}$ charge states and errors are reported as the standard deviation of the integration of these three ion peaks for a single experiment.

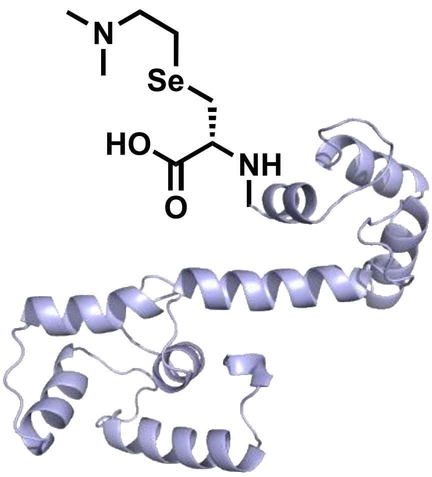

CaM (K148)-$CH_2CH_2N(Me)_2$ Selenoether 22

This PDC reaction was performed on CaM (K148U) diselenide **12** using conditions as described for the PEG_6 example above. The product **22** was afforded in 96.6 ± 1.8% conversion from the diselenide starting material **12**, as calculated through averaging integrations of HRMS-derived extracted ion chromatograms of the $[M+11H]^{11+}$, $[M+10H]^{10+}$ and $[M+9H]^{9+}$ charge states.

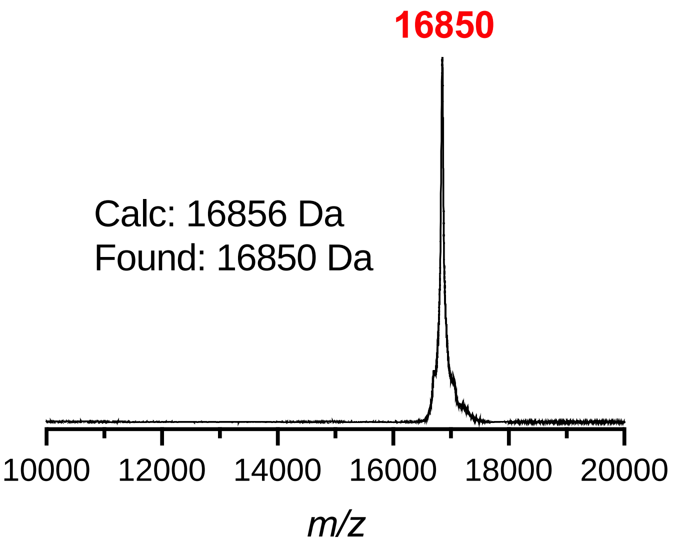

Supplementary Figure 110. MALDI-TOF MS spectrum of the crude reaction mixture of the PDC functionalization of CaM (K148U) diselenide **12** with $[Se\text{-}CH_2CH_2N(Me)_2]_2$ after 5 min irradiation at 450 nm.

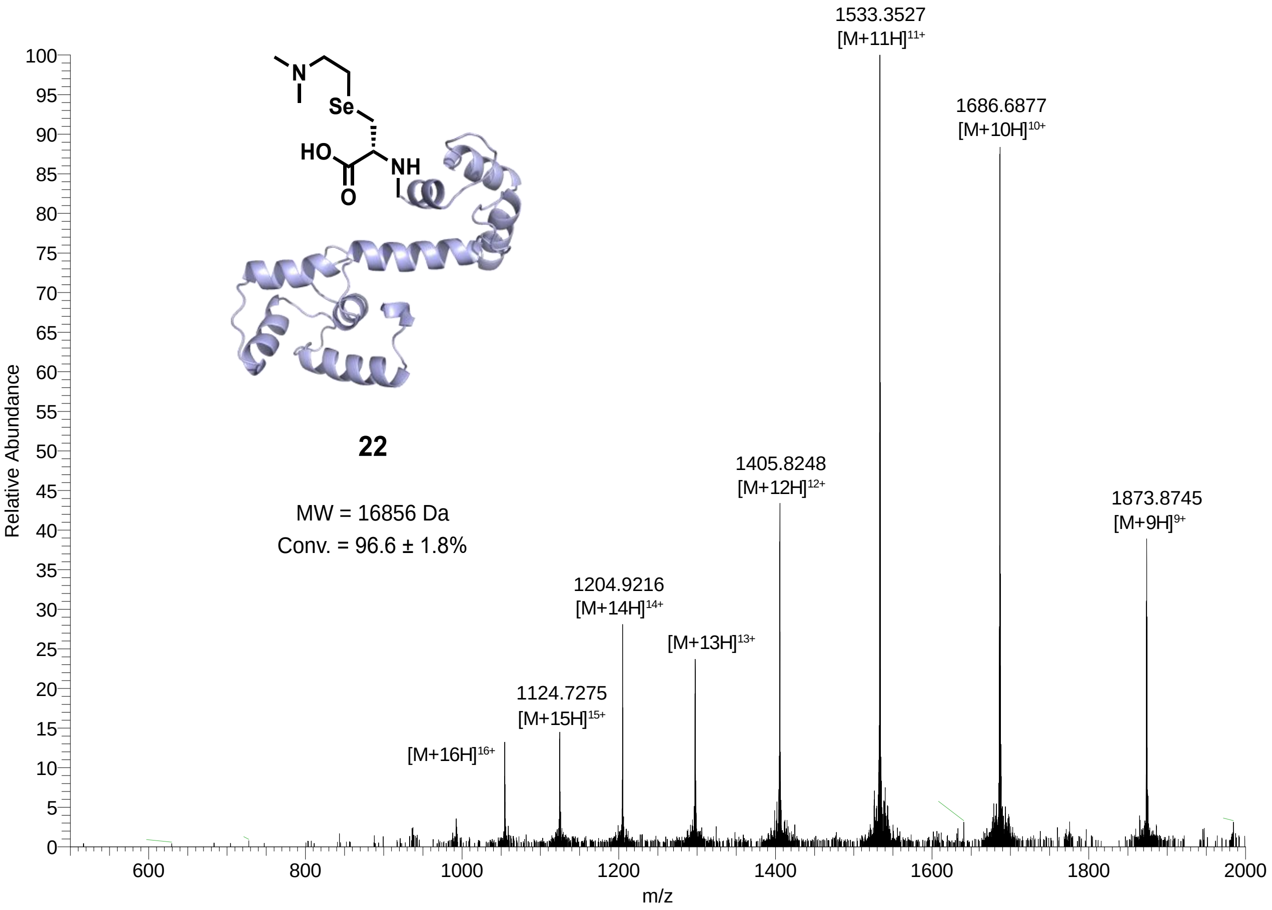

Supplementary Figure 111. HRMS spectrum of the crude reaction mixture of the PDC functionalization of CaM (K148U) diselenide **12** with [Se-$CH_2CH_2N(Me)_2$]$_2$ after 5 min irradiation at 450 nm. Ion peaks for the CaM (K148U)-$CH_2CH_2N(Me)_2$ selenoether **22** product are labelled. Reaction conversions were calculated through averaging integrations of HRMS-derived extracted ion chromatograms of the $[M+11H]^{11+}$, $[M+10H]^{10+}$ and $[M+9H]^{9+}$ charge states and errors are reported as the standard deviation of the integration of these three ion peaks for a single experiment.

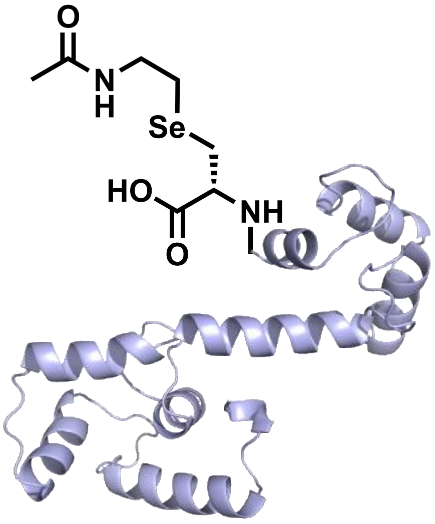

CaM (K148)-CH_2CH_2NHAc Selenoether 23

This PDC reaction was performed on CaM (K148U) diselenide **12** using conditions as described for the PEG_6 example above. The product **23** was afforded in 89.3 ± 0.9% conversion from the diselenide starting material **12**, as calculated through averaging integrations of HRMS-derived extracted ion chromatograms of the $[M+11H]^{11+}$, $[M+10H]^{10+}$ and $[M+9H]^{9+}$ charge states.

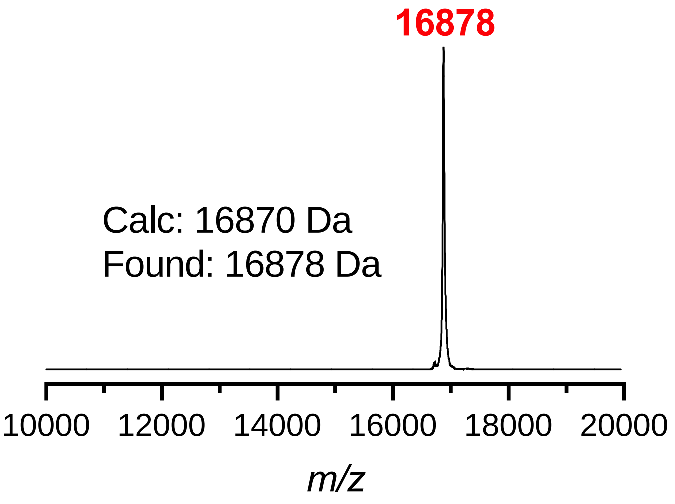

Supplementary Figure 112. MALDI-TOF MS spectrum of the crude reaction mixture of the PDC functionalization of CaM (K148U) diselenide **12** with [Se-CH_2CH_2NHAc]$_2$ after 5 min irradiation at 450 nm.

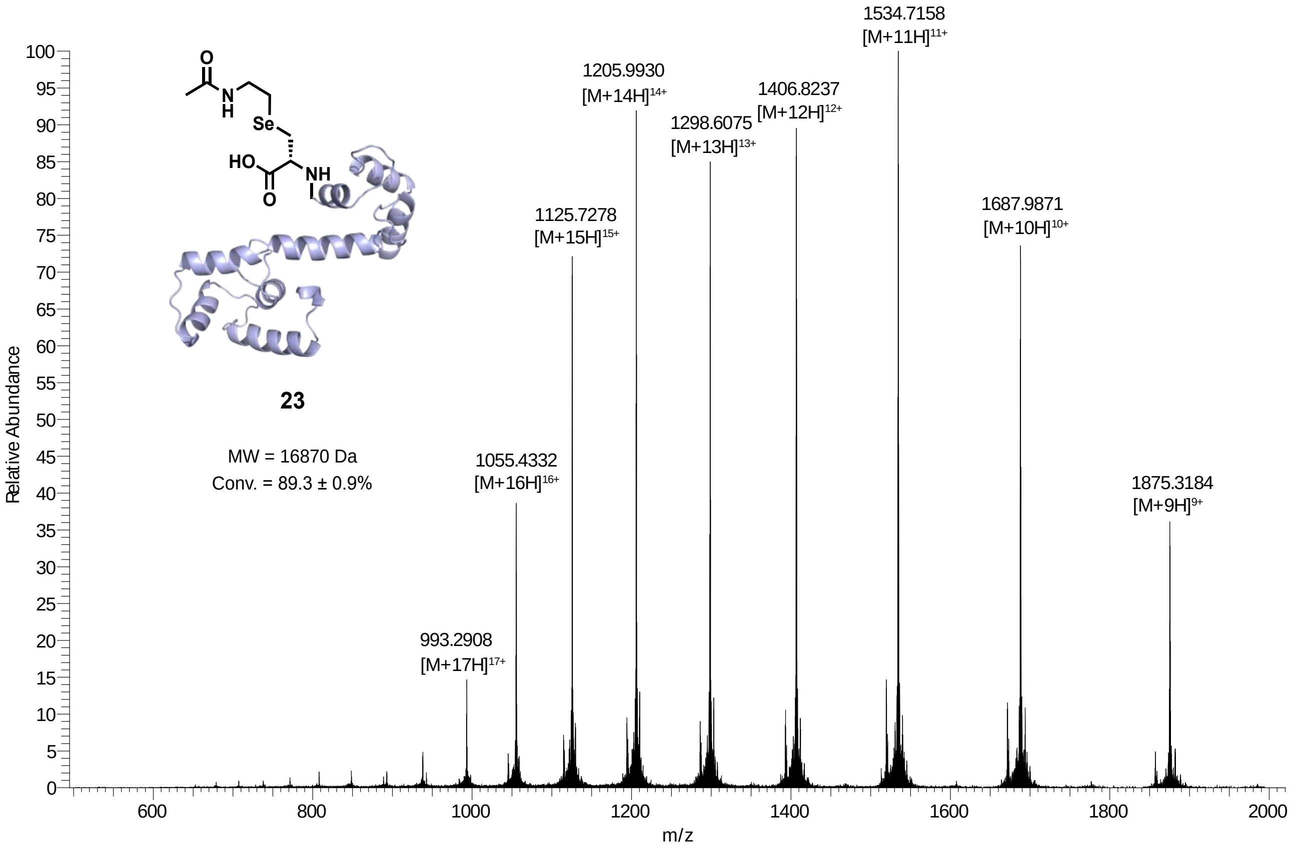

Supplementary Figure 113. HRMS spectrum of the crude reaction mixture of the PDC functionalization of CaM (K148U) diselenide **12** with [Se-CH_2CH_2NHAc]$_2$ after 5 min irradiation at 450 nm. Ion peaks for the CaM (K148U)-CH_2CH_2NHAc selenoether **23** product are labelled. Reaction conversions were calculated through averaging integrations of HRMS-derived extracted ion chromatograms of the $[M+11H]^{11+}$, $[M+10H]^{10+}$ and $[M+9H]^{9+}$ charge states and errors are reported as the standard deviation of the integration of these three ion peaks for a single experiment.

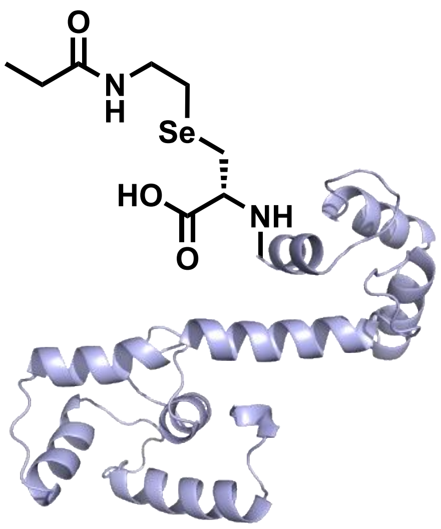

CaM (K148)-CH_2CH_2NH(Propionyl) Selenoether 24

This PDC reaction was performed on CaM (K148U) diselenide **12** using conditions as described for the PEG_6 example above. The product **24** was afforded in 80.1 ± 0.06% conversion from the diselenide starting material **12**, as calculated through averaging integrations of HRMS-derived extracted ion chromatograms of the $[M+11H]^{11+}$, $[M+10H]^{10+}$ and $[M+9H]^{9+}$ charge states.

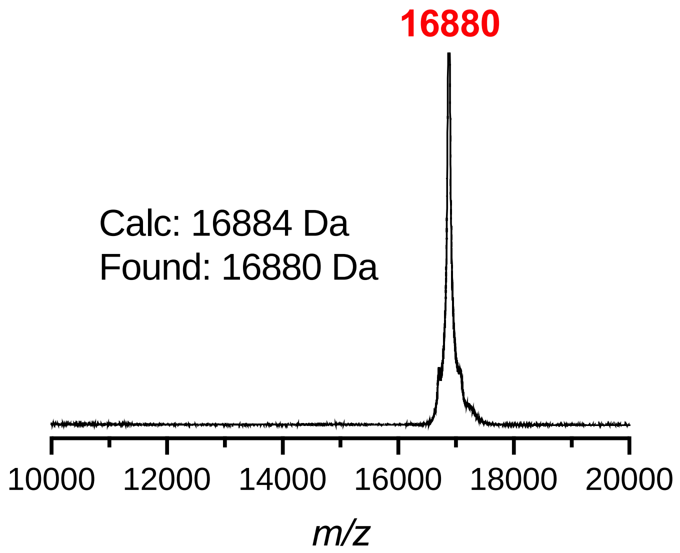

Supplementary Figure 114. MALDI-TOF MS spectrum of the crude reaction mixture of the PDC functionalization of CaM (K148U) diselenide **12** with [Se-CH_2CH_2NH(Propionyl)]$_2$ after 5 min irradiation at 450 nm.

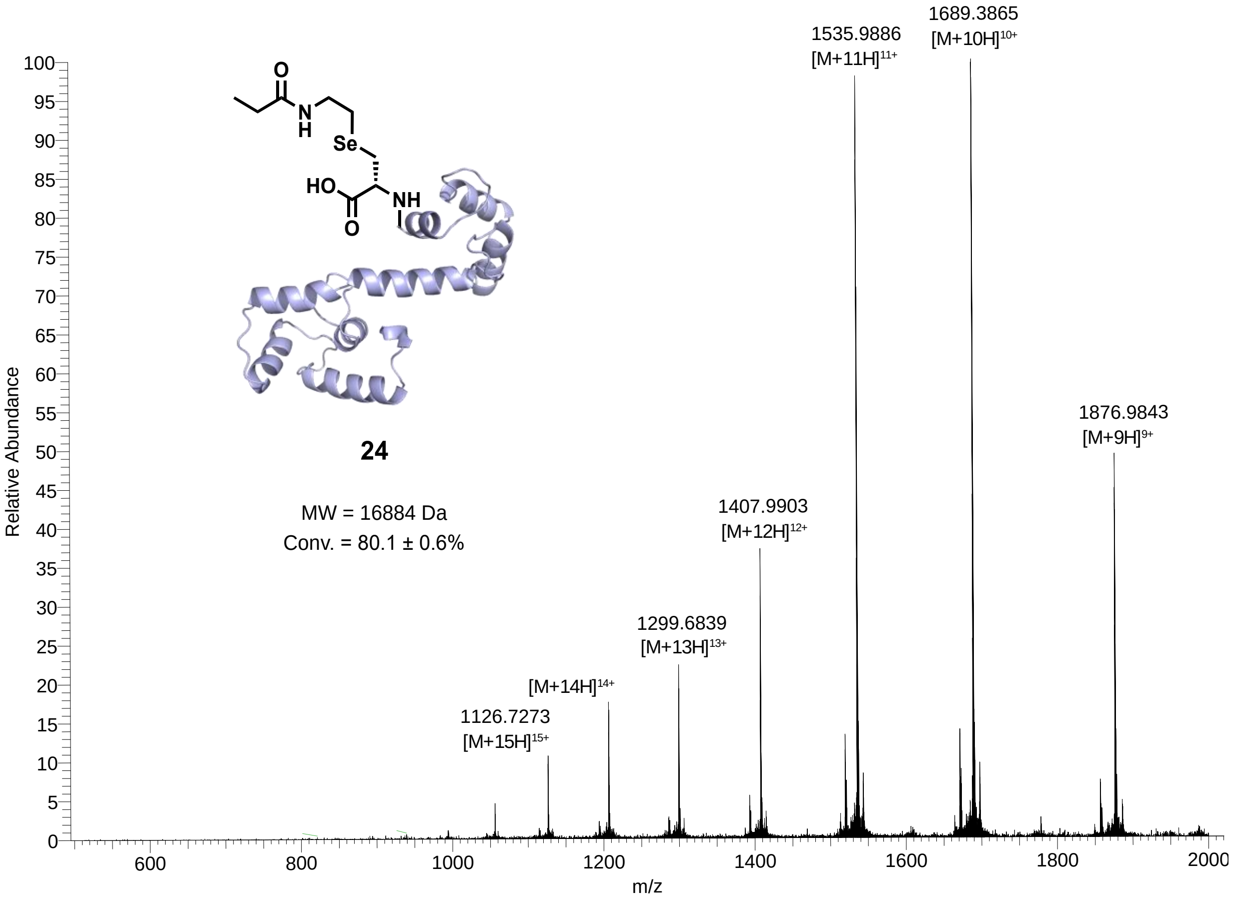

Supplementary Figure 115. HRMS spectrum of the crude reaction mixture of the PDC functionalization of CaM (K148U) diselenide **12** with [Se-CH_2CH_2NH(Propionyl)]$_2$ after 5 min irradiation at 450 nm. Ion peaks for the CaM (K148U)-CH_2CH_2NH(Propionyl) selenoether **24** product are labelled. Reaction conversions were calculated through averaging integrations of HRMS-derived extracted ion chromatograms of the $[M+11H]^{11+}$, $[M+10H]^{10+}$ and $[M+9H]^{9+}$ charge states and errors are reported as the standard deviation of the integration of these three ion peaks for a single experiment.

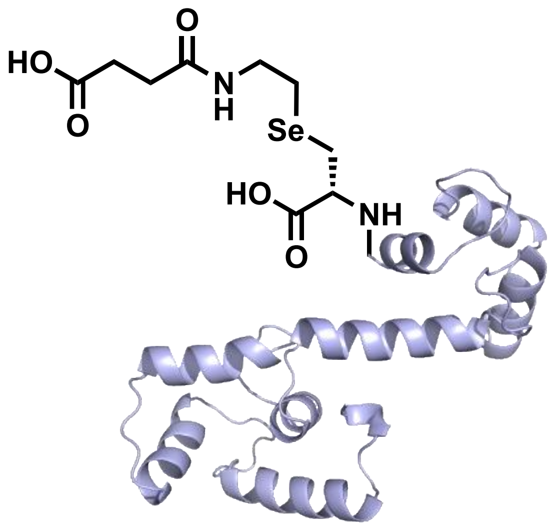

CaM (K148)-CH_2CH_2NH(Succinyl) Selenoether 25

This PDC reaction was performed on CaM (K148U) diselenide **12** using conditions as described for the PEG_6 example above. The product **25** was afforded in 89.9 ± 0.8% conversion from the diselenide starting material **12**, as calculated through averaging integrations of HRMS-derived extracted ion chromatograms of the $[M+11H]^{11+}$, $[M+10H]^{10+}$ and $[M+9H]^{9+}$ charge states.

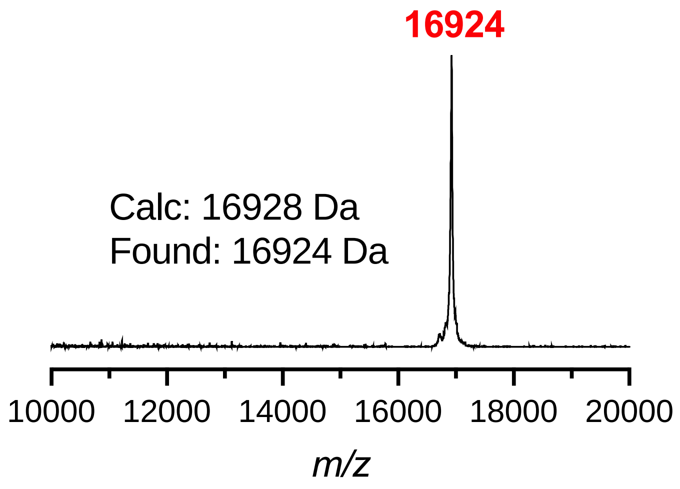

Supplementary Figure 116. MALDI-TOF MS spectrum of the crude reaction mixture of the PDC functionalization of CaM (K148U) diselenide **12** with [Se-CH_2CH_2NH(Succinyl)$]_2$ after 5 min irradiation at 450 nm.

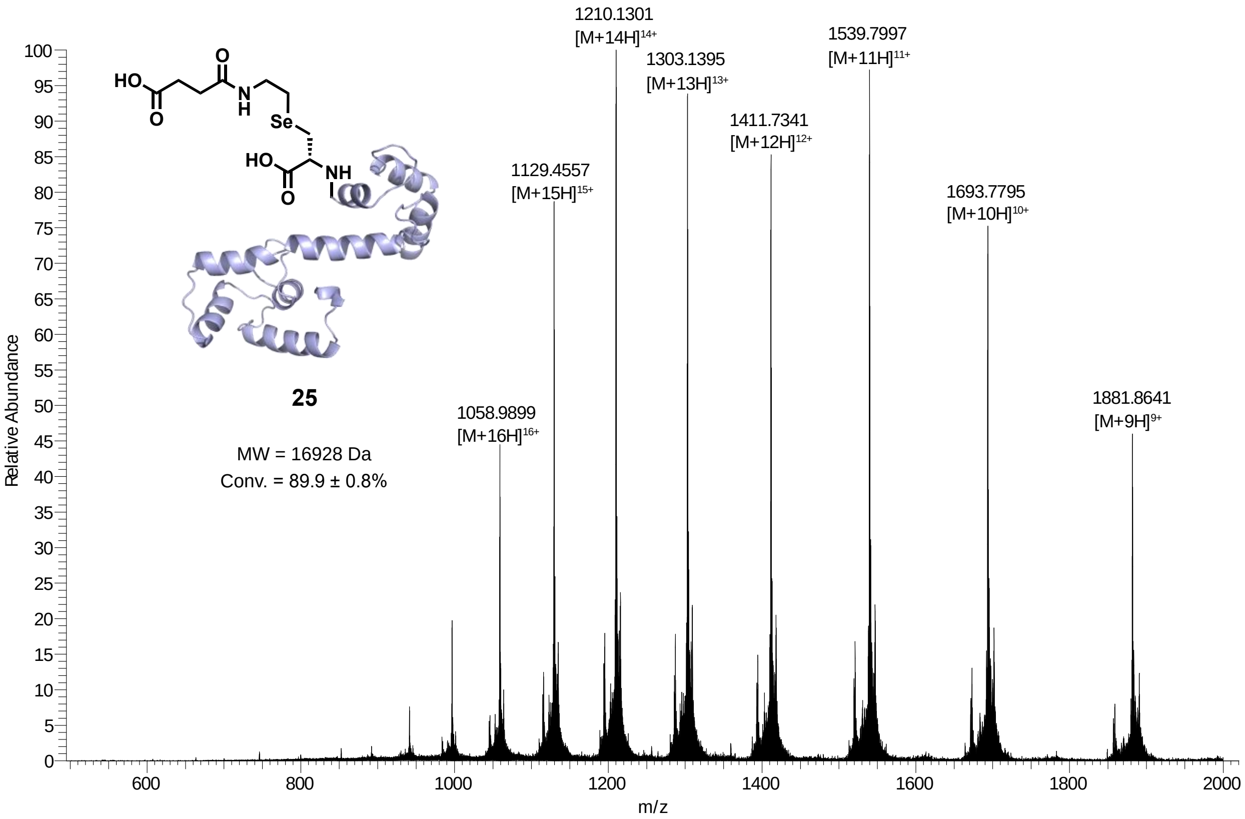

Supplementary Figure 117. HRMS spectrum of the crude reaction mixture of the PDC functionalization of CaM (K148U) diselenide **12** with [Se-CH_2CH_2NH(Succinyl)]$_2$ after 5 min irradiation at 450 nm. Ion peaks for the CaM (K148U)-CH_2CH_2NH(Succinyl) selenoether **25** product are labelled. Reaction conversions were calculated through averaging integrations of HRMS-derived extracted ion chromatograms of the $[M+11H]^{11+}$, $[M+10H]^{10+}$ and $[M+9H]^{9+}$ charge states and errors are reported as the standard deviation of the integration of these three ion peaks for a single experiment.

Bacterial Expression of Ubiquitin (Ub, Ub (K48C)) Acyl Hydrazides

The DNA sequence of human ubiquitin variants (Ub, Ub (K48C)) was amplified by PCR (primers: Fwd_Ub and Rvs_Ub-MxeSpe) (*see Primer Oligonucleotide DNA Sequences*) and subsequently cloned upstream of the *Mycobacterium xenopi* DNA Gyrase A (*Mxe* GyrA) intein, a His_7 tag and a chitin-binding domain (CBD) into a pTXB1 (New England Biolabs, MA, USA) plasmid via *Nde*I and *Spe*I restriction sites. The obtained pTXB1-Ub plasmid was used as a template to create the mutant version pTXB1-Ub-K48C by Q5 site directed mutagenesis (NEB) using the PCR primers: Fwd_K48C and Rvs_K48C (*see Primer Oligonucleotide DNA Sequences*).

Protein expression was performed using the *E. coli* BL21 Rosetta2 (Novagen) strain for Ub-K48C at 37 °C in 2YT medium (16 g L^{-1} Tryptone, 10 g L^{-1} yeast extract, 5 g L^{-1} NaCl) containing 100 µg mL^{-1} ampicillin (for Ub-K48C additional 30 µg mL^{-1} chloramphenicol was added). Overnight cultures were diluted to OD_{600} 0.2, grown until OD_{600} = 0.7 and protein overexpression was induced with 1 mM IPTG. After 2 h, the culture was centrifuged for 20 min at 10,000 x g, the cell pellets were resuspended in TBS buffer (50 mM Tris, 150 mM NaCl, pH 7) and lysed twice in a high-pressure cell disrupter (Constant Systems, UK). The lysate was centrifuged for 30 min at 50,000 x g. Each 45 mL supernatant was incubated with 5 mL TBS-equilibrated chitin resin (NEB) for 2 h. The resin was washed twice with 45 mL TBS and subsequently incubated with 30 mL NH_2NH_2 solution (5 vol% NH_2NH_2 in TBS, 50 mM DTT) for 2 days to produce the Ub acyl hydrazide **26**. After cleavage, the supernatant was separated from the chitin resin by centrifugation, filtered and purified by RP-HPLC on a C4 column (5-30% B over 5 min then 30-70% B over 40 min, 0.1 vol% TFA, flow rate: 10 mL min^{-1}).

See next page for analytical data.

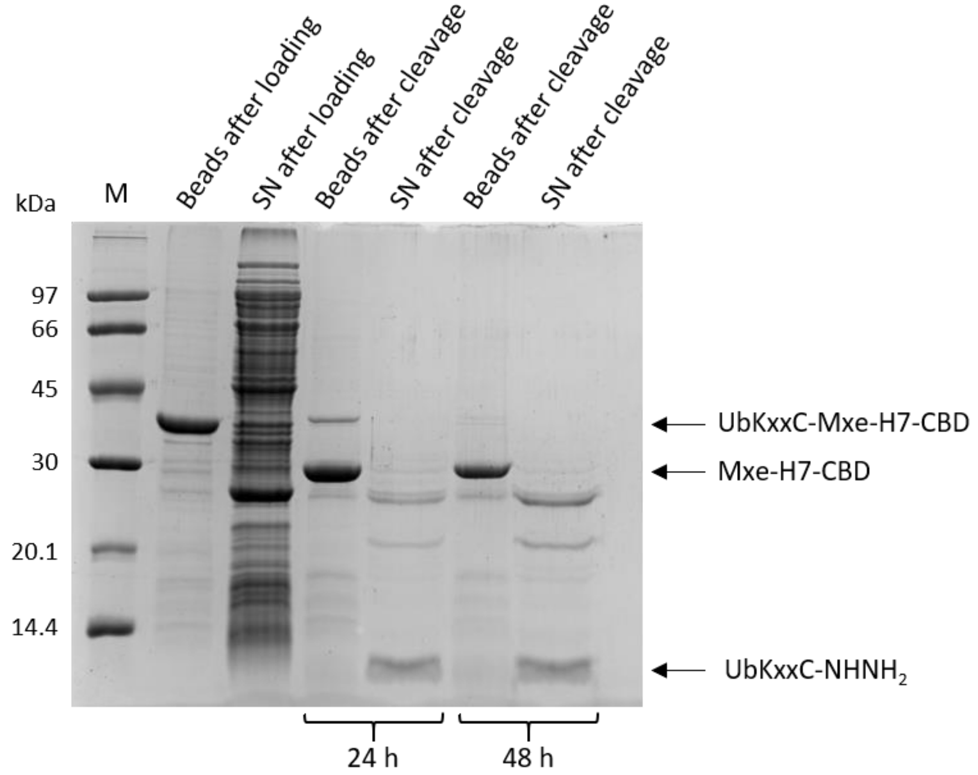

Supplementary Figure 118. Representative SDS-PAGE analysis of Ub (K48C) (1-76) cleavage from intein fusion construct with 5 vol% NH_2NH_2 (xx = 48) from a single large scale expression.

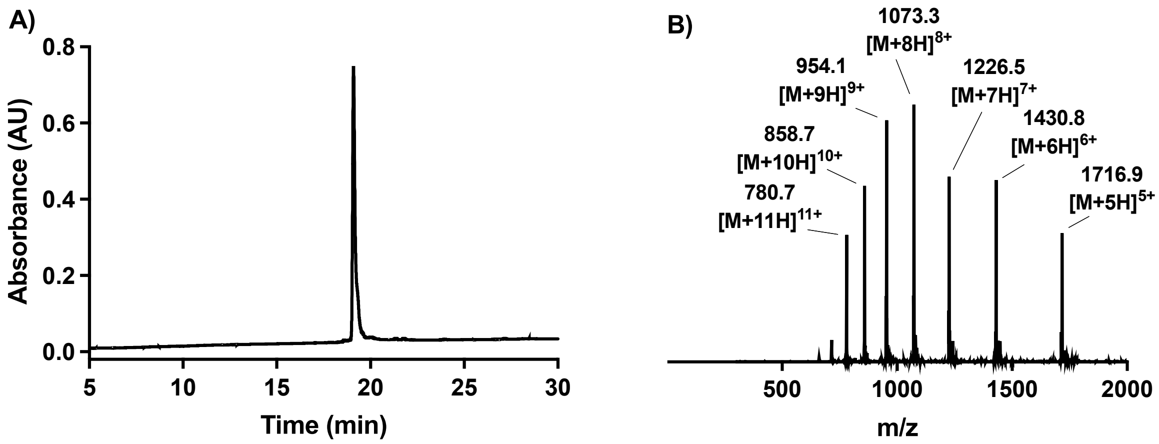

Supplementary Figure 119. A) Analytical HPLC trace of expressed Ubiquitin-$NHNH_2$ (**26**), R_t 19.1 min (1 to 50% B over 30 min, 0.1% v/v TFA, λ = 214 nm); **B)** ESI-MS of pure **26**; Calculated Mass: $[M+5H]^{5+}$: 1716.8; $[M+6H]^{6+}$: 1430.8; $[M+7H]^{7+}$: 1226.6; $[M+8H]^{8+}$: 1073.4; $[M+9H]^{9+}$: 954.2; $[M+10H]^{10+}$: 858.9; $[M+11H]^{11+}$: 780.9. Mass Found (ESI+) 1716.9 $[M+5H]^{5+}$; 1430.8 $[M+6H]^{6+}$; 1226.5 $[M+7H]^{7+}$; 1073.3 $[M+8H]^{8+}$; 954.1 $[M+9H]^{9+}$; 858.7 $[M+10H]^{10+}$; 780.7 $[M+11H]^{11+}$. ESI-MS data was collected over the entire gradient and wash cycle of the UPLC-MS.

One-pot C-terminal Selenoesterification of Ubiquitin (26) and Selenocystamine Fusion

A buffer solution containing 200 mM TCEP, 200 mM HEPES, 50 mM DPDS and 6 M Gnd•HCl was freshly prepared in MilliQ water and the pH was adjusted to 2.0-2.5 using 5 M aqueous HCl, followed by sparging of the solution with Ar for 15-20 min. The recombinantly expressed ubiquitin acyl hydrazide **26** (14.4 mg, 1.68 µmol) was dissolved in the buffer (1 mM final concentration, final pH = 2.0), followed by the addition of 5 eq. of acetylacetone (acac) from a 150 mM aqueous stock. The solution was then allowed to stir at room temperature under an atmosphere of argon for 2-3 h and completion of the selenoesterification reaction was monitored by UPLC-MS. Without purification, selenocystamine (2.7 mg, 83.9 µmol, 5 eq.) was added to the crude reaction mixture and the pH was carefully adjusted to 5.0 using 5 M NaOH solution. Complete conversion of Ubiquitin(1-76)-SePh to the corresponding selenocystamine functionalized ubiquitin **27** was observed in 20 min (based on UPLC-MS analysis). The residual DPDS was extracted using Et_2O (5 × 1 mL) and the crude reaction mixture was subjected to RP-HPLC purification (C18 Sunfire column, 5 µm, 19 × 150 mm, 0 to 50% B over 50 min, 0.1% v/v TFA) followed by lyophilization to afford the purified Ubiquitin-selenocystamine diselenide **27** as a white fluffy solid (9.9 mg, 1.14 µmol, 68% yield over 2 steps).

See next page for analytical data.

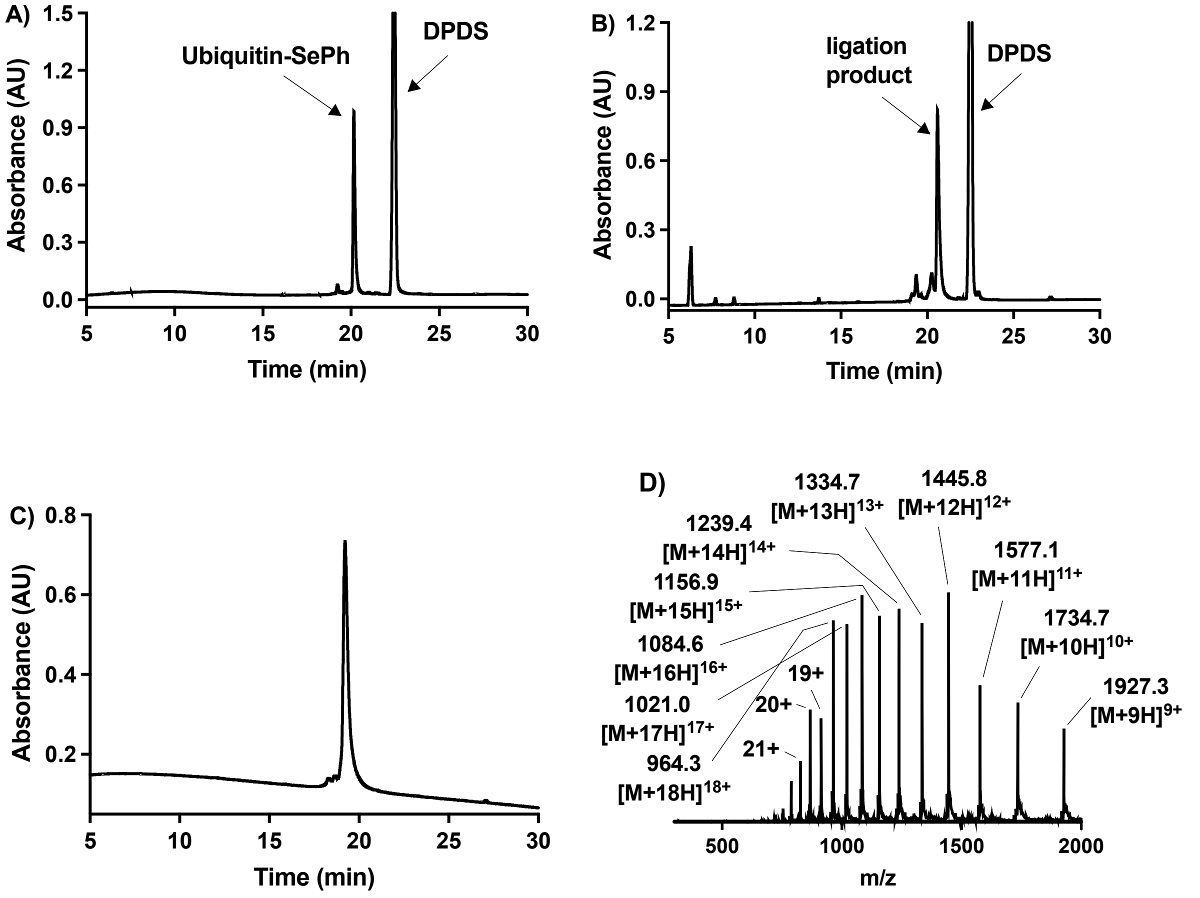

Supplementary Figure 120. A) Analytical HPLC trace of crude Ubiquitin-SePh selenoester intermediate, R_t 20.2 min (1 to 50% B over 30 min, 0.1% v/v TFA, λ = 214 nm); **B)** Analytical HPLC trace of crude Ubiquitin diselenide **27**, R_t 20.6 min (1 to 50% B over 30 min, 0.1% v/v TFA, λ = 214 nm)**; C)** Analytical HPLC trace of RP-HPLC purified Ubiquitin diselenide **27**, R_t 19.3 min (1 to 50% B over 30 min, 0.1% v/v TFA, λ = 214 nm); **D)** ESI-MS of pure **27**; Calculated Mass: $[M+9H]^{9+}$: 1927.6; $[M+10H]^{10+}$: 1735.0; $[M+11H]^{11+}$: 1577.4; $[M+12H]^{12+}$: 1446.0; $[M+13H]^{13+}$: 1334.8; $[M+14H]^{14+}$: 1239.6; $[M+15H]^{15+}$: 1157.0; $[M+16H]^{16+}$: 1084.8; $[M+17H]^{17+}$: 1021.0; $[M+18H]^{18+}$: 964.3; $[M+19H]^{19+}$: 913.6; $[M+20H]^{20+}$: 868.0; $[M+21H]^{21+}$: 826.7; Mass Found (ESI+) 1927.3 $[M+9H]^{9+}$; 1734.7 $[M+10H]^{10+}$; 1577.1 $[M+11H]^{11+}$; 1445.8 $[M+12H]^{12+}$; 1334.7 $[M+13H]^{13+}$; 1239.4 $[M+14H]^{14+}$; 1156.9 $[M+15H]^{15+}$; 1084.6 $[M+16H]^{16+}$; 1021.0 $[M+17H]^{17+}$; 964.3 $[M+18H]^{18+}$; 913.6 $[M+19H]^{19+}$; 868.0 $[M+20H]^{20+}$; 826.7 $[M+21H]^{21+}$. ESI-MS data was collected over the entire gradient and wash cycle of the UPLC-MS.

One-pot C-terminal Selenoesterification of Ubiquitin (K48C) Selenoesterification and Selenocystamine Fusion

Ubiquitin-$NHNH_2$ (K48C) (4 mg) was subjected to the same conditions described above for native Ubiquitin to obtain corresponding Ubiquitin (K48C) as a mixture of selenyl sulfide (monomer) and the diselenide (dimer) in 37% yield (1.5 mg) after RP-HPLC purification.

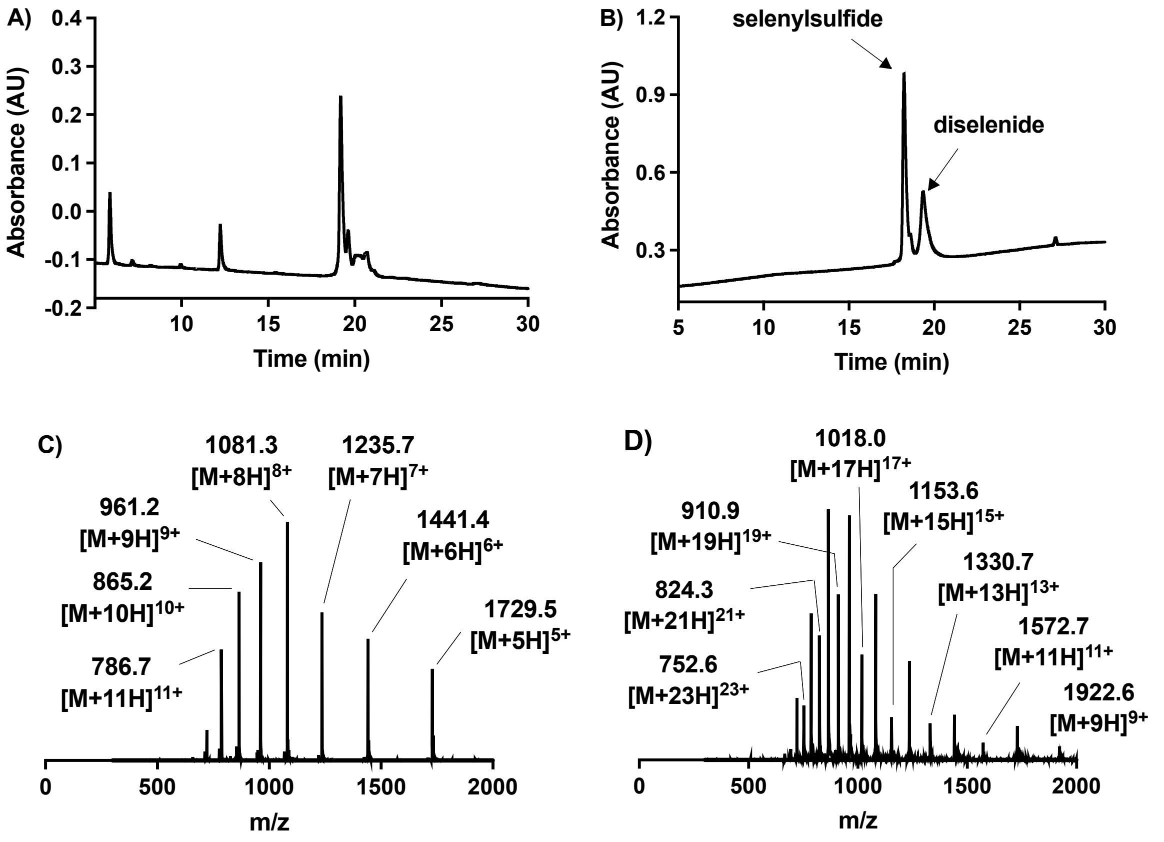

Supplementary Figure 121. A) Analytical HPLC trace of crude Ubiquitin (K48C) diselenide, R_t 19.2 min (1 to 50% B over 30 min, 0.1% v/v TFA, λ = 214 nm); **B)** Analytical HPLC trace of RP-HPLC purified Ubiquitin selanyl sulfide + diselenide, R_t 18.3 min (selanyl sulfide); R_t 19.4 min (diselenide) (1 to 50% B over 30 min, 0.1% v/v TFA, λ = 214 nm); **C)** ESI-MS of RP-HPLC purified Ubiquitin selanyl sulfide; Calculated Mass: $[M+5H]^{5+}$: 1729.8; $[M+6H]^{6+}$: 1441.7; $[M+7H]^{7+}$: 1235.9; $[M+8H]^{8+}$: 1081.5; $[M+9H]^{9+}$: 961.4; $[M+10H]^{10+}$: 865.4; $[M+11H]^{11+}$: 786.8; Mass Found (ESI+) 1729.5 $[M+5H]^{5+}$; 1441.4 $[M+6H]^{6+}$; 1235.7 $[M+7H]^{7+}$; 1081.3 $[M+8H]^{8+}$; 961.2 $[M+9H]^{9+}$; 865.2 $[M+10H]^{10+}$; 786.7 $[M+11H]^{11}$; **D)** ESI-MS of Ubiquitin (K48C) diselenide; Calculated Mass: $[M+9H]^{9+}$: 1922.1; $[M+10H]^{10+}$: 1730.0; $[M+11H]^{11+}$: 1572.8; $[M+12H]^{12+}$: 1441.8; $[M+13H]^{13+}$: 1331.0; $[M+14H]^{14+}$: 1236.0; $[M+15H]^{15+}$: 1153.7; $[M+16H]^{16+}$: 1081.6; $[M+17H]^{17+}$: 1018.1; $[M+18H]^{18+}$: 961.6; $[M+19H]^{19+}$: 911.0; $[M+20H]^{20+}$: 865.5; $[M+21H]^{21+}$: 824.3; $[M+22H]^{22+}$: 786.9; $[M+23H]^{23+}$: 752.7; Mass Found (ESI+) 1922.6 $[M+9H]^{9+}$; 1729.9 $[M+10H]^{10+}$; 1572.7 $[M+11H]^{11+}$; 1441.6 $[M+12H]^{12+}$; 1330.7 $[M+13H]^{13+}$; 1235.9 $[M+14H]^{14+}$; 1153.6 $[M+15H]^{15+}$; 1081.4 $[M+16H]^{16+}$; 1018.0

$[M+17H]^{17+}$; 961.6 $[M+18H]^{18+}$; 910.9 $[M+19H]^{19+}$; 865.5 $[M+20H]^{20+}$; 824.3 $[M+21H]^{21+}$; 786.9 $[M+22H]^{22+}$; 752.6 $[M+23H]^{23+}$.

Late-Stage C-Terminal PDC Functionalization of Ubiquitin Diselenide 27

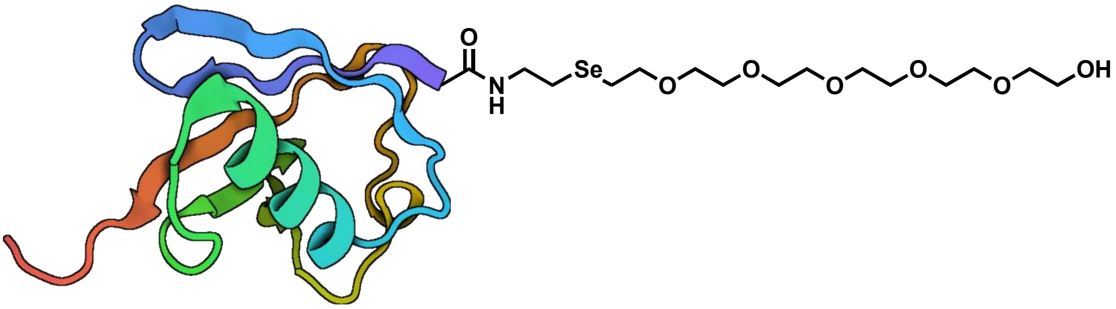

Ubiquitin-PEG$_6$ Selenoether 28

In a 1.5 mL Protein LoBind (Eppendorf, Hamburg, Germany) microcentrifuge tube, Ubiquitin diselenide **27** (1.5 mg, 74.7 nmol), PEG$_6$ diselenide (1.65 mg, 2.39 µmol, 32 eq.), PTA (**4**) (0.77 mg, 4.93 µmol, 66 eq.) and [Ir(dF(CF$_3$)ppy)$_2$(dtbpy)]PF$_6$ (**5**) (10 mol% relative to **27**) [25 µL from a 0.3 µmol mL^{-1} stock in 1:1 v/v MeCN:H_2O] and were dissolved in 125 µL of 50 vol% MeCN in 1 M Gnd.HCl, 0.02 M NaP$_i$, pH 7.0 buffer under a stream of N_2 to achieve Ubiquitin diselenide **27** concentration of 0.5 mM. The reaction tube was capped and immediately irradiated with 450 nm LED light (PennOC M1 photoreactor) at 37 ^oC for 5 min. Two 5 µL aliquots were taken at 0 min and 5 min time points and individually diluted 4-fold in 6 M Gnd.HCl, 0.1 M NaP$_i$, pH 7.0 buffer (15 µL). One aliquot was desalted using a C18 ZipTip (Merck, Darmstadt, Germany) and analyzed by MALDI-TOF MS (*see General Procedures*). The other diluted aliquot was directly analyzed by LC-HRMS (*see General Procedures*). Reaction conversion was calculated through averaging integrations of HRMS-derived extracted ion chromatograms of the $[M+9H]^{9+}$, $[M+8H]^{8+}$ and $[M+7H]^{7+}$ charge states. Conversion errors are reported as the standard deviation of these three ion peak integrations for a single experiment.

To obtain an isolated yield, the same experiment was repeated and after 5 min irradiation the crude reaction mixture was diluted with 6 M Gnd.HCl, 0.1 M NaP$_i$, pH 7.0 buffer (1 mL) and subjected directly to RP-HPLC purification to afford the purified Ubiquitin-PEG$_6$ selenoether **28** in 40% yield (0.62 mg). *See next pages for analytical data.*

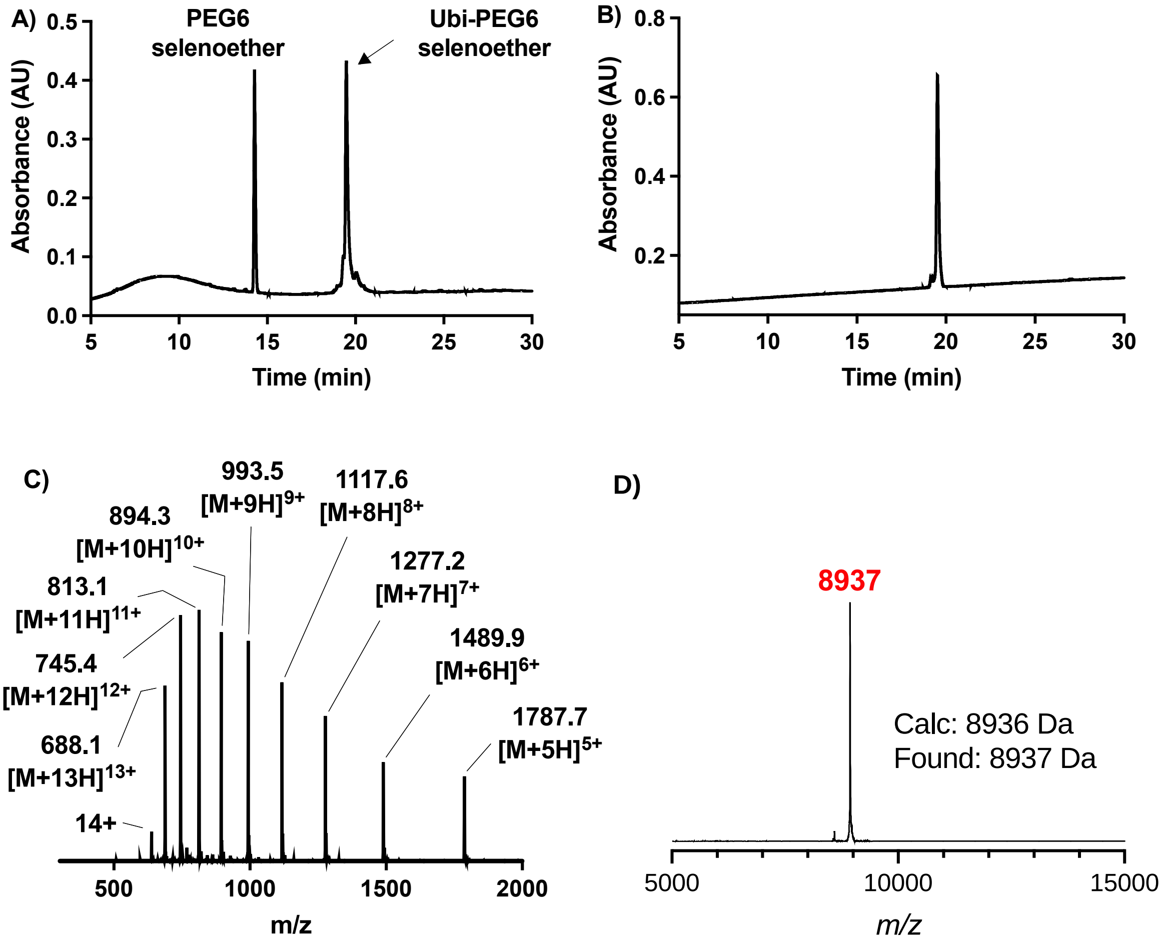

Supplementary Figure 122. A) Crude analytical HPLC trace of PDC reaction between Ubiquitin diselenide and PEG_6 diselenide after 5 min; R_t 19.5 min (1-50%B over 30 min, 0.1% v/v TFA, λ = 214 nm); **B)** Analytical HPLC trace of RP-HPLC purified Ubiquitn-PEG_6 selenoether **28**; R_t 19.5 min (1-50%B over 30 min, 0.1% v/v TFA, λ = 214 nm); **C)** ESI-MS of Ubiquitin-PEG_6 selenoether **28**; Calculated Mass: $[M+5H]^{5+}$: 1788.2; $[M+6H]^{6+}$: 1490.3; $[M+7H]^{7+}$: 1277.6; $[M+8H]^{8+}$: 1118.0; $[M+9H]^{9+}$: 993.9; $[M+10H]^{10+}$: 894.6; $[M+11H]^{11+}$: 813.4; $[M+12H]^{12+}$: 745.7; $[M+13H]^{13+}$: 688.4; $[M+14H]^{14+}$: 639.3; Mass Found (ESI+) 1787.7 $[M+5H]^{5+}$; 1489.9 $[M+6H]^{6+}$; 1277.2 $[M+7H]^{7+}$; 1117.6 $[M+8H]^{8+}$; 993.5 $[M+9H]^{9+}$; 894.3 $[M+10H]^{10+}$; 813.1 $[M+11H]^{11+}$; 745.4 $[M+12H]^{12+}$; 688.1 $[M+13H]^{13+}$; 639.0 $[M+14H]^{14+}$. ESI-MS data was collected over the entire gradient and wash cycle of the UPLC-MS. **D)** MALDI-TOF MS spectrum of the crude reaction mixture of the PDC functionalization of Ub diselenide **27** with $[Se\text{-}PEG_6]_2$ (**16**) after 5 min irradiation at 450 nm.

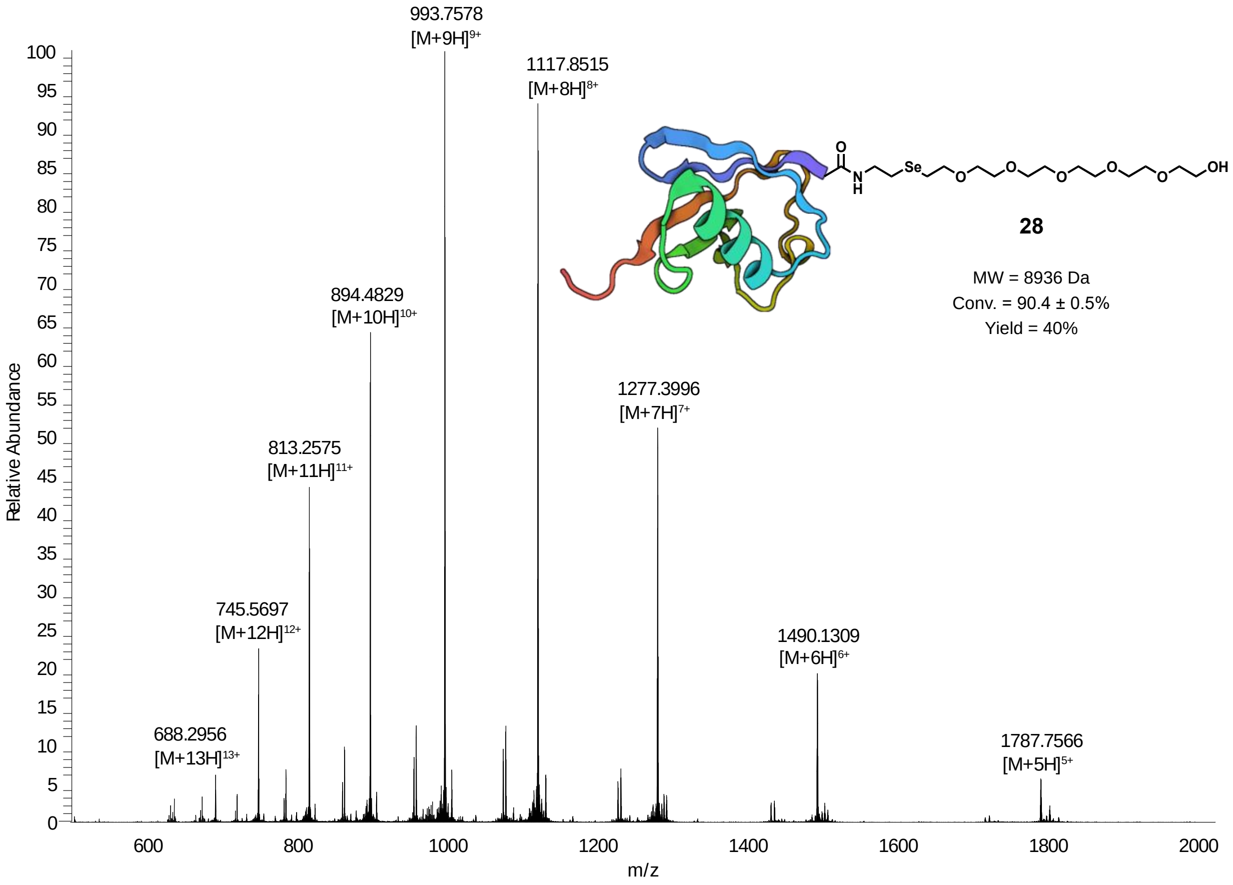

Supplementary Figure 123. HRMS spectrum of the crude reaction mixture of the PDC functionalization of Ub diselenide **27** with [Se-PEG$_6$]$_2$ (**16**) after 5 min irradiation at 450 nm. Ion peaks for the Ub-PEG$_6$ selenoether **28** product are labelled. Reaction conversions were calculated through averaging integrations of HRMS-derived extracted ion chromatograms of the [M+9H]$^{9+}$, [M+8H]$^{8+}$ and [M+7H]$^{7+}$ charge states and errors are reported as the standard deviation of the integration of these three ion peaks for a single experiment.

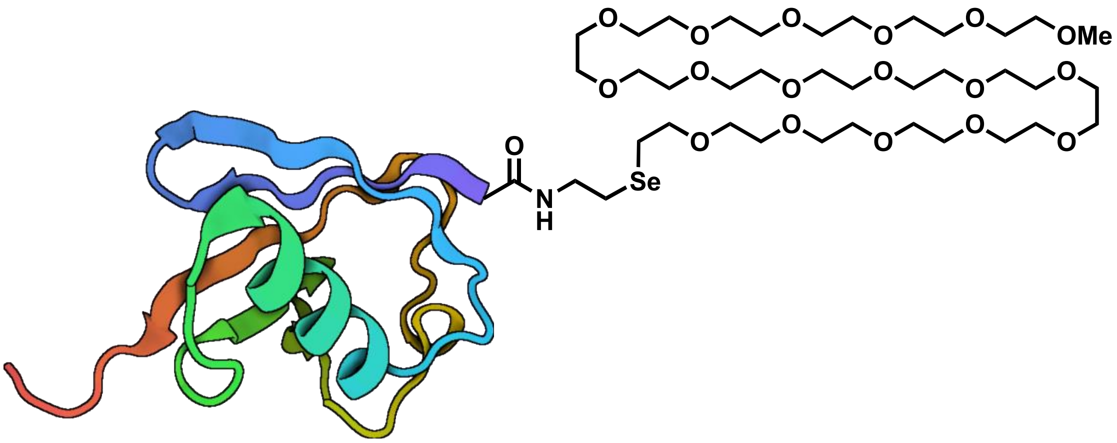

Ubiquitin-mPEG$_{17}$ Selenoether 29

This PDC reaction was performed on 1.2 mg scale of Ubiquitin diselenide **27** using conditions described for the PEG$_6$ example above. To obtain an isolated yield, the same experiment was repeated and the crude reaction mixture was diluted with 6 M Gnd.HCl, 0.1 M NaP$_i$, pH 7.0 buffer (1 mL) and subjected to RP-HPLC purification to afford the purified Ubiquitin-mPEG$_{17}$ selenoether **29** in 71% yield (0.93 mg).

See next pages for analytical data.

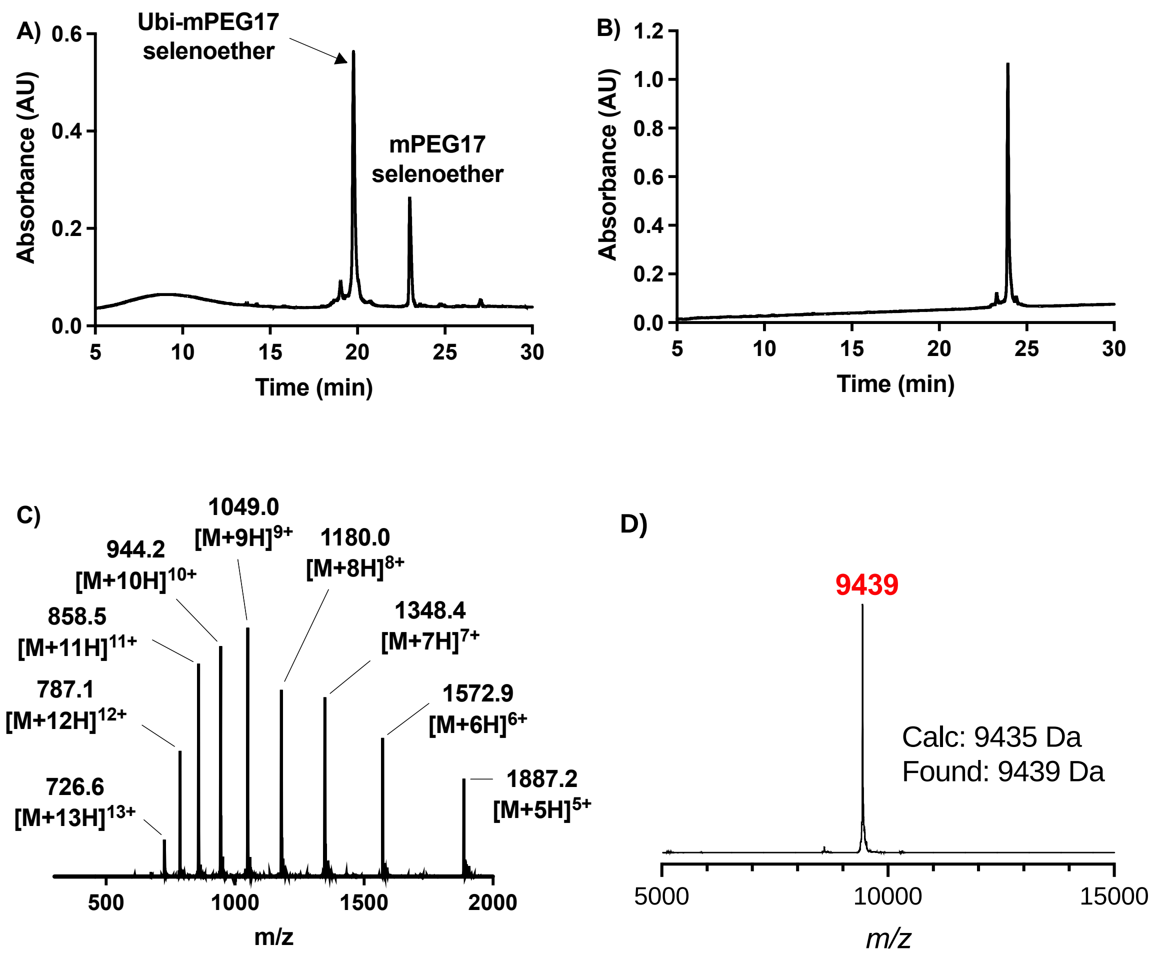

Supplementary Figure 124. A) Crude analytical HPLC trace of PDC reaction between Ubiquitin diselenide **27** and [Se-mPEG$_{17}$]$_2$ diselenide after 5 min; R$_t$ 19.8 min (1-50% B over 30 min, 0.1 vol% TFA, λ = 214 nm); **B)** Analytical HPLC trace of RP-HPLC purified Ubiquitn-mPEG$_{17}$ selenoether **29**; R$_t$ 23.9 min (1-50% B over 30 min, 0.1 vol% TFA, XBridge Peptide BEH300 C18 5 µm, 4.6 × 250 mm column, 60 °C, λ = 214 nm); **C)** ESI-MS of Ubiquitin-mPEG$_{17}$ selenoether **29**; Calculated Mass: $[M+5H]^{5+}$: 1888.0; $[M+6H]^{6+}$: 1573.5; $[M+7H]^{7+}$: 1348.9; $[M+8H]^{8+}$: 1180.4; $[M+9H]^{9+}$: 1049.3; $[M+10H]^{10+}$: 944.5; $[M+11H]^{11+}$: 858.7; $[M+12H]^{12+}$: 787.3; $[M+13H]^{13+}$: 726.8; Mass Found (ESI+) 1887.2 $[M+5H]^{5+}$; 1572.9 $[M+6H]^{6+}$; 1348.4 $[M+7H]^{7+}$; 1180.0 $[M+8H]^{8+}$; 1049.0 $[M+9H]^{9+}$; 944.2 $[M+10H]^{10+}$; 858.5 $[M+11H]^{11+}$; 787.1 $[M+12H]^{12+}$; 726.6 $[M+13H]^{13+}$. ESI-MS data was collected over the entire gradient and wash cycle of the UPLC-MS. **D)** MALDI-TOF MS spectrum of the crude reaction mixture of the PDC functionalization of Ub diselenide **27** with [Se-mPEG$_{17}$]$_2$ after 5 min irradiation at 450 nm.

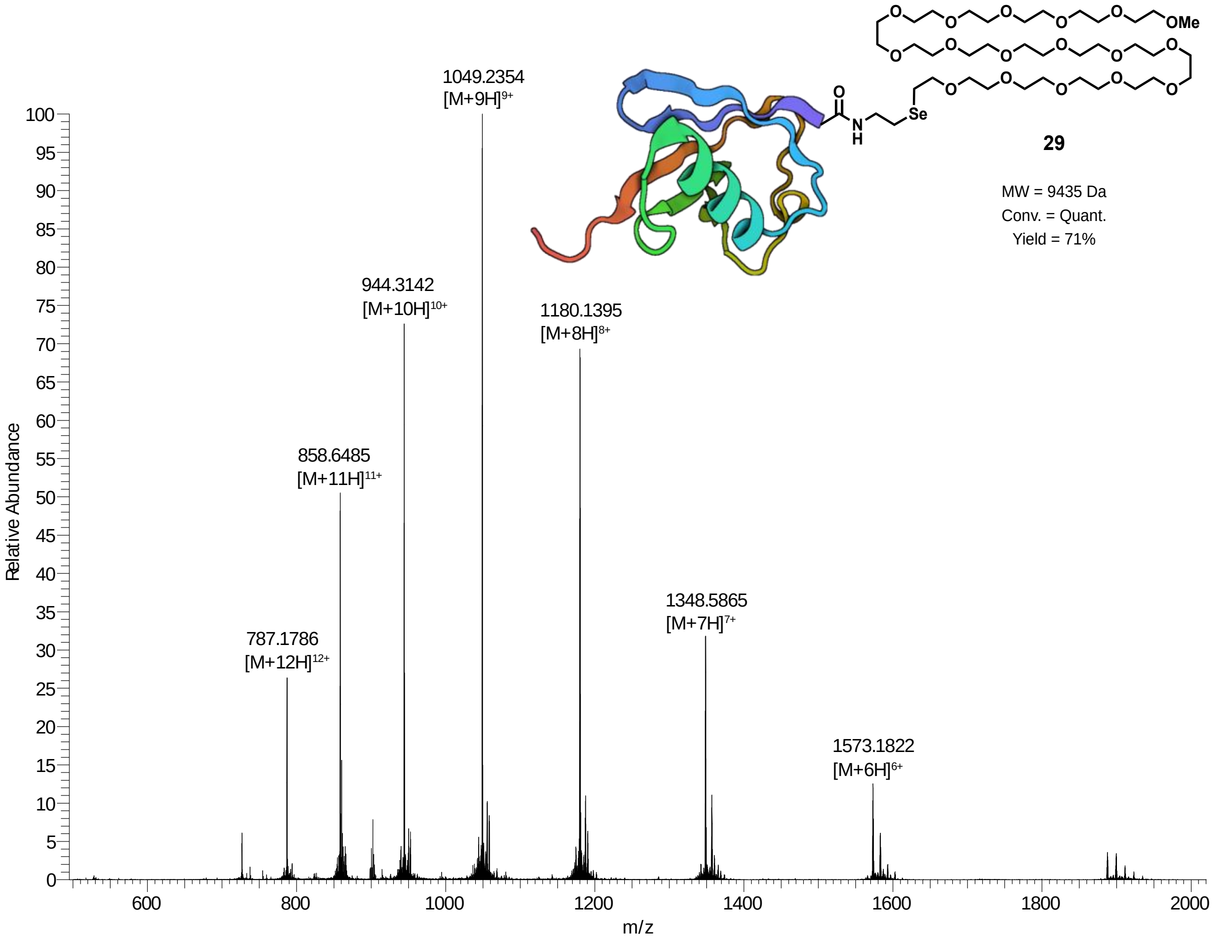

Supplementary Figure 125. HRMS spectrum of the crude reaction mixture of the PDC functionalization of Ub diselenide **27** with [Se-mPEG$_{17}$]$_2$ after 5 min irradiation at 450 nm. Ion peaks for the Ub-mPEG$_{17}$ selenoether **29** product are labelled. Reaction conversions were calculated through averaging integrations of HRMS-derived extracted ion chromatograms of the [M+9H]$^{9+}$, [M+8H]$^{8+}$ and [M+7H]$^{7+}$ charge states and errors are reported as the standard deviation of the integration of these three ion peaks for a single experiment.

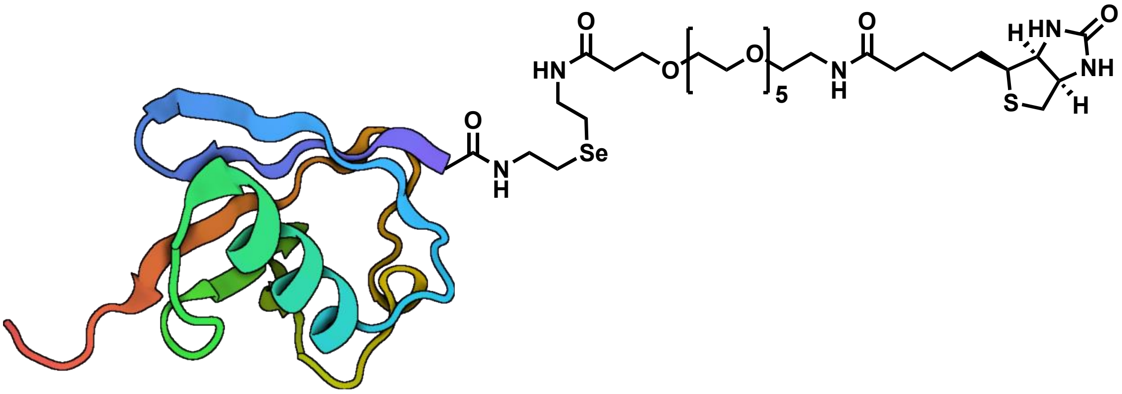

Ubiquitin-PEG$_5$-Biotin Selenoether 30

This PDC reaction was performed on 1.5 mg scale of Ubiquitin diselenide using conditions described for the PEG$_6$ example above. To obtain an isolated yield, the same experiment was repeated and the crude reaction mixture was subjected to RP-HPLC purification to afford the purified Ubiquitin-PEG$_5$-Biotin selenoether **30** in 50% yield (0.8 mg).

See next pages for analytical data.

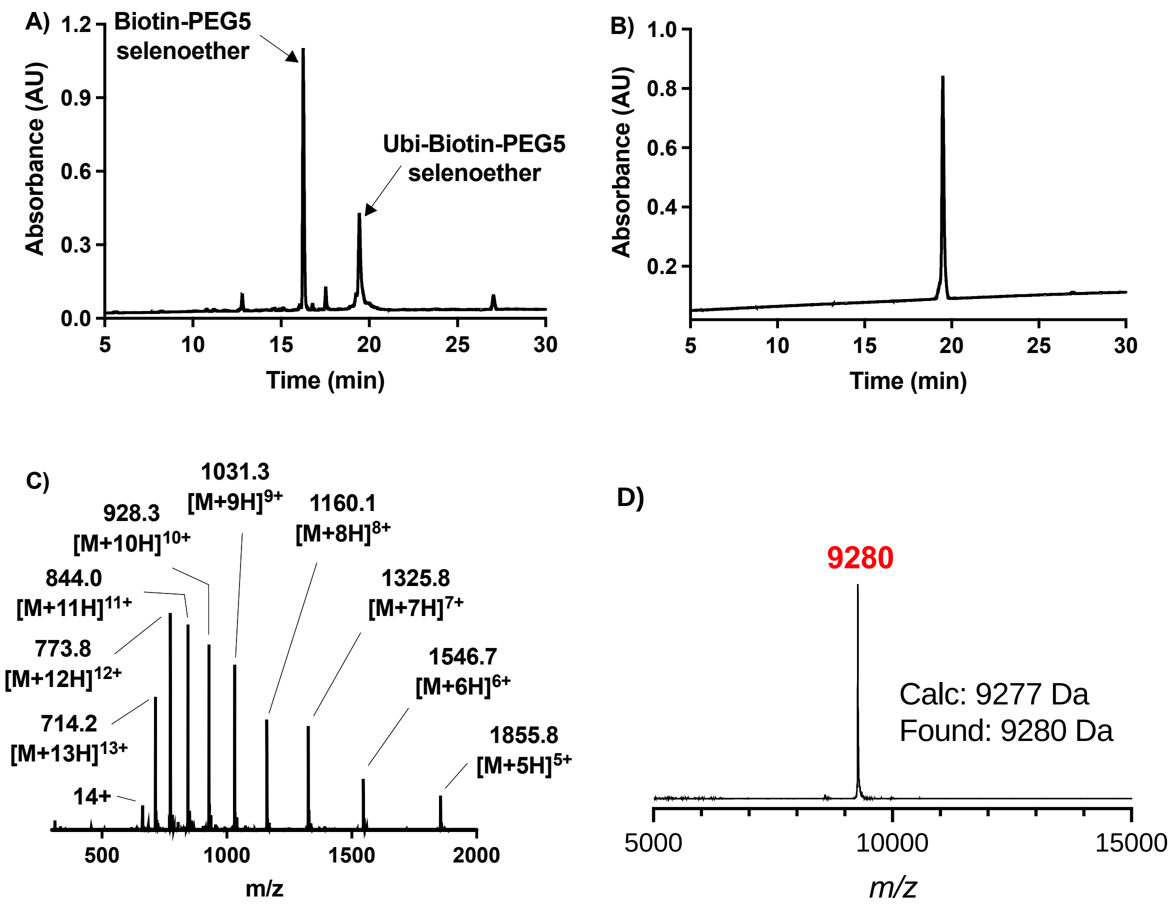

Supplementary Figure 126. A) Crude analytical HPLC trace of PDC reaction between Ubiquitin diselenide **27** and Biotin-PEG$_5$ diselenide after 10 min; R$_t$ 19.5 min (1-50% B over 30 min, 0.1 vol% TFA, λ = 214 nm); **B)** Analytical HPLC trace of RP-HPLC purified Ubiquitn-PEG$_5$-Biotin selenoether **30**; R$_t$ 19.5 min (1-50% B over 30 min, 0.1 vol% TFA, λ = 214 nm); **C)** ESI-MS of Ubiquitn-PEG$_5$-Biotin selenoether **30**; Calculated Mass: $[M+5H]^{5+}$: 1856.4; $[M+6H]^{6+}$: 1547.2; $[M+7H]^{7+}$: 1326.3; $[M+8H]^{8+}$: 1160.6; $[M+9H]^{9+}$: 1031.8; $[M+10H]^{10+}$: 928.7; $[M+11H]^{11+}$: 844.4; $[M+12H]^{12+}$: 774.1; $[M+13H]^{13+}$: 714.6; $[M+14H]^{14+}$: 663.6; Mass Found (ESI+) 1855.8 $[M+5H]^{5+}$; 1546.7 $[M+6H]^{6+}$; 1325.8 $[M+7H]^{7+}$; 1160.1 $[M+8H]^{8+}$; 1031.3 $[M+9H]^{9+}$; 928.3 $[M+10H]^{10+}$; 844.0 $[M+11H]^{11+}$; 773.8 $[M+12H]^{12+}$; 714.2 $[M+13H]^{13+}$; 663.3 $[M+14H]^{14+}$. ESI-MS data was collected over the entire gradient and wash cycle of the UPLC-MS. **D)** MALDI-TOF MS spectrum of the crude reaction mixture of the PDC functionalization of Ub diselenide **27** with [Se-PEG$_5$-Biotin]$_2$ after 10 min irradiation at 450 nm.

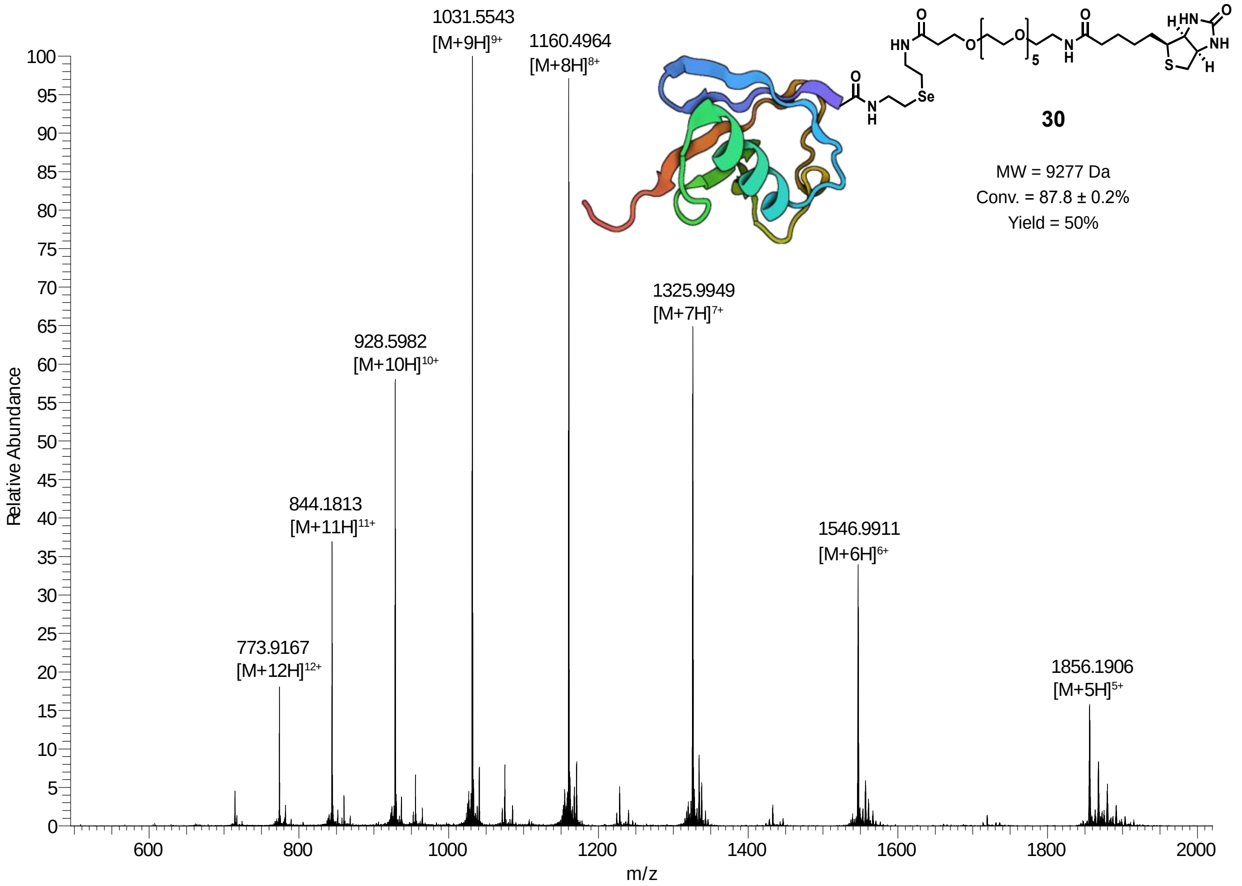

Supplementary Figure 127. HRMS spectrum of the crude reaction mixture of the PDC functionalization of Ub diselenide **27** with [Se-PEG$_5$-Biotin]$_2$ after 10 min irradiation at 450 nm. Ion peaks for the Ub-PEG$_5$-Biotin selenoether **30** product are labelled. Reaction conversions were calculated through averaging integrations of HRMS-derived extracted ion chromatograms of the $[M+9H]^{9+}$, $[M+8H]^{8+}$ and $[M+7H]^{7+}$ charge states and errors are reported as the standard deviation of the integration of these three ion peaks for a single experiment.

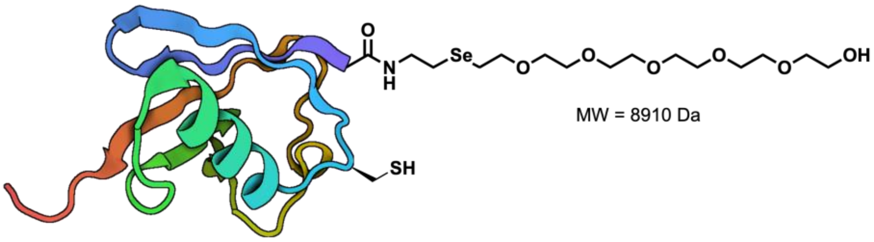

Ubiquitin (K48C)-PEG$_6$ selenoether

This PDC reaction was performed on 0.5 mg scale of Ubiquitin (K48C) with 8 eq. of [Se-PEG$_6$]$_2$ diselenide **16** and 18 eq. of PTA at room temperature to avoid deleterious desulfurization.

Three 5 μL aliquots were taken at 0, 1, 2, 5 and 10 min time points and individually diluted 4-fold in 6 M Gnd.HCl, 0.1 M NaP$_i$, pH 7.0 buffer (15 μL). One aliquot was desalted using a C18 ZipTip (Merck, Darmstadt, Germany) and analyzed by MALDI-TOF MS (*see General Procedures*). The other diluted aliquot was directly analyzed by LC-HRMS (*see General Procedures*). Reaction conversion was calculated through averaging integrations of HRMS-derived extracted ion chromatograms of the $[M+9H]^{9+}$ and $[M+8H]^{8+}$ charge states. Conversion errors are reported as the standard deviation of these two ion peak integrations for a single experiment.

See next pages for analytical data.

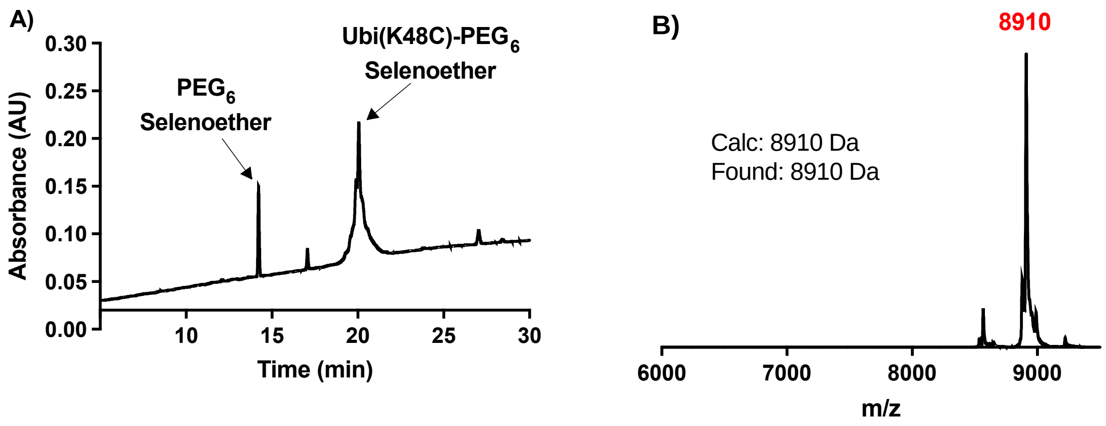

Supplementary Figure 128. A) Crude analytical HPLC trace of PDC reaction between Ub(K48C) and [Se-PEG$_6$]$_2$ diselenide **16** after 10 min; R$_t$ 20.1 min (1-50% B over 30 min, 0.1% v/v TFA, λ = 214 nm); **B)** MALDI-TOF MS spectrum of the crude reaction mixture of the PDC functionalization of Ub(K48C) with [Se-PEG$_6$]$_2$ diselenide **16** after 10 min irradiation at 450 nm.

Synthesis of [H_2N-Sec-$(PEG_4)_2$-Arg_8-$CONH_2$]$_2$ Diselenide

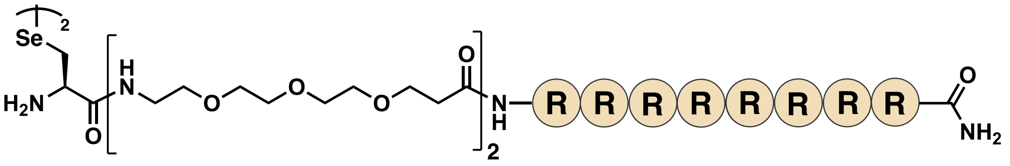

[H_2N-Sec-$(PEG_4)_2$-Arg_8-$CONH_2$]$_2$ Diselenide

Fmoc-Arg(Pbf)-OH was loaded onto Rink amide resin and the peptide was extended (50 µmol) using standard Fmoc-SPPS techniques as outlined in the general methods section. Fmoc-PEG_3-OH (x 2) and [Boc-Sec-OH]$_2$ were coupled manually using DIC/Oxyma coupling conditions (Fmoc-PEG_4-OH (2.5 eq.) or [Boc-Sec-OH]$_2$ (1.25 eq.), DIC (2.5 eq.) and Oxyma (2.5 eq.) in DMF (0.1 M) for 16 h at room temperature). Removal of the acid labile protecting groups, with concomitant cleavage from resin, was achieved *via* treatment with a solution of TFA/*i*Pr_3SiH /H_2O (90:5:5 v/v/v, 7 mL) for 2 h at room temperature. After filtering off the resin, the deprotection solution was concentrated under nitrogen flow and the crude peptide was precipitated from ice-cold Et_2O. The crude peptide (33 µmol) was purified by RP-HPLC (C18 X-bridge column, 5 µm, 30 × 150 mm, 0 to 50% B over 70 min, 0.1 vol% TFA) to afford the target peptide diselenide as a white fluffy solid after lyophilization (25 mg, 42% yield).

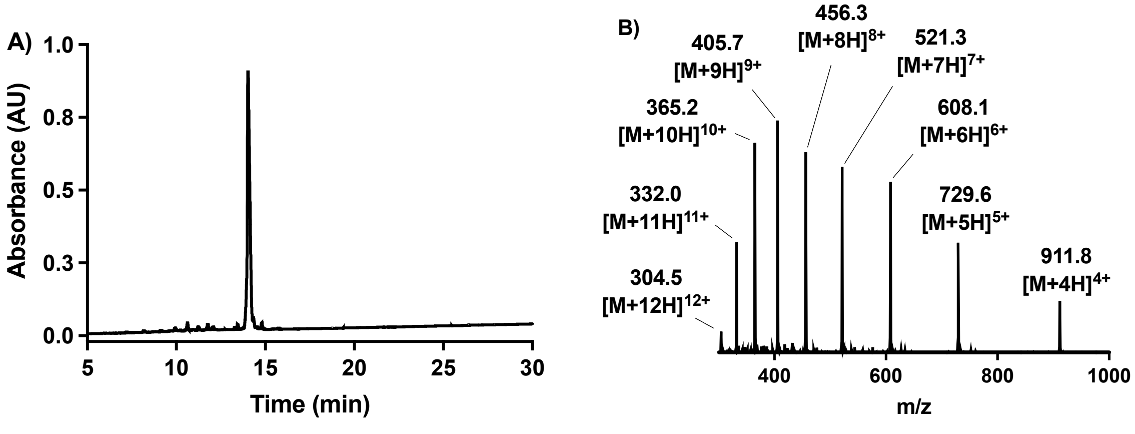

Supplementary Figure 129. A) Analytical HPLC trace of purified [H_2N-Sec-$(PEG_4)_2$-Arg_8-$CONH_2$]$_2$ diselenide, R_t 14.0 min (1 to 30% B over 30 min, 0.1 vol% TFA, λ = 214 nm); **B)** ESI-MS of pure [H_2N-Sec-$(PEG_4)_2$-Arg_8-$CONH_2$]$_2$ diselenide; Calculated Mass: $[M+4H]^{4+}$: 912.0; $[M+5H]^{5+}$: 729.8; $[M+6H]^{6+}$: 608.3; $[M+7H]^{7+}$: 521.6; $[M+8H]^{8+}$: 456.5; $[M+9H]^{9+}$: 405.9; $[M+10H]^{10+}$: 365.4; $[M+11H]^{11+}$: 332.3; $[M+12H]^{12+}$: 304.7. Mass Found (ESI+) 911.8 $[M+4H]^{4+}$; 729.6 $[M+5H]^{5+}$; 608.1 $[M+6H]^{6+}$; 521.3 $[M+7H]^{7+}$; 456.3 $[M+8H]^{8+}$; 405.7 $[M+9H]^{9+}$; 365.2 $[M+10H]^{10+}$; 332.0 $[M+11H]^{11+}$; 304.5 $[M+12H]^{12+}$. ESI-MS data was collected over the entire gradient and wash cycle of the UPLC-MS.

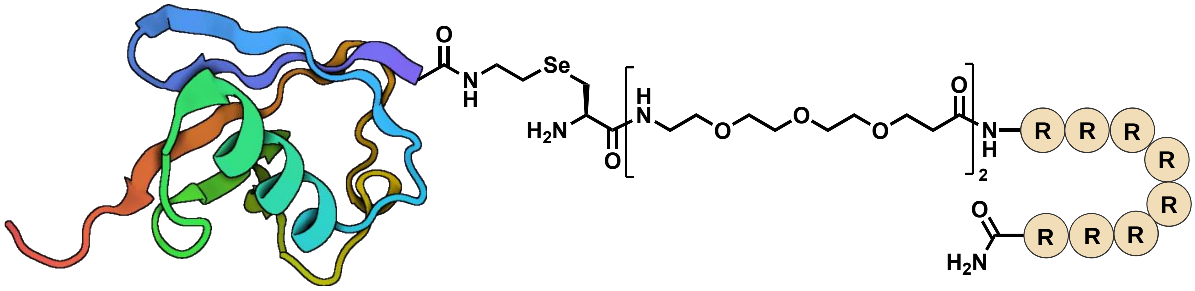

Ubiquitin-$(PEG_4)_2$-Arg_8 Selenoether 31

This PDC reaction was performed on 1.2 mg scale of Ubiquitin diselenide **27** using conditions described for the PEG_6 example above. The same experiment was repeated and the crude reaction mixture was subjected to RP-HPLC purification to afford the purified Ubiquitin-$(PEG_4)_2$-Arg_8 selenoether **31** in 65% yield (1.0 mg).

See next pages for analytical data.

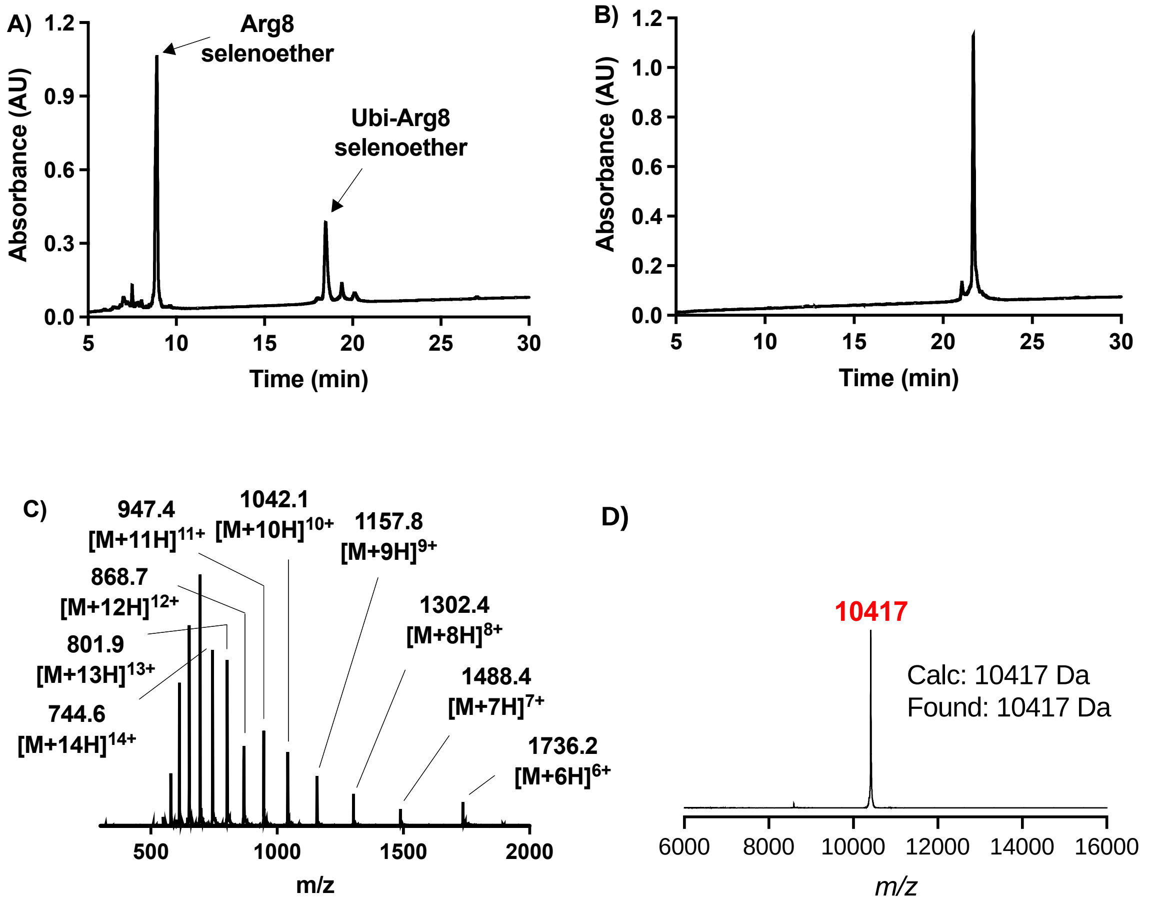

Supplementary Figure 130. A) Crude analytical HPLC trace of PDC reaction between Ubiquitin diselenide **27** and [Sec-(PEG$_4$)$_2$-Arg$_8$]$_2$ diselenide after 5 min; R$_t$ 18.5 min (1-50% B over 30 min, 0.1 vol% TFA, λ = 214 nm); **B)** Analytical HPLC trace of RP-HPLC purified Ubiquitin-(PEG$_4$)$_2$-Arg$_8$ selenoether **31**; R$_t$ 21.7 min (1-50% B over 30 min, 0.1 vol% TFA, XBridge Peptide BEH300 C18 5 µm, 4.6 × 250 mm column, 60 °C, λ = 214 nm); **C)** ESI-MS of RP-HPLC purified Ubiquitin-(PEG$_4$)$_2$-Arg$_8$ selenoether **31**; Calculated Mass: [M+6H]$^{6+}$: 1736.7; [M+7H]$^{7+}$: 1488.7; [M+8H]$^{8+}$: 1302.8; [M+9H]$^{9+}$: 1158.1; [M+10H]$^{10+}$: 1042.4; [M+11H]$^{11+}$: 947.7; [M+12H]$^{12+}$: 868.8; [M+13H]$^{13+}$: 802.1; [M+14H]$^{14+}$: 744.9; [M+15H]$^{15+}$: 695.3; [M+16H]$^{16+}$: 651.9; [M+17H]$^{17+}$: 613.6; [M+18H]$^{18+}$: 579.6; Mass Found (ESI+) 1736.2 [M+6H]$^{6+}$; 1488.4 [M+7H]$^{7+}$; 1302.4 [M+8H]$^{8+}$; 1157.8 [M+9H]$^{9+}$; 1042.1 [M+10H]$^{10+}$; 947.4 [M+11H]$^{11+}$; 868.7 [M+12H]$^{12+}$; 801.9 [M+13H]$^{13+}$; 744.6 [M+14H]$^{14+}$; 695.0 [M+15H]$^{15+}$; 651.6 [M+16H]$^{16+}$; 613.3 [M+17H]$^{17+}$; 579.3 [M+18H]$^{18+}$. ESI-MS data was collected over the entire gradient and wash cycle of the UPLC-MS. **D)** MALDI-TOF MS spectrum of the crude reaction mixture of the PDC functionalization of Ub diselenide **27** with [Sec-(PEG$_3$)$_2$-Arg$_8$]$_2$ after 5 min irradiation at 450 nm.

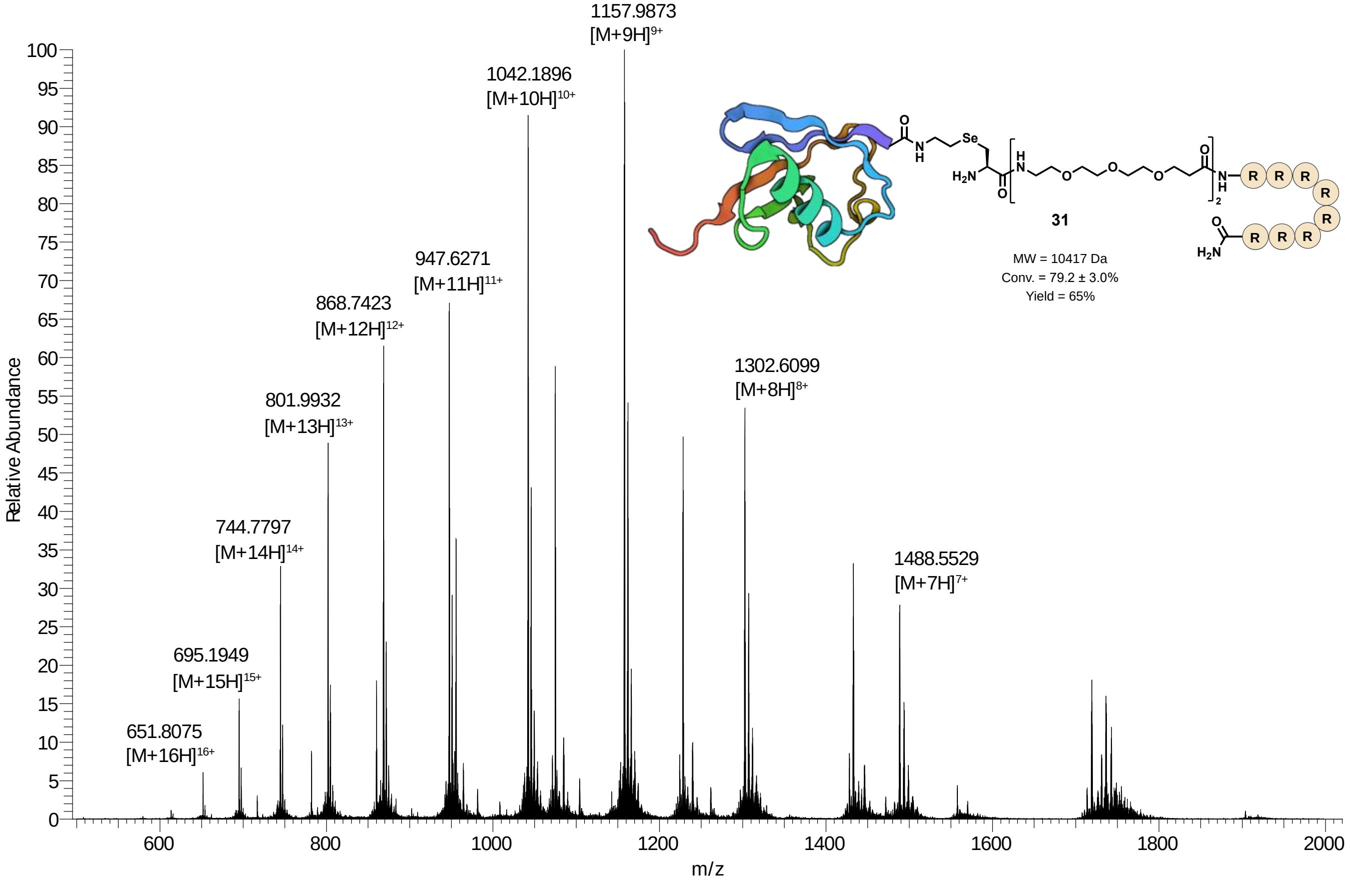

Supplementary Figure 131. HRMS spectrum of the crude reaction mixture of the PDC functionalization of Ub diselenide **27** with [Sec-$(PEG_4)_2$-Arg_8]$_2$ diselenide after 5 min irradiation at 450 nm. Ion peaks for the Ub-$(PEG_4)_2$-Arg_8 selenoether **31** product are labelled. Reaction conversions were calculated through averaging integrations of HRMS-derived extracted ion chromatograms of the $[M+9H]^{9+}$, $[M+8H]^{8+}$ and $[M+7H]^{7+}$ charge states charge states and errors are reported as the standard deviation of the integration of these three ion peaks for a single experiment.

Synthesis of [H2AX$_{120-128}$ (K120U)]$_2$ Diselenide

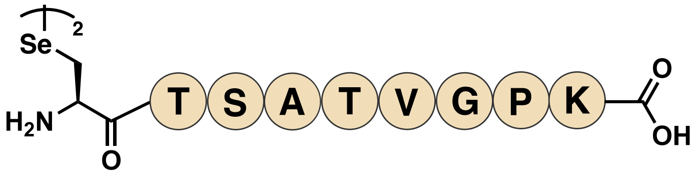

[H2AX$_{120-128}$ (K120U)]$_2$ Diselenide

Fmoc-Lys(Boc)-OH was loaded onto 2-CTC resin and the peptide was extended (50 µmol) using standard Fmoc-SPPS techniques as outlined in the general methods section. (Boc-Sec-OH)$_2$ was coupled manually using DIC/Oxyma coupling conditions [(Boc-Sec-OH)$_2$ (1.25 eq.), DIC (2.5 eq.) and Oxyma (2.5 eq.) in DMF (0.1 M) for 16 h at room temperature]. Removal of the acid labile protecting groups, with concomitant cleavage from resin, was achieved *via* treatment with a solution of TFA/*i*Pr$_3$SiH/H$_2$O (90:5:5 v/v/v, 7 mL) for 2 h at room temperature. After filtering off the resin, the deprotection solution was concentrated under nitrogen flow and the crude peptide was precipitated from ice-cold Et$_2$O. The crude peptide (50 µmol) was purified by RP-HPLC (C18 X-bridge column, 5 µm, 19 × 150 mm, 0 to 30% B over 50 min, 0.1 vol% TFA) to afford the target peptide diselenide as a white fluffy solid after lyophilization (12.3 mg, 27% yield).

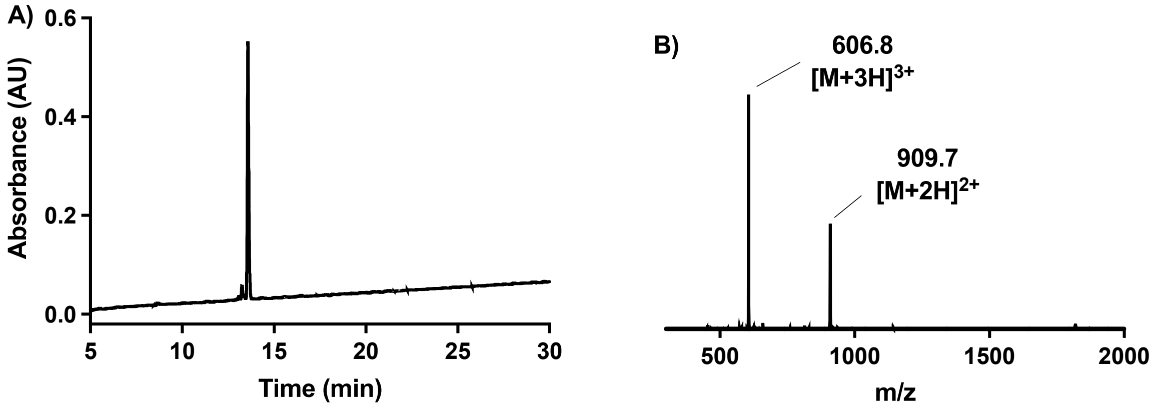

Supplementary Figure 132. A) Analytical HPLC trace of purified peptide [H2AX$_{120-128}$ (K120U)]$_2$ diselenide, R$_t$ 13.6 min (1 to 40% B over 30 min, 0.1 vol% TFA, λ = 214 nm); **B)** ESI-MS of pure [H2AX$_{120-128}$ (K120U)]$_2$ diselenide; Calculated Mass: $[M+2H]^{2+}$: 909.9; $[M+3H]^{3+}$: 606.9. Mass Found (ESI+) 909.7 $[M+2H]^{2+}$; 606.8 $[M+3H]^{3+}$. ESI-MS data was collected over the entire gradient and wash cycle of the UPLC-MS.

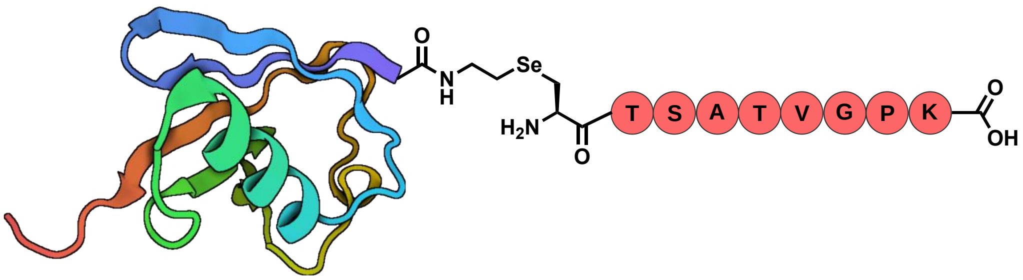

Ubiquitin-H2AX Selenoether 32

This PDC reaction was performed on 1.4 mg scale of Ubiquitin diselenide **27** using conditions described for the PEG_6 example above. The same experiment was repeated and the crude reaction mixture was subjected to RP-HPLC purification to afford the purified Ubiquitin-H2AX selenoether **32** in 58% yield (0.9 mg).

See next pages for analytical data.

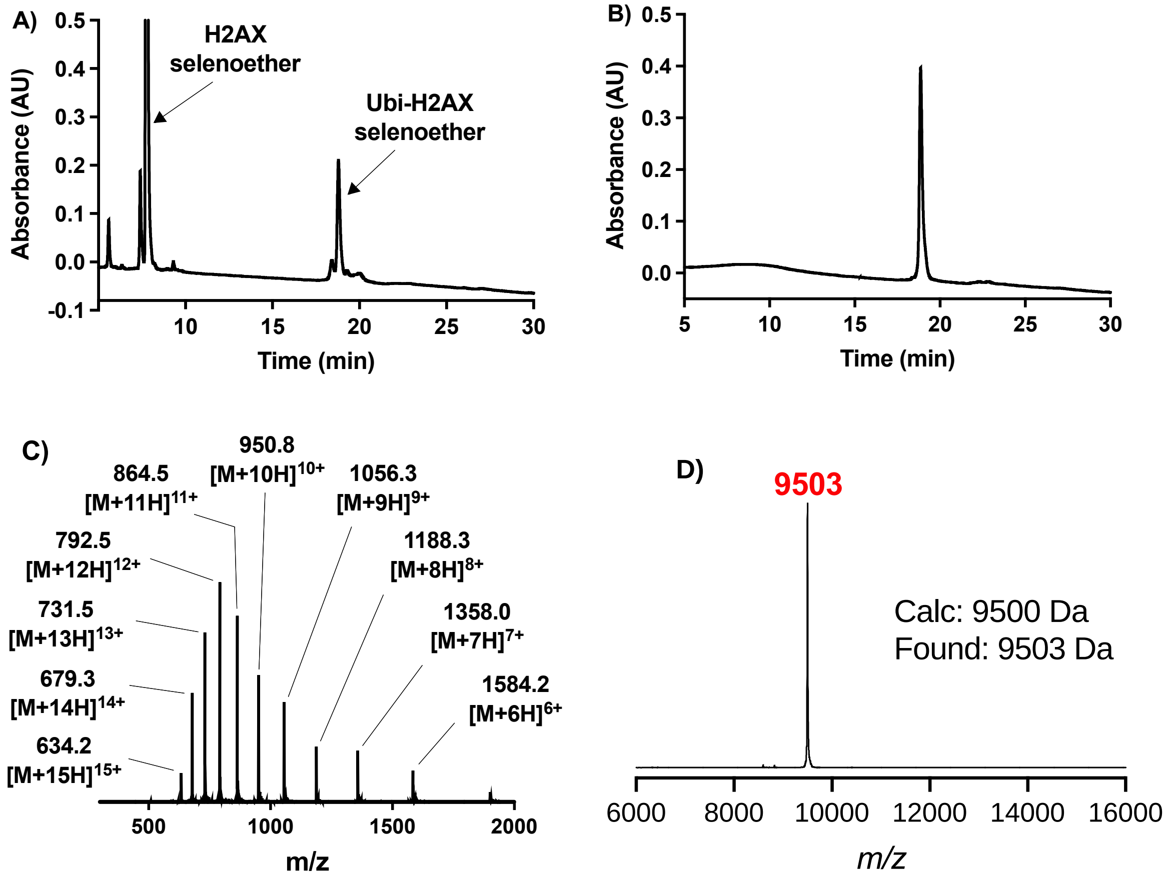

Supplementary Figure 133. A) Crude analytical HPLC trace of PDC reaction between Ubiquitin diselenide **27** and H2AX diselenide after 5 min; R_t 18.8 min (1-50% B over 30 min, 0.1% v/v TFA, λ = 214 nm); **B)** Analytical HPLC trace of RP-HPLC purified Ubiquitin-H2AX selenoether **32**; R_t 18.9 min (1-50% B over 30 min, 0.1% v/v TFA, λ = 214 nm); **C)** ESI-MS of RP-HPLC purified Ubiquitin-H2AX selenoether **32**; Calculated Mass: [M+6H]$^{6+}$: 1584.3; [M+7H]$^{7+}$: 1358.1; [M+8H]$^{8+}$: 1188.5; [M+9H]$^{9+}$: 1056.6; [M+10H]$^{10+}$: 951.0; [M+11H]$^{11+}$: 864.6; [M+12H]$^{12+}$: 792.7; [M+13H]$^{13+}$: 731.8; [M+14H]$^{14+}$: 679.6; [M+15H]$^{15+}$: 634.3; Mass Found (ESI+) 1584.2 [M+6H]$^{6+}$; 1358.0 [M+7H]$^{7+}$; 1188.3 [M+8H]$^{8+}$; 1056.3 [M+9H]$^{9+}$; 950.8 [M+10H]$^{10+}$; 864.5 [M+11H]$^{11+}$; 792.5 [M+12H]$^{12+}$; 731.5 [M+13H]$^{13+}$; 679.3 [M+14H]$^{14+}$; 634.2 [M+15H]$^{15+}$. ESI-MS data was collected over the entire gradient and wash cycle of the UPLC-MS. **D)** MALDI-TOF MS spectrum of the crude reaction mixture of the PDC functionalization of Ub diselenide **27** with H2AX diselenide after 5 min irradiation at 450 nm.

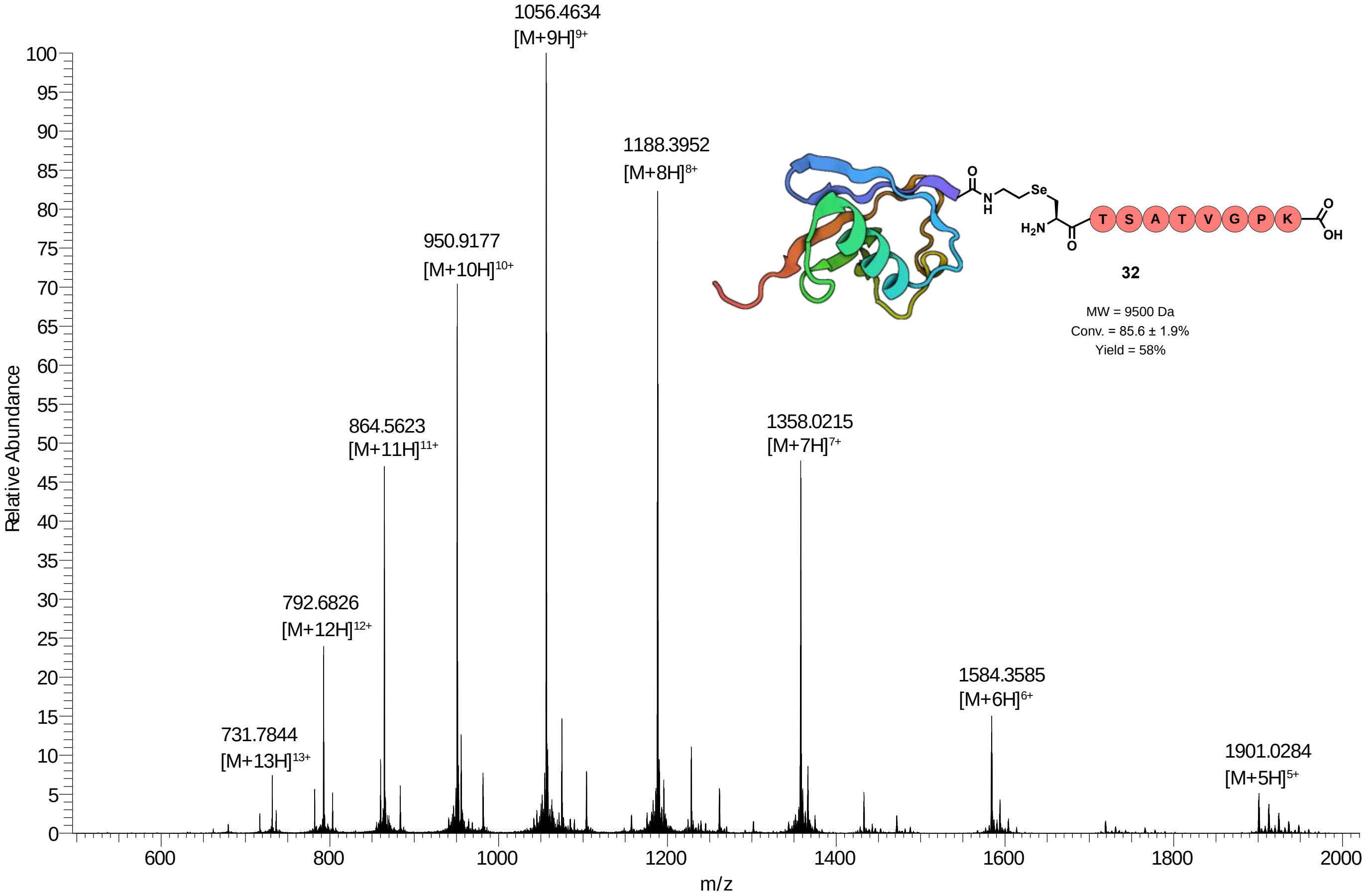

Supplementary Figure 134. HRMS spectrum of the crude reaction mixture of the PDC functionalization of Ub diselenide **27** with $[Sec\text{-}H2AX]_2$ diselenide after 5 min irradiation at 450 nm. Ion peaks for the Ub-H2AX selenoether **32** product are labelled. Reaction conversions were calculated through averaging integrations of HRMS-derived extracted ion chromatograms of the $[M+9H]^{9+}$, $[M+8H]^{8+}$ and $[M+7H]^{7+}$ charge states charge states and errors are reported as the standard deviation of the integration of these three ion peaks for a single experiment.

Synthesis of Small Molecule Diselenides

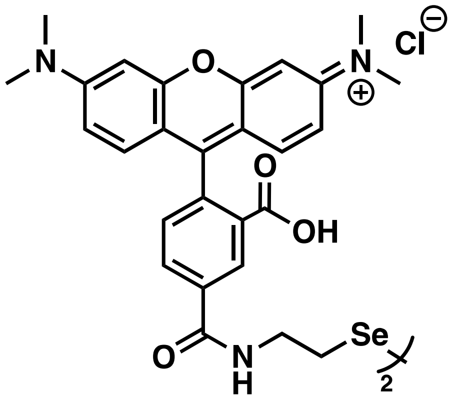

5,5'-(((diselanediylbis(ethane-2,1-diyl))bis(azanediyl))bis(carbonyl))bis(2-(3,6-bis(dimethylamino)xanthylium-9-yl)benzoate) (15)

To a solution of 5-TAMRA (25 mg, 0.058 mmol) in DMF (0.4 mL) at 0 ºC was added triethylamine (16 μL, 0.12 mmol) and PyBOP (30 mg, 0.058 mmol). The mixture was stirred at room temperature for 10 min before selenocystamine dihydrochloride (11 mg, 0.070 mmol) was added. The mixture was stirred at room temperature for 16 h before concentrating *in vacuo*. The crude residue was purified by reverse-phase liquid chromatography (Waters X-bridge C18 19 x 150 mm column, 0–60% B over 50 min, 0.1 vol% TFA) to give the *title compound* **15** (5 mg, 16%) as a purple solid.

^{1}H NMR: (500 MHz; CD_3OD) δ 9.16 (2H, t, *J* = 5.4 Hz, 2 × N<u>H</u>), 8.80 (2H, d, *J* = 1.4 Hz, 2 × Ar-<u>H</u>), 8.27 (2H, dd, *J* = 7.9, 1.5 Hz, 2 × Ar-<u>H</u>), 7.49 (2H, d, *J* = 7.9 Hz, 2 × Ar-<u>H</u>), 7.1–7.05 (4H, m, 4 × Ar-<u>H</u>), 6.97–6.95 (4H, m, 4 × Ar-<u>H</u>), 6.97 (4H, s, 4 × Ar-<u>H</u>), 3.92–3.88 (4H, m, 2 × NHC<u>H</u>$_2$), 3.34 (4H, t, *J* = 6.9 Hz, 2 × SeC<u>H</u>$_2$), 3.29 (24H, s, 8 × C<u>H</u>$_3$). **^{13}C NMR:** (126 MHz; CD_3OD) δ: 168.2 (2 × C), 160.7 (2 × C), 159.0 (6 × C), 158.9 (4 × C), 138.2 (2 × C), 137.6 (4 × C), 132.4 (2 × CH), 132.0 (4 × CH), 131.9 (2 × CH), 131.4 (2 × CH), 115.5 (4 × CH), 114.7 (4 × C), 97.4 (4 × CH), 42.3 (2 × CH_2), 40.9 (8 × CH_3), 29.2 (2 × CH_2); **HRMS (ESI+):** calculated for [$C_{54}H_{53}N_6O_8Se_2$] 1073.22646, found 1073.22598.

Synthesis of (Se-PEG$_6$)$_2$ (16)

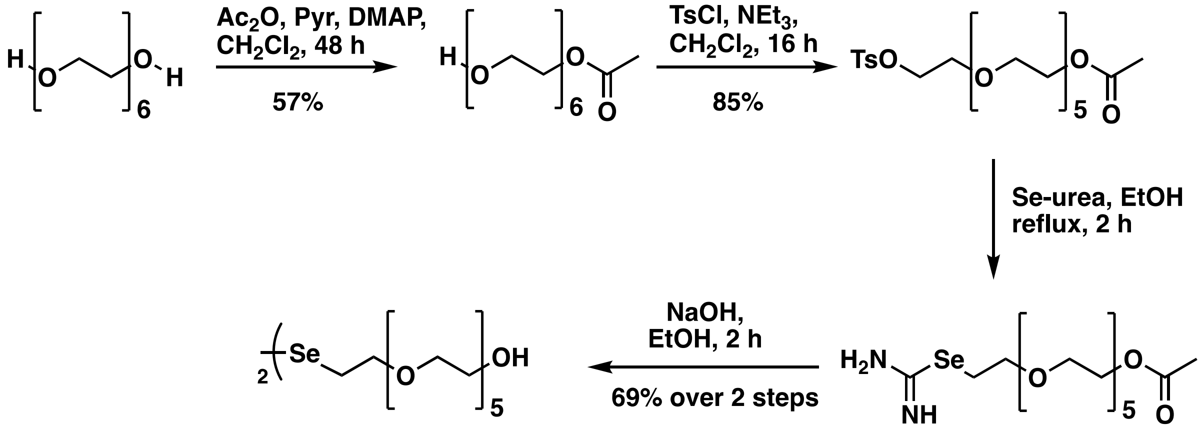

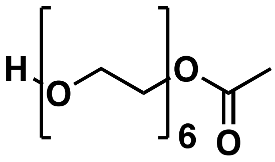

AcO-PEG$_6$-OH

To a solution of hexaethylene glycol (3.0 g, 10 mmol) in CH_2Cl_2 (90 mL) was added pyridine (3.6 mL, 45 mmol) and Ac_2O (500 µL, 5.3 mmol) followed by DMAP (60 mg, 0.41 mmol). The reaction mixture was stirred at room temperature for 48 h before concentrating *in vacuo*. The residue was purified by flash chromatography on silica gel (2–5% MeOH in EtOAc) to give the *title compound* (980 mg, 3.00 mmol, 57%) as a colorless oil.

^{1}H NMR: (400 MHz; $CDCl_3$) δ 4.19–4.14 (2H, m, C<u>H</u>$_2$O$_2$CCH$_3$), 3.70–3.62 (4H, m, C<u>H</u>$_2$CH$_2$OH and C<u>H</u>$_2$CH$_2$O$_2$CCH$_3$), 3.62–3.59 (16H, m, -C<u>H</u>$_2$-), 3.57–3.53 (2H, m, 2 × C<u>H</u>$_2$OH), 2.02 (3H, s, O$_2$CC<u>H</u>$_3$). **^{13}C NMR:** (101 MHz, $CDCl_3$) δ 171.0 (C), 72.6 (CH_2), 70.62 (2 × CH_2), 70.57 (5 × CH_2), 70.3 (CH_2), 69.1 (CH_2), 63.6 (CH_2), 61.7 (CH_2), 21.0 (CH_3). **HRMS (ESI+):** calculated for [$C_{14}H_{29}O_8$] 325.18569, found 325.18572.

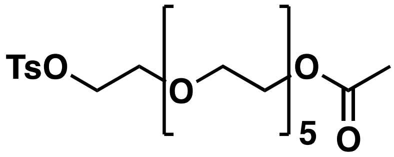

AcO-PEG$_6$-OTs

To a solution of AcO-PEG$_6$-OH (960 mg, 2.96 mmol) (*see above*) in CH_2Cl_2 (30 mL) at 0 ºC was added Et_3N (1.8 mL, 13 mmol) and *p*-TsCl (1.00 g, 5.24 mmol). The reaction mixture was stirred at room temperature for 16 h before concentrating *in vacuo*. The residue was purified by flash chromatography on silica gel (80–100% EtOAc in hexanes) to give the *title compound* (1.20 g, 2.51 mmol, 85%) as a colorless oil.

^{1}H NMR: (400 MHz; $CDCl_3$) δ 7.79–7.76 (2H, m, 2 × Ar-H), 7.34–7.31 (2H, m, 2 × Ar-H), 4.21–4.19 (2H, m, $C\underline{H}_2CH_2O_2CC\underline{H}_3$), 4.16–4.12 (2H, m, $C\underline{H}_2OTs$), 3.69–3.65 (4H, m, -$C\underline{H}_2$- and $C\underline{H}_2CH_2O_2CCH_3$), 3.64–3.59 (12H, m, 6 × -$C\underline{H}_2$-), 3.57–3.55 (4H, m, 2 × -$C\underline{H}_2$-), 2.43 (3H, s, Ar-$C\underline{H}_3$), 2.06 (3H, s, $O_2CC\underline{H}_3$). **^{13}C NMR:** (101 MHz, $CDCl_3$) δ 170.9 (C), 144.7 (C), 133.0 (C), 129.7 (2 × CH), 127.9 (2 × CH), 70.7 (CH_2), 70.53 (2 × CH_2), 70.47 (4 × CH_2), 70.4 (CH_2), 69.1 (CH_2), 69.0 (CH_2), 68.6 (CH_2), 63.5 (CH_2), 21.5 (CH_3), 20.8 (CH_3). **HRMS (ESI+):** calculated for [$C_{21}H_{34}O_{10}SNa$] 501.17649, found 501.17621.

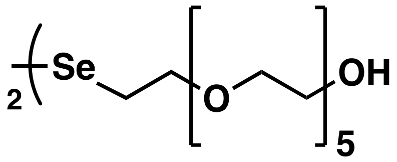

(Se-PEG$_6$)$_2$ (16)

To a solution of AcO-PEG$_6$-OTs (1.17 g, 2.44 mmol) (*see above*) in EtOH (25 mL) was added selenourea (330 mg, 2.69 mmol). The mixture was heated at reflux for 2 h before cooling to 0 ºC. An aqueous solution of NaOH (15 mL, 1 M) was added and the mixture stirred at room temperature for 2 h. The solution was acidified with 2 M HCl, extracted with CH_2Cl_2 (3 × 50 mL) dried ($MgSO_4$) and concentrated *in vacuo*. The residue was purified by flash chromatography on silica gel (3–5% MeOH in CH_2Cl_2) to give the *title compound* **16** (580 mg, 0.84 mmol, 69%) as a yellow oil.

^{1}H NMR: (400 MHz; $CDCl_3$) δ 3.73 (4H, t, *J* = 7.0 Hz, 2 × SeCH$_2$C<u>H</u>$_2$O), 3.70–3.68 (4H, m, 2 × HOC<u>H</u>$_2$), 3.65–3.60 (32H, m, 8 × OC<u>H</u>$_2$C<u>H</u>$_2$O), 3.59–3.56 (4H, m, HOCH$_2$C<u>H</u>$_2$), 3.08 (4H, t, *J* = 7.0 Hz, 2 × SeCH$_2$). **^{13}C NMR:** (101 MHz, $CDCl_3$) δ 72.5 (2 × CH_2), 71.1 (2 × CH_2), 70.48 (2 × CH_2), 70.47 (2 × CH_2), 70.4 (8 × CH_2), 70.2 (2 × CH_2), 70.0 (2 × CH_2), 61.5 (2 × CH_2), 29.0 (2 × CH_2). **HRMS (ESI+):** calculated for [$C_{24}H_{50}O_{12}Se_2Na$] 713.15263, found 713.15251.

Synthesis of biotin diselenide building block

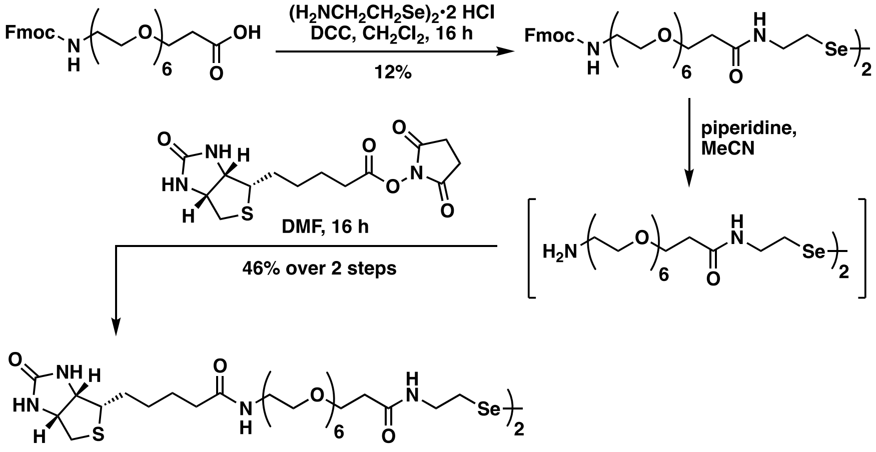

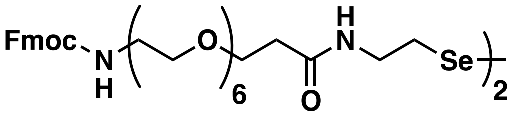

Bis((9*H*-fluoren-9-yl)methyl) (21,30-dioxo-3,6,9,12,15,18,33,36,39,42,45,48-dodecaoxa-25,26-diselena-22,29-diazapentacontane-1,50-diyl)dicarbamate

To a solution of Fmoc-*N*-amido-PEG$_6$-acid (200 mg, 0.35 mmol) in CH_2Cl_2 (3.5 mL) at 0 ºC was added *N,N'*-dicyclohexylcarbodiimide (160 mg, 0.76 mmol) followed by selenocystamine dihydrochloride (83 mg, 0.26 mmol). The mixture was stirred at room temperature for 16 h. The precipitate was removed by filtration and the filtrate concentrated *in vacuo*. The residue was purified by reverse-phase liquid chromatography (X-bridge C18 19 x 150 mm column, 0–88% B over 80 mins) to give the *title compound* (30 mg, 0.022 mmol, 12%) as a pale yellow oil.

^{1}H NMR: (400 MHz; $CDCl_3$) δ 7.76 (4H, d, *J* = 7.5 Hz, 4 × Ar-H), 7.60 (4H, d, *J* = 7.3 Hz, 4 × Ar-H), 7.39 (4H, t, *J* = 7.5 Hz, 4 × Ar-H), 7.31 (4H, t, *J* = 7.4 Hz, 4 × Ar-H), 5.62 (2H, brs, 2 × NH), 4.41 (4H, d, *J* = 6.8 Hz, 2 × CHC$\underline{H}_2$), 4.22 (2H, t, *J* = 6.7 Hz, 2 × C$\underline{H}$CH$_2$), 3.70 (4H, t, *J* = 5.6 Hz, 2 × OC$\underline{H}_2$CH$_2$C(O)NH), 3.67–3.51 (48H, m, 2 × (NHCH$_2$C$\underline{H}_2$O, 5 × OC$\underline{H}_2$C$\underline{H}_2$O and NHC$\underline{H}_2$CH$_2$Se)), 3.41–3.36 (4H, m, 2 × NHC$\underline{H}_2$CH$_2$O), 3.01 (4H, t, *J* = 6.7 Hz, 2 × C$\underline{H}_2$Se), 2.48 (4H, t, *J* = 5.7 Hz, 2 × OCH$_2$C$\underline{H}_2$C(O)NH). **^{13}C NMR:** (101 MHz; $CDCl_3$) δ 173.2 (2 × C), 156.8 (2 × C), 144.1 (4 × C), 141.5 (4 × C), 127.8 (4 × CH), 127.2 (4 × CH), 125.2 (4 × CH), 120.1 (4 × CH), 70.6 (10 × CH$_2$), 70.5 (2 × CH$_2$), 70.4 (2 × CH$_2$), 70.3 (2 × CH$_2$), 70.2 (2 × CH$_2$), 67.1 (2 × CH$_2$), 66.8 (2 × CH$_2$), 47.4 (2 × CH), 41.1 (2 × CH$_2$), 40.3 (2 × CH$_2$), 36.5 (2 × CH$_2$), 28.3 (2 × CH$_2$). **HRMS (ESI+):** calculated for [$C_{64}H_{90}N_4O_{18}Se_2$] 682.23630, found 682.23768.

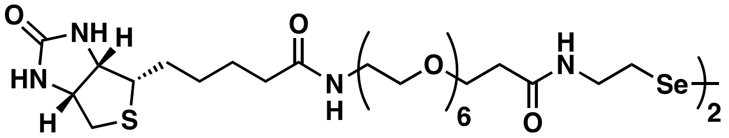

***N*,*N*'-(diselanediylbis(ethane-2,1-diyl))bis(1-(5-((3a*S*,4*S*,6a*R*)-2-oxohexahydro-1*H*-thieno[3,4-*d*]imidazol-4-yl)pentanamido)-3,6,9,12,15,18-hexaoxahenicosan-21-amide)**

To a solution of bis((9*H*-fluoren-9-yl)methyl) (21,30-dioxo-3,6,9,12,15,18,33,36,39,42,45,48-dodecaoxa-25,26-diselena-22,29-diazapentacontane-1,50-diyl)dicarbamate (18 mg, 0.013 mmol) (*see above*) in MeCN (0.4 mL) was added piperidine (8 μL, 0.08 mmol). The mixture was stirred at room temperature for 20 min. H_2O (5 mL) and Et_2O (1 mL) were added. The aqueous phase was separated and concentrated by lyophilization. The crude amine was used without purification. To a solution of the crude amine in DMF (0.3 mL) was added (+)-biotin *N*-hydroxysuccinimide ester (11 mg, 0.033 mmol). The mixture was stirred at room temperature for 16 h. The solution was concentrated under a stream of N_2. The residue was purified by reverse-phase liquid chromatography (X-bridge C18 19 x 150 mm column, 0–45% B over 50 mins) to give the *title compound* (8 mg, 0.006 mmol, 46%) as a pale yellow oil.

^{1}H NMR: (400 MHz; $CDCl_3$) δ 7.52 (2H, brs, 2 × N$\underline{H}$), 7.41 (2H, brs, 2 × N$\underline{H}$), 7.09 (2H, brs, 2 × N$\underline{H}$), 6.79 (2H, brs, 2 × N$\underline{H}$), 4.58–4.52 (2H, m, 2 × $SCH_2C\underline{H}$), 4.40–4.33 (2H, m, 2 × $SCHC\underline{H}$), 3.72 (4H, t, J = 5.8 Hz, 2 × $OC\underline{H}_2CH_2C(O)NH$), 3.66–3.53 (48H, m, 2 × ($NHCH_2C\underline{H}_2O$, 5 × $OC\underline{H}_2C\underline{H}_2O$ and $NHC\underline{H}_2CH_2Se$)), 3.45–3.38 (4H, m, 2 × $NHC\underline{H}_2CH_2O$), 3.19–3.14 (2H, m, 2 × $SC\underline{H}$), 3.01 (4H, t, J = 6.8 Hz, 2 × $SeC\underline{H}_2$), 2.91 (2H, dd, J = 12.9, 4.8 Hz, 2 × $SC\underline{H}H$), 2.75 (2H, d, J = 12.9 Hz, 2 × $SCH\underline{H}$), 2.50 (4H, t, J = 5.8 Hz, 2 × $OCH_2C\underline{H}_2C(O)NH$), 2.24 (4H, t, J = 7.2 Hz, 2 × $C\underline{H}_2C(O)NH$), 1.79–1.59 (8H, m, 2 × $SCHC\underline{H}_2CH_2C\underline{H}_2$), 1.48–1.38 (4H, m, 2 × $SCHCH_2C\underline{H}_2$). **^{13}C NMR**: (101 MHz; $CDCl_3$) δ 174.0 (2 × C), 172.3 (2 × C), 164.5 (C), 160.8 (C), 70.2 (16 × CH_2), 70.0 (4 × CH_2), 69.7 (2 × CH_2), 67.1 (2 × CH_2), 62.1 (2 × CH), 60.5 (2 × CH), 55.4 (2 × CH), 40.3 (2 × CH_2), 40.1 (2 × CH_2), 39.2 (2 × CH_2), 36.5 (2 × CH_2), 35.5 (2 × CH_2), 28.3 (2 × CH_2), 28.0 (2 × CH_2), 27.8 (2 × CH_2), 25.4 (2 × CH_2). **HRMS (ESI+):** calculated for [$C_{54}H_{98}N_8O_{18}S_2Se_2$] 1393.46632, found 1393.47037.

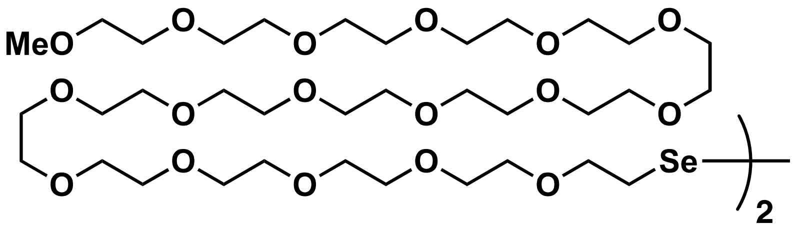

($(mPEG_{17}\text{-}Se)_2$)

To a solution of $mPEG_{17}$-OH (200 mg, 0.27 mmol) in CH_2Cl_2 (1.1 mL) at 0 ºC was added Et_3N (110 µL, 0.82 mmol) and *p*-TsCl (104 mg, 0.54 mmol). The mixture was stirred at room temperature for 16 h. CH_2Cl_2 (3 mL) and H_2O (3 mL) was added and the organic phase was separated and concentrated *in vacuo*. The crude tosylate was used without purification.

To a solution of crude tosylate in acetone (1.4 mL) was added selenourea (50 mg, 0.40 mmol). The mixture was stirred at room temperature for 3 h and concentrated *in vacuo*. The residue was dissolved in EtOH (1.5 mL) and cooled to 0 ºC. A solution of aqueous NaOH (1.5 mL, 1 M) was added. The mixture stirred at room temperature for 10 min and concentrated under a stream of N_2. The residue was purified by reverse-phase liquid chromatography (X-bridge C18 19 x 150 mm column, 0–60% B over 50 min, 0.1 vol% TFA) to give the *title compound* (22 mg, 0.026 mmol, 10%) as a white fluffy solid.

^{1}H NMR: (500 MHz; $CDCl_3$) δ 3.75 (4H, t, *J* = 6.8 Hz, 2 × $SeCH_2C\underline{H}_2$), 3.67–3.62 (128H, m, 64 × -$C\underline{H}_2$-), 3.56–3.53 (4H, m, 2 × $C\underline{H}_2OCH_3$), 3.38 (6H, s, 2 × $C\underline{H}_3$), 3.10 (4H, t, *J* = 6.9 Hz, 2 × $SeC\underline{H}_2$). **^{13}C NMR:** (126 MHz; $CDCl_3$) δ 72.1 (2 × $C\underline{H}_2$), 71.4 (2 × $C\underline{H}_2$), 70.8 (2 × $C\underline{H}_2$), 70.7 (56 × $C\underline{H}_2$), 70.6 (2 × $C\underline{H}_2$), 70.4 (2 × $C\underline{H}_2$), 59.2 (2 × $C\underline{H}_3$), 29.3 (2 × $C\underline{H}_2$). **HRMS (ESI+):** calculated for [$C_{63}H_{144}N_6O_{34}Se_2$] 844.40215, found 844.40202.

Synthesis of 1-Se GalNAc building block

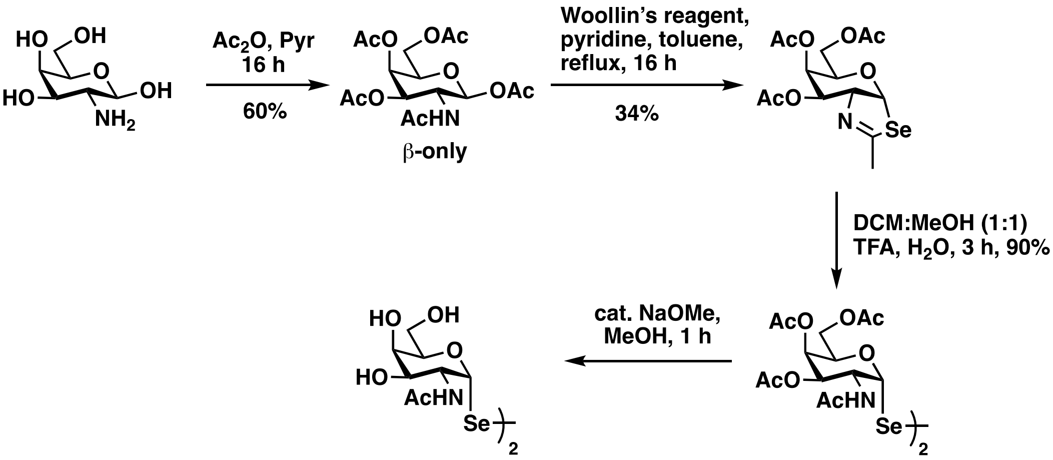

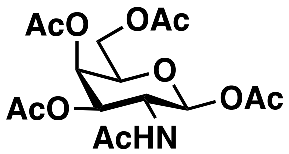

(2*S*,3*R*,4*R*,5*R*,6*R*)-3-acetamido-6-(acetoxymethyl)tetrahydro-2*H*-pyran-2,4,5-triyl triacetate

To a solution of galactosamine hydrochloride (5.00 g, 23.2 mmol) in pyridine (38 mL) at 0 ºC was added Ac_2O (25 mL, 0.26 mol). The mixture was stirred at room temperature for 16 h. Toluene (10 mL) was added and the solution concentrated *in vacuo*. To the residue was added EtOAc (150 mL) forming an off-white precipitate. The solid was collected by vacuum filtration and washed with HCl (3 × 75 mL, 0.1 M), water (2 × 75 mL), EtOH (2 × 75 mL) and Et_2O (3 × 100 mL) to give the *title compound* (5.42 g, 13.9 mmol, 60%) as an off-white solid.

^{1}H NMR: (500 MHz; $CDCl_3$) δ 5.69 (1H, d, *J* = 8.8 Hz, AcOC<u>H</u>O), 5.37 (1H, d, *J* = 3.30 Hz, N<u>H</u>), 5.08 (1H, dd, *J* = 11.3, 3.3 Hz, C<u>H</u>CHCHCH$_2$), 4.45 (1H, dt, *J* = 11.3, 9.3 Hz, HNC<u>H</u>), 4.19–4.09 (2H, m, C<u>H</u>$_2$), 4.02 (1H, td, *J* = 6.5, 0.8 Hz, C<u>H</u>CH$_2$), 2.17 (3H, s, Ac), 2.13 (3H, s, Ac), 2.05 (3H, s, Ac), 2.02 (3H, s, Ac), 1.94 (3H, s, Ac). **^{13}C NMR:** (126 MHz; $CDCl_3$) δ 170.8 (C), 170.4 (C), 170.3 (C), 170.2 (C), 169.6 (C), 93.1 (CH), 71.9 (CH), 70.4 (CH), 66.3 (CH), 61.3 (CH_2), 49.8 (CH), 23.3 (CH_3), 20.9 (CH_3), 20.7 (2 × CH_3), 20.6 (CH_3). **HRMS (ESI+):** calculated for [$C_{16}H_{23}NO_{10}Na$] 412.12142, found 412.12151.

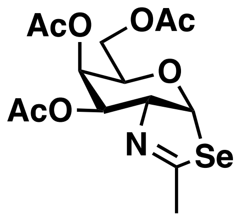

(3a*R*,5*R*,6*R*,7*R*,7a*R*)-5-(acetoxymethyl)-2-methyl-3a,6,7,7a-tetrahydro-5*H*-pyrano[3,2-*d*][1,3]selenazole-6,7-diyl diacetate

To a solution of (2*S*,3*R*,4*R*,5*R*,6*R*)-3-acetamido-6-(acetoxymethyl)tetrahydro-2*H*-pyran-2,4,5-triyl triacetate (439 mg, 0.977 mmol) (*see above*) and Woollins' reagent (778 mg, 1.46 mmol) in toluene (5.2 mL) was added pyridine (870 μL, 11 mmol). The mixture was heated at reflux for 16 h before concentrating under a stream of N_2. The residue was purified by flash chromatography on silica gel (20–60% EtOAc in hexanes) to give the *title compound* (130 mg, 0.331 mmol, 34%) as a yellow oil.

^{1}H NMR: (400 MHz; $CDCl_3$) δ 6.83 (1H, d, *J* = 5.1 Hz, SeC<u>H</u>), 5.47 (1H, dd, *J* = 3.3, 2.3 Hz, C<u>H</u>CHCH$_2$), 5.26 (1H, dd, *J* = 8.6, 3.2 Hz, SeCHCHC<u>H</u>), 4.42–4.38 (1H, m, SeCHC<u>H</u>), 4.29 (1H, td, *J* = 6.5, 2.2 Hz, C<u>H</u>CH$_2$), 4.20–4.11 (2H, m, C<u>H</u>$_2$), 2.37 (3H, s, <u>H</u>$_3$CC(N)Se), 2.14 (3H, s, <u>H</u>$_3$CCO$_2$CH), 2.08 (3H, s, NHCHCHO$_2$CC<u>H</u>$_3$), 2.05 (3H, s, <u>H</u>$_3$CCO$_2$CH$_2$). **^{13}C NMR:** (101 MHz; CD_3OD) δ 171.3 (C), 170.6 (C), 170.4 (C), 170.0 (C), 92.1 (CH), 73.8 (CH), 71.4 (CH), 70.7 (CH), 66.0 (CH), 61.4 (CH$_2$), 25.8 (CH$_3$), 21.0 (CH$_3$), 20.84 (CH$_3$), 20.75 (CH$_3$). **HRMS (ESI+):** calculated for [$C_{14}H_{20}NO_7Se$] 394.03995, found 394.04046.

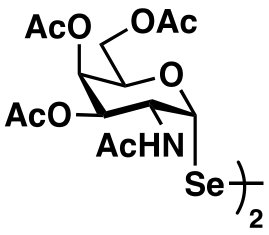

(2*R*,2'*R*,3*R*,3'*R*,4*R*,4'*R*,5*R*,5'*R*,6*R*,6'*R*)-diselanediylbis(5-acetamido-2-(acetoxymethyl)tetrahydro-2*H*-pyran-6,3,4-triyl) tetraacetate

To a solution of (3a*R*,5*R*,6*R*,7*R*,7a*R*)-5-(acetoxymethyl)-2-methyl-3a,6,7,7a-tetrahydro-5*H*-pyrano[3,2-*d*][1,3]selenazole-6,7-diyl diacetate (78 mg, 0.20 mmol) (*see above*) in CH_2Cl_2 (0.4 mL) and MeOH (0.4 mL) at 0 ºC was added dropwise TFA (104 µL) followed by H_2O (104 µL). The mixture was stirred at room temperature for 3 h before concentrating *in vacuo*. The residue was purified by flash chromatography on silica gel (2% MeOH in EtOAc) to give the *title compound* (74 mg, 0.090 mmol, 90%) as a yellow oil.

^{1}H NMR: (400 MHz; CD_3OD) δ 8.33 (2H, d, *J* = 6.9 Hz, 2 × N<u>H</u>), 6.10 (2H, d, *J* = 5.1 Hz, 2 × SeC<u>H</u>), 5.49 (2H, d, *J* = 2.2 Hz, 2 × C<u>H</u>CHCH$_2$), 5.06 (2H, dd, *J* = 11.9, 3.1 Hz, 2 × SeCHCHC<u>H</u>), 4.50–4.43 (2H, m, 2 × SeCHC<u>H</u>), 4.42 (2H, t, *J* = 6.4 Hz, 2 × C<u>H</u>CH$_2$), 4.20–4.11 (4H, m, 2 × C<u>H</u>$_2$), 2.13 (6H, s, 2 × <u>H</u>$_3$CCO$_2$CH), 2.06 (6H, s, 2 × <u>H</u>$_3$CCO$_2$CH$_2$), 1.97 (12H, s, 2 × <u>H</u>$_3$CC(O)NHCHCHO$_2$CC<u>H</u>$_3$). **^{13}C NMR:** (101 MHz; CD_3OD) δ 173.9 (2 × C), 172.2 (2 × C), 171.9 (2 × C), 171.7 (2 × C), 89.2 (2 × CH), 71.2 (2 × CH), 69.8 (2 × CH), 68.2 (2 × CH), 62.8 (2 × CH_2), 50.7 (2 × CH), 22.5 (2 × CH_3), 20.7 (2 × CH_3), 20.6 (2 × CH_3), 20.4 (2 × CH_3). **HRMS (ESI+):** calculated for [$C_{28}H_{41}N_2O_{16}Se_2$] 821.07810, found 821.07805.

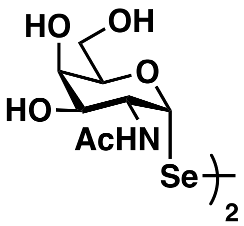

***N*,*N*'-((2*R*,2'*R*,3*R*,3'*R*,4*R*,4'*R*,5*R*,5'*R*,6*R*,6'*R*)-diselanediylbis(4,5-dihydroxy-6-(hydroxymethyl)tetrahydro-2*H*-pyran-2,3-diyl))diacetamide**

To a solution of (2*R*,2'*R*,3*R*,3'*R*,4*R*,4'*R*,5*R*,5'*R*,6*R*,6'*R*)-diselanediylbis(5-acetamido-2-(acetoxymethyl)tetrahydro-2*H*-pyran-6,3,4-triyl) tetraacetate (29 mg, 0.035 mmol) (*see above)* in MeOH (0.4 mL) at 0 ºC was added a solution of NaOMe in MeOH (400 µL, 0.5 M). The mixture was stirred at room temperature for 1 h. The solution was neutralized with amberlite resin, filtered and washed with a 1:1 solution of MeCN and H_2O (10 mL) before concentrating by lyophilization to give the *title compound* as a pale red solid which was used without purification.

^{1}H NMR (400 MHz; CD_3OD) δ: 5.98 (2H, d, *J* = 5.0 Hz, 2 × SeC<u>H</u>), 4.27 (2H, dd, *J* = 11.3, 5.0 Hz, 2 × SeCHC<u>H</u>), 3.99–3.92 (4H, m, 2 × SeCHCHC<u>H</u> and C<u>H</u>CH_2), 3.82–3.71 (6H, m, 2 × C<u>H</u>CHC<u>H</u>$_2$), 1.99 (6H, s, 2 × <u>H</u>$_3$C); **LRMS (ESI$^+$):** calculated for [$C_{16}H_{28}N_2O_{10}Se_2$] 570.0, 568.0, 566.0, 564.0, found 569.8, 568.9, 566.9 and 564.9.

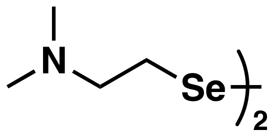

2,2'-diselanediylbis(*N*,*N*-dimethylethan-1-amine): Synthesis adapted from Kerdraon *et al.*[59]

To a solution of K_2CO_3 (11.5 g, 83 mmol) in H_2O (30 mL) was added 1-chloro-2-dimethylaminoethane hydrochloride (6.0 g, 42 mmol). The mixture was stirred at room temperature for 5 min. H_2O (20 mL) was added before extracting with Et_2O (3 × 50 mL). The organic phase was dried (Na_2SO_4) and concentrated *in vacuo*. The residue was taken up in MeCN (90 mL) and KSeCN (1.5 g, 10 mmol) was slowly added. The mixture was stirred at room temperature for 20 h. Et_2O (200 mL) was then added and the solution filtered through celite. The filtrate was concentrated *in vacuo*. The residue was taken up in EtOH (125 mL) and cooled to 0 ºC. A solution of aqueous NaOH (60 mL, 1 M) was then added dropwise. The mixture stirred at room temperature for 2.5 h before concentrating under a stream of N_2. H_2O (20 mL) was added and the aqueous phase extracted with Et_2O (3 × 75 mL). The organic extracts were dried (Na_2SO_4) and concentrated *in vacuo* to afford the *title compound* (1.1 g, 3.7 mmol, 18%) as a yellow oil.

^{1}H NMR: (400 MHz; $CDCl_3$) δ 3.09–3.00 (4H, m, 2 × SeC$\underline{H}_2$), 2.68–2.59 (4H, m, 2 × NC$\underline{H}_2$), 2.26 (12H, s, 4 × C$\underline{H}_3$). **^{13}C NMR:** (101 MHz; $CDCl_3$) δ 60.5 (2 × CH_2), 45.4 (4 × CH_3), 28.4 (2 × CH_2). **HRMS (ESI+):** calculated for [$C_8H_{21}N_2Se_2$] 305.00297, found 305.00366.

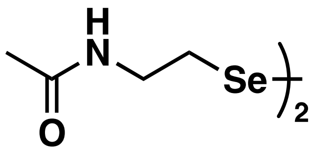

N,N'-(diselanediylbis(ethane-2,1-diyl))diacetamide

To a solution of selenocystamine dihydrochloride (100 mg, 0.31 mmol) in CH_2Cl_2 (5 mL) at 0 ºC was added dropwise *i*Pr_2NEt (420 μL, 2.4 mmol) and Ac_2O (300 μL, 1.6 mmol). The mixture was stirred at room temperature for 1 h. CH_2Cl_2 (30 mL) and HCl (30 mL, 1 M) were added and the aqueous phase extracted with CH_2Cl_2 (3 × 50 mL). The combined organic extracts were washed with HCl (50 mL, 1 M), brine (50 mL), dried (Na_2SO_4) and concentrated *in vacuo*. The residue was purified by flash chromatography on silica gel (5–10% MeOH in CH_2Cl_2) to give the *title compound* (64 mg, 0.19 mmol, 63%) as a yellow solid.

^{1}H NMR: (400 MHz; $CDCl_3$) δ 6.39 (2H, brs, 2 × N$\underline{H}$), 3.59 (4H, q, J = 6.4 Hz, 2 × NHC$\underline{H}_2$), 3.02 (4H, t, J = 6.5 Hz, 2 × $SeCH_2$), 2.01 (6H, s, 2 × C$\underline{H}_3$). **^{13}C NMR:** (101 MHz; $CDCl_3$) δ 170.5 (2 × C), 40.1 (2 × CH_2), 28.3 (2 × CH_2), 23.1 (2 × CH_3). **HRMS (ESI+):** calculated for [$C_8H_{17}N_2O_2Se_2$] 332.96150, found 332.96152.

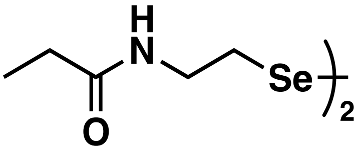

N,N'-(diselanediylbis(ethane-2,1-diyl))dipropionamide

To a solution of selenocystamine dihydrochloride (200 mg, 0.63 mmol) in CH_2Cl_2 (6 mL) at 0 °C was added dropwise *i*Pr_2NEt (540 µL, 3.1 mmol) and propionyl chloride (140 µL, 1.6 mmol). The mixture was stirred at room temperature for 1.5 h. CH_2Cl_2 (30 mL) and HCl (30 mL, 1 M) were added and the aqueous phase extracted with CH_2Cl_2 (3 × 50 mL). The combined organic extracts were washed with HCl (50 mL, 1 M), brine (50 mL), dried (Na_2SO_4) and concentrated *in vacuo*. The residue was purified by flash chromatography on silica gel (5–8% MeOH in CH_2Cl_2) to give the *title compound* (164 mg, 0.46 mmol, 73%) as a yellow solid.

^{1}H NMR: (400 MHz; $CDCl_3$) δ 6.24 (2H, brs, 2 × N$\underline{H}$), 3.61 (4H, q, J = 6.4 Hz, 2 × NHC$\underline{H}_2$), 3.03 (4H, t, J = 6.6 Hz, 2 × SeC$\underline{H}_2$), 2.25 (4H, q, J = 7.6 Hz, 2 × C$\underline{H}_2CH_3$), 1.17 (6H, t, J = 7.6 Hz, 2 × C$\underline{H}_3$). **^{13}C NMR:** (101 MHz; $CDCl_3$) δ 174.5 (2 × C), 40.1 (2 × CH_2), 29.7 (2 × CH_2), 28.7 (2 × CH_2), 9.9 (2 × CH_3). **HRMS (ESI+):** calculated for [$C_{10}H_{21}N_2O_2Se_2$] 360.99280, found 360.99324.

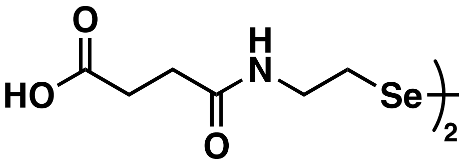

4,4'-((diselanediylbis(ethane-2,1-diyl))bis(azanediyl))bis(4-oxobutanoic acid): Synthesis adapted from Guenther, W. H. H.[60]

To a solution of selenocystamine dihydrochloride (500 mg, 1.56 mmol) and $NaHCO_3$ (656 mg, 7.81 mmol) in H_2O (2.2 mL) at 0 ºC was added portion-wise succinic anhydride (313 mg, 3.13 mmol). The mixture was stirred at room temperature for 1 h before acidifying with concentrated HCl to pH 1–2. The precipitate was collected by filtration and purified by recrystallisation in water to give the *title compound* (385 mg, 0.86 mmol, 55%) as a pale yellow solid.

^{1}H NMR: (400 MHz; CD_3OD) δ 3.52 (4H, t, *J* = 7.0 Hz, 2 × NHC$\underline{H}_2$), 3.02 (4H, t, *J* = 7.0 Hz, 2 × SeC$\underline{H}_2$), 2.61–2.55 (4H, m, 2 × NHC(O)C$\underline{H}_2$), 2.50–2.45 (4H, m, 2 × C$\underline{H}_2$$CO_2$H). **^{13}C NMR:** (101 MHz; CD_3OD) δ 176.4 (2 × C), 174.6 (2 × C), 41.4 (2 × CH_2), 31.7 (2 × CH_2), 30.4 (2 × CH_2), 29.2 (2 × CH_2). **HRMS (ESI+):** calculated for [$C_{12}H_{20}N_2O_6Se_2Na$] 470.95440, found 470.95527

Plasmid DNA Sequence of pUC-PT7-His$_6$-MBP-TEV-CaM (K148U)

** ORF in bold. CaM (K148U) gene underlined.

GCGCCCAATACGCAAACCGCCTCTCCCCGCGCGTTGGCCGATTCATTAATGCAGCTGGCACG
ACAGGTTTCCCGACTGGAAAGCGGGCAGTGAGCGCAACGCAATTAATGTGAGTTAGCTCACG
GATCTCGACGCTCTCCCTTATGCGACTCCTGCAGTACACGGCCGCATAATCGAAATTAATAC
GACTCACTATAGGAGATCTCTCTATCACTGATAGGGACCATCTTAGTATATTAGTTAAGTAT
AAGAAGGAGATATACAT**ATGGGCAGCAGCCATCATCATCATCATCACGGTACCAAAACTGAA**
GAAGGTAAACTGGTAATCTGGATTAACGGCGATAAAGGCTATAACGGTCTCGCTGAAGTCGG
TAAGAAATTCGAGAAAGATACCGGAATTAAAGTCACCGTTGAGCATCCGGATAAACTGGAAG
AGAAATTCCCACAGGTTGCGGCAACTGGCGATGGCCCTGACATTATCTTCTGGGCACACGAC
CGCTTTGGTGGCTACGCTCAATCTGGCCTGTTGGCTGAAATCACCCCGGACAAAGCGTTCCA
GGACAAGCTGTATCCGTTTACCTGGGATGCCGTACGTTACAACGGCAAGCTGATTGCTTACC
CGATCGCTGTTGAAGCGTTATCGCTGATTTATAACAAAGATCTGCTGCCGAACCCGCCAAAA
ACCTGGGAAGAGATCCCGGCGCTGGATAAAGAACTGAAAGCGAAAGGTAAGAGCGCGCTGAT
GTTCAACCTGCAAGAACCGTACTTCACCTGGCCGCTGATTGCTGCTGACGGGGGTTATGCGT
TCAAGTATGAAAACGGCAAGTACGACATTAAAGACGTGGGCGTGGATAACGCTGGCGCGAAA
GCGGGTCTGACCTTCCTGGTTGACCTGATTAAAAACAAACACATGAATGCAGACACCGATTA
CTCCATCGCAGAAGCTGCCTTTAATAAAGGCGAAACAGCGATGACCATCAACGGCCCGTGGG
CATGGTCCAACATCGACACCAGCAAAGTGAATTATGGTGTAACGGTACTGCCGACCTTCAAG
GGTCAACCATCCAAACCGTTCGTTGGCGTGCTGAGCGCAGGTATTAACGCCGCCAGTCCGAA
CAAAGAGCTGGCGAAAGAGTTCCTCGAAAACTATCTGCTGACTGATGAAGGTCTGGAAGCGG
TTAATAAAGACAAACCGCTGGGTGCCGTAGCGCTGAAGTCTTACGAGGAAGAGTTGGCGAAA
GATCCACGTATTGCCGCCACCATGGAAAACGCCCAGAAAGGTGAAATCATGCCGAACATCCC
GCAGATGTCCGCTTTCTGGTATGCCGTGCGTACTGCGGTGATCAACGCCGCCAGCGGTCGTC
AGACTGTCGATGAAGCCCTGAAAGACGCGCAGACTGGTACCGATTACGATATCCCAACGACC
GAAAACCTTTACTTCCAGGGCGCAGATCAGCTGACCGAAGAACAAATTGCCGAATTTAAAGA
AGCCTTCAGCCTGTTCGATAAAGATGGTGATGGCACCATTACCACCAAAGAACTGGGCACCG
TTATGCGTAGCCTGGGTCAGAATCCGACCGAAGCAGAACTGCAGGATATGATTAATGAAGTT
GATGCAGATGGCAACGGCACCATTGATTTTCCGGAATTTCTGACCATGATGGCACGCAAAAT
GAAAGATACCGATAGCGAAGAAGAAATCCGCGAAGCATTTCGTGTCTTTGATAAAGACGGCA
ATGGTTATATTTCAGCAGCCGAACTGCGTCATGTTATGACCAATCTGGGTGAAAAACTGACC
GATGAAGAGGTGGATGAAATGATTCGTGAAGCAGATATTGATGGTGACGGTCAGGTTAACTA
TGAAGAATTTGTTCAGATGATGACCGCCTAGTAACTCGAGTCTGGTAAAGAAACCGCTGCTG
CGAAATTTGAACGCCAGCACATGGACTCGTCTACTAGCGCAGCTTAATTAACCTAGGCTGCT
GCCACCGCTGAGCAATAACTAGCATAACCCCTTGGGGCCTCTAAACGGGTCTTGAGGGGTTT
TTTGCTGAAACCTCAGGCATTTGAGAAGCACACGGTCACACTGCTTCCGGTAGTCAATAAAC
CGGTAAACCAGCAATAGACATAAGCGGCTATTTAACGACCCTGCCCTGAACCGACGACCGGG
TCGAATTTGCTTTCGAATTTCTGCCATTCATCCGCTTATTATCACTTATTCAGGCGTAGCAC
CAGGCGTTTAAGGGCACCAATAACTGCCTTAAAAAAATTACGCCCCGCCCTGCCACTCATCG
CAGTACTGTTGTAATTCATTAAGCATTCTGCCGACATGGAAGCCATCACAGACGGCATGATG
AACCTGAATCGCCAGCGGCATCAGCACCTTGTCGCCTTGCGTATAATATTTGCCCATAGTGA
AAACGGGGGCGAAGAAGTTGTCCATATTGGCCACGTTTAAATCAAAACTGGTGAAACTCACC
CAGGGATTGGCTGAGACGAAAAACATATTCTCAATAAACCCTTTAGGGAAATAGGCCAGGTT
TTCACCGTAACACGCCACATCTTGCGAATATATGTGTAGAAACTGCCGGAAATCGTCGTGGT
ATTCACTCCAGAGCGATGAAAACGTTTCAGTTTGCTCATGGAAAACGGTGTAACAAGGGTGA
ACACTATCCCATATCACCAGCTCACCGTCTTTCATTGCCATACGGAACTCCGGATGAGCATT
CATCAGGCGGGCAAGAATGTGAATAAAGGCCGGATAAAACTTGTGCTTATTTTTCTTTACGG
TCTTTAAAAAGGCCGTAATATCCAGCTGAACGGTCTGGTTATAGGTACATTGAGCAACTGAC
TGAAATGCCTCAAAATGTTCTTTACGATGCCATTGGGATATATCAACGGTGGTATATCCAGT

GATTTTTTTCTCCATTTTAGCTTCCTTAGCTCCTGAAAATCTCGATAACTCAAAAAATACGC
CCGGTAGTGATCTTATTTCATTATGGTGAAAGTTGGAACCTCTTACGTGCCGATCAACGTCT
CATTTTCGCCAAAAGTTGGCCCAGGGCTTCCCGGTATCAACAGGGACACCAGGATTTATTTA
TTCTGCGAAGTGATCTTCCGTCACAGGTATTTCTCATGACCAAAATCCCTTAACGTGAGTTT
TCGTTCCACTGAGCGTCAGACCCCGTAGAAAAGATCAAAGGATCTTCTTGAGATCCTTTTTT
TCTGCGCGTAATCTGCTGCTTGCAAACAAAAAAACCACCGCTACCAGCGGTGGTTTGTTTGC
CGGATCAAGAGCTACCAACTCTTTTTCCGAAGGTAACTGGCTTCAGCAGAGCGCAGATACCA
AATACTGTTCTTCTAGTGTAGCCGTAGTTAGGCCACCACTTCAAGAACTCTGTAGCACCGCC
TACATACCTCGCTCTGCTAATCCTGTTACCAGTGGCTGCTGCCAGTGGCGATAAGTCGTGTC
TTACCGGGTTGGACTCAAGACGATAGTTACCGGATAAGGCGCAGCGGTCGGGCTGAACGGGG
GGTTCGTGCACACAGCCCAGCTTGGAGCGAACGACCTACACCGAACTGAGATACCTACAGCG
TGAGCTATGAGAAAGCGCCACGCTTCCCGAAGGGAGAAAGGCGGACAGGTATCCGGTAAGCG
GCAGGGTCGGAACAGGAGAGCGCACGAGGGAGCTTCCAGGGGGAAACGCCTGGTATCTTTAT
AGTCCTGTCGGGTTTCGCCACCTCTGACTTGAGCGTCGATTTTTGTGATGCTCGTCAGGGGG
GCGGAGCCTATGGAAAAACGCCAGCAACGCGGCCTTTTTACGGTTCCTGGCCTTTTGCTGGC
CTTTTGCTCACATGTTCTTTCCTGCGTTATCCCCTGATTCTGTGGATAACCGTATTACCGCC
TTTGAGTGAGCTGATACCGCTCGCCGCAGCCGAACGACCGAGCGCAGCGAGTCAGTGAGCGA
GGAAGCGGAAGA

Plasmid Sequence for pTXB1-Ub-Mxe-His-CBD

ORF in bold. Ub gene underlined. For the K48C mutant, the AAA codon in italics grey was replaced by TGC

ATGCAGATCTTCGTGAAGACTCTGACTGGTAAGACCATCACTCTCGAAGTGGAGCCGAGTGA
CACCATTGAGAATGTCAAGGCAAAGATCCAAGACAAGGAAGGCATCCCTCCTGACCAGCAGA
GGTTGATCTTTGCTGGGAAACAGCTGGAAGATGGACGCACCCTGTCTGACTACAACATCCAG
AAAGAGTCCACCCTGCACCTGGTACTCCGTCTCAGAGGTGGTTGCATCACGGGAGATGCAct
agTTGCCCTACCCGAGGGCGAGTCGGTACGCATCGCCGACATCGTGCCGGGTGCGCGGCCCA
ACAGTGACAACGCCATCGACCTGAAAGTCCTTGACCGGCATGGCAATCCCGTGCTCGCCGAC
CGGCTGTTCCACTCCGGCGAGCATCCGGTGTACACGGTGCGTACGGTCGAAGGTCTGCGTGT
GACGGGCACCGCGAACCACCCGTTGTTGTGTTTGGTCGACGTCGCCGGGGTGCCGACCCTGC
TGTGGAAGCTGATCGACGAAATCAAGCCGGGCGATTACGCGGTGATTCAACGCAGCGCATTC
AGCGTCGACTGTGCAGGTTTTGCCCGCGGAAAACCCGAATTTGCGCCCACAACCTACACAGT
CGGCGTCCCTGGACTGGTGCGTTTCTTGGAAGCACACCACCGAGACCCGGACGCCCAAGCTA
TCGCCGACGAGCTGACCGACGGGCGGTTCTACTACGCGAAAGTCGCCAGTGTCACCGACGCC
GGCGTGCAGCCGGTGTATAGCCTTCGTGTCGACACGGCAGACCACGCGTTTATCACGAACGG
GTTCGTCAGCCACGCTACTGGCCTCACCGGAATTCACCACCACCACCACCACTCCGGTC
TGAACTCAGGCCTCACGACAAATCCTGGTGTATCCGCTTGGCAGGTCAACACAGCTTATACT
GCGGGACAATTGGTCACATATAACGGCAAGACGTATAAATGTTTGCAGCCCCACACCTCCTT
GGCAGGATGGGAACCATCCAACGTTCCTGCCTTGTGGCAGCTTCAATGACTGCAGGAAGGGG
ATCCGGCTGCTAACAAAGCCCGAAAGGAAGCTGAGTTGGCTGCTGCCACCGCTGAGCAATAA
CTAGCATAACCCCTTGGGGCCTCTAAACGGGTCTTGAGGGGTTTTTTGCTGAAAGGAGGAAC
TATATCCGGATAACTACGTCAGGTGGCACTTTTCGGGGAAATGTGCGCGGAACCCCTATTTG
TTTATTTTTCTAAATACATTCAAATATGTATCCGCTCATGAGACAATAACCCTGATAAATGC
TTCAATAATATTGAAAAAGGAAGAGTATGAGTATTCAACATTTCCGTGTCGCCCTTATTCCC
TTTTTTGCGGCATTTTGCCTTCCTGTTTTTGCTCACCCAGAAACGCTGGTGAAAGTAAAAGA
TGCTGAAGATCAGTTGGGTGCACGAGTGGGTTACATCGAACTGGATCTCAACAGCGGTAAGA

TCCTTGAGAGTTTTCGCCCCGAAGAACGTTCTCCAATGATGAGCACTTTTAAAGTTCTGCTA
TGTGGCGCGGTATTATCCCGTGTTGACGCCGGGCAAGAGCAACTCGGTCGCCGCATACACTA
TTCTCAGAATGACTTGGTTGAGTACTCACCAGTCACAGAAAAGCATCTTACGGATGGCATGA
CAGTAAGAGAATTATGCAGTGCTGCCATAACCATGAGTGATAACACTGCGGCCAACTTACTT
CTGACAACGATCGGAGGACCGAAGGAGCTAACCGCTTTTTTGCACAACATGGGGGATCATGT
AACTCGCCTTGATCGTTGGGAACCGGAGCTGAATGAAGCCATACCAAACGACGAGCGTGACA
CCACGATGCCTGTAGCAATGGCAACAACGTTGCGCAAACTATTAACTGGCGAACTACTTACT
CTAGCTTCCCGGCAACAATTAATAGACTGGATGGAGGCGGATAAAGTTGCAGGACCACTTCT
GCGCTCGGCCCTTCCGGCTGGCTGGTTTATTGCTGATAAATCTGGAGCCGGTGAGCGTGGGT
CTCGCGGTATCATTGCAGCACTGGGGCCAGATGGTAAGCCCTCCCGTATCGTAGTTATCTAC
ACGACGGGGAGTCAGGCAACTATGGATGAACGAAATAGACAGATCGCTGAGATAGGTGCCTC
ACTGATTAAGCATTGGTAACTGTCAGACCAAGTTTACTCATATATACTTTAGATTGATTTAC
CCCGGTTGATAATCAGAAAAGCCCCAAAAACAGGAAGATTGTATAAGCAAATATTTAAATTG
TAAACGTTAATATTTTGTTAAAATTCGCGTTAAATTTTTGTTAAATCAGCTCATTTTTTAAC
CAATAGGCCGAAATCGGCAAAATCCCTTATAAATCAAAAGAATAGCCCGAGATAGGGTTGAG
TGTTGTTCCAGTTTGGAACAAGAGTCCACTATTAAAGAACGTGGACTCCAACGTCAAAGGGC
GAAAAACCGTCTATCAGGGCGATGGCCCACTACGTGAACCATCACCCAAATCAAGTTTTTTG
GGGTCGAGGTGCCGTAAAGCACTAAATCGGAACCCTAAAGGGAGCCCCCGATTTAGAGCTTG
ACGGGGAAAGCCGGCGAACGTGGCGAGAAAGGAAGGGAAGAAAGCGAAAGGAGCGGGCGCTA
GGGCGCTGGCAAGTGTAGCGGTCACGCTGCGCGTAACCACCACACCCGCCGCGCTTAATGCG
CCGCTACAGGGCGCGTAAAAGGATCTAGGTGAAGATCCTTTTTGATAATCTCATGACCAAAA
TCCCTTAACGTGAGTTTTCGTTCCACTGAGCGTCAGACCCCGTAGAAAAGATCAAAGGATCT
TCTTGAGATCCTTTTTTTCTGCGCGTAATCTGCTGCTTGCAAACAAAAAAACCACCGCTACC
AGCGGTGGTTTGTTTGCCGGATCAAGAGCTACCAACTCTTTTTCCGAAGGTAACTGGCTTCA
GCAGAGCGCAGATACCAAATACTGTCCTTCTAGTGTAGCCGTAGTTAGGCCACCACTTCAAG
AACTCTGTAGCACCGCCTACATACCTCGCTCTGCTAATCCTGTTACCAGTGGCTGCTGCCAG
TGGCGATAAGTCGTGTCTTACCGGGTTGGACTCAAGACGATAGTTACCGGATAAGGCGCAGC
GGTCGGGCTGAACGGGGGGTTCGTGCACACAGCCCAGCTTGGAGCGAACGACCTACACCGAA
CTGAGATACCTACAGCGTGAGCTATGAGAAAGCGCCACGCTTCCCGAAGGGAGAAAGGCGGA
CAGGTATCCGGTAAGCGGCAGGGTCGGAACAGGAGAGCGCACGAGGGAGCTTCCAGGGGGAA
ACGCCTGGTATCTTTATAGTCCTGTCGGGTTTCGCCACCTCTGACTTGAGCGTCGATTTTTG
TGATGCTCGTCAGGGGGGCGGAGCCTATGGAAAAACGCCAGCAACGCGGCCTTTTTACGGTT
CCTGGCCTTTTGCTGGCCTTTTGCTCACATGTTCTTTCCTGCGTTATCCCCTGATTCTGTGG
ATAACCGTATTACCGCCTTTGAGTGAGCTGATACCGCTCGCCGCAGCCGAACGACCGAGCGC
AGCGAGTCAGTGAGCGAGGAAGCTATGGTGCACTCTCAGTACAATCTGCTCTGATGCCGCAT
AGTTAAGCCAGTATACACTCCGCTATCGCTACGTGACTGGGTCATGGCTGCGCCCCGACACC
CGCCAACACCCGCTGACGCGCCCTGACGGGCTTGTCTGCTCCCGGCATCCGCTTACAGACAA
GCTGTGACCGTCTCCGGGAGCTGCATGTGTCAGAGGTTTTCACCGTCATCACCGAAACGCGC
GAGGCAGCTGCGGTAAAGCTCATCAGCGTGGTCGTGCAGCGATTCACAGATGTCTGCCTGTT
CATCCGCGTCCAGCTCGTTGAGTTTCTCCAGAAGCGTTAATGTCTGGCTTCTGATAAAGCGG
GCCATGTTAAGGGCGGTTTTTTCCTGTTTGGTCACTGATGCCTCCGTGTAAGGGGGATTTCT
GTTCATGGGGGTAATGATACCGATGAAACGAGAGAGGATGCTCACGATACGGGTTACTGATG
ATGAACATGCCCGGTTACTGGAACGTTGTGAGGGTAAACAACTGGCGGTATGGATGCGGCGG
GACCAGAGAAAAATCACTCAGGGTCAATGCCAGCCGAACGCCAGCAAGACGTAGCCCAGCGC
GTCGGCCGCCATGCCGGCGATAATGGCCTGCTTCTCGCCGAAACGTTTGGTGGCGGGACCAG
TGACGAAGGCTTGAGCGAGGGCGTGCAAGATTCCGAATACCGCAAGCGACAGGCCGATCATC
GTCGCGCTCCAGCGAAAGCGGTCCTCGCCGAAAATGACCCAGAGCGCTGCCGGCACCTGTCC
TACGAGTTGCATGATAAAGAAGACAGTCATAAGTGCGGCGACGATAGTCATGCCCCGCGCCC
ACCGGAAGGAGCTGACTGGGTTGAAGGCTCTCAAGGGCATCGGTCGAGATCCCGGTGCCTAA
TGAGTGAGCTAACTTACATTAATTGCGTTGCGCTCACTGCCCGCTTTCCAGTCGGGAAACCT
GTCGTGCCAGCTGCATTAATGAATCGGCCAACGCGCGGGGAGAGGCGGTTTGCGTATTGGGC

GCCAGGGTGGTTTTTCTTTTCACCAGTGAGACGGGCAACAGCTGATTGCCCTTCACCGCCTG
GCCCTGAGAGAGTTGCAGCAAGCGGTCCACGCTGGTTTGCCCCAGCAGGCGAAAATCCTGTT
TGATGGTGGTTAACGGCGGGATATAACATGAGCTGTCTTCGGTATCGTCGTATCCCACTACC
GAGATATCCGCACCAACGCGCAGCCCGGACTCGGTAATGGCGCGCATTGCGCCCAGCGCCAT
CTGATCGTTGGCAACCAGCATCGCAGTGGGAACGATGCCCTCATTCAGCATTTGCATGGTTT
GTTGAAAACCGGACATGGCACTCCAGTCGCCTTCCCGTTCCGCTATCGGCTGAATTTGATTG
CGAGTGAGATATTTATGCCAGCCAGCCAGACGCAGACGCGCCGAGACAGAACTTAATGGGCC
CGCTAACAGCGCGATTTGCTGGTGACCCAATGCGACCAGATGCTCCACGCCCAGTCGCGTAC
CGTCTTCATGGGAGAAAATAATACTGTTGATGGGTGTCTGGTCAGAGACATCAAGAAATAAC
GCCGGAACATTAGTGCAGGCAGCTTCCACAGCAATGGCATCCTGGTCATCCAGCGGATAGTT
AATGATCAGCCCACTGACGCGTTGCGCGAGAAGATTGTGCACCGCCGCTTTACAGGCTTCGA
CGCCGCTTCGTTCTACCATCGACACCACCACGCTGGCACCCAGTTGATCGGCGCGAGATTTA
ATCGCCGCGACAATTTGCGACGGCGCGTGCAGGGCCAGACTGGAGGTGGCAACGCCAATCAG
CAACGACTGTTTGCCCGCCAGTTGTTGTGCCACGCGGTTGGGAATGTAATTCAGCTCCGCCA
TCGCCGCTTCCACTTTTTCCCGCGTTTTCGCAGAAACGTGGCTGGCCTGGTTCACCACGCGG
GAAACGGTCTGATAAGAGACACCGGCATACTCTGCGACATCGTATAACGTTACTGGTTTCAC
ATTCACCACCCTGAATTGACTCTCTTCCGGGCGCTATCATGCCATACCGCGAAAGGTTTTGC
GCCATTCGATGGTGTCCGGGATCTCGACGCTCTCCCTTATGCGACTCCTGCATTAGGAAGCA
GCCCAGTAGTAGGTTGAGGCCGTTGAGCACCGCCGCCGCAAGGAATGGTGCATGCCGGCATG
CCGCCCTTTCGTCTTCAAGAATTAATTCCCAATTCCCCAGGCATCAAATAAAACGAAAGGCT
CAGTCGAAAGACTGGGCCTTTCGTTTTATCTGTTGTTTGTCGGTGAACGCTCTCCTGAGTAG
GACAAATCCGCCGGGAGCGGATTTGAACGTTGCGAAGCAACGGCCCGGAGGGTGGCGGGCAG
GACGCCCGCCATAAACTGCCAGGAATTAATTCCCCAGGCATCAAATAAAACGAAAGGCTCAG
TCGAAAGACTGGGCCTTTCGTTTTATCTGTTGTTTGTCGGTGAACGCTCTCCTGAGTAGGAC
AAATCCGCCGGGAGCGGATTTGAACGTTGCGAAGCAACGGCCCGGAGGGTGGCGGGCAGGAC
GCCCGCCATAAACTGCCAGGAATTAATTCCCCAGGCATCAAATAAAACGAAAGGCTCAGTCG
AAAGACTGGGCCTTTCGTTTTATCTGTTGTTTGTCGGTGAACGCTCTCCTGAGTAGGACAAA
TCCGCCGGGAGCGGATTTGAACGTTGCGAAGCAACGGCCCGGAGGGTGGCGGGCAGGACGCC
CGCCATAAACTGCCAGGAATTAATTCCCCAGGCATCAAATAAAACGAAAGGCTCAGTCGAAA
GACTGGGCCTTTCGTTTTATCTGTTGTTTGTCGGTGAACGCTCTCCTGAGTAGGACAAATCC
GCCGGGAGCGGATTTGAACGTTGCGAAGCAACGGCCCGGAGGGTGGCGGGCAGGACGCCCGC
CATAAACTGCCAGGAATTAATTCCCCAGGCATCAAATAAAACGAAAGGCTCAGTCGAAAGAC
TGGGCCTTTCGTTTTATCTGTTGTTTGTCGGTGAACGCTCTCCTGAGTAGGACAAATCCGCC
GGGAGCGGATTTGAACGTTGCGAAGCAACGGCCCGGAGGGTGGCGGGCAGGACGCCCGCCAT
AAACTGCCAGGAATTGGGGATCGGAATTAATTCCCGGTTTAAACCGGGGATCTCGATCCCGC
GAAATTAATACGACTCACTATAGGGGAATTGTGAGCGGATAACAATTCCCCTCTAGAAATAA
TTTTGTTTAACTTTAAGAAGGAGATATACAT

Primer Oligonucleotide DNA Sequences

Fwd_T7 (sequencing primer):
TAATACGACTCACTATAGG

Rvs_T7 (sequencing primer):
GCTAGTTATTGCTCAGCGG

Fwd_pUC-PT7:
ATGATGACCGCCTAGTAACTCGAGTCTGGTAAAGAA

Rvs_pUC-PT7:
ATGGCTGCTGCCCATATGTATATCTCCTTCTTATACTTAACTAA

Fwd_His_6-MBP-TEV:
GAAGGAGATATACATATGGGCAGCAGCCA

Rvs_His_6-MBP-TEV:
GGTCAGCTGATCTGCGCCCTGGAAGTAAAGGTTTTC

Fwd_CaM:
CTTTACTTCCAGGGCGCAGATCAGCTGACC

Rvs_CaM:
ACCAGACTCGAGTTACTAGGCGGTCATCAT

Fwd_Ub:
ATACATATGCAGATCTTCGTG

Rvs_Ub-MxeSpe:
AATACTAGTGCATCTCCCGTGATGCAACCACCTCTGAGACGGAGTA

Fwd_K48C:
CTTTGCTGGGtgcCAGCTGGAAGATG

Rvs_K48C:
ATCAACCTCTGCTGGTCA

Calmodulin Protein Sequences

CaM (K148U) Diselenide 12 *(mutation underlined and bold)*

GADQLTEEQI AEFKEAFSLF DKDGDGTITT KELGTVMRSL
GQNPTEAELQ DMINEVDADG NGTIDFPEFL TMMARKMKDT
DSEEEIREAF RVFDKDGNGY ISAAELRHVM TNLGEKLTDE
EVDEMIREAD IDGDGQVNYE EFVQMMTA**U**

His$_6$-MBP-TEV-CaM (K148U) Construct

MGSSHHHHHH GTKTEEGKLV IWINGDKGYN GLAEVGKKFE
KDTGIKVTVE HPDKLEEKFP QVAATGDGPD IIFWAHDRFG
GYAQSGLLAE ITPDKAFQDK LYPFTWDAVR YNGKLIAYPI
AVEALSLIYN KDLLPNPPKT WEEIPALDKE LKAKGKSALM
FNLQEPYFTW PLIAADGGYA FKYENGKYDI KDVGVDNAGA
KAGLTFLVDL IKNKHMNADT DYSIAEAAFN KGETAMTING
PWAWSNIDTS KVNYGVTVLP TFKGQPSKPF VGVLSAGINA
ASPNKELAKE FLENYLLTDE GLEAVNKDKP LGAVALKSYE
EELAKDPRIA ATMENAQKGE IMPNIPQMSA FWYAVRTAVI
NAASGRQTVD EALKDAQTGT DYDIPTTENL YFQGADQLTE
EQIAEFKEAF SLFDKDGDGT ITTKELGTVM RSLGQNPTEA
ELQDMINEVD ADGNGTIDFP EFLTMMARKM KDTDSEEEIR
EAFRVFDKDG NGYISAAELR HVMTNLGEKL TDEEVDEMIR
EADIDGDGQV NYEEFVQMMT AU

Ubiquitin Protein Sequences

Ubiquitin

MQIFVKTLTG KTITLEVEPS DTIENVKAKI QDKEGIPPDQ
QRLIFAGKQL EDGRTLSDYN IQKESTLHLV LRLRGG

Ubiquitin (K48C) ***(mutation underlined and bold)***

MQIFVKTLTG KTITLEVEPS DTIENVKAKI QDKEGIPPDQ
QRLIFAG**C**QL EDGRTLSDYN IQKESTLHLV LRLRGG

Mxe Intein – His – CBD Construct

CITGDALVAL PEGESVRIAD IVPGARPNSD NAIDLKVLDR
HGNPVLADRL FHSGEHPVYT VRTVEGLRVT GTANHPLLCL
VDVAGVPTLL WKLIDEIKPG DYAVIQRSAF SVDCAGFARG
KPEFAPTTYT VGVPGLVRFL EAHHRDPDAQ AIADELTDGR
FYYAKVASVT DAGVQPVYSL RVDTADHAFI TNGFVSHATG
LTGIHHHHHH HSGLNSGLTT NPGVSAWQVN TAYTAGQLVT
YNGKTYKCLQ PHTSLAGWEP SNVPALWQLQ

Supplementary References

1 Kambanis, L., Chisholm, T. S., Kulkarni, S. S. & Payne, R. J. Rapid one-pot iterative diselenide–selenoester ligation using a novel coumarin-based photolabile protecting group. *Chem. Sci.* **12**, 10014-10021 (2021).

2 Mitchell, N. J. *et al.* Accelerated Protein Synthesis via One-Pot Ligation-Deselenization Chemistry. *Chem* **2**, 703-715 (2017).

3 Ford, D. J. *et al.* Potent Cyclic Peptide Inhibitors of FXIIa Discovered by mRNA Display with Genetic Code Reprogramming. *J. Med. Chem.* **64**, 7853-7876 (2021).

4 Thyer, R. *et al.* Custom selenoprotein production enabled by laboratory evolution of recoded bacterial strains. *Nat. Biotechnol.* **36**, 624-631 (2018).

5 Fisher, K. J., Alyea, E. C. & Shahnazarian, N. A ^{31}P NMR Study Of The Water Soluble Derivatives Of 1,3,5-Triaza-7-Phosphaadamantane (PTA). *Phosphorus Sulfur Silicon Relat. Elem.* **48**, 37-40 (1990).

6 Perdew, J. P. in *Electronic Structure of Solids '91*. (Akademie Verlag, Berlin, 1991).

7 Perdew, J. P. *et al.* Atoms, molecules, solids, and surfaces: Applications of the generalized gradient approximation for exchange and correlation. *Phys. Rev. B* **46**, 6671-6687 (1992).

8 Becke, A. D. Density-functional thermochemistry. III. The role of exact exchange. *J. Chem. Phys* **98**, 5648-5652 (1993).

9 Rassolov, V. A., Ratner, M. A., Pople, J. A., Redfern, P. C. & Curtiss, L. A. 6-31G* basis set for third-row atoms. *J. Comput. Chem.* **22**, 976-984 (2001).

10 Francl, M. M. *et al.* Self-consistent molecular orbital methods. XXIII. A polarization-type basis set for second-row elements. *J. Chem. Phys.* **77**, 3654-3665 (1982).

11 Hariharan, P. C. & Pople, J. A. The influence of polarization functions on molecular orbital hydrogenation energies. *Theor. Chim. Acta* **28**, 213-222 (1973).

12 Hehre, W. J., Ditchfield, R. & Pople, J. A. Self—Consistent Molecular Orbital Methods. XII. Further Extensions of Gaussian—Type Basis Sets for Use in Molecular Orbital Studies of Organic Molecules. *J. Chem. Phys.* **56**, 2257-2261 (1972).

13 Clark, T., Chandrasekhar, J., Spitznagel, G. W. & Schleyer, P. V. R. Efficient diffuse function-augmented basis sets for anion calculations. III. The 3-21+G basis set for first-row elements, Li–F. *J. Comput. Chem.* **4**, 294-301 (1983).

14 Weigend, F. Accurate Coulomb-fitting basis sets for H to Rn. *Phys. Chem. Chem. Phys.* **8**, 1057-1065 (2006).

15 Weigend, F. & Ahlrichs, R. Balanced basis sets of split valence, triple zeta valence and quadruple zeta valence quality for H to Rn: Design and assessment of accuracy. *Phys. Chem. Chem. Phys.* **7**, 3297-3305 (2005).

16 Grimme, S., Antony, J., Ehrlich, S. & Krieg, H. A consistent and accurate ab initio parametrization of density functional dispersion correction (DFT-D) for the 94 elements H-Pu. *J. Chem. Phys.* **132**, 154104 (2010).

17 Grimme, S., Ehrlich, S. & Goerigk, L. Effect of the damping function in dispersion corrected density functional theory. *J. Comput. Chem.* **32**, 1456-1465 (2011).

18 Pearson, J. K., Ban, F. & Boyd, R. J. An Evaluation of Various Computational Methods for the Treatment of Organoselenium Compounds. *J. Chem. Phys. A* **109**, 10373-10379 (2005).

19 Heverly-Coulson, G. S. & Boyd, R. J. Systematic study of the performance of density functional theory methods for prediction of energies and geometries of organoselenium compounds. *J. Chem. Phys. A* **115**, 4827-4831 (2011).

20 Marenich, A. V., Cramer, C. J. & Truhlar, D. G. Universal Solvation Model Based on

Solute Electron Density and on a Continuum Model of the Solvent Defined by the Bulk Dielectric Constant and Atomic Surface Tensions. *J. Chem. Phys. B* **113**, 6378-6396 (2009).
21 Cancès, E., Mennucci, B. & Tomasi, J. A new integral equation formalism for the polarizable continuum model: Theoretical background and applications to isotropic and anisotropic dielectrics. *J. Chem. Phys.* **107**, 3032-3041 (1997).
22 Mennucci, B., Cancès, E. & Tomasi, J. Evaluation of Solvent Effects in Isotropic and Anisotropic Dielectrics and in Ionic Solutions with a Unified Integral Equation Method: Theoretical Bases, Computational Implementation, and Numerical Applications. *J. Chem. Phys. B* **101**, 10506-10517 (1997).
23 Scalmani, G. & Frisch, M. J. Continuous surface charge polarizable continuum models of solvation. I. General formalism. *J. Chem. Phys.* **132**, 114110 (2010).
24 Tomasi, J., Mennucci, B. & Cancès, E. The IEF version of the PCM solvation method: an overview of a new method addressed to study molecular solutes at the QM ab initio level. *J. Mol. Struct.-Theochem.* **464**, 211-226, (1999).
25 Mennucci, B. & Tomasi, J. Continuum solvation models: A new approach to the problem of solute's charge distribution and cavity boundaries. *J. Chem. Phys.* **106**, 5151-5158 (1997).
26 Downs, R. T. & Hall-Wallace, M. The American Mineralogist crystal structure database. *Am. Mineral.* **88**, 247-250 (2003).
27 Gražulis, S. *et al.* Crystallography Open Database - an open-access collection of crystal structures. *J. Appl. Crystallogr.* **42**, 726-729 (2009).
28 Hua, G., Fuller, A. L., Slawin, A. M. Z. & Woollins, J. D. 1,2-Bis(2-bromobenzyl)diselane. *Acta Crystallogr. Sect. E* **66**, o2579 (2010).
29 Bungu, P. N. & Otto, S. 2-Isobutyl-2-phosphabicyclo-[3.3.1]nonane 2-selenide. *Acta Crystallogr. Sect. E Struct. Rep. Online* **65**, o560-561 (2009).
30 Alegre-Requena, J. V., Sowndarya, S., Pérez-Soto, R., Alturaifi, T. M. & Paton, R. S. AQME v1.0. (2022), https://github.com/jvalegre/aqme.
31 Supporting Computational Data: Site-Selective Photocatalytic Functionalization of Peptides and Proteins at Selenocysteine. Zenodo. DOI: 10.5281/zenodo.7224862.
32 Luchini, G., Alegre-Requena, J., Funes-Ardoiz, I. & Paton, R. GoodVibes: automated thermochemistry for heterogeneous computational chemistry data. *F1000Research* **9**, 291 (2020).
33 Fukui, K. The path of chemical reactions - the IRC approach. *Acc. Chem. Res.* **14**, 363-368 (1981).
34 M. J. Frisch *et al.* Gaussian 16, Revision C.01. *Gaussian 16, Revision C.01, Gaussian, Inc., Wallingford, CT* (2016).
35 Paton, R. S. Pymol_style. gist.github.com/bobbypaton (2022).
36 Grimme, S. Supramolecular Binding Thermodynamics by Dispersion-Corrected Density Functional Theory. *Chem. Eur. J.* **18**, 9955-9964 (2012).
37 Bryantsev, V. S., Diallo, M. S. & Goddard Iii, W. A. Calculation of Solvation Free Energies of Charged Solutes Using Mixed Cluster/Continuum Models. *J. Phys. Chem. B* **112**, 9709-9719 (2008).
38 Sure, R. & Grimme, S. Comprehensive Benchmark of Association (Free) Energies of Realistic Host–Guest Complexes. *J. Chem. Theory Comput.* **11**, 3785-3801 (2015).
39 Bailey, W. F. & Monahan, A. S. Statistical effects and the evaluation of entropy differences in equilibrium processes. Symmetry corrections and entropy of mixing. *J. Chem. Ed.* **55**, 489 (1978).
40 Plata, R. E. & Singleton, D. A. A Case Study of the Mechanism of Alcohol-Mediated Morita Baylis–Hillman Reactions. The Importance of Experimental Observations. *J.*

Am. Chem. Soc. **137**, 3811-3826 (2015).
41 Patchkovskii, S. Brute Force Symmetry Analyzer (1996, 2003).
42 Groom, C. R., Bruno, I. J., Lightfoot, M. P. & Ward, S. C. The Cambridge Structural Database. *Acta Crystallogr. Sect. B* **72**, 171-179 (2016).
43 Cordero, B. *et al.* Covalent radii revisited. *Dalton Trans.*, 2832-2838 (2008).
44 Marcus, R. A. On the Theory of Oxidation-Reduction Reactions Involving Electron Transfer. I. *J. Chem. Phys.* **24**, 966-978 (1956).
45 Marcus, R. A. Electron Transfer Reactions in Chemistry: Theory and Experiment (Nobel Lecture). *Angew. Chem. Int. Ed.* **32**, 1111-1121 (1993).
46 Jones, G. O., Liu, P., Houk, K. N. & Buchwald, S. L. Computational Explorations of Mechanisms and Ligand-Directed Selectivities of Copper-Catalyzed Ullmann-Type Reactions. *J. Am. Chem. Soc.* **132**, 6205-6213 (2010).
47 Vaissier, V., Barnes, P., Kirkpatrick, J. & Nelson, J. Influence of polar medium on the reorganization energy of charge transfer between dyes in a dye sensitized film. *Phys. Chem. Chem. Phys.* **15**, 4804-4814 (2013).
48 Value taken from the Sigma-Aldrich vendor with acetonitrile gradient grade, >99.9%. www.sigmaaldrich.com.
49 University of California Los Angeles. Solvent. www.chem.ucla.edu/~bacher/General/30BL/tips/solvent.html (2022).
50 López-Estrada, O., Laguna, H. G., Barrueta-Flores, C. & Amador-Bedolla, C. Reassessment of the Four-Point Approach to the Electron-Transfer Marcus–Hush Theory. *ACS Omega* **3**, 2130-2140 (2018).
51 Berkeley Madonna, version 9.1.3 (University of California, Berkeley, CA, 2018).
52 Sonsona, I. G., Alegre-Requena, J. V., Marqués-López, E., Gimeno, M. C. & Herrera, R. P. Asymmetric Organocatalyzed Aza-Henry Reaction of Hydrazones: Experimental and Computational Studies. *Chem. Eur. J.* **26**, 5469-5478 (2020).
53 Rush, L. E., Pringle, P. G. & Harvey, J. N. Computational Kinetics of Cobalt-Catalyzed Alkene Hydroformylation. *Angew. Chem. Int. Ed.* **53**, 8672-8676 (2014).
54 Aragao, D. *et al.* MX2: a high-flux undulator microfocus beamline serving both the chemical and macromolecular crystallography communities at the Australian Synchrotron. *J. Synchrotron Radiat.* **25**, 885-891 (2018).
55 Winn, M. D. *et al.* Overview of the CCP4 suite and current developments. *Acta Crystallogr. D Biol. Crystallogr.* **67**, 235-242 (2011).
56 Potterton, E., Briggs, P., Turkenburg, M. & Dodson, E. A graphical user interface to the CCP4 program suite. *Acta Crystallogr. D Biol. Crystallogr.* **59**, 1131-1137 (2003).
57 Emsley, P., Lohkamp, B., Scott, W. G. & Cowtan, K. Features and development of Coot. *Acta Crystallogr. D Biol. Crystallogr.* **66**, 486-501 (2010).
58 Adams, P. D. *et al.* PHENIX: a comprehensive Python-based system for macromolecular structure solution. *Acta Crystallogr. D Biol. Crystallogr.* **66**, 213-221 (2010).
59 Kerdraon, F. *et al.* Insights into the Mechanism and Catalysis of Peptide Thioester Synthesis by Alkylselenols Provide a New Tool for Chemical Protein Synthesis. *Molecules* **26**, 1386 (2021).
60 Guenther, W. H. H. Methods in selenium chemistry. III. Reduction of diselenides with dithiothreitol. *J. Org. Chem.* **32**, 3931-3933 (1967).
