# Supplementary material for: Site-selective photocatalytic functionalization of peptides and proteins at selenocysteine
Source: Nat Commun. 2022 Nov 12;13:6885. doi: 10.1038/s41467-022-34530-z (PMC9653470; doi:10.1038/s41467-022-34530-z)
Supplement: Supplementary file 2 — Description of Additional Supplementary Files [file 41467_2022_34530_MOESM2_ESM.pdf]

Description of Additional Supplementary Files

Supplementary Data 1: Thermochemical data

Supplementary Data 2: Molecular coordinates from computational mechanistic studies
