# Supplementary material for: Site-selective photocatalytic functionalization of peptides and proteins at selenocysteine
Source: Nat Commun. 2022 Nov 12;13:6885. doi: 10.1038/s41467-022-34530-z (PMC9653470; doi:10.1038/s41467-022-34530-z)
Supplement: Supplementary file 5 — Reporting Summary [file 41467_2022_34530_MOESM5_ESM.pdf]

nature portfolio

Corresponding author(s): Richard J. Payne

Last updated by author(s): Oct 26, 2022

Reporting Summary

Nature Portfolio wishes to improve the reproducibility of the work that we publish. This form provides structure for consistency and transparency in reporting. For further information on Nature Portfolio policies, see our Editorial Policies and the Editorial Policy Checklist.

Statistics

For all statistical analyses, confirm that the following items are present in the figure legend, table legend, main text, or Methods section.

n/a	Confirmed	
x	☐	The exact sample size (n) for each experimental group/condition, given as a discrete number and unit of measurement
x	☐	A statement on whether measurements were taken from distinct samples or whether the same sample was measured repeatedly
x	☐	The statistical test(s) used AND whether they are one- or two-sided<br>*Only common tests should be described solely by name; describe more complex techniques in the Methods section.*
x	☐	A description of all covariates tested
x	☐	A description of any assumptions or corrections, such as tests of normality and adjustment for multiple comparisons
x	☐	A full description of the statistical parameters including central tendency (e.g. means) or other basic estimates (e.g. regression coefficient) AND variation (e.g. standard deviation) or associated estimates of uncertainty (e.g. confidence intervals)
x	☐	For null hypothesis testing, the test statistic (e.g. F, t, r) with confidence intervals, effect sizes, degrees of freedom and P value noted<br>*Give P values as exact values whenever suitable.*
x	☐	For Bayesian analysis, information on the choice of priors and Markov chain Monte Carlo settings
x	☐	For hierarchical and complex designs, identification of the appropriate level for tests and full reporting of outcomes
x	☐	Estimates of effect sizes (e.g. Cohen's d, Pearson's r), indicating how they were calculated

Our web collection on statistics for biologists contains articles on many of the points above.

Software and code

Policy information about availability of computer code

Data collection	Diffraction data was collected at the Australian Synchrotron MX2 beamline using the Eiger detector.
Data analysis	Data was analyzed using Prism 9 (version 9.3.1), Microsoft Excel 2017 and MestReNova14.2. Diffraction data was scaled and merged with AIMLESS from the CCP4 (implemented in CCP4 7.1.010). Structures were solved using PhaserMR. The model was built using COOT (version 0.8.6.1), followed by refinement using Phenix (implemented in CCP4 7.1.010). Thermochemical data was obtained using GoodVibes version 3.0.0. (DOI: 10.5281/zenodo.3346166).

For manuscripts utilizing custom algorithms or software that are central to the research but not yet described in published literature, software must be made available to editors and reviewers. We strongly encourage code deposition in a community repository (e.g. GitHub). See the Nature Portfolio guidelines for submitting code & software for further information.

Data

Policy information about availability of data

All manuscripts must include a data availability statement. This statement should provide the following information, where applicable:

- Accession codes, unique identifiers, or web links for publicly available datasets
- A description of any restrictions on data availability
- For clinical datasets or third party data, please ensure that the statement adheres to our policy

The quality of the final model for Calmodulin (K148U) Selenoether 18 was validated using wwPBD server and deposited to the PDB (ID: 7T2Q). Thermochemical data (Supplementary Data 1) and molecular coordinates from computational mechanistic studies (Supplementary Data 2), and the PDB validation report for 7T2Q (http://www.rcsb.org/structure/7T2Q) are provided with this manuscript as Supplementary Data Sets. The thermochemical data (obtained with the GoodVibes software version 3.0.0, https://github.com/bobbypaton/GoodVibes) and molecular coordinates from computational mechanistic studies are also available in Zenodo (https://

doi.org/10.5281/zenodo.7224862). The mass spectrometry proteomics data have been deposited to the ProteomeXchange Consortium via the PRIDE partner repository with the dataset identifier PXD037525 (http://www.ebi.ac.uk/pride/archive/projects/PXD037525). Data is available from the corresponding authors upon request.

Field-specific reporting

Please select the one below that is the best fit for your research. If you are not sure, read the appropriate sections before making your selection.

- [x] Life sciences
- [ ] Behavioural & social sciences
- [ ] Ecological, evolutionary & environmental sciences

For a reference copy of the document with all sections, see nature.com/documents/nr-reporting-summary-flat.pdf

Life sciences study design

All studies must disclose on these points even when the disclosure is negative.

Sample size	Reactions were performed with a sample size of n = 1 and reaction conversions were calculated through averaged integrations of the three most abundant ion peaks in the HRMS spectrum.
Data exclusions	No data was excluded.
Replication	For protein-based PDC reactions, conversions were calculated through integration of extracted ion chromatograms for the three most abundant ion peaks of each protein related mass envelope. This data was collected from a single experiment. All attempts at replication were successful.
Randomization	No studies or experiments were randomized.
Blinding	No studies or experiments were blinded.

Reporting for specific materials, systems and methods

We require information from authors about some types of materials, experimental systems and methods used in many studies. Here, indicate whether each material, system or method listed is relevant to your study. If you are not sure if a list item applies to your research, read the appropriate section before selecting a response.

Materials & experimental systems

n/a	Involved in the study	
☒	☐	Antibodies
☒	☐	Eukaryotic cell lines
☒	☐	Palaeontology and archaeology
☒	☐	Animals and other organisms
☐	☒	Human research participants
☒	☐	Clinical data
☒	☐	Dual use research of concern

Methods

n/a	Involved in the study	
☒	☐	ChIP-seq
☒	☐	Flow cytometry
☒	☐	MRI-based neuroimaging

Human research participants

Policy information about studies involving human research participants

Population characteristics	Human donor blood samples were obtained with written informed consent, and all participants confirmed devoid of any anti-platelet medication for the preceding 2 weeks and were moreover free from any history of bleeding disorder, anemia, renal impairment, and acute coronary syndrome or coronary intervention (<30 days). The donor cohort was made up of approximately equal numbers of male and female donors between the age range of 18-60.
Recruitment	Human plasma was isolated from blood samples collected from healthy volunteers employed by the Heart Research Institute (Newtown, NSW, Australia)
Ethics oversight	All procedures involving the collection of blood from healthy donors were approved by the University of Sydney Human Research Ethics Committee (HREC, Project 2014/244) and all studies conformed to the principles outlined in the Declaration of Helsinki.

Note that full information on the approval of the study protocol must also be provided in the manuscript.
